# Palladium-Catalyzed Alkoxycarbonylation of Alcohols for the Synthesis of Cyclobutanecarboxylates with α-Quaternary Carbon Centers

**DOI:** 10.1021/acs.orglett.5c00087

**Published:** 2025-01-27

**Authors:** Yu-Kun Liu, Xing-Wei Gu, Xiao-Feng Wu

**Affiliations:** † Leibniz-Institut für Katalyse e. V., Albert-Einstein-Straße 29a, 18059 Rostock, Germany; ‡ Dalian National Laboratory for Clean Energy, Dalian Institute of Chemical Physics, Chinese Academy of Sciences, Dalian 116023, Liaoning, China

## Abstract

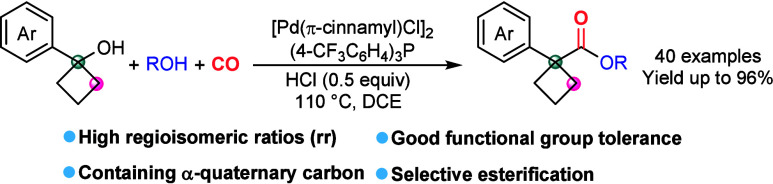

A palladium-catalyzed alkoxycarbonylation with two different alcohols for the synthesis of cyclobutanecarboxylates bearing an α-quaternary carbon center is presented. The reaction utilizes readily accessible starting materials, tolerates a broad scope of functional groups, and provides a straightforward and efficient approach for the synthesis of a diverse array of cyclobutanecarboxylates bearing an α-quaternary carbon. Meanwhile, this strategy effectively prevents the transition-metal-catalyzed ring-opening of cyclobutanols, preserves the cyclobutane framework, and affords 1,1-disubstituted cyclobutanecarboxylates in high yields with excellent regioisomeric ratios.

Cyclobutanecarboxylates are a vital class of compounds, widely found in biological molecules and natural products, and is also extensively used in organic synthesis (Scheme 1a).^1^ Characterized by the cyclobutane motif as the core structural element, significant attention has been directed toward the synthesis of its substituted derivatives, fueled by ongoing advancements in the methodological development.^2^ For instance, the [2 + 2] cycloaddition of olefins directly produces diverse multisubstituted cyclobutane conformations.^3^ Nevertheless, heterodimerization of olefins encounters major challenges, such as restricted parent olefin compatibility and competing homodimerization reactions.^4^ Meanwhile, the direct modification of compounds containing a cyclobutane framework, as exemplified by bicyclo [1.1.0] butanes (BCBs), has proven to be an effective strategy for synthesizing multisubstituted cyclobutanes.^5^ However, the challenging synthesis process and high reactivity restrict the application of BCBs in such reactions.^6^

**Scheme 1:**
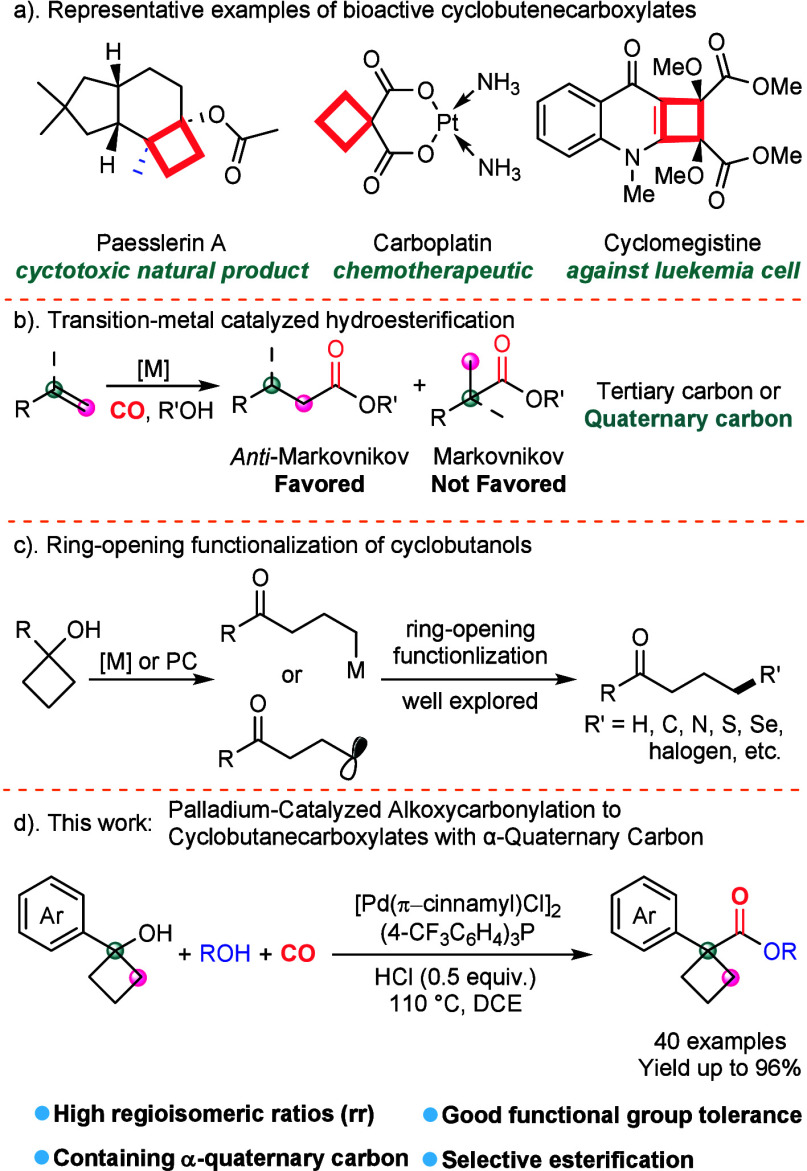
Transition-Metal-Catalyzed Hydroesterification and Ring-Opening Functionalization of Cyclobutanols

Alkoxycarbonylation represents a broadly employed reaction in organic synthesis serving as an effective method for the direct formation of esters^7^ in which the transition-metal-catalyzed carbonylation reaction can be utilized to achieve efficient alkoxycarbonylation. It is distinguished by its versatility and the use of carbon monoxide (CO) as a cost-effective and abundant source of C1.^8^ Recently, Pd-catalyzed Markovnikov hydroesterification approaches have been introduced for the synthesis of branched esters, but the reported methods are limited to monosubstituted alkenes for the formation of α-tertiary esters.^9^ Due to the steric effects and Keuleman’s rule,^10^ the Markovnikov hydroesterification of alkenes catalyzed by transition metals is not preferred (Scheme 1b).^11^ Moreover, the Koch–Haaf reaction^12^ typically requires strong mineral acids (H_2_SO_4_ or a/mix of HF/BF_3_) in overstoichiometric amounts and under harsh reaction conditions, which makes it particularly challenging to process substrates with strained ring structures.^13^ On the other hand, the synthesis of esters containing α-quaternary carbon-linked structures is of great significance in research.

Cyclobutanols are important scaffolds that are commonly employed in ring-opening reactions involving β-carbon elimination,^14^ or radical ring-opening,^15^ catalyzed by either transition metals or visible light though the cleavage of the cyclic C–C bonds, to generate γ-substituted ketones (Scheme 1c).^16^ However, the synthesis of cyclobutanecarboxylates containing an α-quaternary carbon center remains exceptionally challenging, requiring the preservation of the cyclobutane motif throughout the reaction process. In this context, the successful development of transition-metal-catalyzed alkoxycarbonylation of cyclobutanols to 1,1-disubstituted cyclobutanecarboxylates would provide an ideal method to produce esters with α-quaternary carbon. Herein, we report a novel palladium-catalyzed alkoxycarbonylation of cyclobutanols to 1,1-disubstituted cyclobutanecarboxylates (Scheme 1d).

To initiate this work, cyclobutanol **1a** and 3-phenyl-1-propanol **2a** were selected as substrates for the model reaction (Table 1). Employing Pd(CH_3_CN)_2_Cl_2_ as the catalyst, PPh_3_ as the ligand, and HCl as an additive in DCE at 110 °C, the reaction produced the target product **3a** in moderate yield, accompanied by the formation of the 1,2-disubstituted byproduct **4a** (Table 1, entry 1). Changing ligand to **L4**, which contains a −CF_3_ group that reduces the electron density on the benzene ring, the yield increases to 83% and enhances the regioisomeric ratios (*rr*) (Table 1, entry 2). In contrast, **L5** afforded the desired product **3a** with low activity, albeit with high regioisomeric ratios (Table 1, entry 3). Other monodentate phosphine ligands tested in this study demonstrated low reactivity or insufficient regioisomeric ratios (Table 1, entries 4–6). Among the bisphosphine ligands tested, DPEphos shows no reactivity, while Xantphos and Nixantphos promote the formation of **4a** under identical conditions (Table 1, entries 7–9). Furthermore, the corresponding product was not generated when dppf, dppp, or bpy was employed as the ligand (Table 1, entry 10). The reaction concentration also significantly influences the yield (Table 1, entries 11–12). By increasing the concentration and extending the reaction time, the reaction can afford the target product with a 94% isolated yield and 18:1 regioisomeric ratios (Table 1, entry 14). Notably, no byproducts related with the ring opening of cyclobutanol could be detected which usually go through a radical mechanism.^15^

**Table 1:**
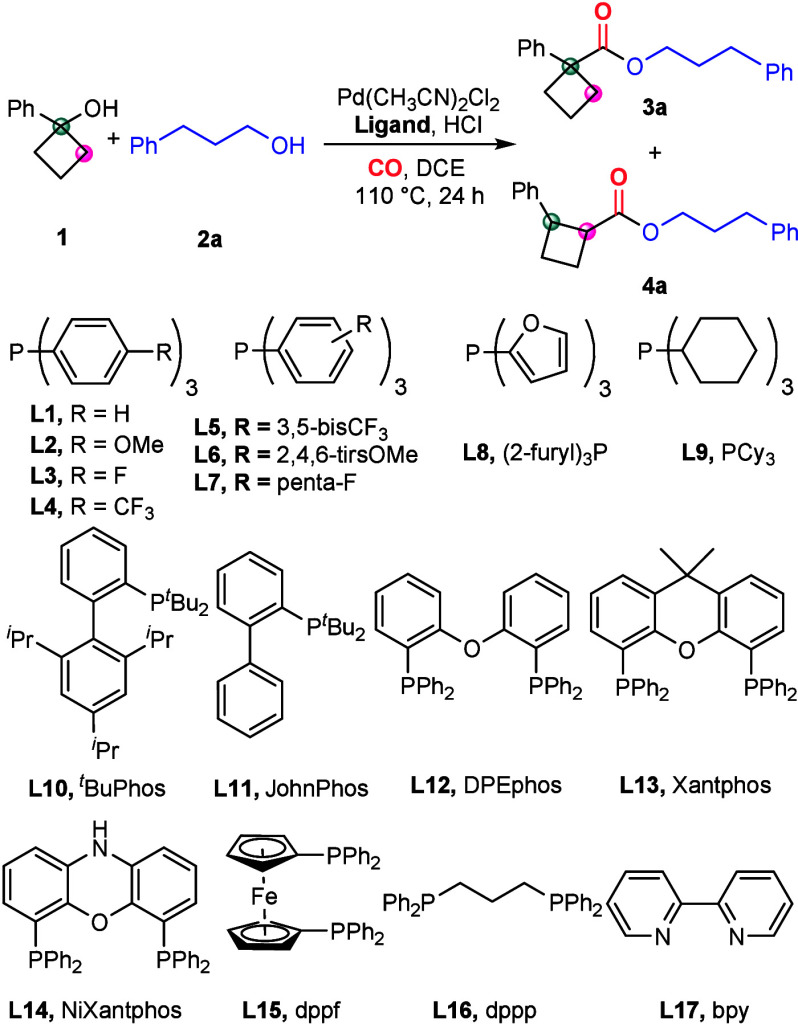
Optimization of the Reaction Conditions^a^^,^^b^

entry	ligand	**3a** (%)	*rr* (3a:4a)
1	**L1–L3**	51–54	10–18:1
2	**L4**	83 (80)^c^	19:1
3	**L5**	11	>20:1
4	**L6, L7**	ND	-
5	**L8**	4	8:1
6	**L9–L11**	2–16	>20:1
7	**L12**	ND	-
8	**L13**	ND (63)^d^	<1:20
9	**L14**	ND (61)^d^	1:12
10	**L15–L17**	ND	-
11^e^	**L4**	50	19:1
12^f^	**L4**	85	>20:1
13^g^	**L4**	94 (89)^c^	16:1
14^g^^,^^h^	**L4**	96 (94)^c^	18:1

a Unless otherwise noted, the reactions were performed with Pd(CH_3_CN)_2_Cl_2_ (3 mol %), bisphophine ligand (3 mol %) or monophophine ligand (6 mol %), 0.5 equiv HCl (4.0 M in dioxane), **1a** (0.12 mmol), **2a** (0.1 mmol), CO (40 bar), and DCE (0.1 mL) at 110 °C for 24 h.

b The yields and regioisomeric ratios (*rr*) were determined by GC and GC-MS with *n*-hexadecane as the internal standard.

c Isolated yield of **3a**.

d The GC yield of **4a**.

e 2.0 mL DCE.

f 0.5 mL DCE.

g Pd(CH_3_CN)_2_Cl_2_ (3 mol %), (4-CF_3_C_6_H_4_)_3_P (6 mol %), 0.5 equiv HCl (4 M in 1,4-dioxane), **1a** (0.24 mmol), **2a** (0.2 mmol), CO (40 bar), and DCE (0.7 mL) at 110 °C for 36 h.

h [Pd(π-cinnamyl)Cl]_2_ (1.5 mol %) as catalyst.

Under the optimized conditions, the potential range of cyclobutyl alcohols and alcohols was explored, as illustrated in Scheme 2. The steric effect on the reaction was evaluated using cyclobutanols containing an *ortho*-, *meta*-, or *para*-methyl group on the benzene ring (**3b**–**3d**). When *ortho*-substituted cyclobutanol was used as the substrate, both the *rr* and the yield of the corresponding product decreased significantly (**3c**). Cyclobutanols bearing *para*-position halides and *meta*-methylthio were smoothly converted to the desired products with good yields and excellent *rr* (**3e**–**3h**). Additionally, the *meta*-isobutoxyphenyl-substituted cyclobutanol can also be successfully transformed into the targeted product (**3i**). The reaction also demonstrates excellent compatibility with heterocycles such as thiophene, benzothiophene, dibenzothiophene, benzofuran, and dibenzofuran, all of which were obtained in satisfactory yields and outstanding *rr* (**3j**–**3n**). Moreover, fluorocyclobutanols also afforded the target product with a high *rr* (**3o**–**3p**). Furthermore, this method was successfully extended to multisubstituted cyclobutanol, producing the desired product in moderate yield (**3r**).

**Scheme 2:**
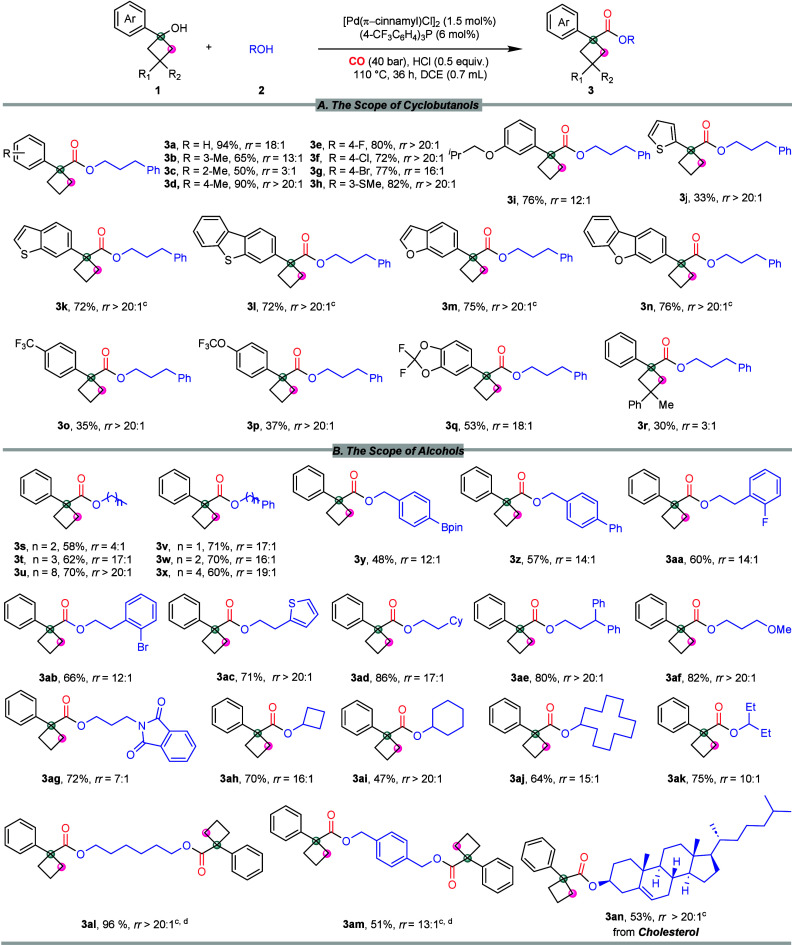
Scope of Cyclobutanols and Alcohols The reactions were performed with [Pd(π-cinnamyl)Cl]_2_ (1.5 mol %), (4-CF_3_C_6_H_4_)_3_P (6 mol %), 0.5 equiv HCl (4.0 M in dioxane), **1** (0.24 mmol), **2** (0.2 mmol), CO (40 bar), and DCE (0.7 mL) at 110 °C for 36 h. The regioisomeric ratios (*rr*) were determined by GC and GC-MS with *n*-hexadecane as the internal standard. Regioisomeric ratios (*rr*) were determined by ^1^H NMR of crude products. Condition for the substrates of double sites: [Pd(π-cinnamyl)Cl]_2_ (1.5 mol %), (4-CF_3_C_6_H_4_)_3_P (6 mol %), 0.5 equiv HCl (4.0 M in dioxane), **1** (0.24 mmol), **2** (0.1 mmol), CO (40 bar), and DCE (0.7 mL) at 110 °C for 36 h.

Subsequently, a series of aliphatic alcohols, including ethanol, propanol, 1-octanol, benzyl alcohol, 2-phenylethanol, and 4-phenylbutanol, were effectively converted into the corresponding cyclobutanecarboxylates, with respectable yields (**3s**–**3x**). Remarkably, the oxidant-sensitive −Bpin group demonstrated good tolerance, delivering the desired product (**3y**). In general, various functional groups, including −Ph, −F, and −Cl, on the aromatic rings of alcohols showed excellent compatibility with this protocol (**3z**–**3ab**). Specifically, substrates bearing thiophene, cyclohexane, diphenyl, methoxy, and phthalimido substituents afforded products **3ac**–**3ag** in 71–86% yields with moderate to high *rr*. Interestingly, secondary alcohols can also be successfully utilized in this procedure, delivering cyclobutanecarboxylates **3ah**–**3ak** in synthetically relevant yields. Afterward, several diols are employed in this reaction, each leading to the formation of a disubstituted product (**3al**, **3am**). Notably, the successful conversion of cholesterol into the corresponding product in the reaction confirms its compatibility with bioactive molecules (**3an**).

Next, we investigated the reaction priorities of different alcohols by simultaneously introducing 1-octanol (**2u**) and 3-pentanol (**2ak**) into the reaction. The result demonstrated that the reaction predominantly produced products derived from the primary alcohol (Scheme 3a). Then, site-selective experiments were conducted using 1,2-propanediol (**2ao**) as a substrate in the reaction. The reaction exclusively generates compounds at the primary alcohol site (Scheme 3b). Here, the coordination of diol with palladium influenced the coordination of phosphine with the metal center and subsequently decreased the regioselectivity.

**Scheme 3:**
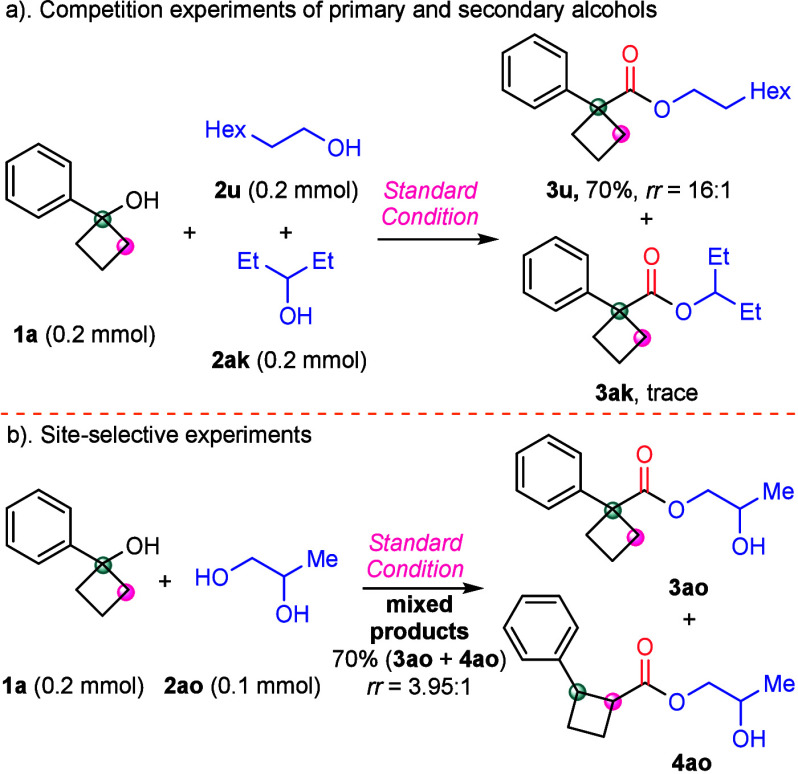
Competition Experiments

To further evaluate the practicality of the reaction, we conducted a large-scale experiment and several synthetic transformations (Scheme 4). Under standard conditions, the corresponding product was obtained with 74% yield and *rr* exceeding 20:1 (Scheme 4a). Additionally, the resulting cyclobutanecarboxylates can be smoothly transformed into a variety of cyclobutene derivatives (Scheme 4b). At ambient temperature, **3a** undergoes reduction with LiAlH_4_ to afford alcohol **4** in 80% yield. Furthermore, **3a** can be hydrolyzed under the appropriate condition to provide the corresponding acid **5** in 84% yield.

**Scheme 4:**
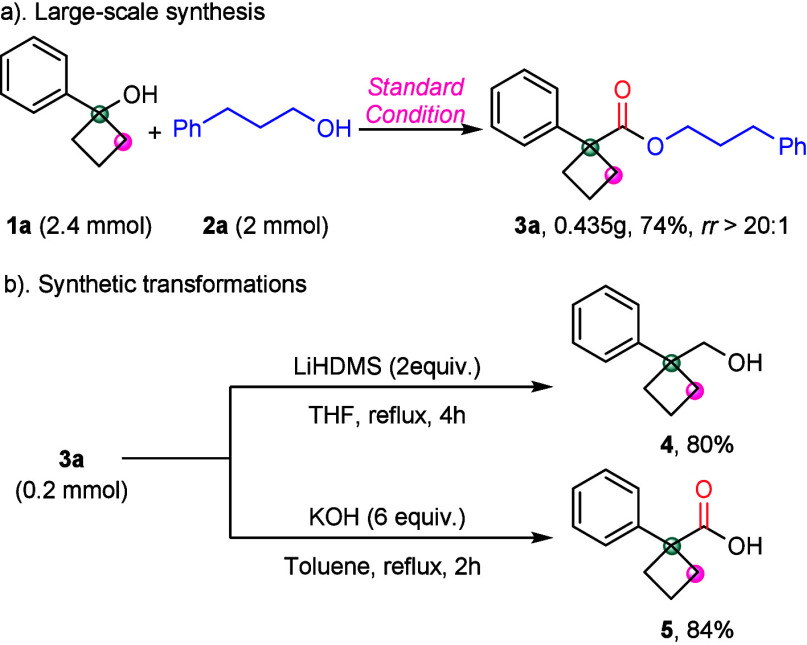
Synthesis Applicability

To further understand the mechanism, some mechanistic investigations were performed (Scheme 5). The reaction proceeds efficiently in the presence of radical scavengers (Scheme 5a). These radical quenching experiments suggest that the reaction does not proceed through a radical mechanism. Moreover, a verification experiment has confirmed that cyclobutene is a key intermediate in this reaction (Scheme 5b).

**Scheme 5:**
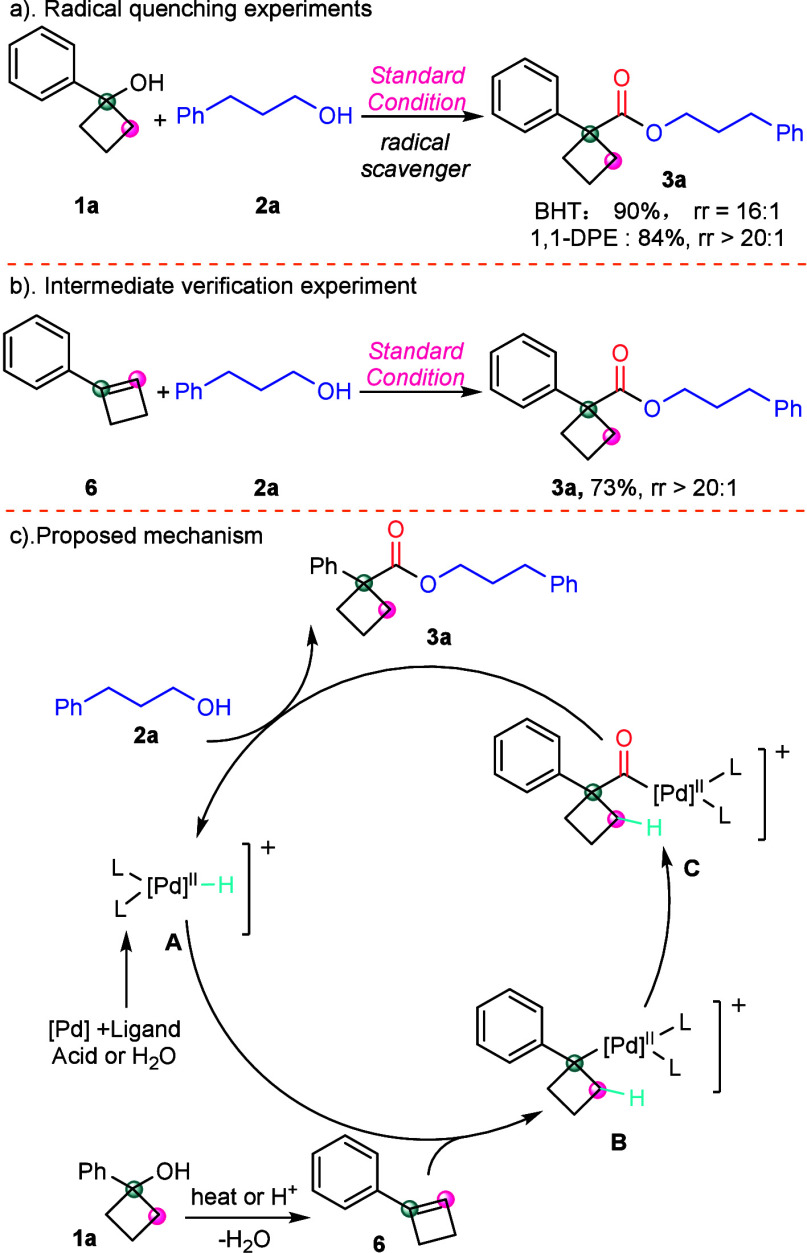
Mechanistic Studies

Based on the experimental results presented above and in the literature,^17^ a plausible reaction mechanism is proposed (Scheme 5c). Initially, under heating or in the presence of acid, cyclobutanol was transformed into cyclobutene. Concurrently, the palladium catalyst reacted with the ligand and either H_2_O or acid to generate a Pd–H complex **A**, which subsequently interacts with cyclobutene to form the 1,1-disubstituted alkyl-palladium intermediate **B**; carbon monoxide was then incorporated into intermediate **B**, yielding intermediate **C**. Ultimately, the acetyl complex **C** reacts with alcohol, leading to the formation of the target product, meanwhile regenerating Pd–H complex **A** to participate in the subsequent catalytic cycles.

In conclusion, a palladium-catalyzed alkoxycarbonylation of alcohols has been developed for the synthesis of cyclobutanecarboxylates that simultaneously exhibit a strained ring structure and a quaternary carbon center. This method strategically utilizes two distinct alcohols, including cyclobutyl alcohol, as the key starting materials. It achieves high efficiency and outstanding regioisomeric ratios while effectively preventing the transition-metal-catalyzed ring opening of the cyclobutanol framework. Notably, the reaction demonstrated a broad substrate scope and exceptional functional group compatibility. The potential for practical applications is evidenced by the large-scale synthesis and compatibility with bioactive molecules.

## Data Availability

The data underlying this study are available in the published article and its Supporting Information.

## Supporting Information Available

General comments, general procedure, optimization details, analytic data, and NMR spectra (PDF)
