# Supplementary material for: Palladium-Catalyzed Alkoxycarbonylation of Alcohols for the Synthesis of Cyclobutanecarboxylates with α-Quaternary Carbon Centers
Source: Org Lett. 2025 Jan 27;27(5):1316–21. doi: 10.1021/acs.orglett.5c00087 (PMC11811996; doi:10.1021/acs.orglett.5c00087)
Supplement: Supplementary file 1 — ol5c00087_si_001.pdf [file ol5c00087_si_001.pdf]

Supporting Information

Palladium-Catalyzed Alkoxycarbonylation of Alcohols for the Synthesis of Cyclobutanecarboxylates with α-Quaternary Carbon Center

Yu-Kun Liu[a], Xing-Wei Gu[a], Xiao-Feng Wu[a,b]*

[a]Leibniz-Institut fur Katalyse e. V., Albert-Einstein-Straße 29a, 18059 Rostock, Germany. E-mail: xiao-feng.wu@catalysis.de
[b]Dalian National Laboratory for Clean Energy, Dalian Institute of Chemical Physics, Chinese Academy of Sciences, Dalian, 116023, Liaoning, China. E-mail: xwu2020@ dicp.ac.cn

Content

1.General information S2
2. Optimization of Reaction Conditions S3
3. General procedure for the preparation of cyclobutanols...S7
   3.1 List of cycobutanols S7
   3.2 Typical procedure A..S7
   3.3 Typical procedure B S7
4.General procedure for the synthesis of Cyclobutanecarboxylate S8
5. Spectroscopic Data of Products..S9
6.Synthetic transformations of 3a..S18
   6.1 Reduction of compound 3a...S18
   6.2 Hydrolysis of compound 3a..S18
7.Reference..S19
8. NMR Spectra...S20

1.General information

Reagents and solvents: Unless otherwise noted, reagents were ordered from *Sigma-Aldrich, TCI, ABCR, Alfa Aesar* or *BLD pharm*, and used without purification. Pure solvents were available from *Thermo Fisher*, and degassed (3 times) under argon atmosphere, then store under standard Schlenk technique (anhydrous and under inert atmosphere).

Purification: Analytical thin layer chromatography was performed using *MACHEREY-NAGEL Gmbn & Co. KG* silica gel plates (Silica gel 60 UV_{254}). Visualization was by ultraviolet fluorescence (λ = 254 nm) and/or staining with potassium permanganate ($KMnO_4$). The products were isolated from the reaction mixture by column chromatography on silica gel 60, 0.063-0.2 mm, 70-230 mesh (Merck). Gradient flash chromatography was conducted eluting with PE/EA, PE refers to pentane and EA refers to ethyl acetate, they were listed as volume/volume ratios.

Data collection: GC analysis was performed on an Agilent HP-7890A instrument with FID detector and HP-5 capillary column (polydimethylsiloxane with 5% phenyl groups, 30 m, 0.32 mm i.d., 0.25 μm film thickness) using argon as carrier gas. Electron impact (EI) mass spectra were recorded on AMD 402 mass spectrometer (70 eV). The data are given as mass units per charge (m/z). High resolution mass spectra (HRMS) were recorded on Agilent 6210. NMR spectra were recorded on Bruker Avance 300 and Bruker ARX 400 spectrometers. Multiplets were assigned as s(singlet), d (doublet), t (triplet), q (quartet), dd (doublet of doublet), m (multiplet) and br. s (broad singlet). Chemical shifts (ppm) are given relative to solvent: references for $CDCl_3$ were 7.26 ppm (^{1}H NMR) and 77.00 ppm (^{13}C NMR). All measurements were carried out at room temperature unless otherwise stated.

NOTE: Because of the high toxicity of carbon monoxide, all the reactions should be performed in an autoclave. The laboratory should be well-equipped with a CO detector and alarm system.

2. Optimization of Reaction Conditions

Table S1. Optimization of acids

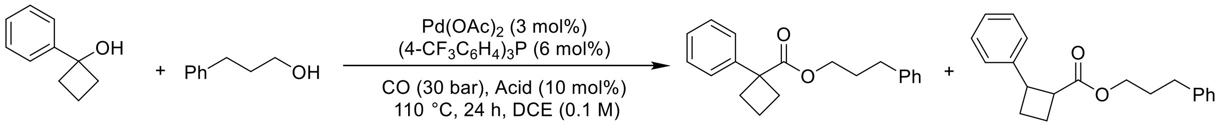

Entry	Acid	Yield (%)	*rr* (3a:4a)
1	HCl (4 M in1,4-dioxane)	11	13:1
2	CF_3COOH	ND	-
3	PhCOOH	ND	-
4	$PhB(OH)_3$	ND	-
5	5-Cl-SA	ND	-
6	HOAc	ND	-
7	NH_4Cl	ND	-
8	Without acid	ND	-

Reaction conditions: $Pd(OAc)_2$ (3 mol%), $(4\text{-}CF_3C_6H_4)_3P$ (6 mol%), acid (10 mol%), **1a** (0.12 mmol), **2a** (0.1 mmol), CO (30 bar), DCE (1 mL) at 110 °C for 24 h. The yields and regioisomeric ratios (*rr*) were determined by GC and GC-MS with n-hexadecane as internal standard.

Table S2. Optimization the amount of acid

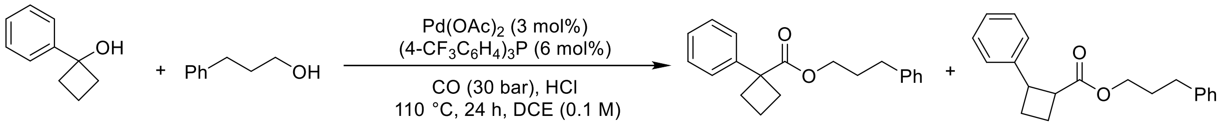

Entry	Acid	Yield (%)	*rr* (3a:4a)
1	10 mol% HCl	11	13:1
2	20 mol% HCl	21	4:1
3	30 mol% HCl	27	>20:1
4	50 mol% HCl	40	12:1
5	1 equiv. HCl	25	>20:1

Reaction conditions: $Pd(OAc)_2$ (3 mol%), $(4\text{-}CF_3C_6H_4)_3P$ (6 mol%), HCl (4 M in 1,4-dioxane), **1a** (0.12 mmol), **2a** (0.1 mmol), CO (30 bar), DCE (1 mL) at 110 °C for 24 h. The yields and regioisomeric ratios (*rr*) were determined by GC and GC-MS with n-hexadecane as internal standard.

Table S3. Optimization of catalysts

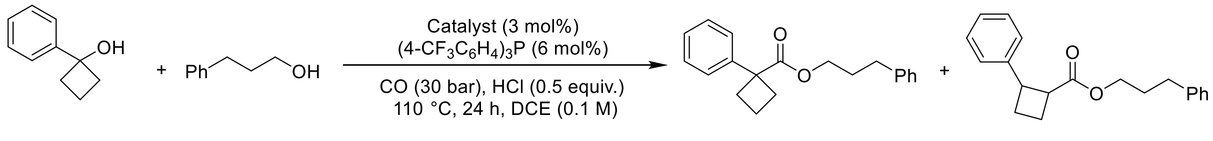

Entry	Catalysts	Yield (%)	*rr* (3a:4a)
1	$Pd(P^tBu_3)_2$	35	5:1
2	$Pd_2(dBa)_3$	24	>20:1
3	$Pd(TFA)_2$	28	>20:1
4	$Pd(CH_3CN)_2Cl_2$	46	>20:1
5	$Pd(OAc)_2$	40	12:1
6	$PdCl_2$	27	>20:1
7	$Pd(acac)_2$	40	>20:1
8[b]	$[Pd(\pi\text{-cinnamyl})Cl]_2$	44	>20:1

Reaction conditions: Catalyst (3 mol%), $(4\text{-}CF_3C_6H_4)_3P$ (6 mol%), 0.5 equiv. HCl (4 M in 1,4-dioxane), **1a** (0.12 mmol), **2a** (0.1 mmol), CO (30 bar), DCE (1 mL) at 110 °C for 24 h. The yields were determined by GC and GC-MS with n-hexadecane as internal standard. [b] $[Pd(\pi\text{-cinnamyl})Cl]_2$ (1.5 mol%).

Table S4. Optimization of temperature, pressure, and time.

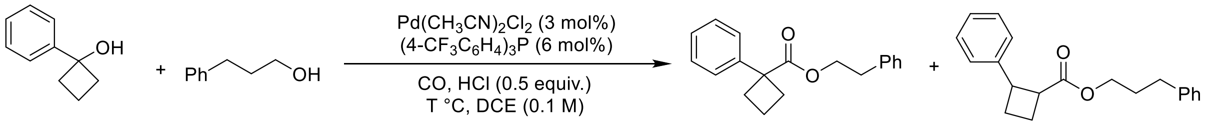

Entry	Temperature (°C)	Pressure (bar)	Time (h)	Yield (%)	*rr* (3a:4a)
1	120	30	24	76	14:1
2[b]	120	30	24	74	17:1
3	110	40	24	83(80)	19:1
4[b]	110	40	24	80	>20:1
5	120	40	24	52	13:1
6[b]	120	40	24	50	14:1
7	110	40	36	88	13:1
8[b]	110	40	36	81	>20:1

Reaction conditions: $Pd(CH_3CN)_2Cl_2$ (3 mol%), $(4\text{-}CF_3C_6H_4)_3P$ (6 mol%), 0.5 equiv. HCl (4 M in 1,4-dioxane), **1a** (0.12 mmol) ,**2a** (0.1 mmol), CO, DCE (1 mL). The yields were determined by GC with n-hexadecane as internal standard. Isolated yields in parentheses. [b] $[Pd(\pi\text{-cinnamyl})Cl]_2$ (1.5 mol%) as catalyst.

Table S5. Optimization of amount of **1a** and concentration.

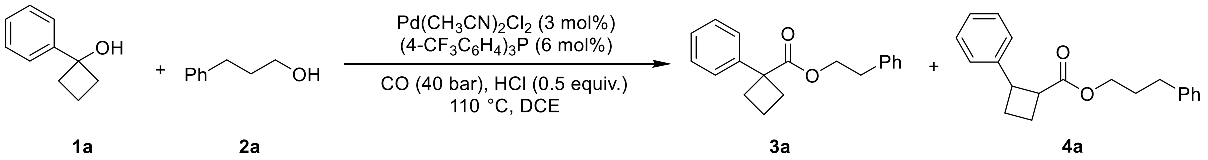

Entry	1a	DCE	Yield (%)	*rr* (3a:4a)
1	1.2 equiv.	1.0 mL	83(80)	13:1
2	1.5 equiv.	1.0 mL	76	17:1
3	1.2 equiv.	2.0 mL	50	19:1
4	1.2 equiv.	0.5 mL	85	>20:1
5[b]	1.2 equiv.	0.7 mL	94(89)	16:1
6[b, c]	1.2 equiv.	0.7 mL	96(94)	18:1

Reaction conditions: $Pd(CH_3CN)_2Cl_2$ (3 mol%), $(4\text{-}CF_3C_6H_4)_3P$ (6 mol%), 0.5 equiv. HCl (4 M in 1,4-dioxane), **1a** (0.12 mmol) ,**2a** (0.1 mmol), CO (40 bar), DCE (1 mL) for 24h. The yields were determined by GC with n-hexadecane as internal standard. Isolated yields in parentheses. [b] $Pd(CH_3CN)_2Cl_2$ (3 mol%), $(4\text{-}CF_3C_6H_4)_3P$ (6 mol%), 0.5 equiv. HCl (4 M in 1,4-dioxane), **1a** (0.24 mmol), **2a** (0.2 mmol), CO (40 bar), DCE (0.7 mL) at 110 °C for 36 h. [c] [Pd(π-cinnamyl)Cl]2 (1.5 mol%) as catalyst.

Table S6. Optimization of solvents.

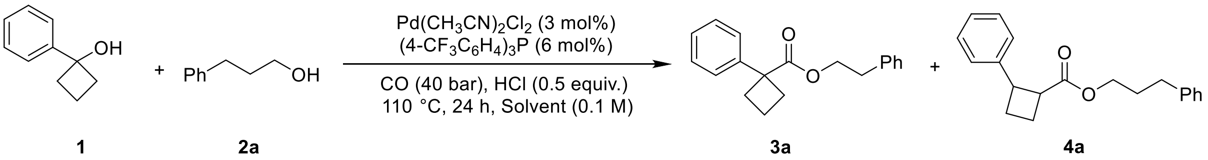

Entry	Variation of standard conditions	Yield of 3a [a] (%)	*rr (3a:4a)*
1	THF	trace	-
2	CH_3CN	9	1:1
3	Dioxane	trace	-
4	Toluene	trace	-
5	DMSO	ND	-
6	DMAc	ND	-
7	DCM	34	10:1
8	PhF	11	12:1
9	PhCl	15	10:1
10	DCE	83(80)	19:1

[a] Reaction conditions: $Pd(CH_3CN)_2Cl_2$ (3 mol%), $(4\text{-}CF_3C_6H_4)_3P$ (6 mol%), 0.5 equiv. HCl (4.0 M in dioxane), **1a** (0.12 mmol), **2a** (0.1 mmol), CO (40 bar), DCE (0.1 mL) at 110 °C for 24 h. [b] The yields and regioisomeric ratios (*rr*) were determined by GC and GC-MS with n-hexadecane as internal standard. Isolated yield in parentheses.

Table S7. Optimization of ligands.

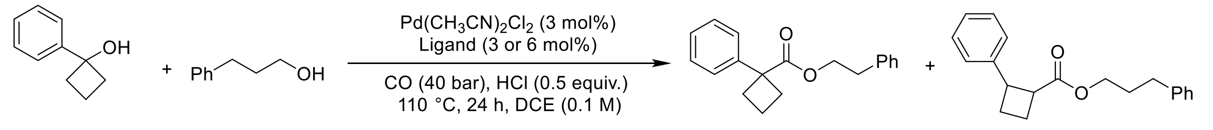

1 **2a** **3a** 4a

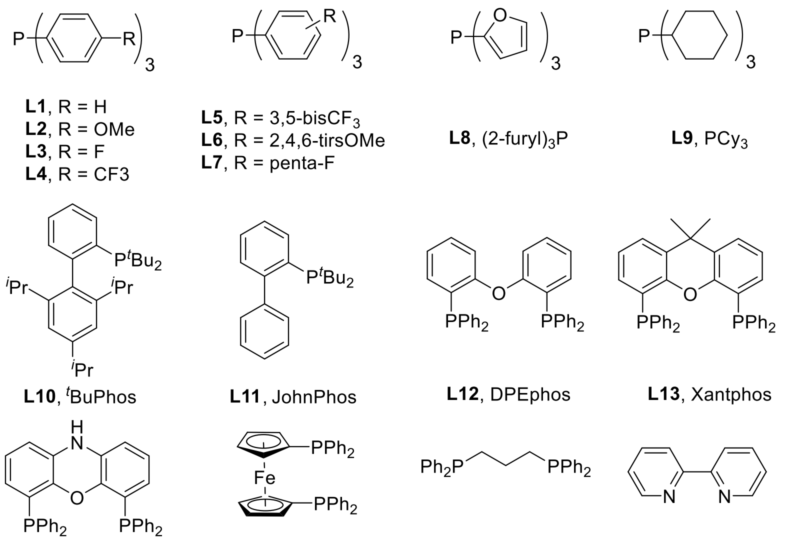

L1, R = H; **L2**, R = OMe; **L3**, R = F; **L4**, R = CF3; **L5**, R = 3,5-bisCF$_3$; **L6**, R = 2,4,6-tirsOMe; **L7**, R = penta-F; **L8**, (2-furyl)$_3$P; **L9**, PCy$_3$; **L10**, ^{t}BuPhos; **L11**, JohnPhos; **L12**, DPEphos; **L13**, Xantphos; **L14**, NiXantphos; **L15**, dppf; **L16**, dppp; **L17**, bpy

Entry	Ligand	Yield of 3a [a] (%)	*rr* (3a:4a)
1	**L1**	51	10:1
2	**L2**	53	12:1
3	**L3**	54	18:1
4	**L4**	83(80)[c]	19:1
5	**L5**	11	>20:1
6	**L6, L7**	ND	-
7	**L8**	4	8:1
8	**L9**	2	>20:1
9	**L10**	12	>20:1
10	**L11**	16	>20:1
11	**L12**	ND	-
12	**L13**	ND (63)[d]	>1:20
13	**L14**	5 (61)[d]	1:12
14	**L15-17**	ND	-

[a] unless otherwise noted, the reactions were performed with Pd(CH$_3$CN)$_2$Cl$_2$ (3 mol%), bisphophine ligand (3 mol%) or monophophine ligand (6 mol%), 0.5 equiv. HCl (4.0 M in dioxane), **1a** (0.12 mmol), **2a** (0.1 mmol), CO (40 bar), DCE (0.1 mL) at 110 °C for 24 h. [b] The yields and regioisomeric ratios (*rr*) were determined by GC and GC-MS with n-hexadecane as internal standard. [c] Isolated yield of **3a**. [d] The GC yield of **4a**.

3. General procedure for the preparation of cyclobutanols

3.1 List of cycobutanols

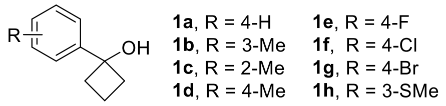

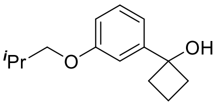

1i

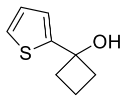

1j

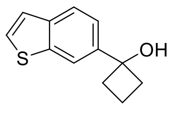

1k

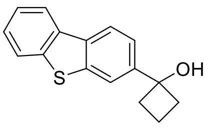

1l

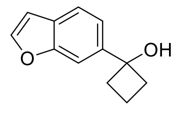

1m

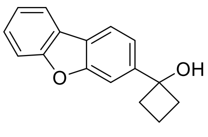

1n

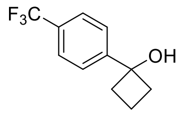

1o

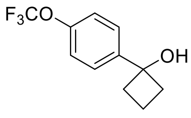

1p

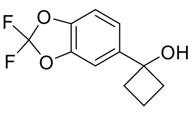

1q

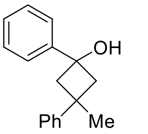

1r

All cyclobutanols **1a**-**1s** were prepared according to reported literature procedures and their characterization data match the reported data[1].

3.2 Typical procedure A

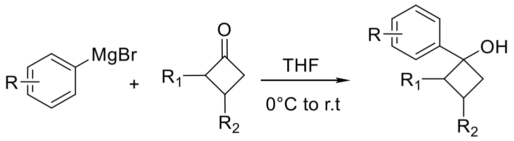

1a-**1f**, **1h**-**1s** were synthesized according to the reported procedure[1a]. Corresponding cyclobutanone (30 mmol) was dissolved in 30 mL of dried THF and purged with N_2. The mixture was cooled to 0 °C. An appropriate Grignard reagent (1.5 equiv.) was added dropwise with stirring under the same temperature. Then it was allowed to warm to room temperature and overnight. After that, the mixture was quenched with NH_4Cl (aq.). The aqueous layer was extracted with ethyl acetate (3×40 mL). The combined organic layers were dried over anhydrous Na_2SO_4, filtered, and concentrated under reduced pressure. After removal of the solvent in vacuo, the crude material was purified by flash column chromatography on silica gel to give the product **1a**-**1f**, **1h**-**1s**.

3.3 Typical procedure B

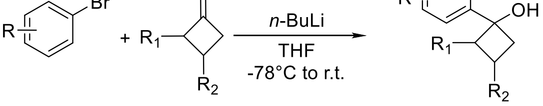

1j were synthesized according to the reported procedure[1b]. To the solution of ArBr (1.2 eq.) in dry THF (0.25 M) was added dropwise *n*-BuLi solution in hexane (1.64 M, 1.4 eq.) at -78 °C under argon. After stirring for 1 hour, cyclobutanone (1.0 eq.) was added dropwise. The reaction mixture was stirred for 1 hour and warmed to room temperature. After the further stirring for the adequate time, water was added dropwise and extracted with ethyl

acetate (3×40 mL). The combined organics were dried over Na_2SO_4, filtered, and concentrated under reduced pressure. The residue was purified by silica-gel column chromatography to give the product **1j**.

4.General procedure for the synthesis of Cyclobutanecarboxylates

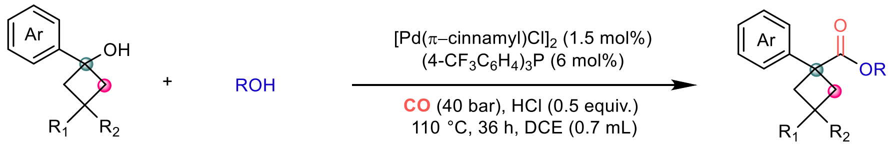

1 **2** **3**

A vial (4 mL) was charged with [Pd(π-cinnamyl)Cl]$_2$ (1.5 mol%, 1.5 mg), (4-$CF_3C_6H_4$)$_3$P (6 mol%, 5.6 mg), cyclobutanol (1.2 equiv., 0.24 mmol,), and a stirring bar. The vial was closed by PTFE/white rubber septum (Wheaton 13 mm Septa) and phenolic cap and connected with atmosphere with a needle. The vial was evacuated under vacuum and recharged with argon for three times. After that, DCE (0.7 mL) were injected under argon by using a syringe. Then the alcohol (0.2 mmol, 1.0 equiv.) and 0.5 equiv. HCl (4 M in 1,4-dioxane, 25 μL), were added with a syringe under nitrogen atmosphere. Subsequently, the vial (or several vials) was placed in an alloy plate, which was transferred into a 300 mL autoclave of the 4560 series from Parr Instruments. After flushing the autoclave three times with CO, a pressure of 40 bar of CO was adjusted at ambient temperature. Then, the reaction was performed for 36 h at 110 °C (aluminum block). After 36 hours, the autoclave was cooled down with ice water to room temperature and the pressure was released carefully. The solution was concentrated in vacuo then purified by silica-gel column chromatography using pentane and ethyl acetate to afford the corresponding product **3**.

2 mmol scale: A vial (12 mL) was charged with [Pd(π-cinnamyl)Cl]$_2$ (1.5 mol%), (4-$CF_3C_6H_4$)$_3$P (6 mol%), cyclobutanol **1a** (1.2 equiv., 2.4 mmol,), and a stirring bar. The vial was closed by PTFE/white rubber septum and phenolic cap and connected with atmosphere with a needle. The vial was evacuated under vacuum and recharged with argon for three times. After that, DCE (7 mL) were injected under argon by using a syringe. Then the alcohol **2a** (2 mmol, 1.0 equiv.) and 0.5 equiv. HCl (4 M in 1,4-dioxane, 125 μL), were added with a syringe under nitrogen atmosphere. Subsequently, the vial (or several vials) was placed in an alloy plate, which was transferred into a 300 mL autoclave of the 4560 series from Parr Instruments. After flushing the autoclave three times with CO, a pressure of 40 bar of CO was adjusted at ambient temperature. Then, the reaction was performed for 36 h at 110 °C (aluminum block). After 36 hours, the autoclave was cooled down with ice water to room temperature and the pressure was released carefully. The solution was concentrated in vacuo then purified by silica-gel column chromatography using pentane and ethyl acetate to afford the corresponding product **3a** in 74% yield (0.435 g).

5. Spectroscopic Data of Products

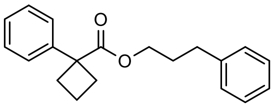

3-Phenylpropyl 1-phenylcyclobutane-1-carboxylate (3a)
55.4 mg, 94% yield, colorless oil. Eluent: pentane/ethyl acetate = 100/1, Rf = 0.3.
^{1}H NMR (300 MHz, CDCl$_3$) δ 7.30 – 7.21 (m, 4H), 7.19 – 7.13 (m, 3H), 7.11 – 7.05 (m, 1H), 6.96 – 6.92 (m, 2H), 3.97 (t, *J* = 6.3 Hz, 2H), 2.83 – 2.72 (m, 2H), 2.51 – 2.37 (m, 4H), 2.06 – 1.89 (m, 1H), 1.89 – 1.71 (m, 3H).
^{13}C NMR (75 MHz, CDCl$_3$) δ 176.0, 144.0, 141.3, 128.5, 128.5, 128.4, 126.7, 126.4, 126.0, 64.1, 52.6, 32.4, 32.0, 30.4, 16.8.
HRMS (ESI-TOF): m/z calcd. for $[M + Na]^+$ $C_{20}H_{22}NaO_2^+$ 317.1512; Found: 317.1515.

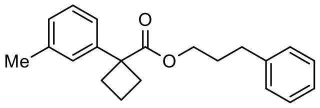

3-Phenylpropyl 1-(*m*-tolyl)cyclobutane-1-carboxylate (3b)
40.0 mg, 65%, colorless oil. Eluent: pentane/ethyl acetate = 100/1, Rf = 0.3.
^{1}H NMR (400 MHz, CDCl$_3$) δ 7.18 – 7.13 (m, 3H), 7.11 – 7.04 (m, 3H), 7.00 – 6.89 (m, 3H), 3.96 (t, *J* = 6.3 Hz, 2H), 2.81 – 2.70 (m, 2H), 2.50 – 2.38 (m, 4H), 2.27 (s, 3H), 2.04 – 1.88 (m, 1H), 1.86 – 1.72 (m, 3H).
^{13}C NMR (101 MHz, CDCl$_3$) δ 176.1, 143.9, 141.3, 137.9, 128.5, 128.5, 128.3, 127.5, 127.1, 126.0, 123.4, 64.0, 52.57, 32.4, 32.0, 30.4, 21.6, 16.8.
HRMS (ESI-TOF): m/z calcd. for $[M + Na]^+$ $C_{21}H_{24}NaO_2^+$ 331.1669; Found: 331.1673.

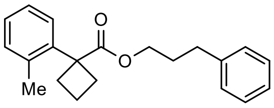

3-Phenylpropyl 1-(*o*-tolyl)cyclobutane-1-carboxylate (3c)
30.6 mg, 50%, colorless oil, Eluent: pentane/ethyl acetate = 100/1, Rf = 0.3.
^{1}H NMR (400 MHz, CDCl$_3$) δ 7.20 – 7.13 (m, 4H), 7.10 – 7.02 (m, 3H), 6.92 – 6.88 (m, 2H), 3.97 (t, *J* = 6.3 Hz, 3H), 2.82 – 2.71 (m, 2H), 2.57 – 2.44 (m, 2H), 2.40 – 2.30 (m, 2H), 2.14 (s, 3H), 1.85 – 1.70 (m, 3H).
^{13}C NMR (101 MHz, CDCl$_3$) δ 175.9, 142.1, 141.2, 136.2, 131.0, 128.6, 128.5, 127.2, 126.9, 126.0, 125.8, 64.0, 53.35, 32.7, 31.9, 30.4, 20.0, 17.2.
HRMS (ESI-TOF): m/z calcd. for $[M + Na]^+$ $C_{21}H_{24}NaO_2^+$ 331.1669; Found: 331.1673.

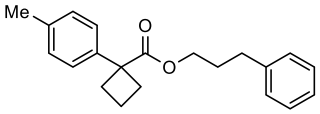

3-Phenylpropyl 1-(*p*-tolyl)cyclobutane-1-carboxylate (3d)
55.5 mg, 90%, colorless oil, Eluent: pentane/ethyl acetate = 100/1, Rf = 0.3.
^{1}H NMR (300 MHz, CDCl$_3$) δ 7.18 – 7.12 (m, 4H), 7.11 – 7.04 (m, 3H), 6.95 – 6.90 (m, 1H), 3.96 (t, *J* = 6.3 Hz, 2H), 2.83 – 2.68 (m, 2H), 2.50 – 2.34 (m, 2H), 2.25 (s, 3H), 2.03 – 1.89 (m, 1H), 1.88 – 1.69 (m, 3H).
^{13}C NMR (75 MHz, CDCl$_3$) δ 176.1, 141.3, 141.0, 136.3, 129.0, 128.5, 128.4, 126.3, 126.0, 64.0, 52.3, 32.4, 32.0, 30.4, 21.1, 16.8.
HRMS (ESI-TOF): m/z calcd. for $[M + Na]^+$ $C_{21}H_{24}NaO_2^+$ 331.1669; Found: 331.1673.

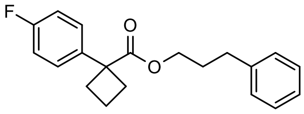

3-Phenylpropyl 1-(4-fluorophenyl)cyclobutane-1-carboxylate (3e)
49.8 mg, 80%, colorless oil, Eluent: pentane/ethyl acetate = 80/1, Rf = 0.3.
^{1}H NMR (300 MHz, CDCl$_3$) δ 7.24 – 7.14 (m, 4H), 7.12 – 7.06 (m, 1H), 6.98 – 6.90 (m, 4H), 3.97 (t, *J* = 6.4 Hz, 2H), 2.83 – 2.71 (m, 2H), 2.48 – 2.35 (m, 4H), 2.06 – 1.90 (m, 1H), 1.87 – 1.73 (m, 3H).
^{13}C NMR (75 MHz, CDCl$_3$) δ 175.8, 161.7 (d, *J* = 245.3 Hz), 141.2, 139.7 (d, *J* = 3.1 Hz), 128.5, 128.5, 128.1 (d, *J* = 8.0 Hz), 126.1, 115.2 (d, *J* = 21.3 Hz), 64.2, 52.0, 32.5, 32.0, 30.3, 16.7.

^{19}F NMR (282 MHz, $CDCl_3$) δ -113.66 – -118.60 (m).
HRMS (ESI-TOF): m/z calcd. for $[M + Na]^+$ $C_{20}H_{21}FNaO_2^+$ 335.1418; Found: 335.1419.

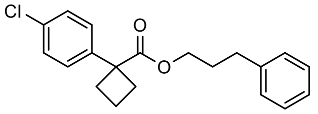

3-Phenylpropyl 1-(4-chlorophenyl)cyclobutane-1-carboxylate (3f)
47.2 mg, 72%, colorless oil, Eluent: pentane/ethyl acetate = 80/1, Rf = 0.3.
^{1}H NMR (300 MHz, $CDCl_3$) δ 7.26 – 7.14 (m, 6H), 7.12 – 7.06 (m, 1H), 7.00 – 6.91 (m, 2H), 3.97 (t, *J* = 6.4 Hz, 2H), 2.83 – 2.68 (m, 2H), 2.49 – 2.33 (m, 4H), 2.08 – 1.89 (m, 1H), 1.86 – 1.71 (m, 3H).
^{13}C NMR (75 MHz, $CDCl_3$) δ 175.5, 142.5, 141.1, 132.6, 128.5, 128.5, 128.5, 127.9, 126.1, 64.3, 52.2, 32.4, 32.0, 30.3, 16.8.
HRMS (ESI-TOF): m/z calcd. for $[M + Na]^+$ $C_{20}H_{21}ClNaO_2^+$ 351.1122; Found: 351.1125.

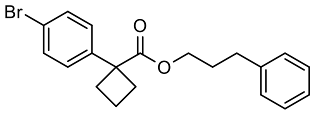

3-Phenylpropyl 1-(4-bromophenyl)cyclobutane-1-carboxylate (3g)
57.6 mg, 77%, colorless oil, Eluent: pentane/ethyl acetate = 80/1, Rf = 0.3.
^{1}H NMR (300 MHz, $CDCl_3$) δ 7.41 – 7.35 (m, 2H), 7.21 – 7.14 (m, 2H), 7.14 – 7.05 (m, 3H), 6.99 – 6.92 (m, 2H), 3.97 (t, *J* = 6.3 Hz, 2H), 2.81 – 2.70 (m, 2H), 2.46 – 2.34 (m, 4H), 2.06 – 1.89 (m, 1H), 1.87 – 1.72 (m, 3H).
^{13}C NMR (75 MHz, $CDCl_3$) δ 175.4, 143.0, 141.1, 131.5, 128.5, 128.4, 128.3, 126.1, 120.7, 64.3, 52.2, 32.3, 32.1, 30.3, 16.8.
HRMS (ESI-TOF): m/z calcd. for $[M + Na]^+$ $C_{20}H_{21}BrNaO_2^+$ 395.0617; Found: 395.0625.

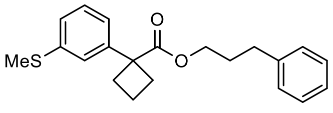

3-Phenylpropyl 1-(3-(methylthio)phenyl)cyclobutane-1-carboxylate (3h)
55.8 mg, 82%, colorless oil, Eluent: pentane/ethyl acetate = 80/1, Rf = 0.4.
^{1}H NMR (400 MHz, $CDCl_3$) δ 7.21 – 7.13 (m, 4H), 7.11 – 7.00 (m, 3H), 6.97 – 6.92 (m, 2H), 3.96 (t, *J* = 12.6 Hz, 2H), 2.81 – 2.71 (m, 2H), 2.49 – 2.40 (m, 4H), 2.39 (s, 3H), 2.05 – 1.89 (m, 1H), 1.87 – 1.72 (m, 3H).
^{13}C NMR (101 MHz, $CDCl_3$) δ 175.7, 144.7, 141.2, 138.6, 128.8, 128.5, 128.4, 126.0, 124.8, 124.7, 123.3, 64.1, 52.56, 32.3, 32.0, 30.3, 16.8, 16.0.
HRMS (ESI-TOF): m/z calcd. for $[M + H]^+$ $C_{21}H_{25}O_2S^+$ 341.1570; Found: 341.1577.

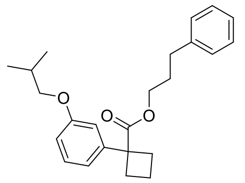

3-Phenylpropyl 1-(3-isobutoxyphenyl)cyclobutane-1-carboxylate (3i)
56.0 mg 76%, colorless oil, Eluent: pentane/ethyl acetate = 80/1, Rf = 0.4.
^{1}H NMR (400 MHz, $CDCl_3$) δ 7.18 – 7.12 (m, 3H), 7.10 – 7.05 (m, 1H), 6.96 – 6.91 (m, 2H), 6.85 – 6.76 (m, 2H), 6.69 (ddd, *J* = 8.2, 2.5, 0.9 Hz, 1H), 3.97 (t, *J* = 6.3 Hz, 2H), 3.62 (d, *J* = 6.5 Hz, 2H), 2.80 – 2.69 (m, 2H), 2.50 – 2.38 (m, 4H), 2.06 – 1.88 (m, 2H), 1.87 – 1.72 (m, 3H), 0.93 (d, *J* = 6.7 Hz, 6H).
^{13}C NMR (101 MHz, $CDCl_3$) δ 175.9, 159.4, 145.5, 141.3, 129.3, 128.5, 128.4, 126.0, 118.6, 113.0, 112.5, 74.5, 64.02, 52.7, 32.4, 32.0, 30.4, 28.5, 19.4, 16.8.
HRMS (ESI-TOF): m/z calcd. for $[M + H]^+$ $C_{24}H_{31}O_3^+$ 367.2268; Found: 367.2273.

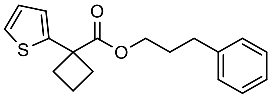

3-Phenylpropyl 1-(thiophen-2-yl)cyclobutane-1-carboxylate (3j)
19.6 mg, 33%, colorless oil, Eluent: pentane/ethyl acetate = 80/1, Rf = 0.3.
^{1}H NMR (300 MHz, CDCl$_3$) δ 7.23 – 7.09 (m, 4H), 7.05 – 6.99 (m, 2H), 6.92 – 6.87 (m, 2H), 4.04 (t, *J* = 6.4 Hz, 2H), 2.84 – 2.73 (m, 2H), 2.56 – 2.38 (m, 4H), 2.01 – 1.80 (m, 4H).
^{13}C NMR (75 MHz, CDCl$_3$) δ 174.9, 147.5, 141.3, 128.6, 128.5, 126.8, 126.1, 124.5, 124.4, 64.4, 49.6, 34.0, 32.1, 30.4, 16.7.
HRMS (ESI-TOF): m/z calcd. for $[M + Na]^+$ $C_{18}H_{20}O_2SNa^+$ 323.1076; Found: 323.1083.

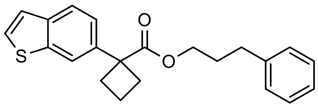

3-Phenylpropyl 1-(benzo[*b*]thiophen-6-yl)cyclobutane-1-carboxylate (3k)
50.6 mg, 72%, colorless oil, Eluent: pentane/ethyl acetate = 80/1, Rf = 0.3.
^{1}H NMR (300 MHz, CDCl$_3$) δ 7.75 (dt, *J* = 8.4, 0.8 Hz, 1H), 7.71 – 7.69 (d, *J* = 1.8 Hz, 1H), 7.35 (dd, *J* = 5.4, 0.5 Hz, 1H), 7.25 – 7.22 (m, 2H), 7.14 – 7.01 (m, 3H), 6.88 – 6.83 (m, 2H), 3.96 (t, *J* = 6.3 Hz, 2H), 2.89 – 2.78 (m, 2H), 2.57 – 2.45 (m, 2H), 2.39 (dd, *J* = 8.7, 6.7 Hz, 2H), 2.09 – 1.92 (m, 1H), 1.90 – 1.69 (m, 3H).
^{13}C NMR (75 MHz, CDCl$_3$) δ 176.1, 141.2, 140.3, 139.8, 138.2, 128.5, 127.0, 126.0, 123.9, 123.1, 122.4, 121.3, 64.1, 52.6, 32.6, 32.0, 30.4, 16.9.
HRMS (ESI-TOF): m/z calcd. for $[M + Na]^+$ $C_{22}H_{22}O_2SNa^+$ 373.1233; Found: 373.1238.

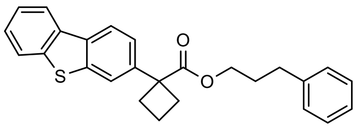

3-Phenylpropyl 1-(dibenzo[b,d]thiophen-3-yl)cyclobutane-1-carboxylate (3l)
57.2 mg, 72%, colorless oil, Eluent: pentane/ethyl acetate = 80/1, Rf = 0.3.
^{1}H NMR (300 MHz, CDCl$_3$) δ 8.13 – 8.06 (m, 1H), 8.02 (d, *J* = 1.2 Hz, 1H), 7.81 – 7.68 (m, 2H), 7.41 – 7.30 (m, 3H), 7.11 – 6.98 (m, 3H), 6.89 – 6.79 (m, 2H), 3.97 (t, *J* = 6.3 Hz, 2H), 2.93 – 2.83 (m, 2H), 2.61 – 2.51 (m, 2H), 2.42 – 2.36 (m, 2H), 2.12 – 1.95 (m, 1H), 1.92 – 1.70 (m, 3H).
^{13}C NMR (75 MHz, CDCl$_3$) δ 176.0, 141.1, 140.5, 140.0, 137.9, 135.7, 135.5, 128.4, 126.9, 126.0, 125.4, 124.5, 123.0, 122.7, 121.8, 119.3, 64.2, 52.7, 32.6, 32.0, 30.3, 16.9.
HRMS (ESI-TOF): m/z calcd. for $[M + Na]^+$ $C_{26}H_{24}O_2SNa^+$ 423.1389; Found: 423.1396.

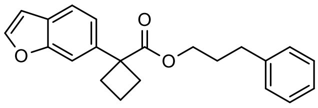

3-Phenylpropyl 1-(benzofuran-6-yl)cyclobutane-1-carboxylate (3m)
50.0 mg, 75%, colorless oil, Eluent: pentane/ethyl acetate = 80/1, Rf = 0.3.
^{1}H NMR (300 MHz, CDCl$_3$) δ 7.55 – 7.46 (m, 1H), 7.40 – 7.35 (m, 1H), 7.21 – 7.01 (m, 4H), 6.91 – 6.86 (m, 2H), 6.66 (dd, *J* = 2.2, 1.0 Hz, 1H), 3.96 (t, *J* = 6.3 Hz, 2H), 2.87 – 2.77 (m, 2H), 2.49 (tdd, *J* = 9.4, 8.1, 2.8 Hz, 2H), 2.43 – 2.37 (m, 2H), 2.07 – 1.91 (m, 1H), 1.88 – 1.71 (m, 3H).
^{13}C NMR (75 MHz, CDCl$_3$) δ 176.3, 153.9, 145.5, 141.2, 138.7, 128.5, 127.5, 126.0, 123.0, 118.9, 111.2, 106.7, 64.1, 52.6, 32.7, 32.0, 30.4, 16.8.
HRMS (ESI-TOF): m/z calcd. for $[M + Na]^+$ $C_{22}H_{22}O_3Na^+$ 357.1461; Found: 357.1466.

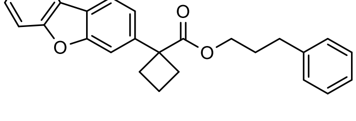

3-Phenylpropyl 1-(dibenzo[b,d]furan-3-yl)cyclobutane-1-carboxylate (3n)
58.3 mg, 76%, colorless oil, Eluent: pentane/ethyl acetate = 80/1, Rf = 0.3.

^{1}H NMR (300 MHz, CDCl$_3$) δ 7.89 – 7.80 (m, 2H), 7.49 – 7.41 (m, 2H), 7.38 – 7.30 (m, 2H), 7.24 (td, *J* = 7.5, 1.1 Hz, 1H), 7.11 – 6.98 (m, 3H), 6.90 – 6.84 (m, 2H), 3.97 (t, *J* = 6.3 Hz, 2H), 2.93 – 2.81 (m, 1H), 2.61 – 2.48 (m, 2H), 2.44 – 2.35 (m, 2H), 2.11 – 1.95 (m, 1H), 1.92 – 1.70 (m, 3H).
^{13}C NMR (75 MHz, CDCl$_3$) δ 176.2, 156.7, 155.1, 141.1, 138.7, 128.4, 127.3, 126.0, 125.7, 124.3, 124.2, 122.8, 120.81, 118.5, 111.8, 111.4, 64.2, 52.6, 32.7, 32.0, 30.3, 16.9.
HRMS (ESI-TOF): m/z calcd. for $[M + Na]^+$ $C_{26}H_{24}O_3Na^+$ 407.1617; Found: 407.1626.

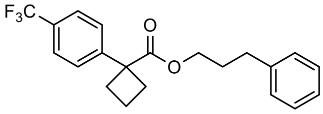

3-Phenylpropyl 1-(4-(trifluoromethyl)phenyl)cyclobutane-1-carboxylate (3o)
25.8 mg, 35%, colorless oil, Eluent: pentane/ethyl acetate = 80/1, Rf = 0.3.
^{1}H NMR (300 MHz, CDCl$_3$) δ 7.56 – 7.50 (m, 2H), 7.38 – 7.33 (m, 2H), 7.20 – 7.14 (m, 2H), 7.12 – 7.06 (m, 1H), 7.03 – 6.88 (m, 2H), 3.99 (t, *J* = 6.4 Hz, 2H), 2.88 – 2.73 (m, 2H), 2.51 – 2.40 (m, 4H), 2.11 – 1.95 (m, 1H), 1.90 – 1.72 (m, 3H).
^{13}C NMR (75 MHz, CDCl$_3$) δ 175.2, 148.0, 141.1, 128.6, 128.5, 126.9, 126.2, 125.3 (q, *J* = 3.8 Hz), 64.5, 52.6, 32.5, 32.1, 30.3, 16.9.
^{19}F NMR (282 MHz, CDCl$_3$) δ -62.43 (s).
HRMS (ESI-TOF): m/z calcd. for $[M + Na]^+$ $C_{21}H_{21}F_3O_2Na^+$ 385.1386; Found: 385.1388.

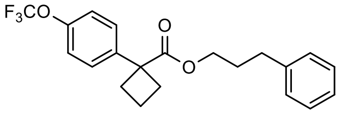

3-Phenylpropyl 1-(4-(trifluoromethoxy)phenyl)cyclobutane-1-carboxylate (3p)
28.5 mg, 37%, colorless oil, Eluent: pentane/ethyl acetate = 80/1, Rf = 0.3.
^{1}H NMR (300 MHz, CDCl$_3$) δ 7.32 – 7.24 (m, 1H), 7.20 – 7.14 (m, 3H), 7.12 – 7.06 (m, 2H), 7.05 – 7.00 (m, 1H), 6.97 – 6.93 (m, 2H), 3.99 (d, *J* = 6.3 Hz, 2H), 2.84 – 2.73 (m, 2H), 2.49 – 2.37 (m, 4H), 2.10 – 1.93 (m, 1H), 1.89 – 1.73 (m, 3H).
^{13}C NMR (75 MHz, CDCl$_3$) δ 175.2, 149.4, 146.4, 141.1, 129.7, 128.5, 128.5, 126.1, 124.9, 119.3 (q, *J* = 15.5 Hz), 64.4, 52.4, 32.4, 32.0, 30.3, 16.8.
^{19}F NMR (282 MHz, CDCl$_3$) δ -57.72 (s).
HRMS (ESI-TOF): m/z calcd. for $[M + Na]^+$ $C_{21}H_{21}F_3O_3Na^+$ 401.1335; Found: 401.1339.

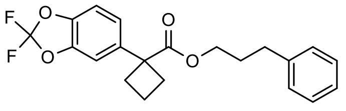

3-Phenylpropyl 1-(2,2-difluorobenzo[d][1,3]dioxol-5-yl)cyclobutane-1-carboxylate (3q)
39.6 mg, 53%, colorless oil, Eluent: pentane/ethyl acetate = 80/1, Rf = 0.3.
^{1}H NMR (300 MHz, CDCl$_3$) δ 7.21 – 7.14 (m, 2H), 7.15 – 7.04 (m, 1H), 7.02 – 6.89 (m, 5H), 3.99 (t, *J* = 6.4 Hz, 2H), 2.81 – 2.71 (m, 2H), 2.50 – 2.32 (m, 4H), 2.07 – 1.91 (m, 1H), 1.87 – 1.74 (m, 3H).
^{13}C NMR (75 MHz, CDCl$_3$) δ 175.4, 143.9, 142.5, 141.1, 140.4, 133.5 (t, *J* = 255.1 Hz), 128.6, 128.5, 126.2, 121.6, 109.1, 108.2, 64.5, 52.5, 32.6, 32.1, 30.3, 16.7.
^{19}F NMR (282 MHz, CDCl$_3$) δ -49.97 (s).
HRMS (ESI-TOF): m/z calcd. for $[M + Na]^+$ $C_{21}H_{20}F_2O_4Na^+$ 397.1222; Found: 397.1228.

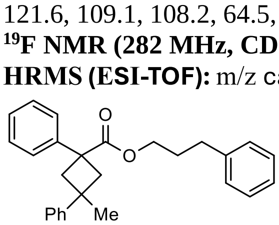

3-Phenylpropyl 3-methyl-1,3-diphenylcyclobutane-1-carboxylate (3r)
23.0mg, 30%, colorless oil, Eluent: pentane/ethyl acetate = 50/1, Rf = 0.4.
^{1}H NMR (300 MHz, CDCl$_3$) δ 7.45 – 7.35 (m, 2H), 7.34 – 7.24 (m, 3H), 7.23 – 7.16 (m, 4H), 7.16 – 7.04 (m, 4H), 6.95 – 6.82 (m, 2H), 3.77 (t, *J* = 6.3 Hz, 2H), 3.36 – 3.26 (m, 2H), 2.79 – 2.67 (m, 2H), 2.38 – 2.27 (m, 2H), 1.70 – 1.55 (m, 2H), 1.18 (s, 3H).
^{13}C NMR (75 MHz, CDCl$_3$) δ 175.7, 150.5, 143.7, 141.3, 128.5, 128.5, 128.4, 126.9, 126.8, 126.0, 125.7, 125.4, 64.1, 46.7, 44.5, 38.1, 32.5, 31.9, 30.2.
HRMS (ESI-TOF): m/z calcd. for $[M + Na]^+$ $C_{27}H_{28}O_2Na^+$ 407.1982; Found: 407.1988.

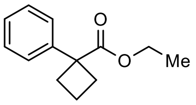

Ethyl 1-phenylcyclobutane-1-carboxylate (3s)
23.7 mg, 58%, colorless oil, Eluent: pentane/ethyl acetate = 200/1, Rf = 0.2.
^{1}H NMR (300 MHz, $CDCl_3$) δ 7.37 – 7.29 (m, 4H), 7.26 – 7.20 (m, 1H), 4.10 (q, *J* = 7.1 Hz, 2H), 2.89 – 2.80 (m, 2H), 2.56 – 2.43 (m, 2H), 2.12 – 1.96 (m, 1H), 1.94 – 1.80 (m, 1H), 1.18 (t, *J* = 7.1 Hz, 3H).
^{13}C NMR (75 MHz, $CDCl_3$) δ 176.1, 143.9, 128.3, 126.6, 126.4, 61.0, 52.6, 32.5, 16.8, 14.2.
HRMS (ESI-TOF): m/z calcd. for $[M + Na]^+$ $C_{13}H_{16}O_2Na^+$ 227.1043; Found: 227.1051.

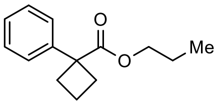

Propyl 1-phenylcyclobutane-1-carboxylate (3t)
27.1mg, 62%, colorless oil, Eluent: pentane/ethyl acetate = 200/1, Rf = 0.2.
^{1}H NMR (300 MHz, $CDCl_3$) δ 7.34 – 7.29 (m, 4H), 7.26 – 7.19 (m, 1H), 4.01 (t, *J* = 6.5 Hz, 2H), 2.93 – 2.77 (m, 2H), 2.60 – 2.43 (m, 2H), 2.10 – 1.97 (m, 1H), 1.94 – 1.80 (m, 1H), 1.62 – 1.52 (m, 2H), 0.82 (t, *J* = 7.4 Hz, 3H).
^{13}C NMR (75 MHz, $CDCl_3$) δ 176.1, 144.0, 128.3, 126.6, 126.4, 66.5, 52.6, 32.5, 22.1, 16.8, 10.4.
HRMS (ESI-TOF): m/z calcd. for $[M + Na]^+$ $C_{14}H_{18}O_2Na^+$ 241.1199; Found: 241.1205.

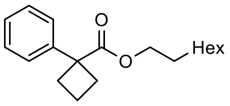

Octyl 1-phenylcyclobutane-1-carboxylate (3u)
40.4mg, 70%, colorless oil, Eluent: pentane/ethyl acetate = 200/1, Rf = 0.2.
^{1}H NMR (400 MHz, $CDCl_3$) δ 7.33 – 7.29 (m, 4H), 7.25 – 7.20 (m, 1H), 4.04 (t, *J* = 6.6 Hz, 2H), 2.90 – 2.80 (m, 2H), 2.58 – 2.46 (m, 2H), 2.10 – 1.99 (m, 1H), 1.94 – 1.80 (m, 1H), 1.53 (t, *J* = 6.8 Hz, 2H), 1.33 – 1.19 (m, 10H), 0.88 (t, *J* = 7.0 Hz, 3H).
^{13}C NMR (101 MHz, $CDCl_3$) δ 176.1, 144.0, 128.3, 126.6, 126.4, 65.1, 52.6, 32.4, 31.9, 29.3, 29.2, 28.6 25.8, 22.8, 16.8, 14.2.
HRMS (ESI-TOF): m/z calcd. for $[M + Na]^+$ $C_{19}H_{28}O_2Na^+$ 311.1982; Found: 311.1988.

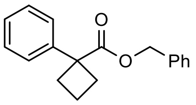

Benzyl 1-phenylcyclobutane-1-carboxylate (3v)
37.7 mg, 71%, colorless oil, Eluent: pentane/ethyl acetate = 80/1, Rf = 0.3.
^{1}H NMR (300 MHz, $CDCl_3$) δ 7.26 – 7.23 (m, 4H), 7.21 – 7.12 (m, 4H), 7.10 – 7.06 (m, 2H), 5.00 (s, 2H), 2.86 – 2.70 (m, 2H), 2.54 – 2.37 (m, 2H), 2.04 – 1.88 (m, 1H), 1.86 – 1.72 (m, 1H).
^{13}C NMR (75 MHz, $CDCl_3$) δ 175.8, 143.7, 136.4, 128.5, 128.4, 128.0, 127.5, 126.7, 126.5, 66.5, 52.6, 32.4, 16.8.
HRMS (ESI-TOF): m/z calcd. for $[M + Na]^+$ $C_{18}H_{18}O_2Na^+$ 289.1199; Found: 289.1208.

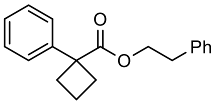

Phenethyl 1-phenylcyclobutane-1-carboxylate (3w)
39.2 mg, 70%, colorless oil, Eluent: pentane/ethyl acetate = 80/1, Rf = 0.3.
^{1}H NMR (300 MHz, $CDCl_3$) δ 7.26 – 7.20 (m, 2H), 7.20 – 7.14 (m, 4H), 7.14 – 7.07 (m, 2H), 7.03 – 6.93 (m, 2H), 4.18 (t, *J* = 6.8 Hz, 2H), 2.79 – 2.65 (m, 4H), 2.45 – 2.35 (m, 2H), 1.99 – 1.83 (m, 1H), 1.82 – 1.69 (m, 1H).
^{13}C NMR (75 MHz, $CDCl_3$) δ 175.9, 143.8, 138.0, 129.0, 128.5, 128.3, 126.6, 126.5, 126.4, 65.5, 52.5, 35.1, 32.4, 16.8.
HRMS (ESI-TOF): m/z calcd. for $[M + Na]^+$ $C_{19}H_{20}O_2Na^+$ 303.1356; Found: 303.1359.

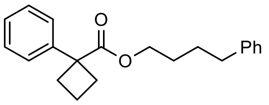

4-Phenylbutyl 1-phenylcyclobutane-1-carboxylate (3x)
37.1 mg, 60%, colorless oil, Eluent: pentane/ethyl acetate = 80/1, Rf = 0.3.
^{1}H NMR (300 MHz, CDCl$_3$) δ 7.25 – 7.18 (m, 5H), 7.18 – 7.07 (m, 3H), 7.05 – 6.99 (m, 2H), 3.99 (t, *J* = 6.1 Hz, 2H), 2.81 – 2.68 (m, 2H), 2.49 – 2.37 (m, 4H), 2.04 – 1.88 (m, 1H), 1.88 – 1.69 (m, 1H), 1.55 – 1.39 (m, 4H).
^{13}C NMR (75 MHz, CDCl$_3$) δ 176.1, 143.9, 142.2, 128.5, 128.4, 128.3, 126.6, 126.4, 125.9, 64.7, 52.6, 35.4, 32.4, 28.3, 27.6, 16.8.
HRMS (ESI-TOF): m/z calcd. for [M + Na]$^+$ $C_{21}H_{24}O_2Na^+$ 331.1669; Found: 331.1666.

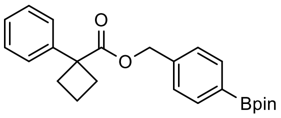

4-(4,4,5,5-Tetramethyl-1,3,2-dioxaborolan-2-yl)benzyl 1-phenylcyclobutane-1-carboxylate (3y)
37.8mg, 48%, colorless oil, Eluent: pentane/ethyl acetate = 20/1, Rf = 0.3.
^{1}H NMR (400 MHz, CDCl$_3$) δ 7.67 – 7.64 (m, 2H), 7.26 – 7.22 (m, 4H), 7.18 – 7.14 (m, 1H), 7.06 (d, *J* = 8.3 Hz, 2H), 5.02 (s, 2H), 2.82 – 2.73 (m, 2H), 2.52 – 2.40 (m, 2H), 2.04 – 1.88 (m, 1H), 1.86 – 1.72 (m, 1H), 1.26 (s, 12H).
^{13}C NMR (101 MHz, CDCl$_3$) δ 175.7, 143.7, 139.5, 135.0, 128.4, 126.8, 126.6, 126.5, 83.9, 66.4, 52.6, 32.4, 25.0, 16.8.
HRMS (ESI-TOF): m/z calcd. for [M + Na]$^+$ $C_{24}H_{29}BO_4Na^+$ 415.2051; Found: 415.2059.

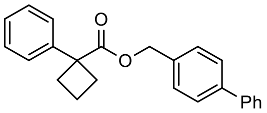

[1,1'-Biphenyl]-4-ylmethyl 1-phenylcyclobutane-1-carboxylate (3z)
38.7mg, 57%, colorless oil, Eluent: pentane/ethyl acetate = 80/1, Rf = 0.3.
^{1}H NMR (400 MHz, CDCl$_3$) δ 7.50 – 7.47 (m, 2H), 7.44 – 7.41 (m, 2H), 7.37 – 7.33 (m, 2H), 7.28 – 7.24 (m, 5H), 7.19 – 7.13 (m, 3H), 5.05 (s, 2H), 2.85 – 2.75 (m, 2H), 2.52 – 2.43 (m, 2H), 2.04 – 1.92 (m, 1H), 1.86 – 1.75 (m, 1H).
^{13}C NMR (101 MHz, CDCl$_3$) δ 175.8, 143.7, 140.9, 140.8, 135.4, 128.9, 128.4, 128.0, 127.5, 127.3, 127.2, 126.8, 126.5, 66.3, 52.6, 32.4, 16.8.
HRMS (ESI-TOF): m/z calcd. for [M + Na]$^+$ $C_{24}H_{22}O_2Na^+$ 365.1512; Found: 365.1518.

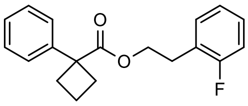

2-Fluorophenethyl 1-phenylcyclobutane-1-carboxylate (3aa)
35.5 mg, 60%, colorless oil, Eluent: pentane/ethyl acetate = 80/1, Rf = 0.4.
^{1}H NMR (300 MHz, CDCl$_3$) δ 7.26 – 7.19 (m, 2H), 7.19 – 7.14 (m, 3H), 7.14 – 7.06 (m, 1H), 6.95 – 6.86 (m, 3H), 4.19 (t, *J* = 6.6 Hz, 2H), 2.82 (td, *J* = 6.6, 1.2 Hz, 2H), 2.75 – 2.64 (m, 2H), 2.47 – 2.33 (m, 2H), 2.00 – 1.85 (m, 1H), 1.84 – 1.68 (m, 1H).
^{13}C NMR (75 MHz, CDCl$_3$) δ 175.9, 161.3 (d, *J* = 245.4 Hz), 143.8, 131.3 (d, *J* = 4.7 Hz), 128.4, 128.3 (d, *J* = 3.1 Hz), 126.6, 126.4, 124.9 (d, *J* = 15.6 Hz), 124.0 (d, *J* = 3.6 Hz), 115.3 (d, *J* = 22.1 Hz), 64.2 (d, *J* = 1.5 Hz), 52.5, 32.4, 28.4 (d, *J* = 2.6 Hz), 16.8.
^{19}F NMR (282 MHz, CDCl$_3$) δ -118.38 – -118.47 (m).
HRMS (ESI-TOF): m/z calcd. for [M + Na]$^+$ $C_{19}H_{19}FO_2Na^+$ 321.1261; Found: 321.1268.

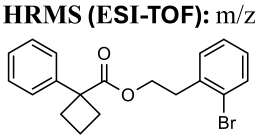

2-Bromophenethyl 1-phenylcyclobutane-1-carboxylate (3ab)
47.2 mg, 66%, colorless oil, Eluent: pentane/ethyl acetate = 80/1, Rf = 0.4.

^{1}H NMR (300 MHz, CDCl$_3$) δ 7.43 (dd, *J* = 7.9, 1.4 Hz, 1H), 7.27 – 7.12 (m, 5H), 7.09 – 6.94 (m, 2H), 6.92 – 6.83 (m, 1H), 4.21 (t, *J* = 6.6 Hz, 2H), 2.92 (t, *J* = 6.6 Hz, 2H), 2.76 – 2.65 (m, 2H), 2.46 – 2.35 (m, 2H), 2.00 – 1.86 (m, 1H), 1.85 – 1.69 (m, 1H).
^{13}C NMR (75 MHz, CDCl$_3$) δ 175.9, 143.8, 137.3, 132.9, 131.3, 128.4, 128.3, 127., 126.6, 126.5, 124.7, 63.8, 52.5, 35.3, 32.4, 16.8.
HRMS (ESI-TOF): m/z calcd. for $[M + Na]^+$ $C_{19}H_{19}BrO_2Na^+$ 381.0461; Found: 381.0470.

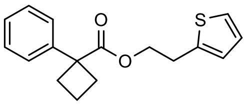

2-(Thiophen-2-yl)ethyl 1-phenylcyclobutane-1-carboxylate (3ac)
40.3mg, 71%, colorless oil, Eluent: pentane/ethyl acetate = 80/1, Rf = 0.3.
^{1}H NMR (300 MHz, CDCl$_3$) δ 7.27 – 7.15 (m, 5H), 7.11 (dd, *J* = 4.9, 3.0 Hz, 1H), 6.76 – 6.67 (m, 2H), 4.17 (t, *J* = 6.6 Hz, 2H), 2.79 (t, *J* = 6.6 Hz, 2H), 2.75 – 2.67 (m, 2H), 2.48 – 2.36 (m, 2H), 1.99 – 1.88 (m, 1H), 1.77 (m, 1H).
^{13}C NMR (75 MHz, CDCl$_3$) δ 175.9, 143.8, 138.1, 128.4, 128.3, 126.7, 126.4, 125.5, 121.7, 64.8, 52.5, 32.4, 29.6, 16.8.
HRMS (ESI-TOF): m/z calcd. for $[M + Na]^+$ $C_{17}H_{18}O_2SNa^+$ 309.0920; Found: 309.0929.

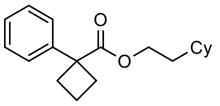

2-Cyclohexylethyl 1-phenylcyclobutane-1-carboxylatec (3ad)
46.2mg, 86%, colorless oil, Eluent: pentane/ethyl acetate = 80/1, Rf = 0.3.
^{1}H NMR (300 MHz, CDCl$_3$) δ 7.28 – 7.20 (m, 4H), 7.17 – 7.12 (m, 1H), 4.00 (t, *J* = 6.6 Hz, 2H), 2.82 – 2.70 (m, 2H), 2.50 – 2.38 (m, 2H), 2.04 – 1.88 (m, 1H), 1.86 – 1.72 (m, 1H), 1.59 – 1.46 (m, 5H), 1.34 (q, *J* = 6.7 Hz, 2H), 1.13 – 0.98 (m, 4H), 0.85 – 0.69 (m, 2H).
^{13}C NMR (75 MHz, CDCl$_3$) δ 176.1, 144.0, 128.3, 126.6, 126.4, 63.2, 52.6, 35.9, 34.5, 33.2, 32.4, 26.6, 26.3, 16.8.
HRMS (ESI-TOF): m/z calcd. for $[M + Na]^+$ $C_{19}H_{26}O_2Na^+$ 309.1825; Found: 309.1831.

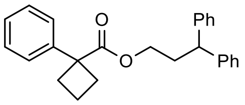

3,3-Diphenylpropyl 1-phenylcyclobutane-1-carboxylate (3ae)
59.4mg, 80%, colorless oil, Eluent: pentane/ethyl acetate = 80/1, Rf = 0.2.
^{1}H NMR (300 MHz, CDCl$_3$) δ 7.33 – 7.23 (m, 4H), 7.22 – 7.11 (m, 5H), 7.09 – 7.02 (m, 2H), 6.98 – 6.94 (m, 4H), 3.87 (t, *J* = 6.3 Hz, 2H), 3.69 (t, *J* = 7.9 Hz, 1H), 2.82 – 2.68 (m, 2H), 2.52 – 2.37 (m, 2H), 2.18 (dt, *J* = 8.0, 6.3 Hz, 2H), 2.04 – 1.88 (m, 1H), 1.86 – 1.72 (m, 1H).
^{13}C NMR (75 MHz, CDCl$_3$) δ 175.8, 144.1, 144.0, 128.6, 128.4, 127.9, 126.8, 126.5, 126.4, 63.1, 52.6, 47.1, 34.3, 32.3, 16.8.
HRMS (ESI-TOF): m/z calcd. for $[M + Na]^+$ $C_{26}H_{26}O_2Na^+$ 393.1825; Found: 393.1828.

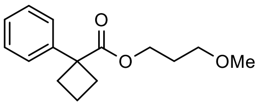

3-Methoxypropyl 1-phenylcyclobutane-1-carboxylate (3af)
40.8mg, 82%, colorless oil, Eluent: pentane/ethyl acetate = 50/1, Rf = 0.2.
^{1}H NMR (400 MHz, CDCl$_3$) δ 7.35 – 7.28 (m, 4H), 7.25 – 7.20 (m, 1H), 4.13 (t, *J* = 6.3 Hz, 2 H), 3.26 – 3.22 (m, 5H), 2.90 – 2.78 (m, 2H), 2.58 – 2.45 (m, 2H), 2.12 – 1.96 (m, 1H), 1.92 – 1.83 (m, 1H), 1.79 (p, *J* = 6.3 Hz, 2H).
^{13}C NMR (101 MHz, CDCl$_3$) δ 175.9, 143.9, 128.3, 126.6, 126.3, 68.9, 62.0, 58.7, 52.6, 32.4, 29.0, 16.8.
HRMS (ESI-TOF): m/z calcd. for $[M + Na]^+$ $C_{15}H_{20}O_3Na^+$ 271.1305; Found: 271.1312.

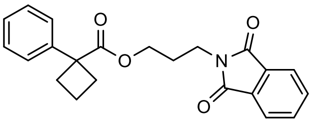

3-(1,3-Dioxoisoindolin-2-yl)propyl 1-phenylcyclobutane-1-carboxylate (3ag)
52.5 mg, 72%, colorless oil, Eluent: pentane/ethyl acetate = 10/1, Rf = 0.4.
^{1}H NMR (300 MHz, CDCl$_3$) δ 7.87 – 7.80 (m, 2H), 7.77 – 7.66 (m, 2H), 7.36 – 7.27 (m, 4H), 7.24 – 7.17 (m, 1H), 4.09 (t, *J* = 6.2 Hz, 2H), 3.67 (t, *J* = 7.0 Hz, 2H), 2.92 – 2.81 (m, 2H), 2.57 – 2.44 (m, 2H), 2.13 – 2.02 (m, 1H), 2.00 – 1.81 (m, 3H).
^{13}C NMR (75 MHz, CDCl$_3$) δ 175.9, 168.3, 143.7, 134.1, 132.2, 128.3, 126.6, 126.4, 123.4, 62.2, 52.5, 34.9, 32.4, 27.9, 16.8.
HRMS (ESI-TOF): m/z calcd. for $[M + Na]^+$ $C_{22}H_{21}NO_4Na^+$ 386.1363; Found: 386.1369.

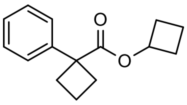

Cyclobutyl 1-phenylcyclobutane-1-carboxylate (3ah)
32.4 mg, 70%, colorless oil, Eluent: pentane/ethyl acetate = 100/1, Rf = 0.3.
^{1}H NMR (300 MHz, CDCl$_3$) δ 7.26 – 7.21 (m, 4H), 7.19 – 7.12 (m, 1H), 4.85 (ttd, *J* = 8.1, 7.1, 0.9 Hz, 1H), 2.80 – 2.70 (m, 2H), 2.48 – 2.36 (m, 2H), 2.26 – 2.15 (m, 2H), 1.98 – 1.75 (m, 4H), 1.71 – 1.59 (m, 1H), 1.55 – 1.44 (m, 1H).
^{13}C NMR (75 MHz, CDCl$_3$) δ 175.5, 143.9, 128.3, 126.6, 126.4, 69.4, 52.4, 32.4, 30.2, 16.7, 13.6.
HRMS (ESI-TOF): m/z calcd. for $[M + Na]^+$ $C_{15}H_{18}O_2Na^+$ 253.1199; Found: 253.1208.

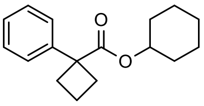

Cyclohexyl 1-phenylcyclobutane-1-carboxylate (3ai)
24.4 mg, 47%, colorless oil, Eluent: pentane/ethyl acetate = 80/1, Rf = 0.3.
^{1}H NMR (300 MHz, CDCl$_3$) δ 7.35 – 7.26 (m, 4H), 7.24 – 7.18 (m, 1H), 4.73 (td, *J* = 8.1, 3.7 Hz, 1H), 2.90 – 2.78 (m, 2H), 2.57 – 2.43 (m, 2H), 2.12 – 1.99 (m, 1H), 1.91 – 1.83 (m, 1H), 1.74 – 1.64 (m, 2H), 1.58 (d, *J* = 7.0 Hz, 2H), 1.48 – 1.24 (m, 6H).
^{13}C NMR (75 MHz, CDCl$_3$) δ 175.5, 144.2, 128.2, 126.5, 126.3, 72.6, 52.8, 32.4, 31.2, 25.5, 23.3, 16.8.
HRMS (ESI-TOF): m/z calcd. for $[M + Na]^+$ $C_{17}H_{22}O_2Na^+$ 281.1512; Found: 281.1519.

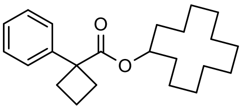

Cyclododecyl 1-phenylcyclobutane-1-carboxylate (3aj)
43.1 mg, 64%, colorless oil, Eluent: pentane/ethyl acetate = 80/1, Rf = 0.3.
^{1}H NMR (300 MHz, CDCl$_3$) δ 7.33 – 7.28 (m, 4H), 7.24 – 7.18 (m, 1H), 4.95 (tt, *J* = 7.3, 4.6 Hz, 1H), 2.88 – 2.77 (m, 2H), 2.55 – 2.44 (m, 2H), 2.12 – 1.96 (m, 1H), 1.93 – 1.81 (m, 1H), 1.66 – 1.56 (m, 2H), 1.44 – 1.20 (m, 20H).
^{13}C NMR (75 MHz, CDCl$_3$) δ 175.8, 144.2, 128.2, 126.5, 126.3, 72.7, 52.8, 32.4, 28.9, 24.2, 24.0, 23.5, 23.3, 20.8, 16.8.
HRMS (ESI-TOF): m/z calcd. for $[M + Na]^+$ $C_{23}H_{34}O_2Na^+$ 365.2451; Found: 365.2458.

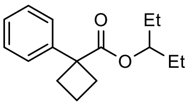

Pentan-3-yl 1-phenylcyclobutane-1-carboxylate (3ak)
36.9 mg, 75%, colorless oil, Eluent: pentane/ethyl acetate = 100/1, Rf = 0.3.
^{1}H NMR (300 MHz, CDCl$_3$) δ 7.31 (d, *J* = 4.5 Hz, 4H), 7.24 – 7.19 (m, 1H), 4.70 (tt, *J* = 7.0, 5.3 Hz, 1H), 2.90 – 2.80 (m, 2H), 2.57 – 2.46 (m, 2H), 2.12 – 1.97 (m, 1H), 1.94 – 1.80 (m, 1H), 1.50 – 1.44 (m, 3H), 0.95 – 0.81 (m, 1H), 0.71 (t, *J* = 7.5 Hz, 6H).
^{13}C NMR (75 MHz, CDCl$_3$) δ 175.9, 144.2, 128.2, 126.5, 126.3, 77.0, 52.9, 32.4, 26.6, 16.9, 9.5.
HRMS (ESI-TOF): m/z calcd. for $[M + Na]^+$ $C_{16}H_{22}O_2Na^+$ 269.1512; Found: 269.1518.

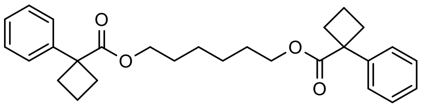

Hexane-1,6-diyl bis(1-phenylcyclobutane-1-carboxylate) (3al)
42.0 mg, 96%, white solid, Eluent: pentane/ethyl acetate = 100/1, Rf = 0.3.
^{1}H NMR (400 MHz, CDCl$_3$) δ 7.34 – 7.28 (m, 8H), 7.23 – 7.19 (m, 2H), 3.99 (t, *J* = 6.5 Hz, 4H), 2.88 – 2.79 (m, 4H), 2.56 – 2.48 (m, 4H), 2.12 – 1.97 (m, 2H), 1.94 – 1.82 (m, 2H), 1.50 – 1.42 (m, 4H), 1.14 – 1.07 (m, 4H).
^{13}C NMR (101 MHz, CDCl$_3$) δ 175.9, 143.9, 128.2, 126.5, 126.2, 64.7, 52.5, 32.3, 28.4, 25.2, 16.7.
HRMS (ESI-TOF): m/z calcd. for $[M + Na]^+$ $C_{28}H_{34}O_4Na^+$ 457.2349; Found: 457.2358.

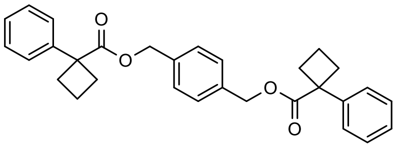

1,4-Phenylenebis(methylene) bis(1-phenylcyclobutane-1-carboxylate) (3am)
23.1mg, 51%, white solid, Eluent: pentane/ethyl acetate = 100/1, Rf = 0.3.
^{1}H NMR (400 MHz, CDCl$_3$) δ 7.26 – 7.21 (m, 8H), 7.19 – 7.14 (m, 2H), 6.99 (s, 4H), 4.98 (s, 4H), 2.82 – 2.74 (m, 4H), 2.50 – 2.42 (m, 4H), 2.02 – 1.90 (m, 2H), 1.85 – 1.75 (m, 2H).
^{13}C NMR (101 MHz, CDCl$_3$) δ 175.7, 143.6, 136.0, 128.4, 127.6, 126.8, 126.5, 66.2, 52.6, 32.4, 16.8.
HRMS (ESI-TOF): m/z calcd. for $[M + Na]^+$ $C_{30}H_{30}O_4Na^+$ 477.2036; Found: 477.2045.

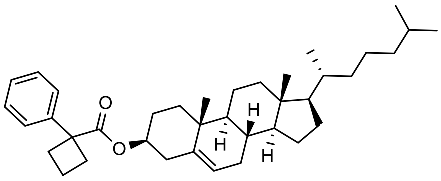

(3S,8S,9S,10R,13R,14S,17R)-10,13-Dimethyl-17-((R)-6-methylheptan-2-yl)-2,3,4,7,8,9,10,11,12,13,14,15,16,17-tetradecahydro-1H-cyclopenta[a]phenanthren-3-yl 1-phenylcyclobutane-1-carboxylate (3an, from Cholesterol)
57.9mg, 53%, colorless oil, Eluent: pentane/ethyl acetate = 80/1, Rf = 0.3.
^{1}H NMR (400 MHz, CDCl$_3$) δ 7.33 – 7.31 (m, 4H), 7.24 – 7.20 (m, 1H), 5.35 – 5.30 (m, 1H), 4.63 – 4.52 (m, 1H), 2.87 – 2.79 (m, 2H), 2.56 – 2.44 (m, 2H), 2.21 (d, *J* = 7.8 Hz, 2H), 2.08 – 1.72 (m, 8H), 1.60 – 1.31 (m, 11H), 1.20 – 0.96 (m, 12H), 0.91 (d, *J* = 6.6 Hz, 3H), 0.87 (dd, *J* = 6.6, 1.8 Hz, 6H), 0.67 (s, 3H).
^{13}C NMR (101 MHz, CDCl$_3$) δ 175.5, 144.0, 139.8 (d, *J* = 3.5 Hz), 128.3, 126.5, 126.3, 122.7, 74.3, 56.8, 56.3, 52.7, 50.1, 42.4, 39.8, 39.6, 37.9, 37.0, 36.7, 36.3, 35.9, 32.5, 32.0, 31.9, 28.4, 28.1, 27.6, 24.4, 24.0, 23.0, 22.7, 21.2, 19.5, 18.8, 16.7, 12.0.
HRMS (ESI-TOF): m/z calcd. for $[M + Na]^+$ $C_{38}H_{56}O_2Na^+$ 567.4173; Found: 567.4181.

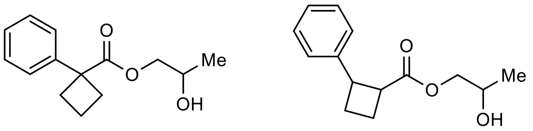

3ao **4ao**

*the mixture of **3ao** and **4ao** (ratio = 3.95 : 1)*

2-Hydroxypropyl 1-phenylcyclobutane-1-carboxylate (3ao, major)
2-Hydroxypropyl 2-phenylcyclobutane-1-carboxylate (4ao, minor)
16.4 mg, 70%, colorless oil, Eluent: pentane/ethyl acetate = 40/1, Rf = 0.3
^{1}H NMR (300 MHz, CDCl$_3$) δ 7.27 – 7.11 (m, 5H), 5.03 – 4.91 (m, 0.75H), 4.15 – 3.94 (m, 0.61H), 3.48 – 3.33 (m, 1.65H), 2.84 – 2.70 (m, 2H), 2.55 – 2.36 (m, 2H), 2.07 – 1.92 (m, 1H), 1.85 – 1.73 (m, 1H), 1.28 (d, *J* = 6.3 Hz, 0.61H), 1.14 (d, *J* = 6.4 Hz, 2.41H).
^{13}C NMR (75 MHz, CDCl$_3$) δ 175.5, 175.3, 143.6, 143.5, 128.4, 128.3, 126.8, 126.7, 126.4, 126.3, 70.0, 68.8, 54.0, 52.7, 46.9, 32.4, 32.4, 21.4, 17.4, 16.8, 16.7.
HRMS (ESI-TOF): m/z calcd. for $[M + Na]^+$ $C_{14}H_{18}O_3Na^+$ 257.1148; Found: 257.1156.

6.Synthetic transformations of 3a

6.1 Reduction of compound 3a

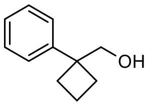

(1-Phenylcyclobutyl)methanol (4)

4 were synthesized according to the reported procedure[2].To a suspension of lithium aluminum hydride (30 mg, 0.8 mmol) in THF (2 mL), a solution of 3-phenylpropyl 1-phenylcyclobutane-1-carboxylate (0.2 mmol, 58.8 mg) in THF (2 mL) was added dropwise at 25 ºC. The mixture was reacted to reflux (oil bath) for 4 h and quenched with water. The aqueous layer was extracted with ethyl acetate three times, and the combined organic layer was dried and concentrated to provide the crude product. Flash chromatography on silica gel using pentane/ethyl acetate = 5/1, TLC Rf = 0.4; gave the product **(1-phenylcyclobutyl)methanol (**25.8mg, 80% yield, colorless oil).

^{1}H NMR (300 MHz, $CDCl_3$) δ 7.37 – 7.31 (m, 2H), 7.25 – 7.13 (m, 3H), 3.74 (s, 2H), 2.40 – 2.20 (m, 4H), 2.17 – 2.03 (m, 1H), 1.96 – 1.83 (m, 1H), 1.34 (s, 1H).
^{13}C NMR (75 MHz, $CDCl_3$) δ 147.7, 128.4, 126.1, 126.0, 70.9, 48.0, 29.7, 16.1.
HRMS (ESI-TOF): m/z calcd. for $[M + Na]^+$ $C_{11}H_{14}ONa^+$ 185.0937; Found: 185.0946.

6.2 Hydrolysis of compound 3a

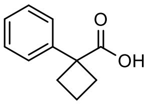

5 were synthesized according to the reported procedure[2]. The adduct 3-phenylpropyl 1-phenylcyclobutane-1-carboxylate (0.2 mmol, 58.8 mg) and potassium hydroxide (73 mg, 1.3 mmol) was dissolved in toluene (5 mL), and then the solution was heated to reflux (oil bath) for 2 h. After the mixture was cooled to 25° C, water (10 mL) was added, and the mixture was extracted with ethyl acetate (2× 10 mL). The aqueous phase was acidified with 1.0 M aqueous HCl to pH = 1. The acidified aqueous layer was extracted with ethyl acetate (3 × 10 mL), and the combined organic phase was washed with water and brine and dried over anhydrous Na_2SO_4. The solvent was evaporated and the residue was purified by flash column chromatography on silica gel, eluting with 20/1 DCM/MeOH, TLC Rf = 0.30 (5% DCM/MeOH); gave the product **1-phenylcyclobutane-1-carboxylic acid** 29.7 mg, 84% yield, colorless oil.

^{1}H NMR (300 MHz, $CDCl_3$) δ 7.36 – 7.28 (m, 4H), 7.26 – 7.20 (m, 1H), 2.90 – 2.77 (m, 2H), 2.62 – 2.45 (m, 2H), 2.12 – 1.99 (m, 1H), 1.93 – 1.79 (m, 1H).
^{13}C NMR (75 MHz, $CDCl_3$) δ 182.6, 143.3, 128.5, 126.9, 126.5, 52.3, 32.4, 16.8.
The observed characterization data (^{1}H, ^{13}C NMR) was consistent with that previously reported[3].

7.Reference

(1) (a) Miki, Y.; Tomita, N.; Ban, K.; Sajiki, H. and Sawama, Y. Synthesis of 1-Pyrroline by Denitrogenative Ring Expansion of Cyclobutyl Azides under Thermal Conditions. *Adv. Synth. Catal.* **2021**, 363, 3481-3484. (b) Wu, M.; Yan, C.; Zhuang, D. and Yan, R. Metal-free C–S bond formation in elemental sulfur and cyclobutanol derivatives: The synthesis of substituted thiophenes. *Org. Lett.* **2022**, 24, 5309-5313.

(2) Chen, Y. J.; Hu, T. J.; Feng, C. G. and Lin, G. Q. Synthesis of chiral cyclobutanes via rhodium/diene-catalyzed asymmetric 1,4-addition: a dramatic ligand effect on the diastereoselectivity. *Chem. Commun.* **2015**, 51, 8773-8776.

(3) Dubois, M. A.; Smith, M. A.; White, A. J.; Lee Wei Jie, A.; Mousseau, J. J.; Choi, C. and Bull, J. A. Short synthesis of oxetane and azetidine 3-Aryl-3-carboxylic acid derivatives by selective furan oxidative cleavage. *Org. Lett.* **2020**, 22, 5279-5283.

8. NMR Spectra

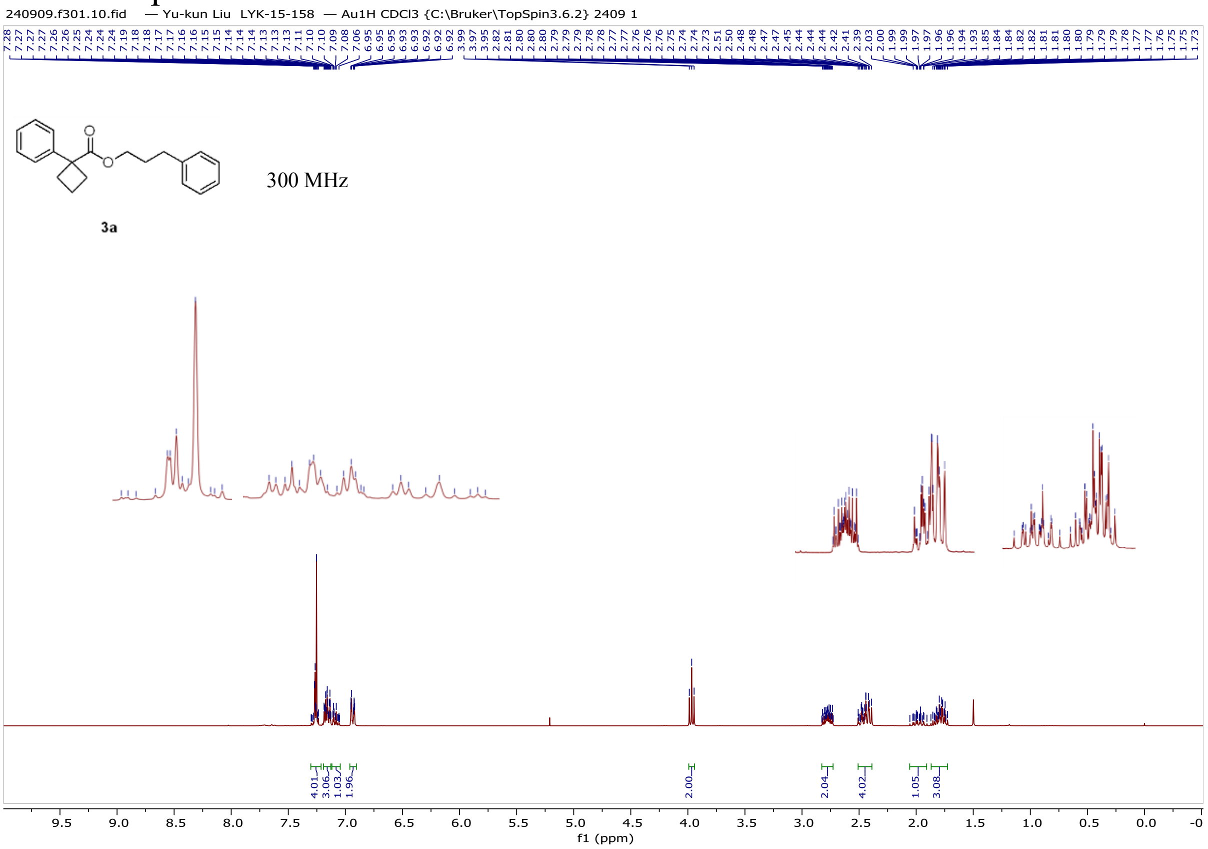

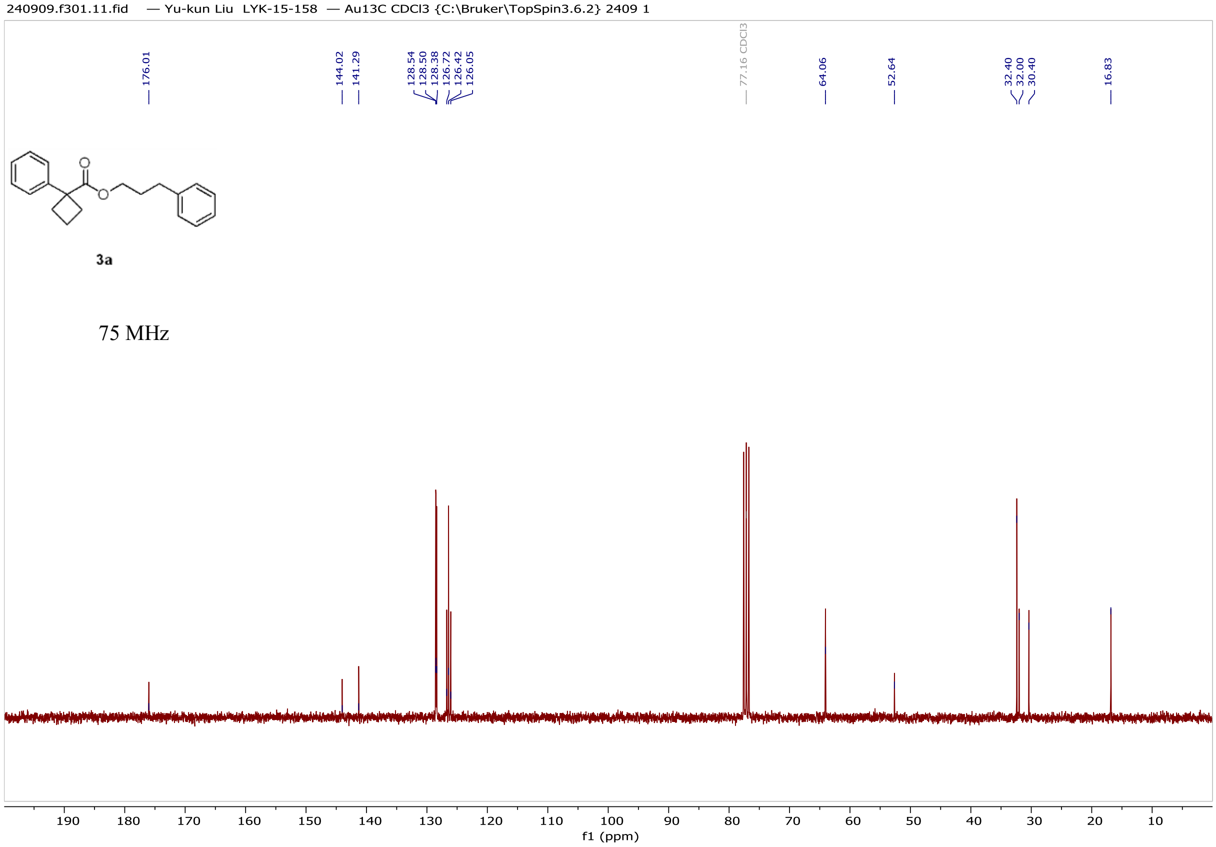

241111.402.10.fid — Yu-kun Liu LYK15-363 — Au1H CDCl3 {C:\Bruker\TopSpin3.6.2} 2411 2

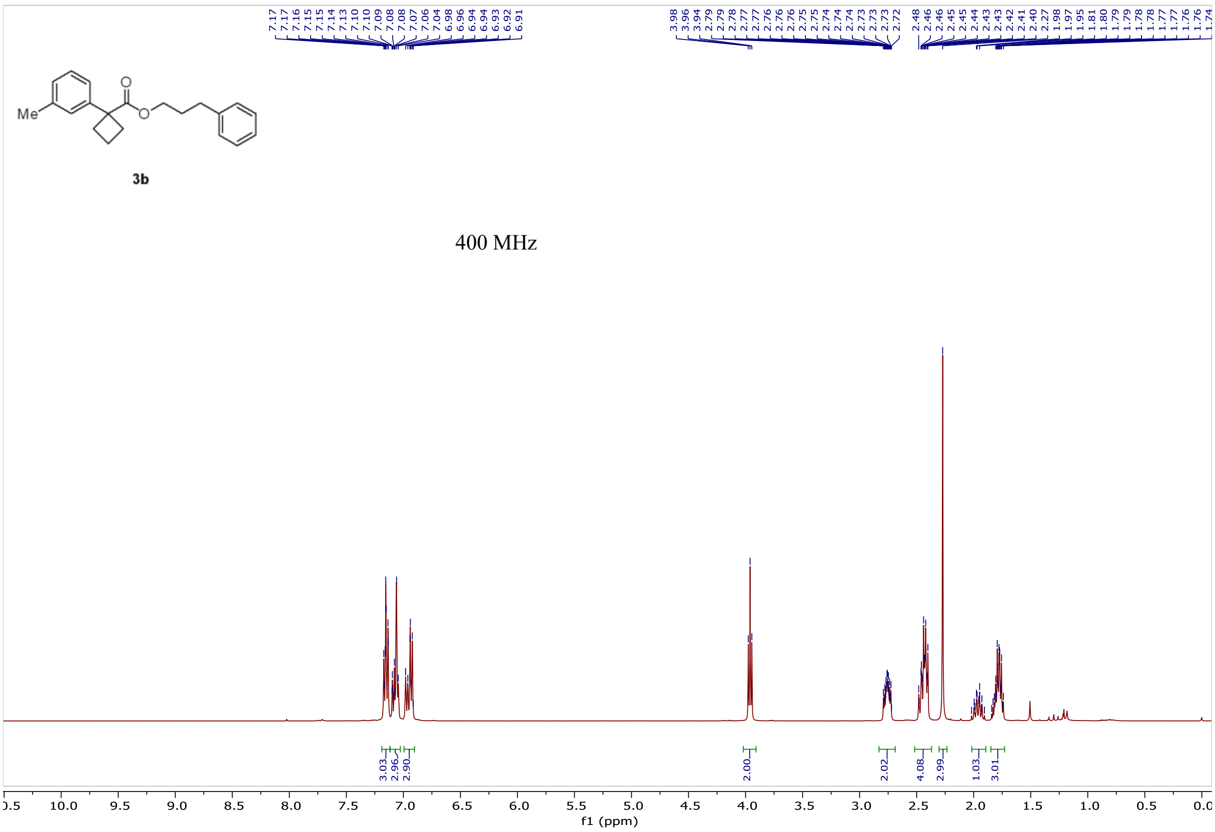

241111.402.11.fid — Yu-kun Liu LYK15-363 — Au13C CDCl3 {C:\Bruker\TopSpin3.6.2} 2411 2

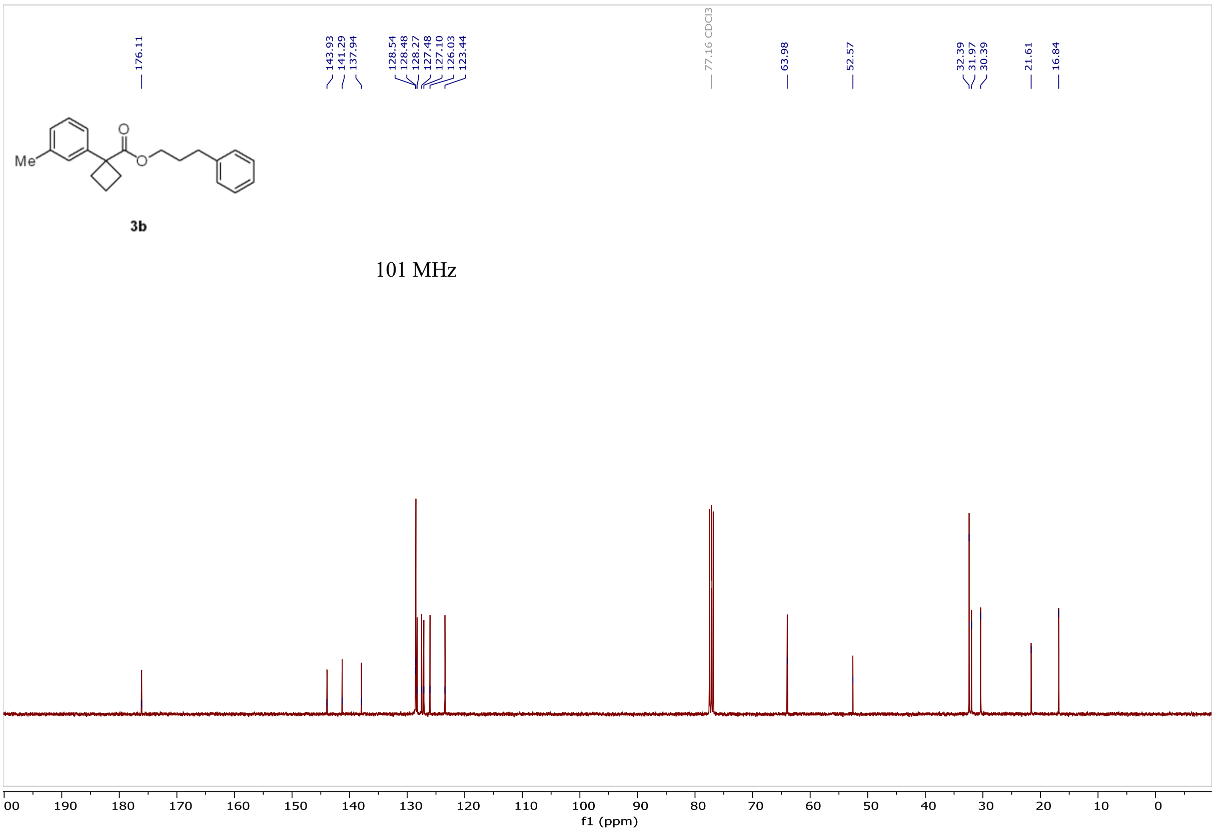

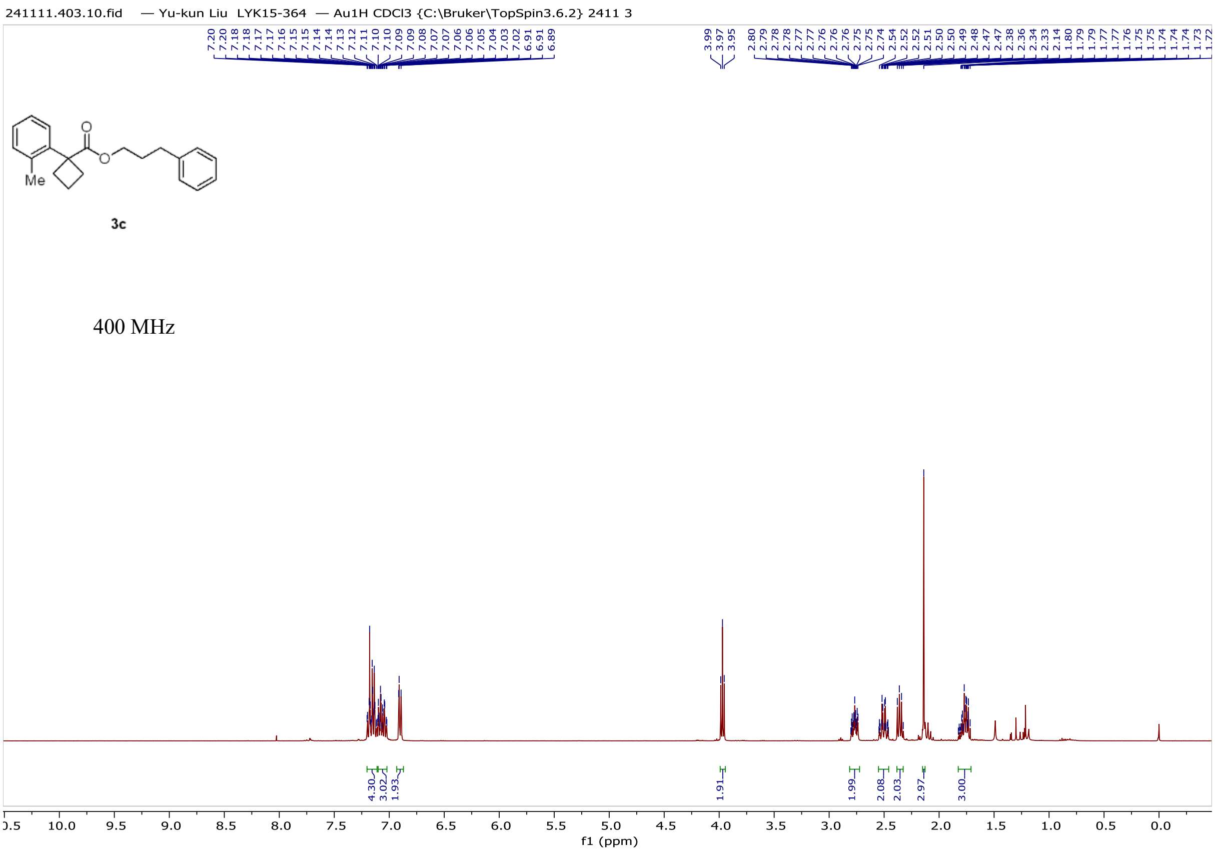
241111.403.10.fid — Yu-kun Liu LYK15-364 — Au1H CDCl3 {C:\Bruker\TopSpin3.6.2} 2411 3
O
O
Me
3c
400 MHz
f1 (ppm)

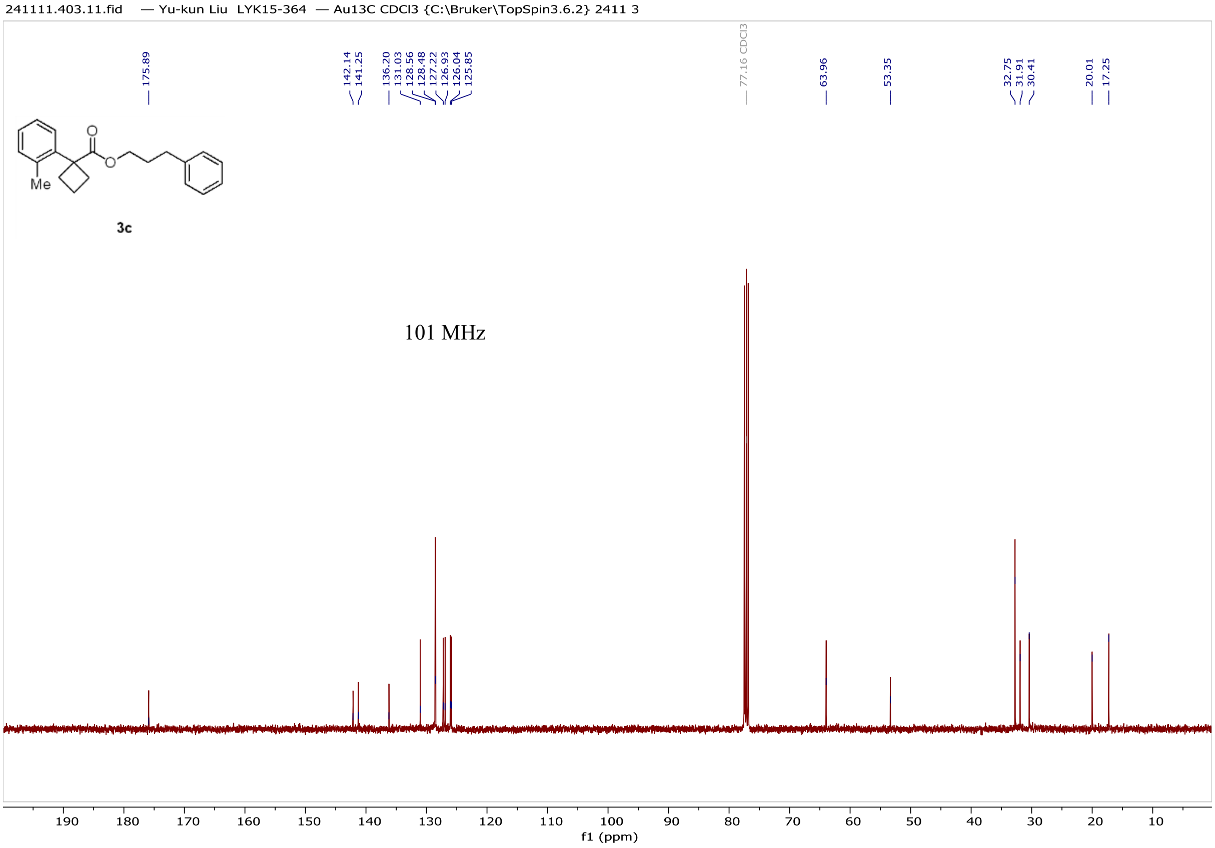
241111.403.11.fid — Yu-kun Liu LYK15-364 — Au13C CDCl3 {C:\Bruker\TopSpin3.6.2} 2411 3
O
O
Me
3c
101 MHz
f1 (ppm)

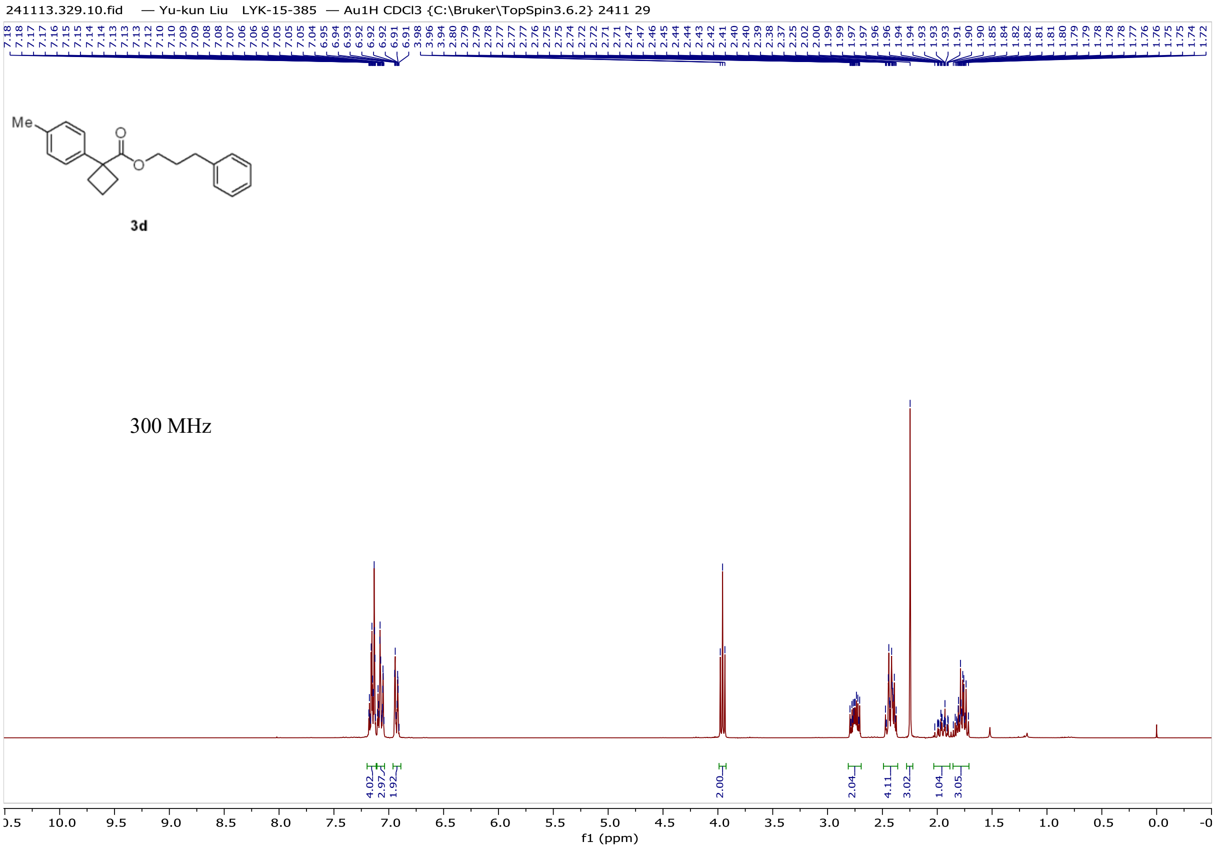
241113.329.10.fid — Yu-kun Liu LYK-15-385 — Au1H CDCl3 {C:\Bruker\TopSpin3.6.2} 2411 29
7.18 7.18 7.17 7.16 7.15 7.15 7.14 7.13 7.13 7.13 7.12 7.10 7.09 7.09 7.08 7.08 7.07 7.06 7.06 7.05 7.05 7.05 7.04 6.95 6.94 6.93 6.92 6.92 6.92 6.91 6.91 3.98 3.96 3.94 2.80 2.79 2.79 2.78 2.78 2.77 2.77 2.76 2.75 2.75 2.74 2.72 2.72 2.71 2.71 2.47 2.47 2.46 2.45 2.44 2.44 2.43 2.42 2.41 2.40 2.40 2.39 2.38 2.37 2.25 2.02 2.00 1.99 1.99 1.97 1.97 1.96 1.96 1.94 1.94 1.93 1.93 1.93 1.91 1.90 1.85 1.84 1.82 1.82 1.81 1.81 1.80 1.79 1.79 1.78 1.78 1.77 1.76 1.76 1.75 1.75 1.74 1.72
Me
O
O
3d
300 MHz
4.02 2.97 1.92 2.00 2.04 4.11 3.02 1.04 3.05
f1 (ppm)

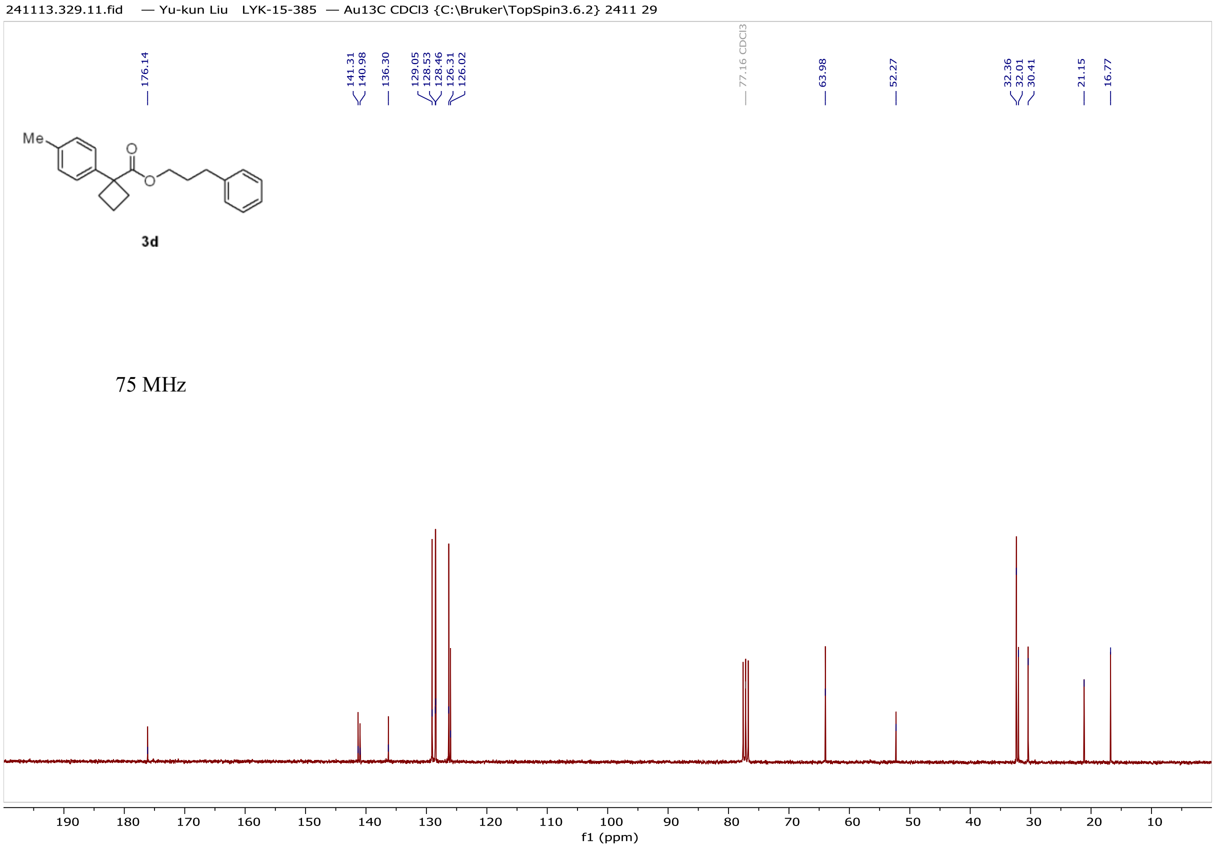
241113.329.11.fid — Yu-kun Liu LYK-15-385 — Au13C CDCl3 {C:\Bruker\TopSpin3.6.2} 2411 29
176.14
141.31 140.98
136.30
129.05 128.53 128.46 126.31 126.02
77.16 CDCl3
63.98
52.27
32.36 32.01 30.41
21.15
16.77
Me
O
O
3d
75 MHz
f1 (ppm)

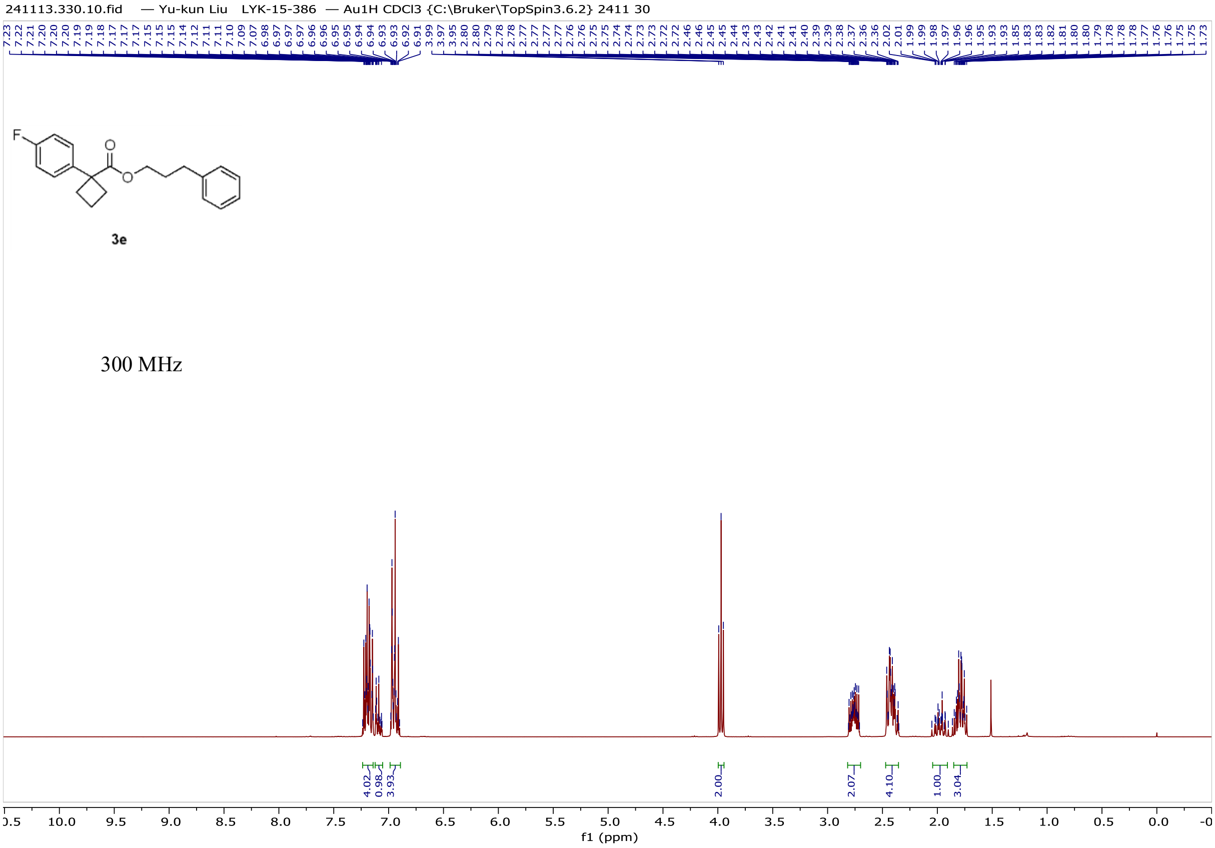
241113.330.10.fid — Yu-kun Liu LYK-15-386 — Au1H CDCl3 {C:\Bruker\TopSpin3.6.2} 2411 30
F
O
O
3e
300 MHz
4.02
0.98
3.93
2.00
2.07
4.10
1.00
3.04
f1 (ppm)

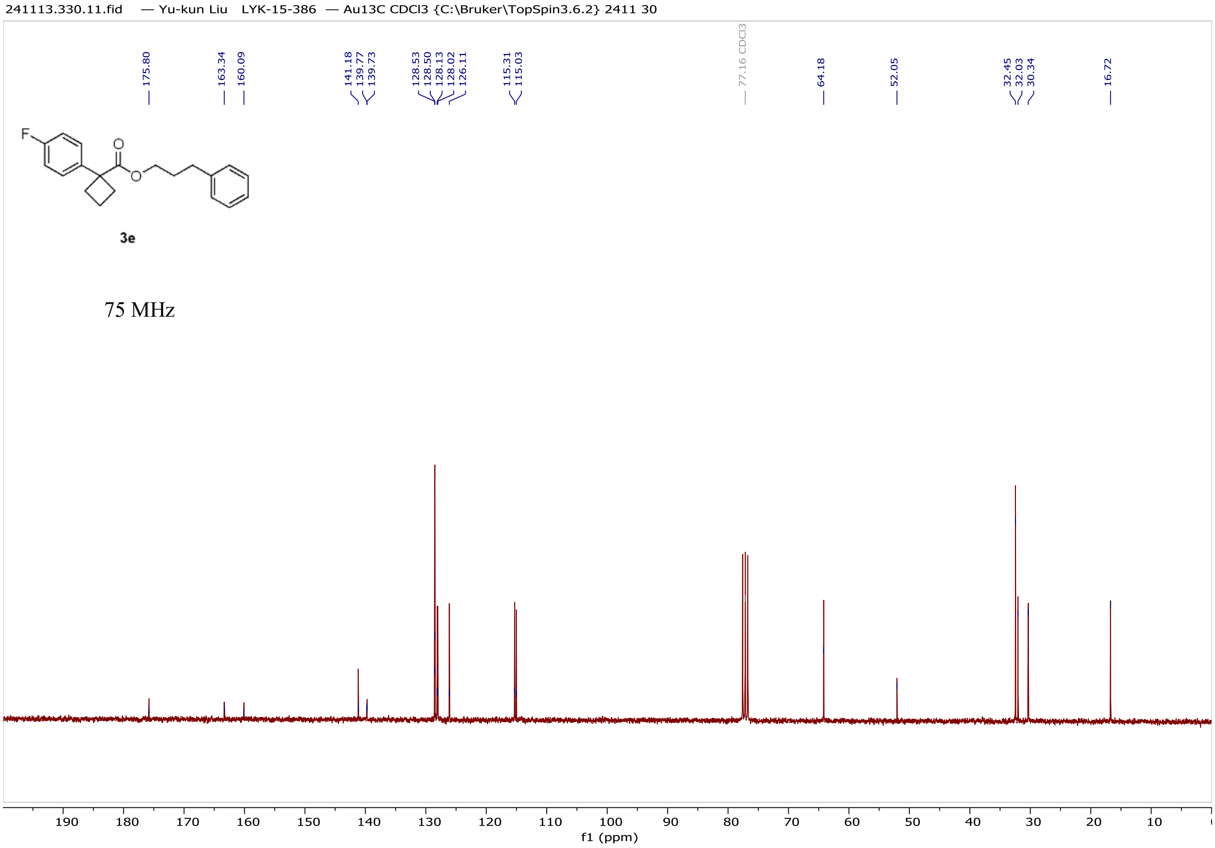
241113.330.11.fid — Yu-kun Liu LYK-15-386 — Au13C CDCl3 {C:\Bruker\TopSpin3.6.2} 2411 30
175.80
163.34
160.09
141.18
139.77
139.73
128.53
128.50
128.13
128.02
126.11
115.31
115.03
77.16 CDCl3
64.18
52.05
32.45
32.03
30.34
16.72
F
O
O
3e
75 MHz
f1 (ppm)

241113.330.12.fid — Yu-kun Liu LYK-15-386 — Au19F CDCl3 {C:\Bruker\TopSpin3.6.2} 2411 30

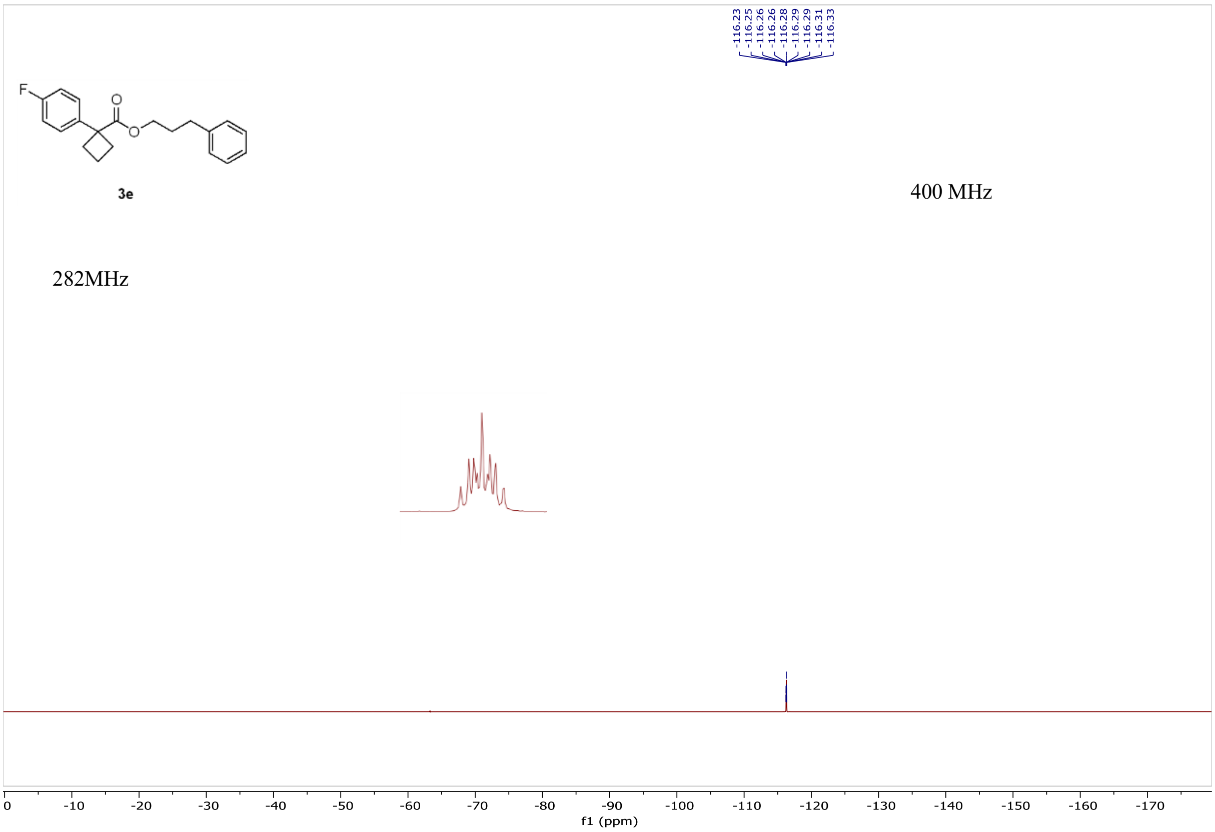

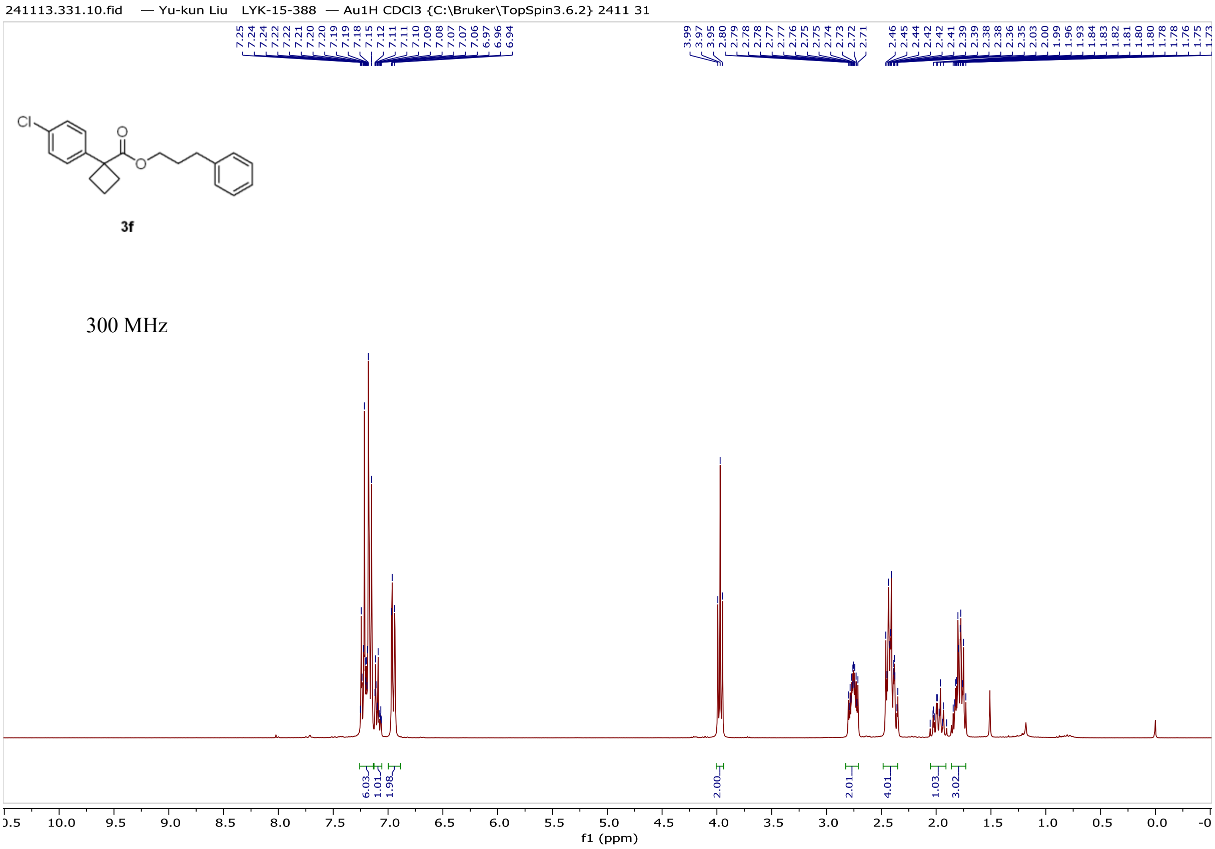
241113.331.10.fid — Yu-kun Liu LYK-15-388 — Au1H CDCl3 {C:\Bruker\TopSpin3.6.2} 2411 31
3f
300 MHz
6.03
1.01
1.98
2.00
2.01
4.01
1.03
3.02
f1 (ppm)

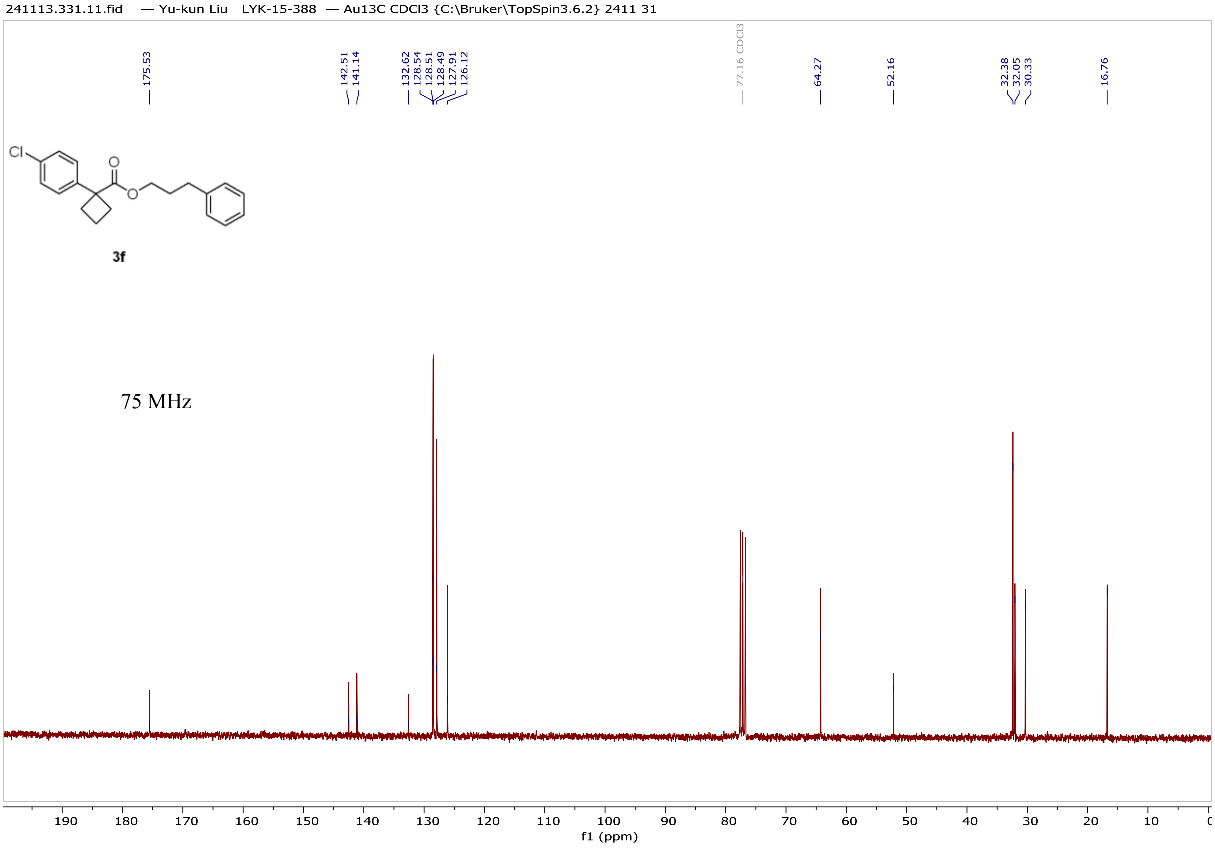
241113.331.11.fid — Yu-kun Liu LYK-15-388 — Au13C CDCl3 {C:\Bruker\TopSpin3.6.2} 2411 31
175.53
142.51
141.14
132.62
128.54
128.51
128.49
127.91
126.12
77.16 CDCl3
64.27
52.16
32.38
32.05
30.33
16.76
3f
75 MHz
f1 (ppm)

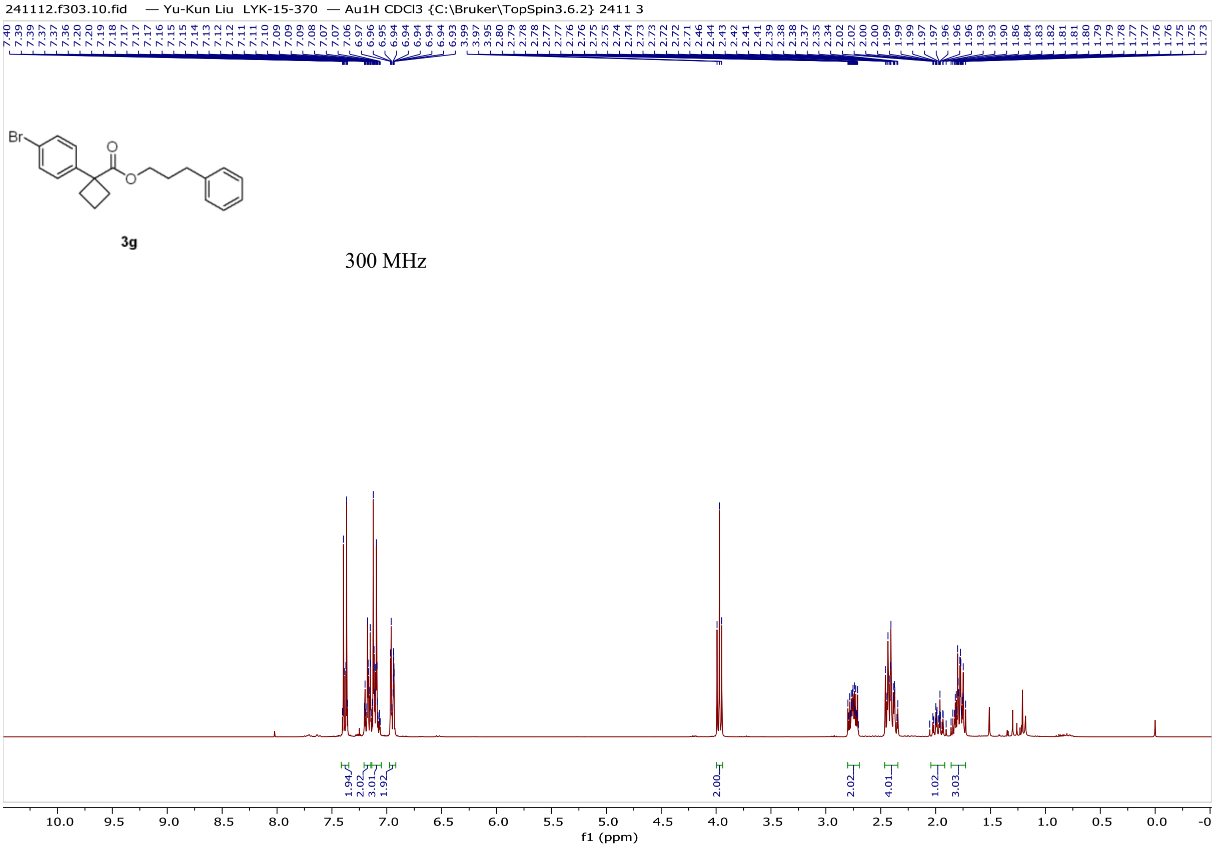
241112.f303.10.fid — Yu-Kun Liu LYK-15-370 — Au1H CDCl3 {C:\Bruker\TopSpin3.6.2} 2411 3
7.40 7.39 7.39 7.37 7.37 7.36 7.20 7.20 7.19 7.18 7.17 7.17 7.17 7.16 7.15 7.15 7.14 7.13 7.12 7.12 7.11 7.11 7.10 7.09 7.09 7.09 7.08 7.07 7.07 7.06 6.97 6.96 6.95 6.94 6.94 6.94 6.94 6.94 6.93 3.99 3.97 3.95 2.80 2.79 2.78 2.78 2.77 2.76 2.76 2.75 2.75 2.74 2.74 2.73 2.73 2.72 2.72 2.71 2.46 2.44 2.43 2.42 2.41 2.39 2.38 2.38 2.37 2.35 2.34 2.02 2.02 2.00 2.00 1.99 1.99 1.99 1.97 1.97 1.96 1.96 1.96 1.93 1.93 1.90 1.86 1.84 1.83 1.82 1.81 1.81 1.80 1.79 1.79 1.78 1.77 1.77 1.76 1.75 1.75 1.73
Br
O
O
3g
300 MHz
1.94
2.02
3.01
1.92
2.00
2.02
4.01
1.02
3.03
10.0 9.5 9.0 8.5 8.0 7.5 7.0 6.5 6.0 5.5 5.0 4.5 4.0 3.5 3.0 2.5 2.0 1.5 1.0 0.5 0.0
f1 (ppm)

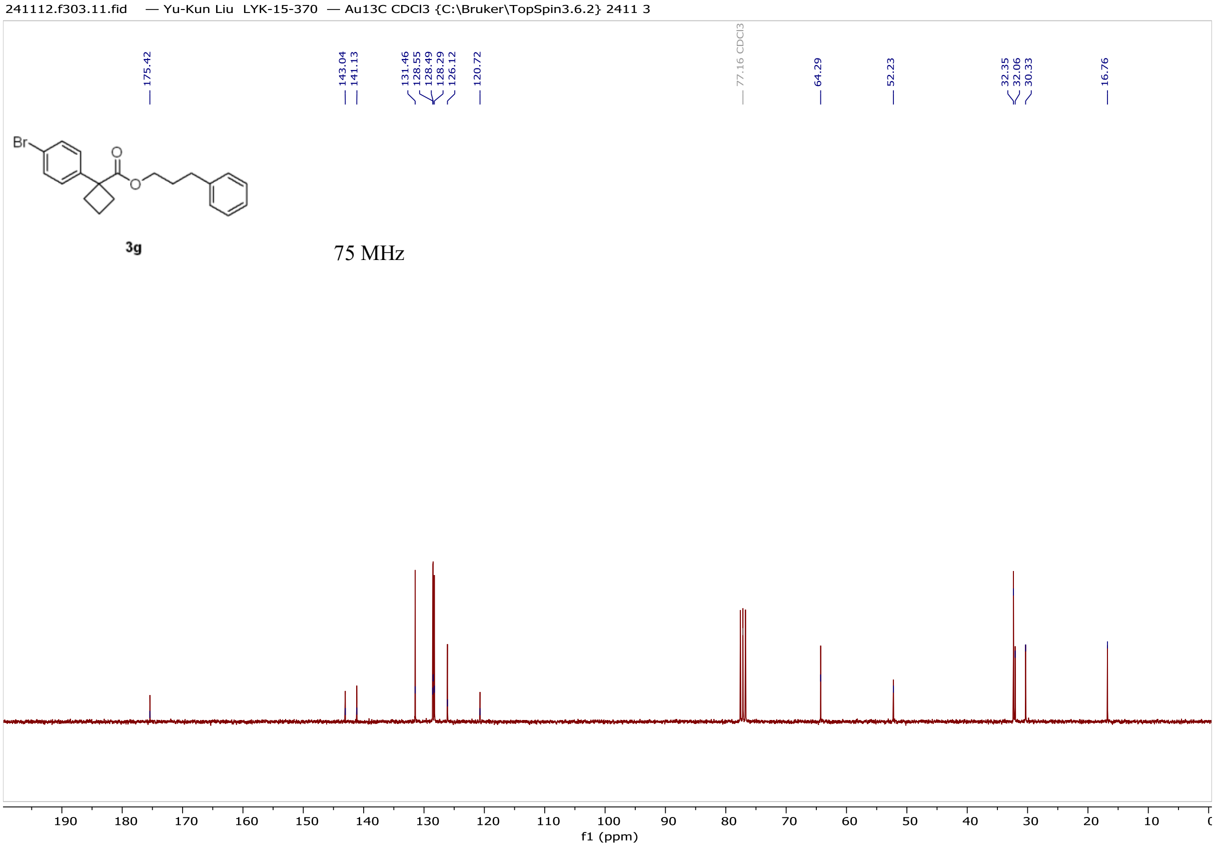
241112.f303.11.fid — Yu-Kun Liu LYK-15-370 — Au13C CDCl3 {C:\Bruker\TopSpin3.6.2} 2411 3
175.42
143.04 141.13
131.46 128.55 128.49 128.29 126.12
120.72
77.16 CDCl3
64.29
52.23
32.35 32.06 30.33
16.76
Br
O
O
3g
75 MHz
190 180 170 160 150 140 130 120 110 100 90 80 70 60 50 40 30 20 10
f1 (ppm)

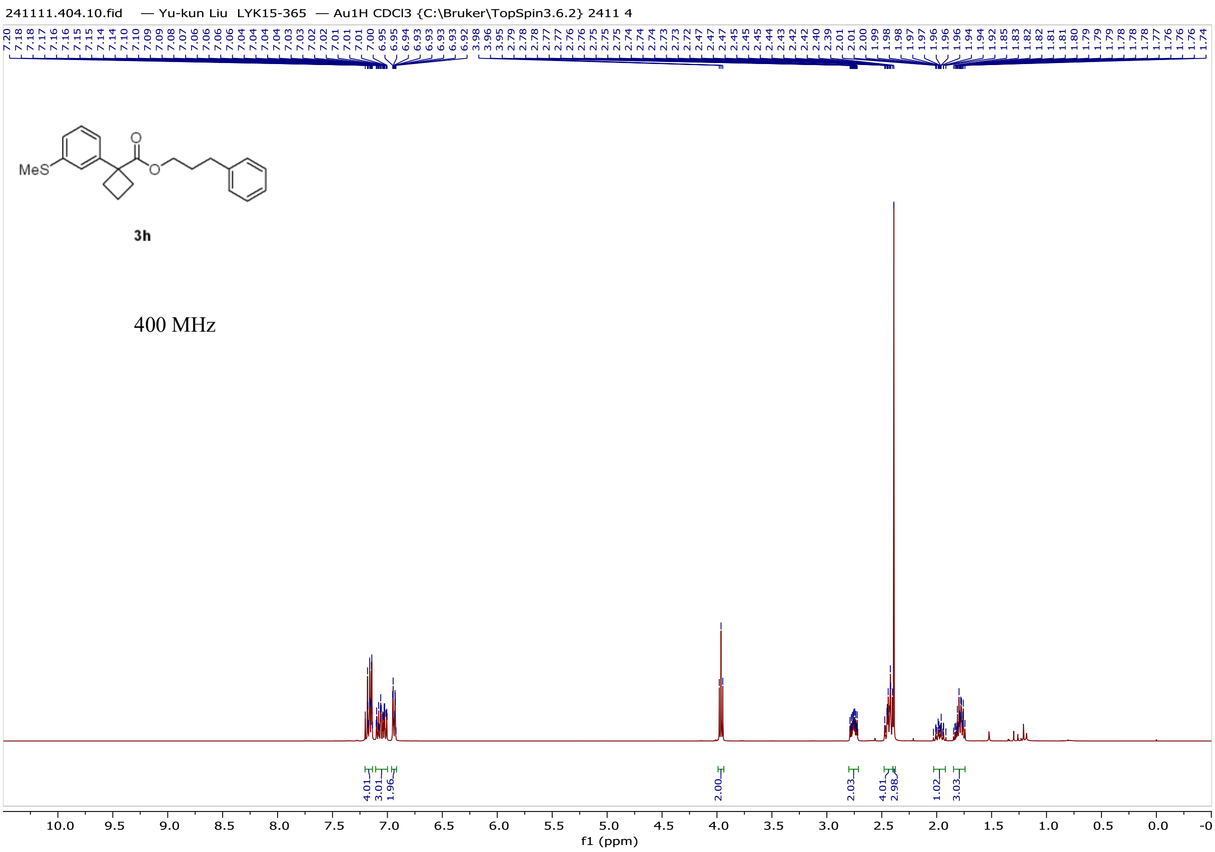
241111.404.10.fid — Yu-kun Liu LYK15-365 — Au1H CDCl3 {C:\Bruker\TopSpin3.6.2} 2411 4
MeS
O
3h
400 MHz
4.01 3.01 1.96 2.00 2.03 4.01 2.98 1.02 3.03
f1 (ppm)

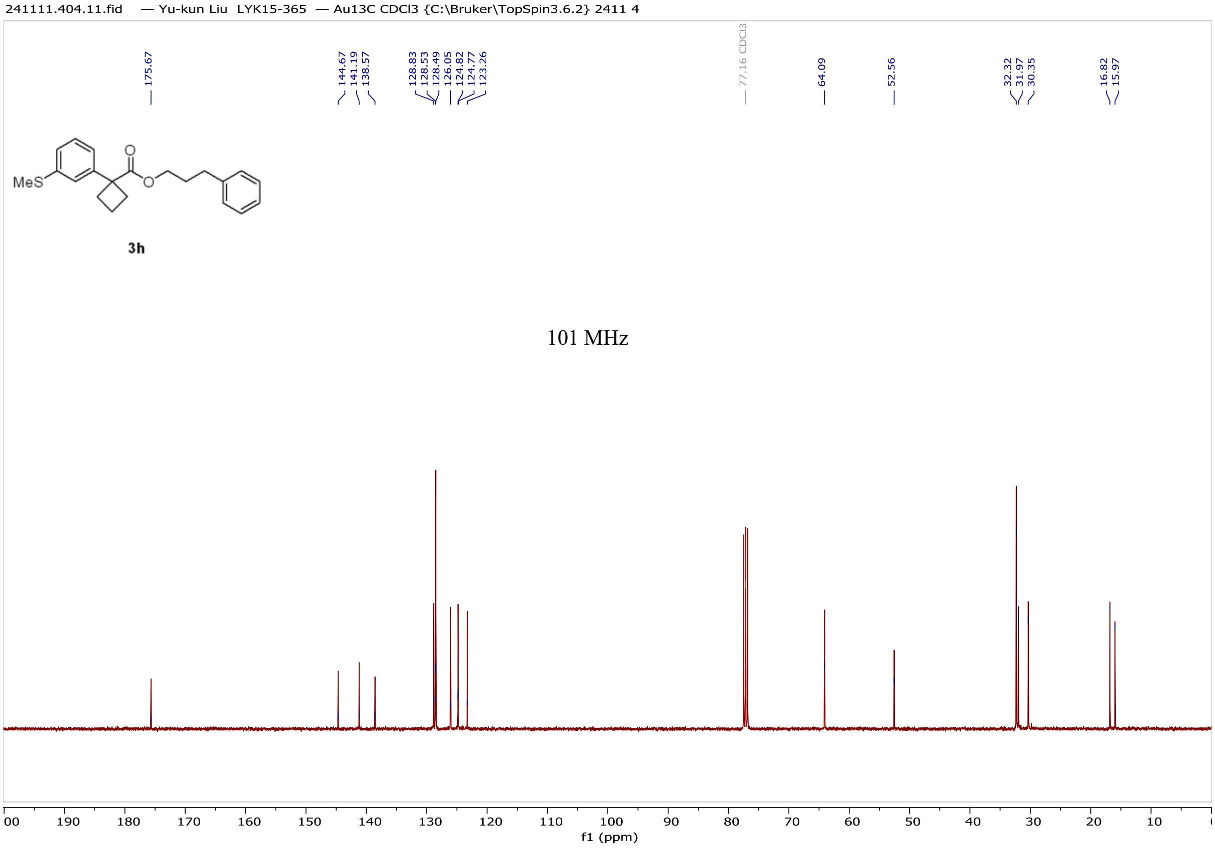
241111.404.11.fid — Yu-kun Liu LYK15-365 — Au13C CDCl3 {C:\Bruker\TopSpin3.6.2} 2411 4
175.67
144.67 141.19 138.57
128.83 128.53 128.49 126.05 124.82 124.77 123.26
77.16 CDCl3
64.09
52.56
32.32 31.97 30.35
16.82 15.97
MeS
O
3h
101 MHz
f1 (ppm)

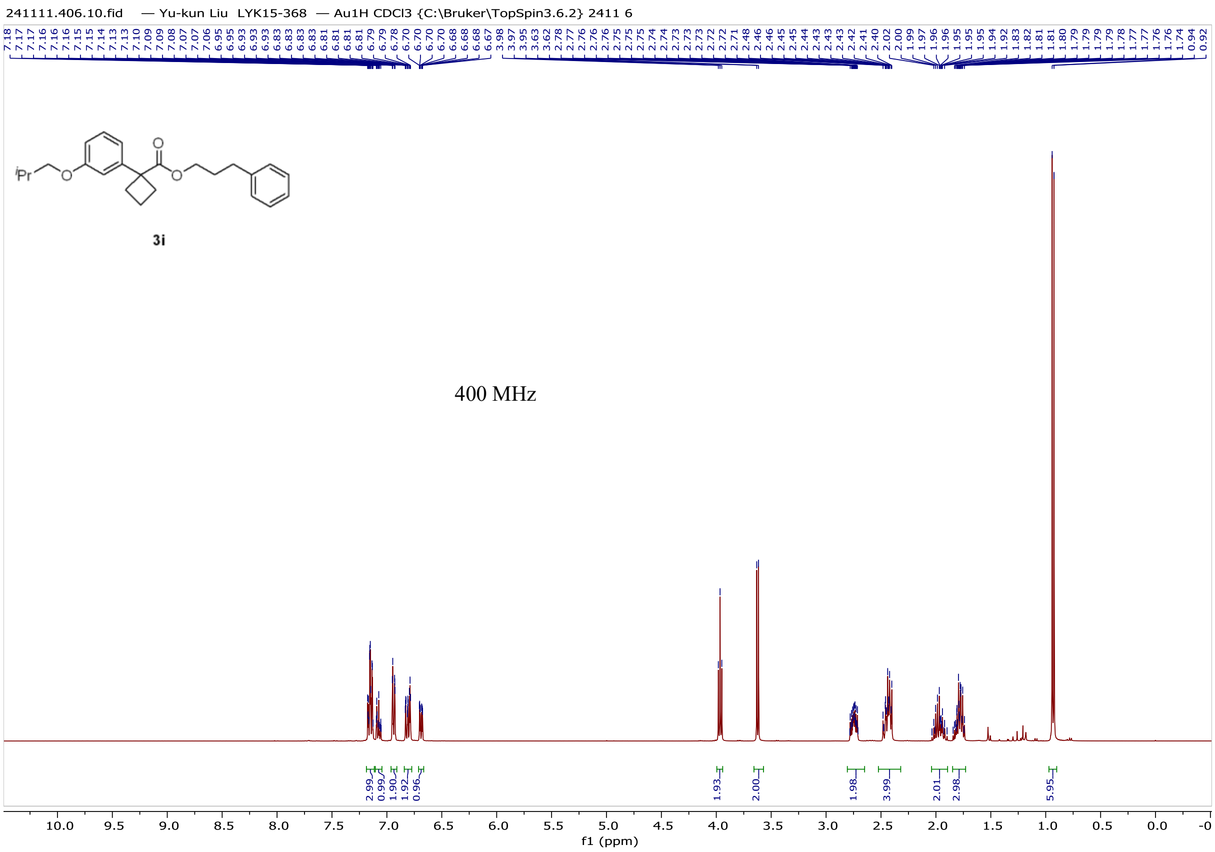
241111.406.10.fid — Yu-kun Liu LYK15-368 — Au1H CDCl3 {C:\Bruker\TopSpin3.6.2} 2411 6
7.18 7.17 7.16 7.16 7.15 7.15 7.14 7.13 7.13 7.10 7.09 7.09 7.08 7.07 7.07 7.06 6.95 6.95 6.93 6.93 6.93 6.83 6.83 6.83 6.83 6.81 6.81 6.81 6.81 6.79 6.79 6.78 6.70 6.70 6.70 6.70 6.68 6.68 6.68 6.67 3.98 3.97 3.95 3.63 3.62 2.78 2.77 2.76 2.76 2.76 2.75 2.75 2.75 2.74 2.74 2.73 2.73 2.73 2.72 2.72 2.71 2.48 2.46 2.46 2.45 2.45 2.44 2.43 2.43 2.43 2.42 2.41 2.40 2.02 2.00 1.99 1.97 1.96 1.96 1.95 1.95 1.95 1.94 1.92 1.83 1.82 1.81 1.81 1.80 1.79 1.79 1.79 1.79 1.78 1.77 1.77 1.76 1.76 1.74 0.94 0.92
iPr
O
O
O
3i
400 MHz
2.99 0.99 1.90 1.92 0.96 1.93 2.00 1.98 3.99 2.01 2.98 5.95
10.0 9.5 9.0 8.5 8.0 7.5 7.0 6.5 6.0 5.5 5.0 4.5 4.0 3.5 3.0 2.5 2.0 1.5 1.0 0.5 0.0 -0
f1 (ppm)

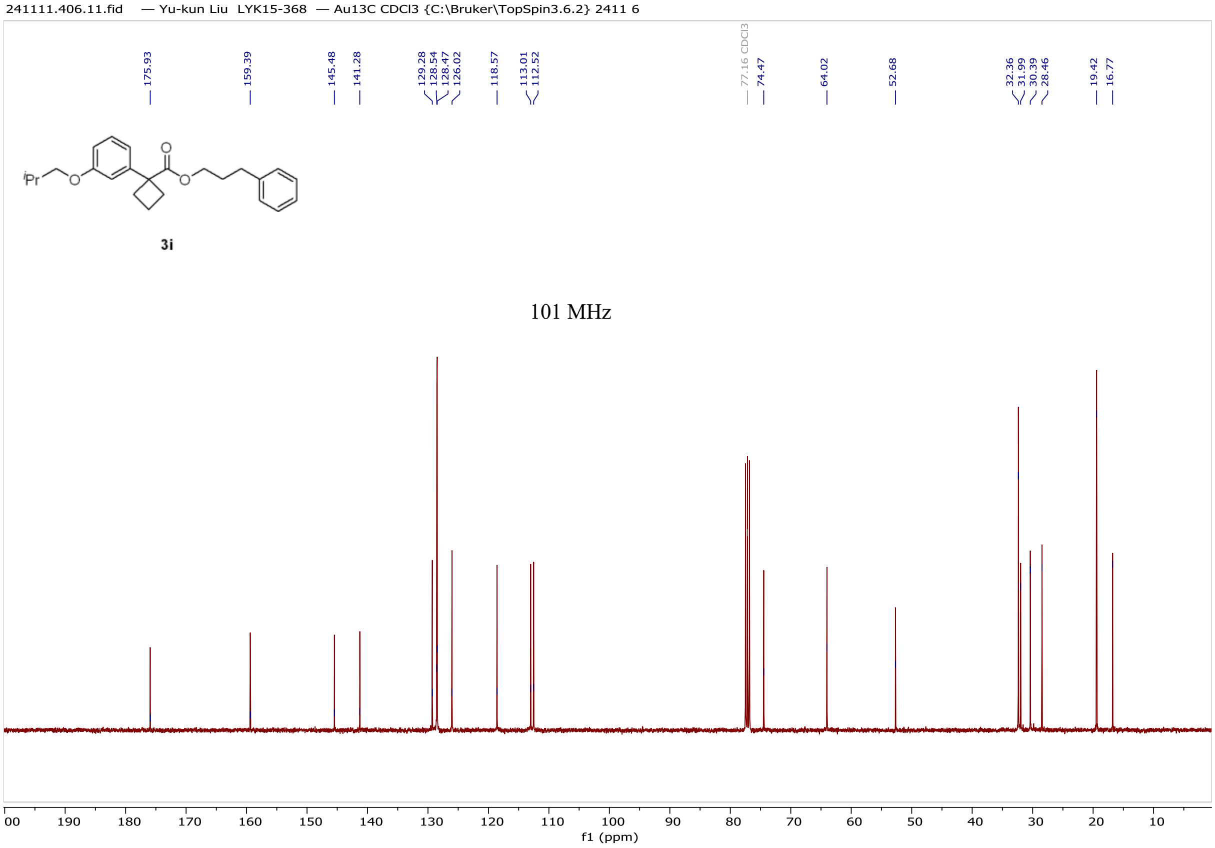
241111.406.11.fid — Yu-kun Liu LYK15-368 — Au13C CDCl3 {C:\Bruker\TopSpin3.6.2} 2411 6
175.93
159.39
145.48
141.28
129.28 128.54 128.47 126.02
118.57
113.01 112.52
77.16 CDCl3
74.47
64.02
52.68
32.36 31.99 30.39 28.46
19.42 16.77
iPr
O
O
O
3i
101 MHz
00 190 180 170 160 150 140 130 120 110 100 90 80 70 60 50 40 30 20 10
f1 (ppm)

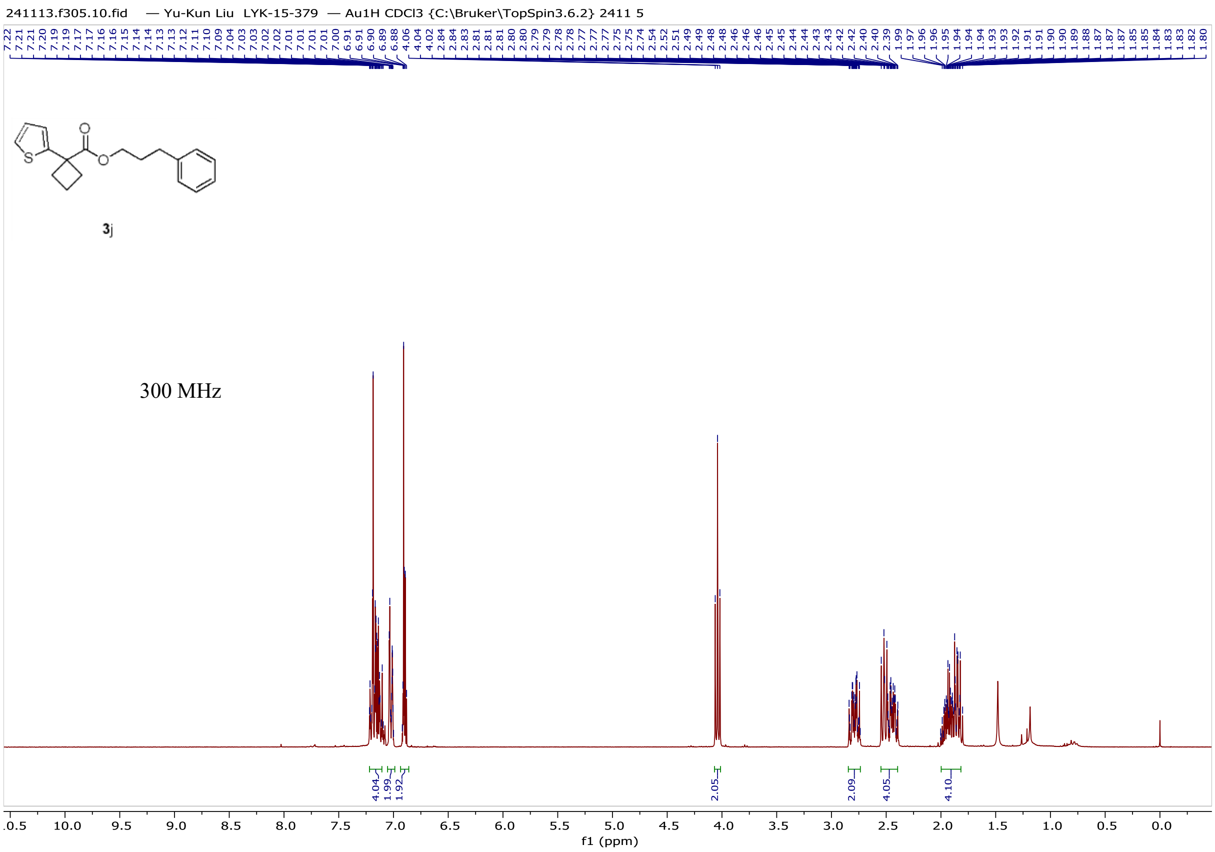
241113.f305.10.fid — Yu-Kun Liu LYK-15-379 — Au1H CDCl3 {C:\Bruker\TopSpin3.6.2} 2411 5
3j
300 MHz
4.04
1.99
1.92
2.05
2.09
4.05
4.10
f1 (ppm)

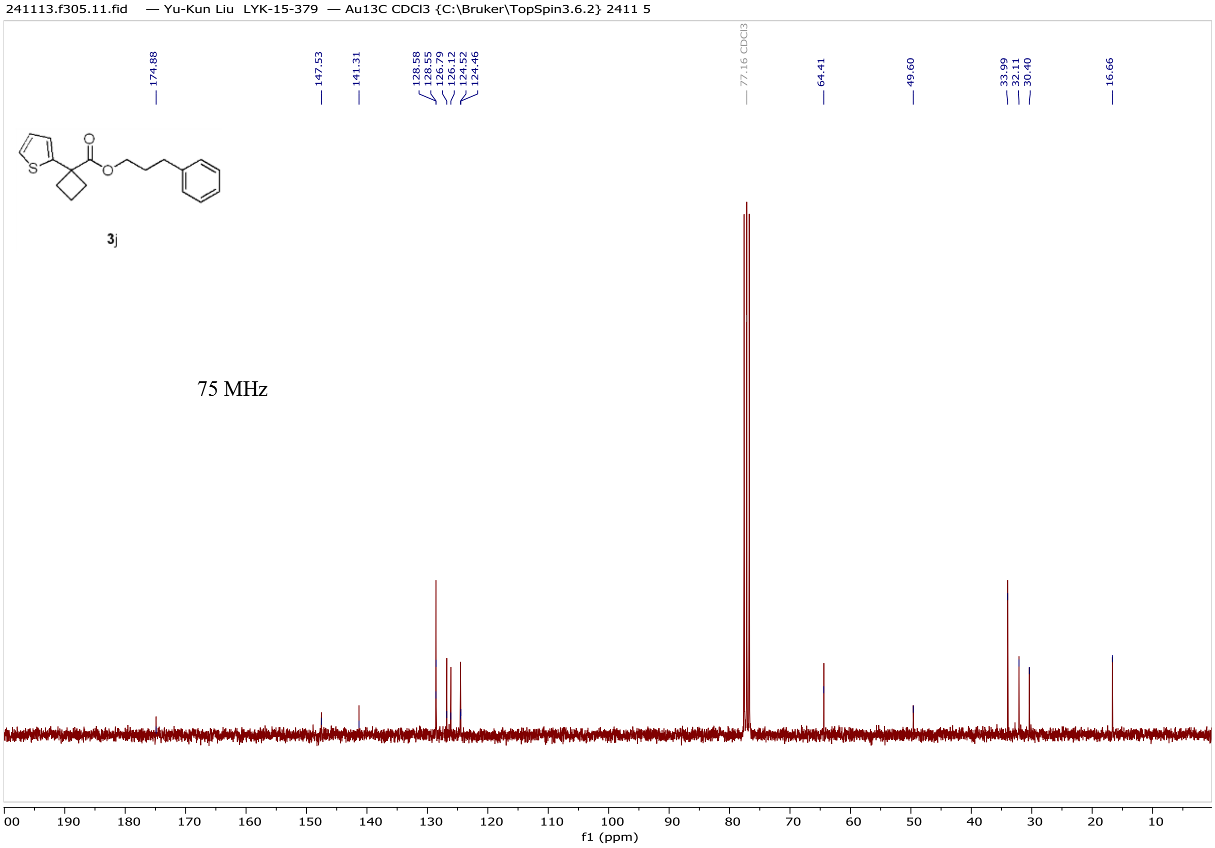
241113.f305.11.fid — Yu-Kun Liu LYK-15-379 — Au13C CDCl3 {C:\Bruker\TopSpin3.6.2} 2411 5
174.88
147.53
141.31
128.58
128.55
126.79
126.12
124.52
124.46
77.16 CDCl3
64.41
49.60
33.99
32.11
30.40
16.66
3j
75 MHz
f1 (ppm)

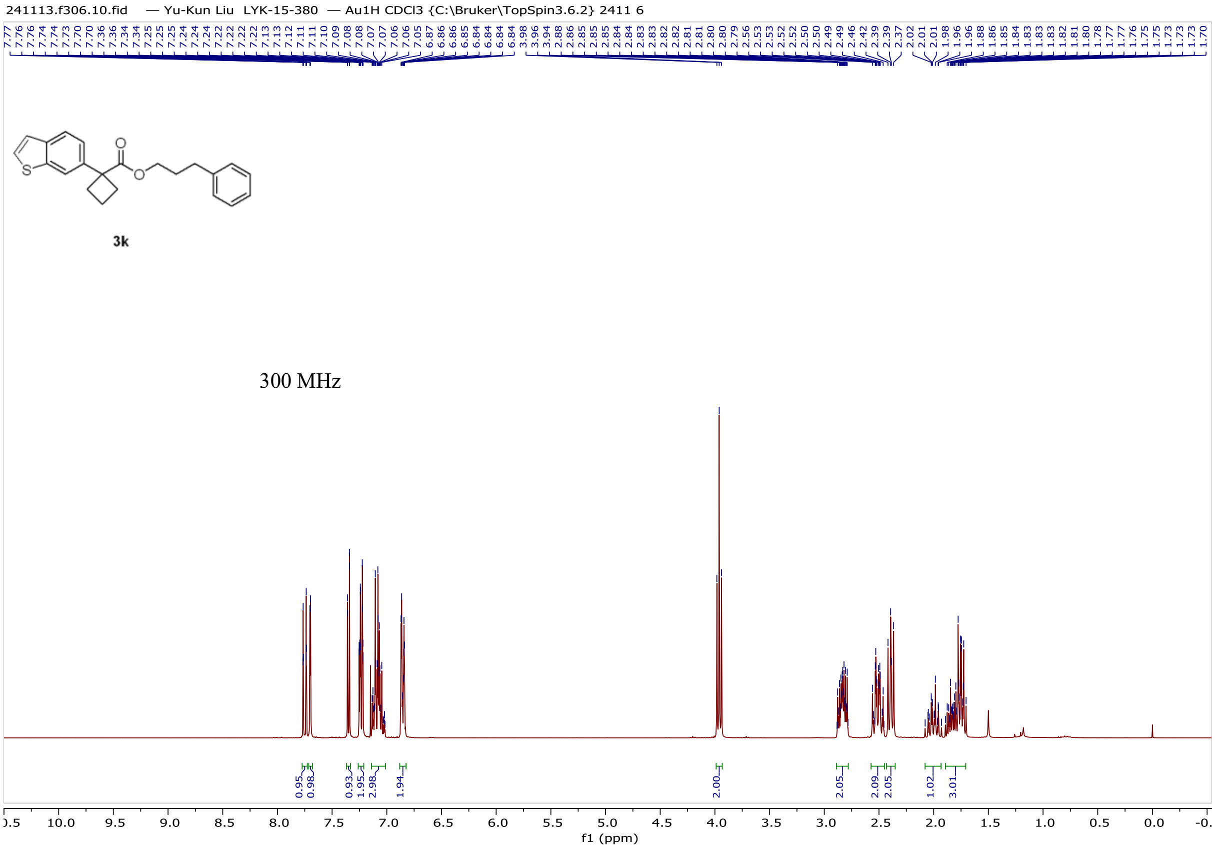
241113.f306.10.fid — Yu-Kun Liu LYK-15-380 — Au1H CDCl3 {C:\Bruker\TopSpin3.6.2} 2411 6
3k
300 MHz
f1 (ppm)

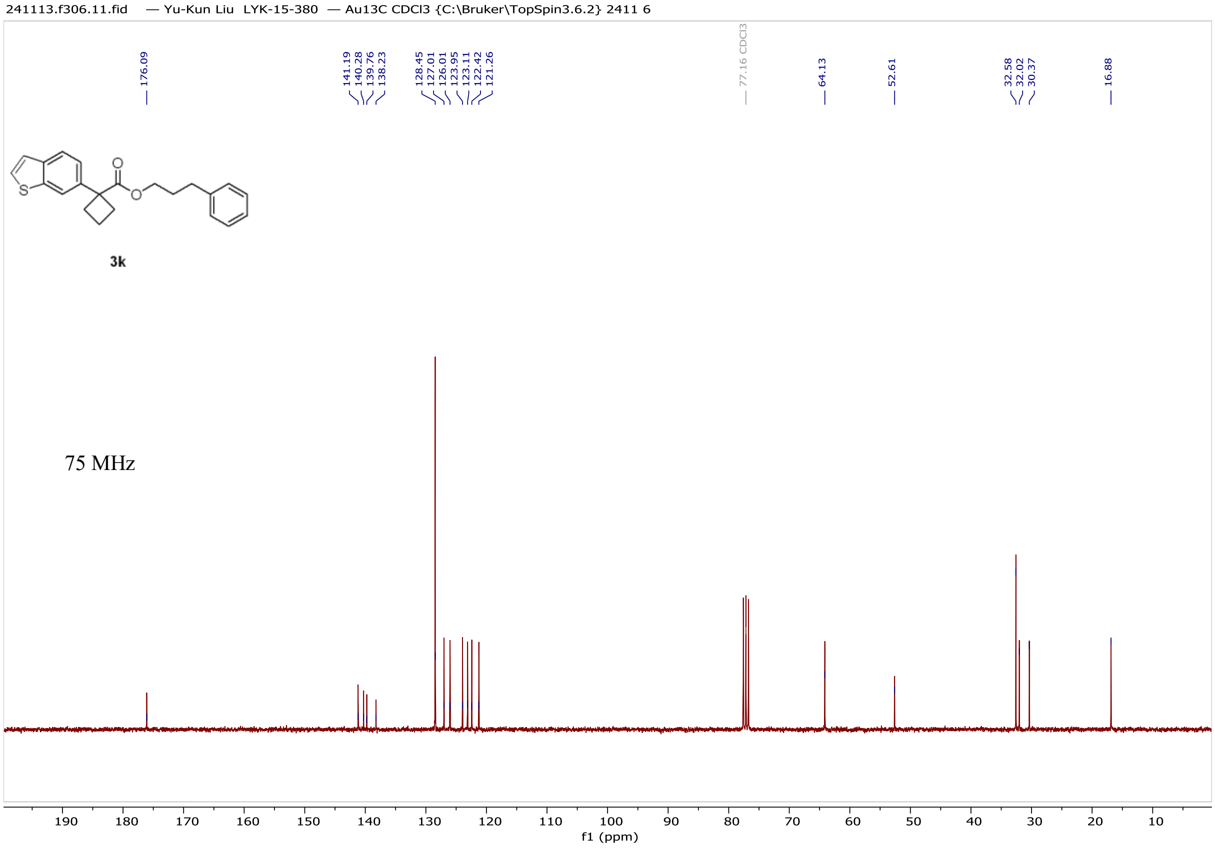
241113.f306.11.fid — Yu-Kun Liu LYK-15-380 — Au13C CDCl3 {C:\Bruker\TopSpin3.6.2} 2411 6
176.09
141.19
140.28
139.76
138.23
128.45
127.01
126.01
123.95
123.11
122.42
121.26
77.16 CDCl3
64.13
52.61
32.58
32.02
30.37
16.88
3k
75 MHz
f1 (ppm)

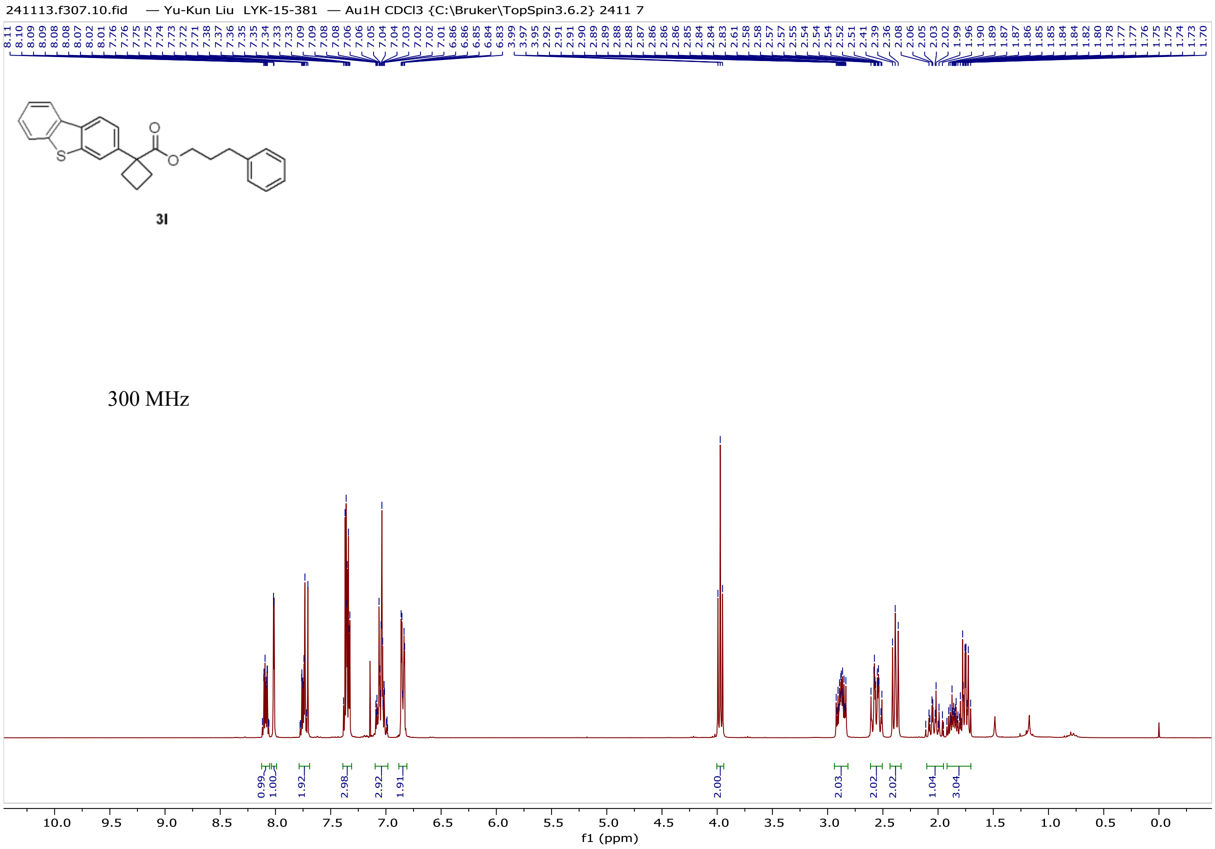
241113.f307.10.fid — Yu-Kun Liu LYK-15-381 — Au1H CDCl3 {C:\Bruker\TopSpin3.6.2} 2411 7
8.11 8.10 8.09 8.08 8.08 8.07 8.02 8.01 7.76 7.76 7.75 7.75 7.74 7.73 7.72 7.71 7.38 7.37 7.36 7.35 7.34 7.33 7.33 7.09 7.09 7.08 7.08 7.06 7.06 7.05 7.04 7.04 7.03 7.02 7.01 6.86 6.86 6.85 6.84 6.83 3.99 3.97 3.95 2.92 2.91 2.91 2.90 2.89 2.89 2.88 2.88 2.87 2.86 2.86 2.85 2.85 2.84 2.84 2.83 2.61 2.58 2.58 2.57 2.57 2.55 2.54 2.54 2.54 2.52 2.51 2.41 2.39 2.36 2.08 2.06 2.05 2.03 2.02 1.99 1.96 1.90 1.89 1.87 1.87 1.86 1.85 1.85 1.84 1.84 1.82 1.80 1.78 1.77 1.76 1.75 1.74 1.73 1.70
S
O
O
3l
300 MHz
0.99 1.00 1.92 2.98 2.92 1.91 2.00 2.03 2.02 2.02 1.04 3.04
10.0 9.5 9.0 8.5 8.0 7.5 7.0 6.5 6.0 5.5 5.0 4.5 4.0 3.5 3.0 2.5 2.0 1.5 1.0 0.5 0.0
f1 (ppm)

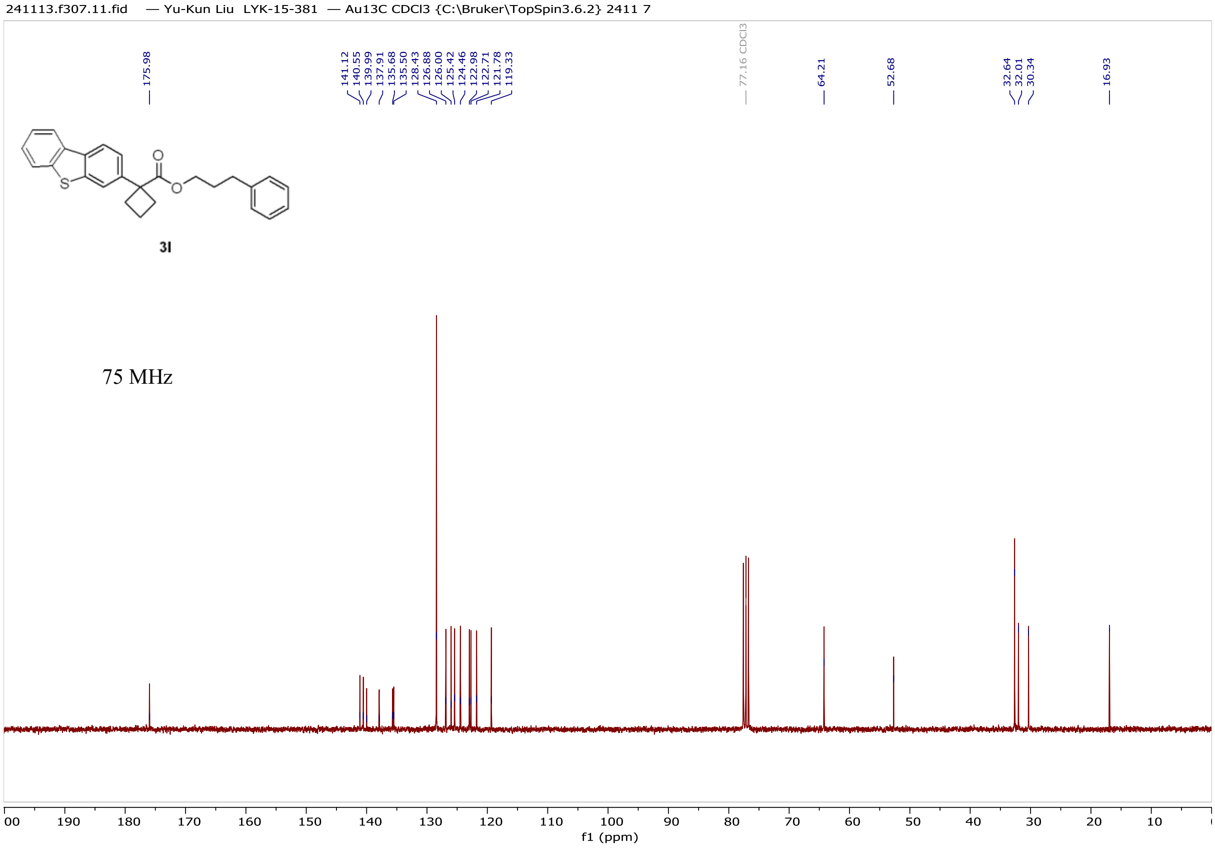
241113.f307.11.fid — Yu-Kun Liu LYK-15-381 — Au13C CDCl3 {C:\Bruker\TopSpin3.6.2} 2411 7
175.98
141.12 140.55 139.99 137.91 135.68 135.50 128.43 126.88 126.00 125.42 124.46 122.98 122.71 121.78 119.33
77.16 CDCl3
64.21
52.68
32.64 32.01 30.34
16.93
S
O
O
3l
75 MHz
00 190 180 170 160 150 140 130 120 110 100 90 80 70 60 50 40 30 20 10
f1 (ppm)

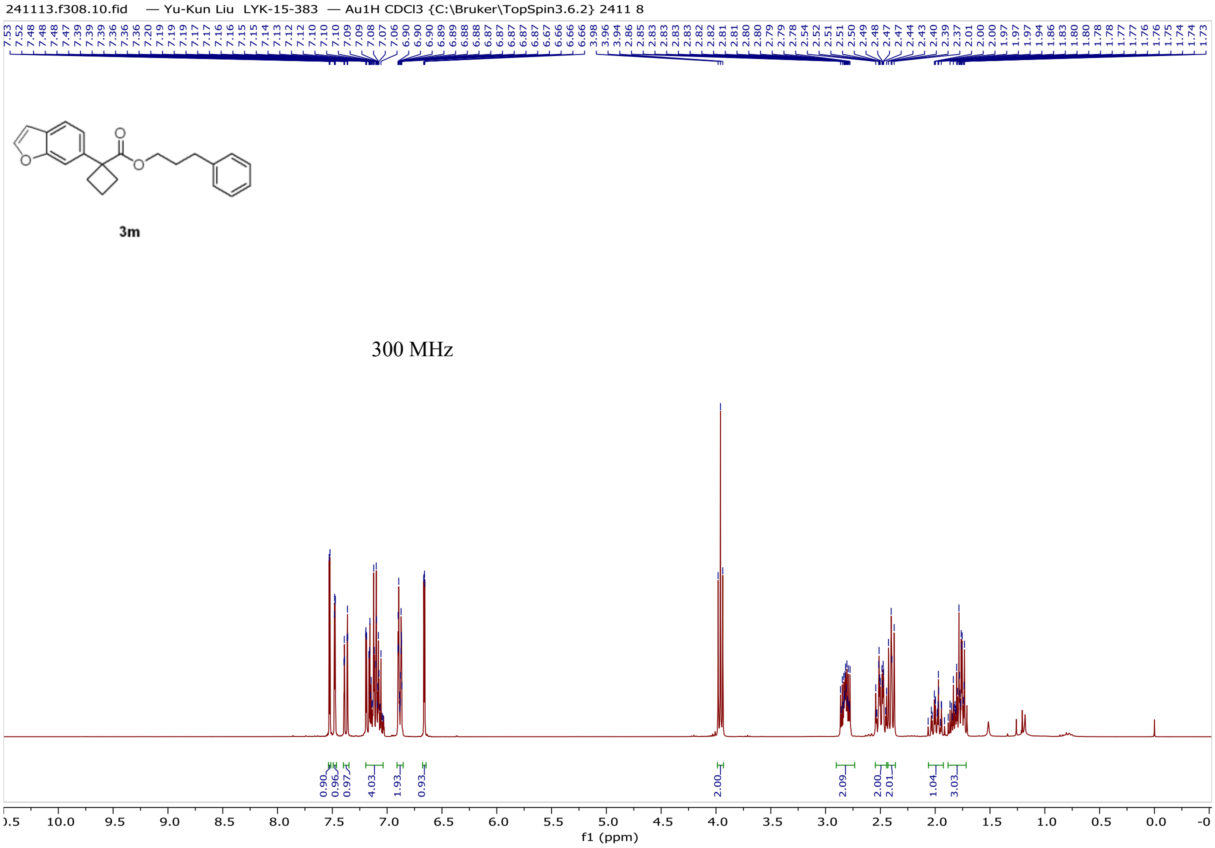
241113.f308.10.fid — Yu-Kun Liu LYK-15-383 — Au1H CDCl3 {C:\Bruker\TopSpin3.6.2} 2411 8
3m
300 MHz
f1 (ppm)

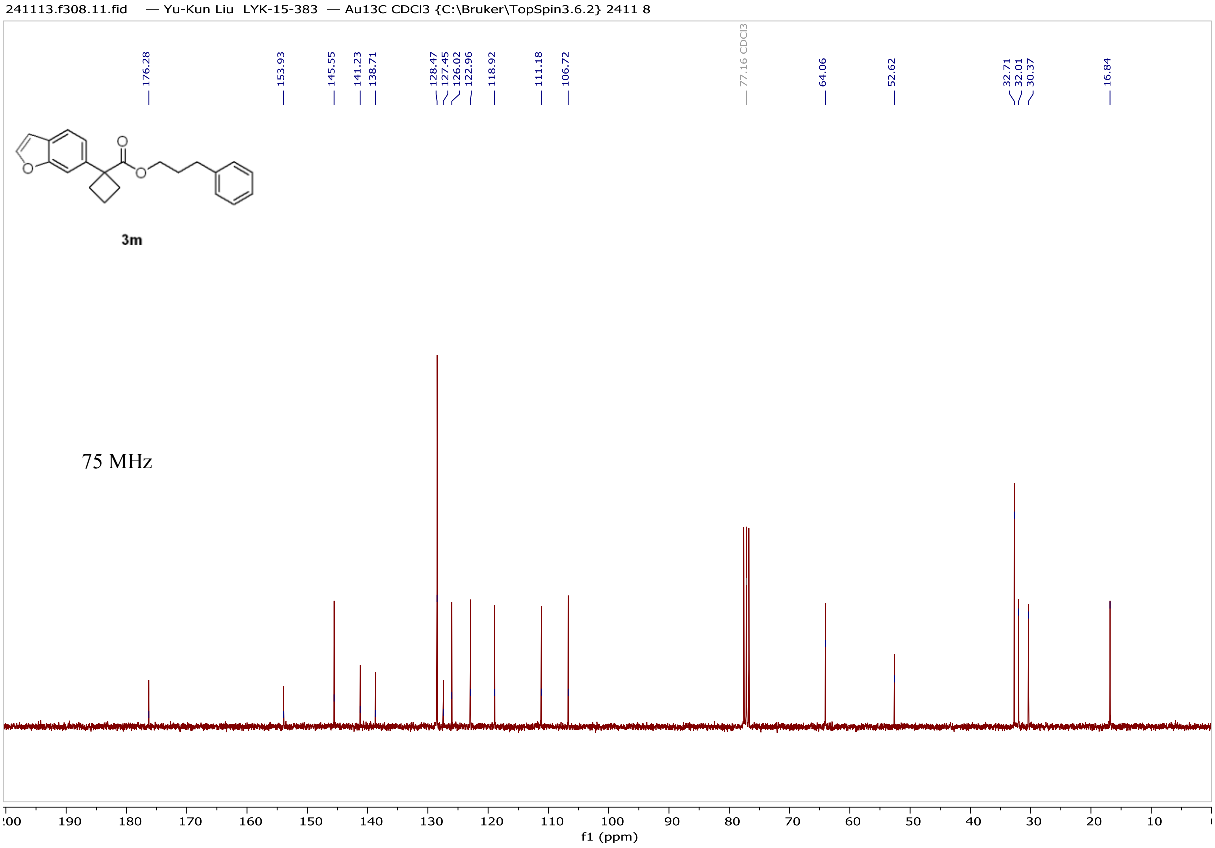
241113.f308.11.fid — Yu-Kun Liu LYK-15-383 — Au13C CDCl3 {C:\Bruker\TopSpin3.6.2} 2411 8
176.28
153.93
145.55
141.23
138.71
128.47
127.45
126.02
122.96
118.92
111.18
106.72
77.16 CDCl3
64.06
52.62
32.71
32.01
30.37
16.84
3m
75 MHz
f1 (ppm)

241113.f310.10.fid — Yu-Kun Liu LYK-15-384 — Au1H CDCl3 {C:\Bruker\TopSpin3.6.2} 2411 10

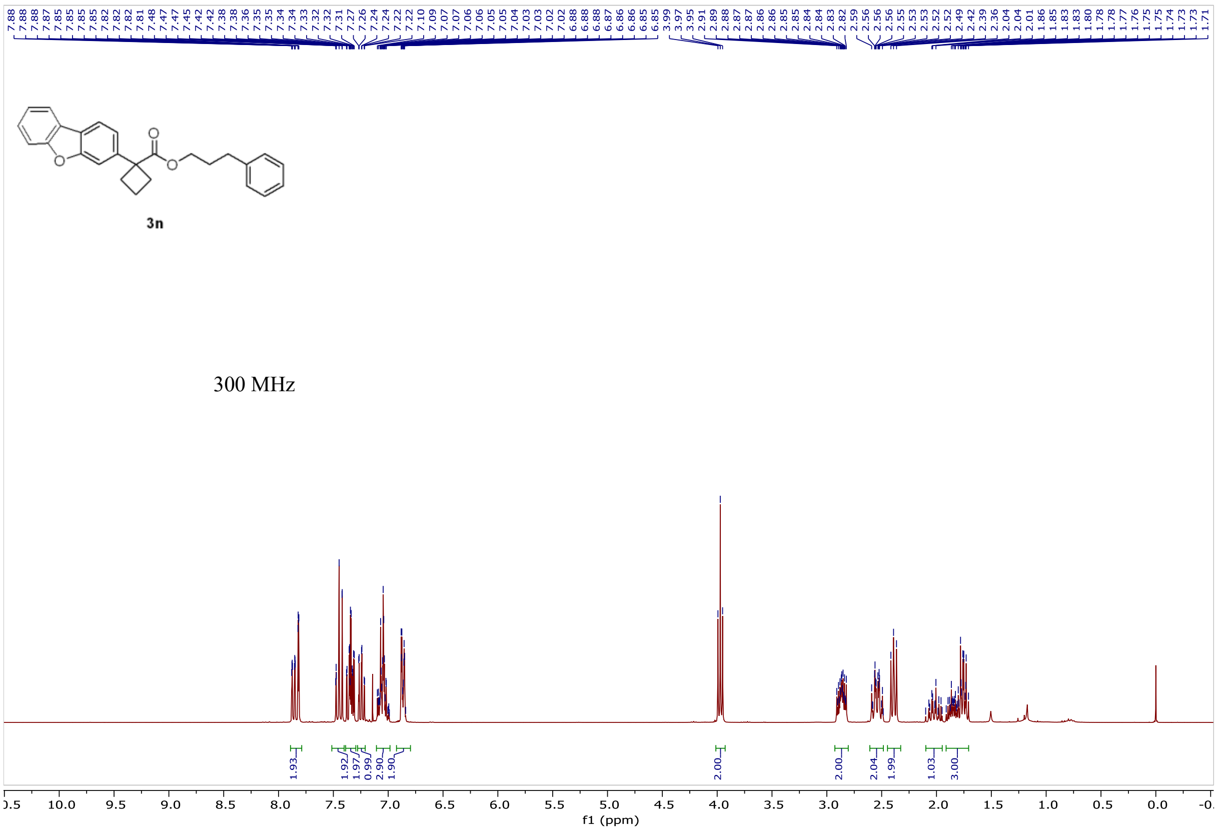

241113.f310.11.fid — Yu-Kun Liu LYK-15-384 — Au13C CDCl3 {C:\Bruker\TopSpin3.6.2} 2411 10

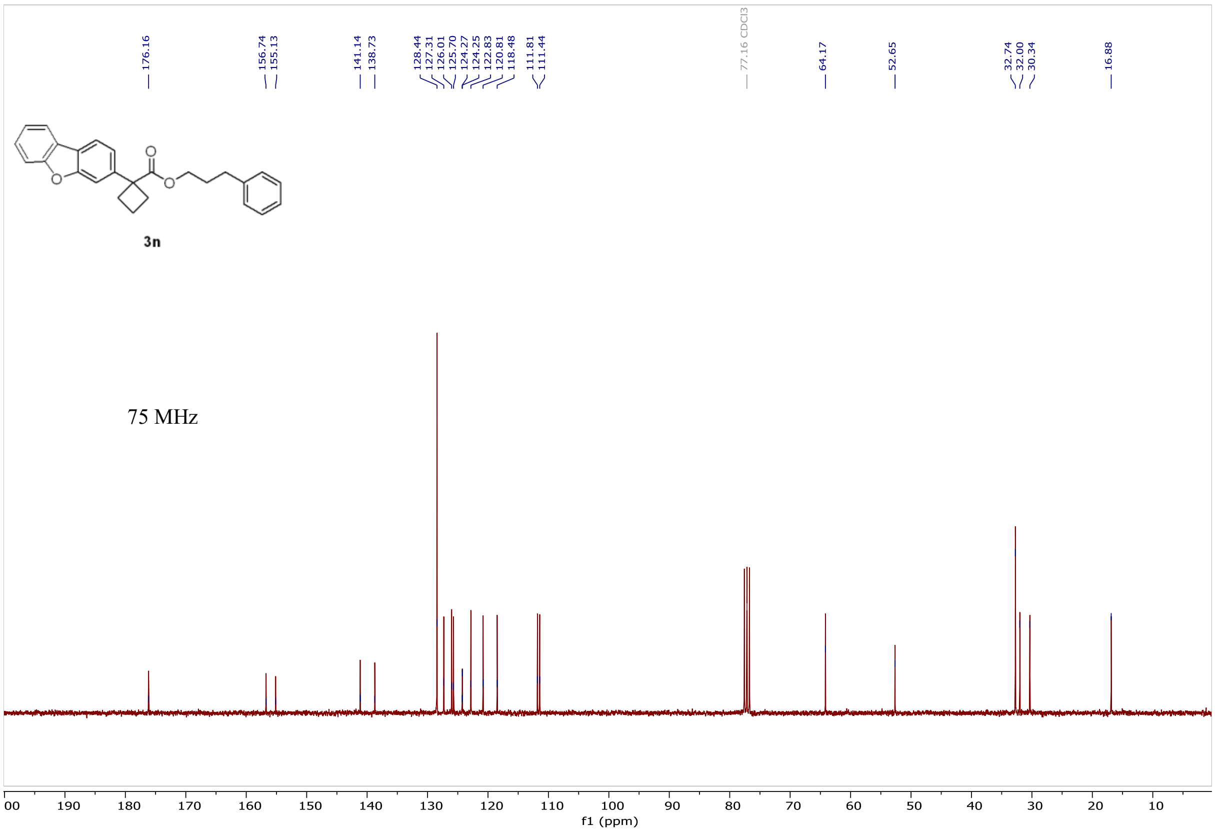

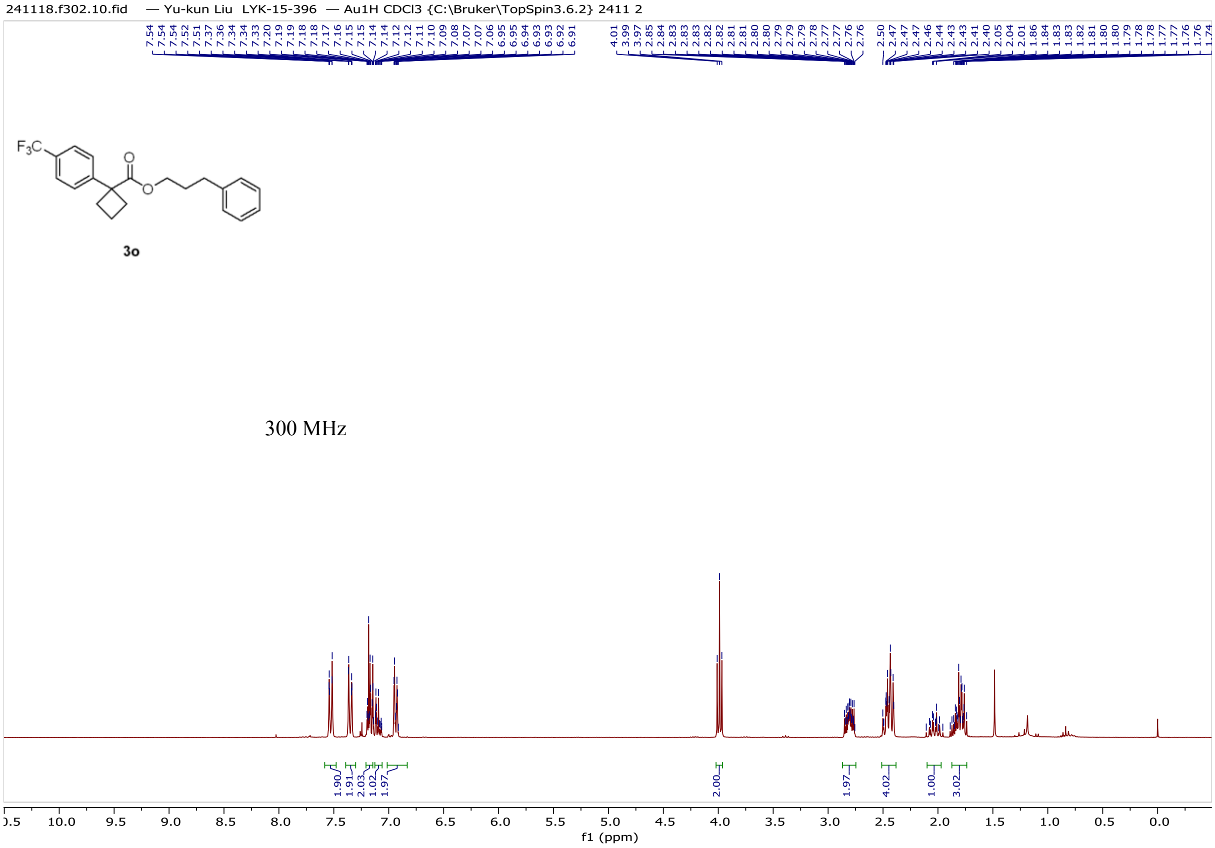
241118.f302.10.fid — Yu-kun Liu LYK-15-396 — Au1H CDCl3 {C:\Bruker\TopSpin3.6.2} 2411 2
F_3C
3o
300 MHz
1.90
1.91
2.03
1.02
1.97
2.00
1.97
4.02
1.00
3.02
f1 (ppm)

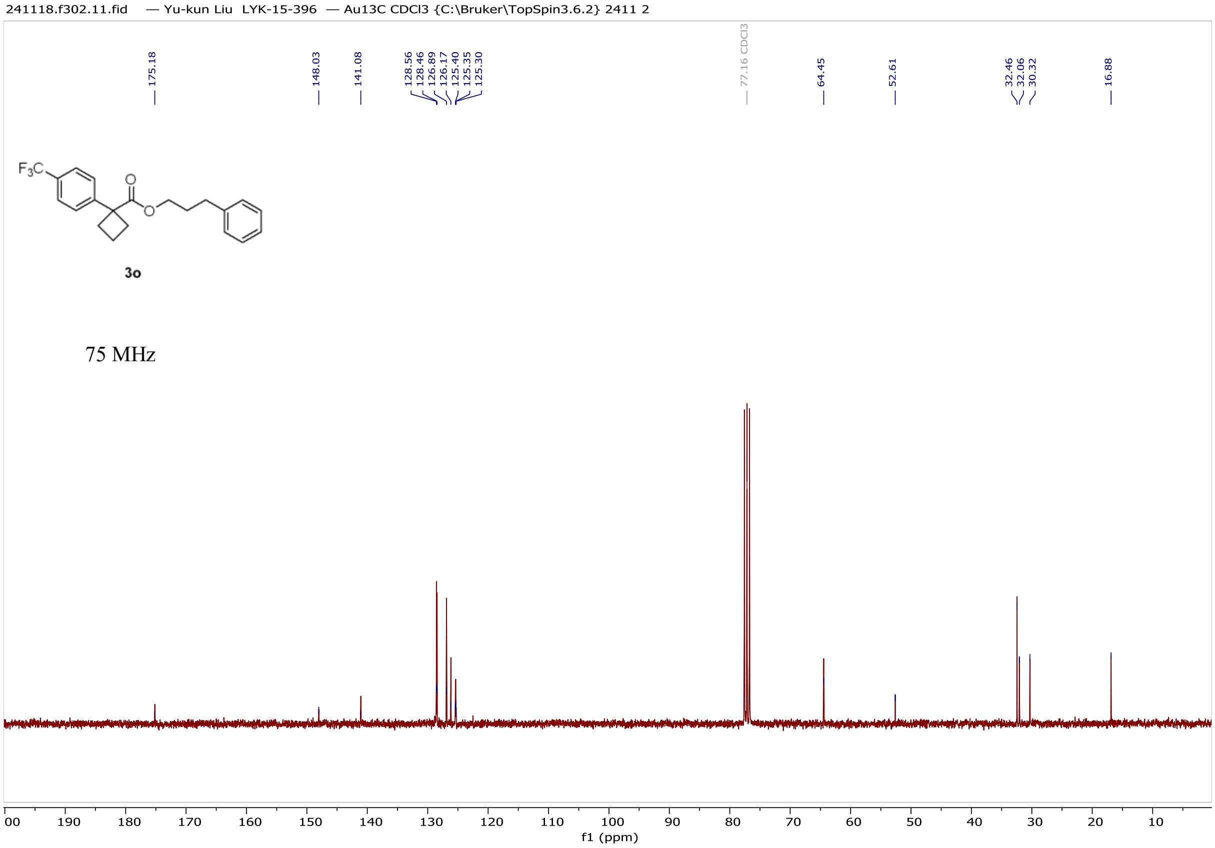
241118.f302.11.fid — Yu-kun Liu LYK-15-396 — Au13C CDCl3 {C:\Bruker\TopSpin3.6.2} 2411 2
175.18
148.03
141.08
128.56
128.46
126.89
126.17
125.40
125.35
125.30
77.16 CDCl3
64.45
52.61
32.46
32.06
30.32
16.88
F_3C
3o
75 MHz
f1 (ppm)

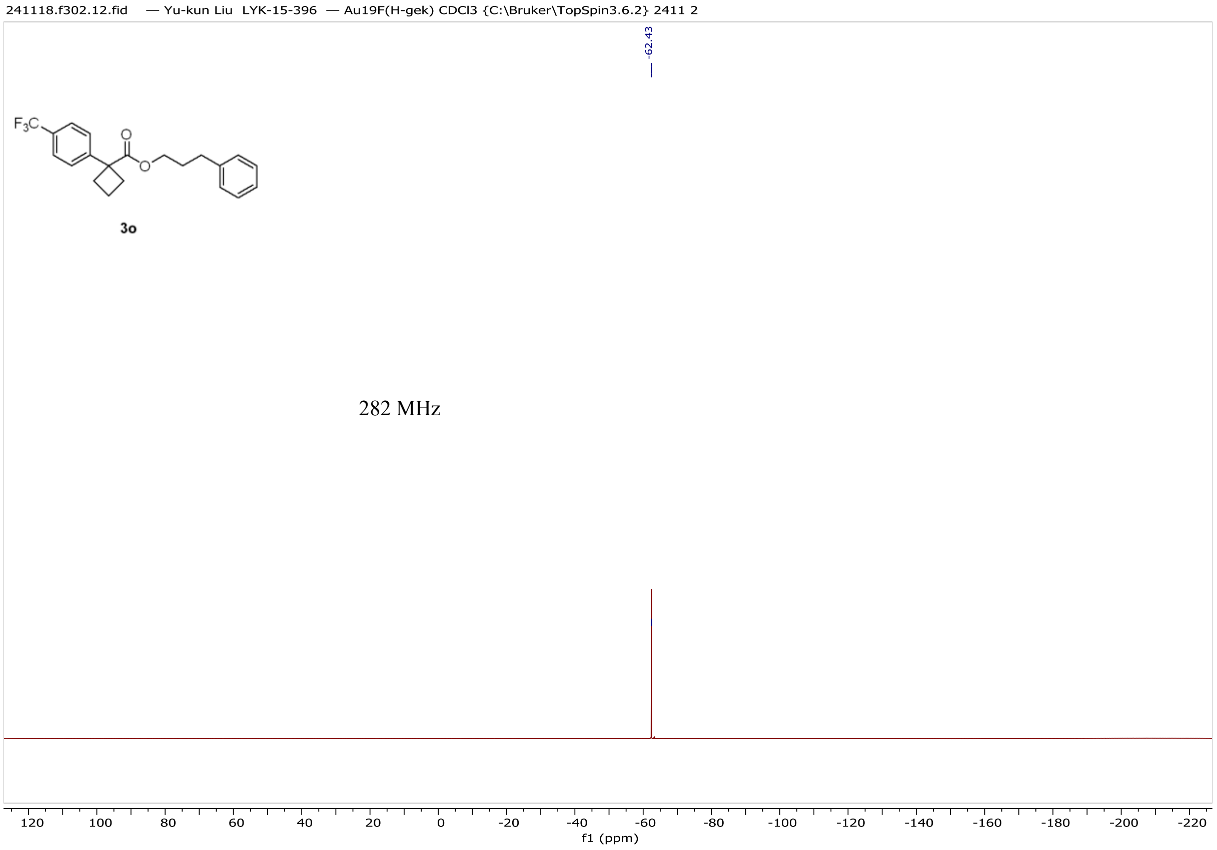

241118.f302.12.fid — Yu-kun Liu LYK-15-396 — Au19F(H-gek) CDCl3 {C:\Bruker\TopSpin3.6.2} 2411 2
-62.43
F3C
O
O
3o
282 MHz
120 100 80 60 40 20 0 -20 -40 -60 -80 -100 -120 -140 -160 -180 -200 -220
f1 (ppm)

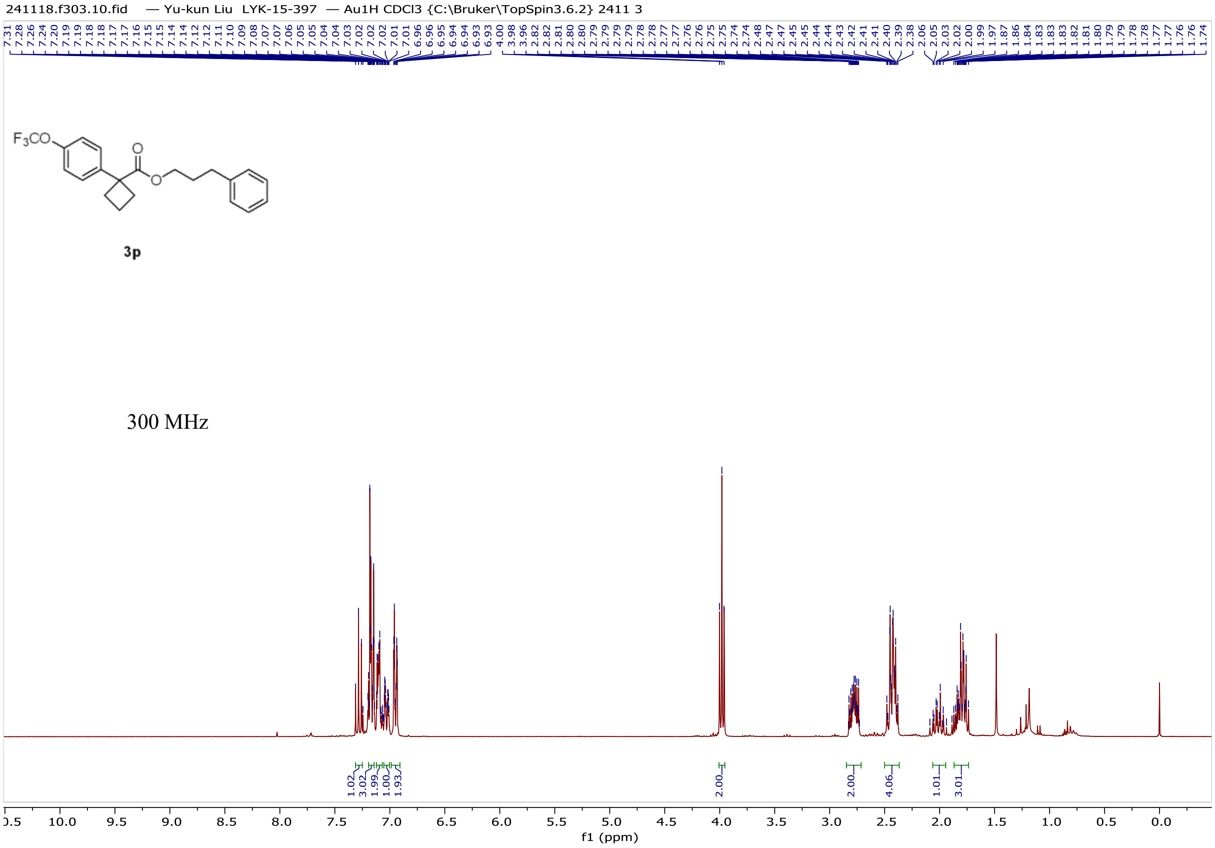
241118.f303.10.fid — Yu-kun Liu LYK-15-397 — Au1H CDCl3 {C:\Bruker\TopSpin3.6.2} 2411 3
7.31 7.28 7.26 7.24 7.20 7.19 7.19 7.18 7.17 7.17 7.16 7.15 7.15 7.14 7.14 7.12 7.12 7.11 7.10 7.09 7.08 7.07 7.07 7.06 7.05 7.05 7.05 7.04 7.04 7.03 7.02 7.02 7.02 7.01 7.01 6.96 6.96 6.95 6.94 6.94 6.93 6.93 4.00 3.98 3.96 2.82 2.82 2.81 2.80 2.80 2.79 2.79 2.79 2.78 2.78 2.77 2.77 2.76 2.76 2.75 2.75 2.74 2.74 2.48 2.47 2.47 2.45 2.45 2.44 2.44 2.43 2.42 2.41 2.41 2.40 2.39 2.38 2.06 2.05 2.03 2.02 2.00 1.99 1.97 1.87 1.86 1.84 1.84 1.83 1.83 1.82 1.81 1.80 1.79 1.79 1.78 1.78 1.77 1.77 1.76 1.76 1.74
F_3CO
O
O
3p
300 MHz
1.02 3.02 1.99 1.00 1.93 2.00 2.00 4.06 1.01 3.01
f1 (ppm)

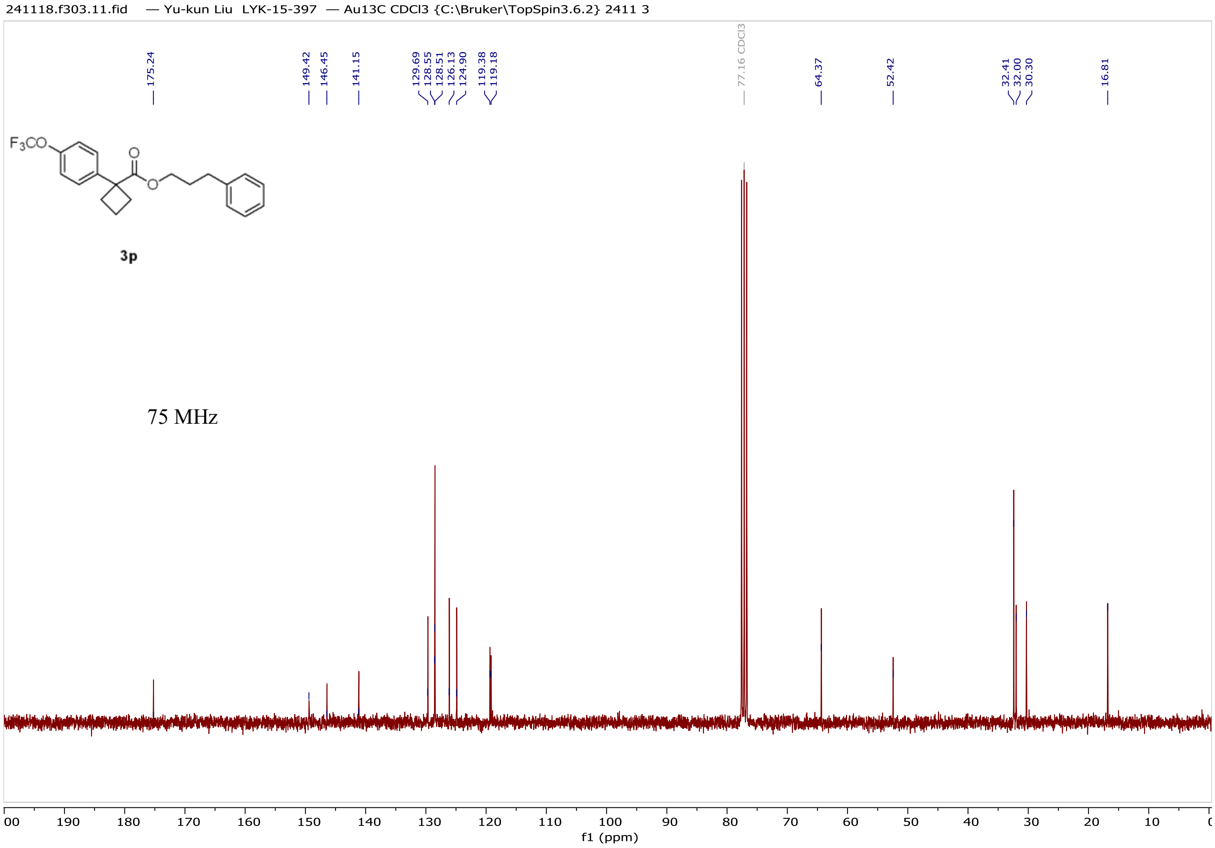
241118.f303.11.fid — Yu-kun Liu LYK-15-397 — Au13C CDCl3 {C:\Bruker\TopSpin3.6.2} 2411 3
175.24 149.42 146.45 141.15 129.69 128.55 128.51 126.13 124.90 119.38 119.18 77.16 CDCl3 64.37 52.42 32.41 32.00 30.30 16.81
F_3CO
O
O
3p
75 MHz
f1 (ppm)

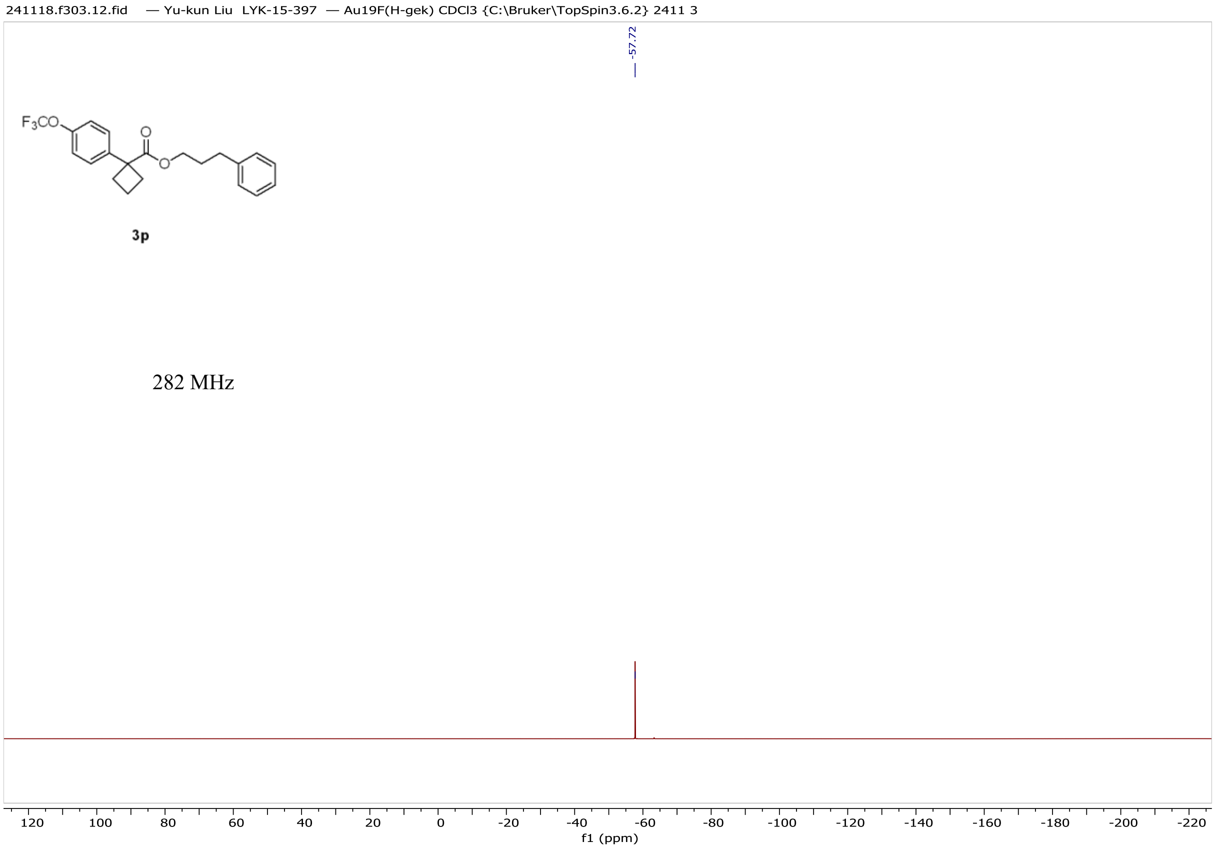
241118.f303.12.fid — Yu-kun Liu LYK-15-397 — Au19F(H-gek) CDCl3 {C:\Bruker\TopSpin3.6.2} 2411 3
-57.72
F3CO
3p
282 MHz
120 100 80 60 40 20 0 -20 -40 -60 -80 -100 -120 -140 -160 -180 -200 -220
f1 (ppm)

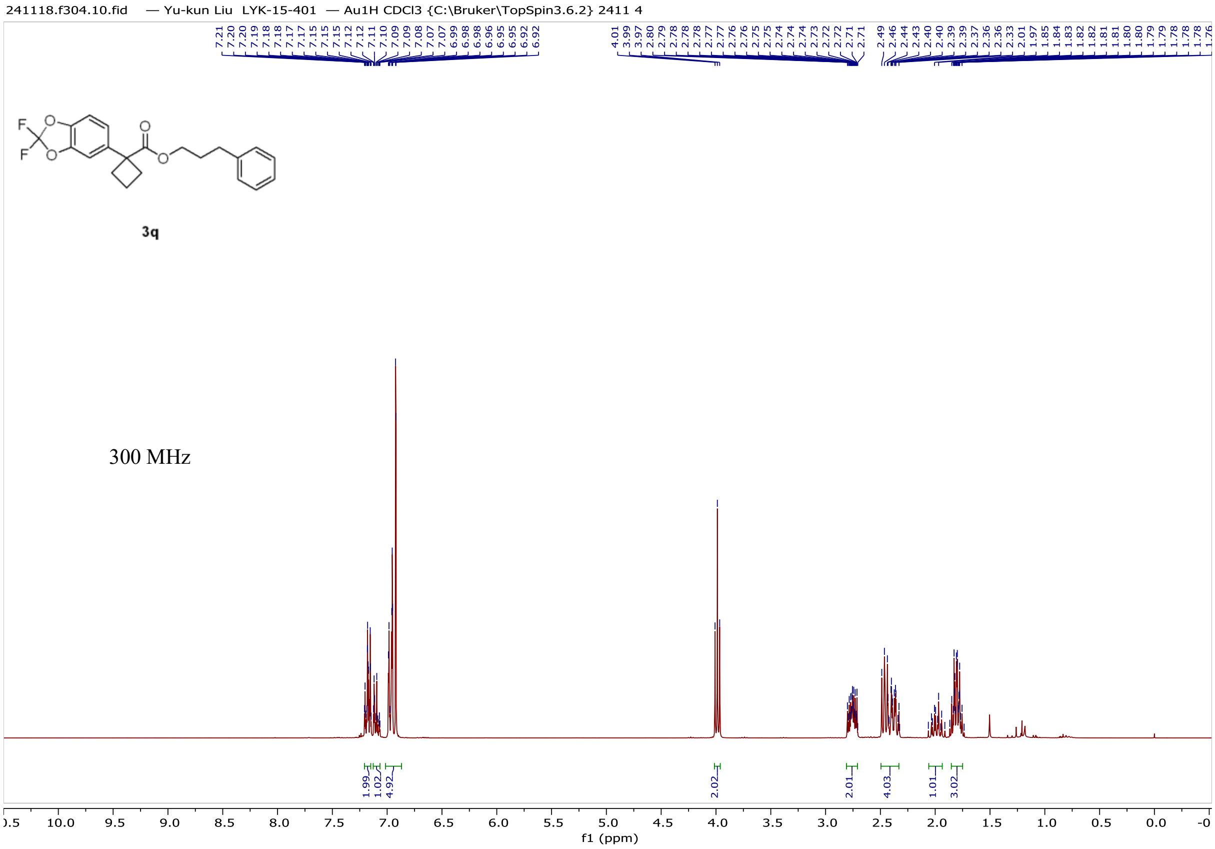
241118.f304.10.fid — Yu-kun Liu LYK-15-401 — Au1H CDCl3 {C:\Bruker\TopSpin3.6.2} 2411 4
7.21 7.20 7.19 7.18 7.18 7.17 7.17 7.15 7.15 7.15 7.12 7.11 7.10 7.09 7.09 7.08 7.07 7.07 6.99 6.98 6.98 6.96 6.95 6.92 6.92 6.92
4.01 3.99 3.97 2.80 2.79 2.78 2.78 2.78 2.77 2.77 2.76 2.76 2.75 2.75 2.74 2.74 2.74 2.73 2.73 2.72 2.72 2.71 2.71 2.71
2.49 2.46 2.44 2.43 2.40 2.40 2.39 2.39 2.37 2.36 2.36 2.33 2.01 1.97 1.85 1.84 1.83 1.82 1.82 1.81 1.81 1.80 1.80 1.79 1.79 1.78 1.78 1.76
F
F
O
O
O
O
3q
300 MHz
1.99
1.02
4.92
2.02
2.01
4.03
1.01
3.02
f1 (ppm)

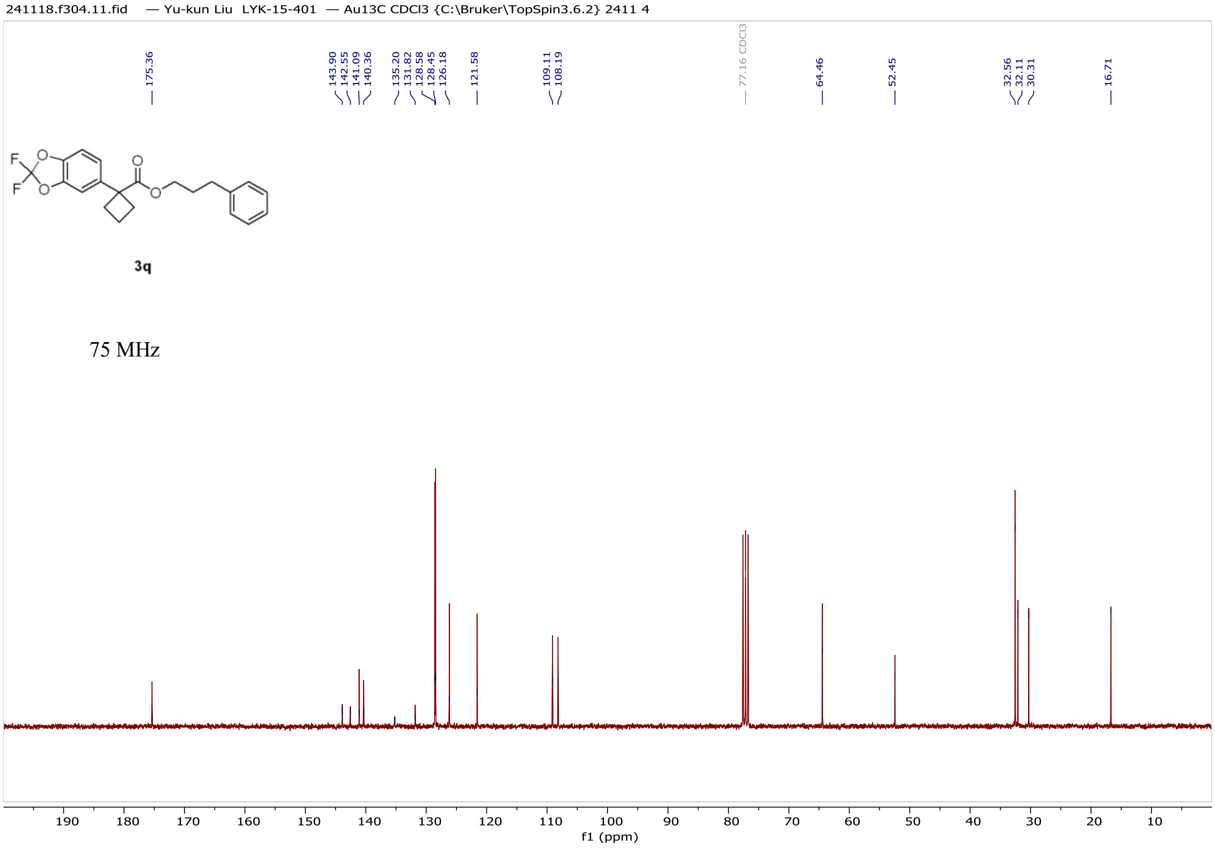
241118.f304.11.fid — Yu-kun Liu LYK-15-401 — Au13C CDCl3 {C:\Bruker\TopSpin3.6.2} 2411 4
175.36
143.90 142.55 141.09 140.36 135.20 131.82 128.58 128.45 126.18 121.58 109.11 108.19
77.16 CDCl3
64.46
52.45
32.56 32.11 30.31
16.71
F
F
O
O
O
O
3q
75 MHz
f1 (ppm)

241118.f304.12.fid — Yu-kun Liu LYK-15-401 — Au19F(H-gek) CDCl3 {C:\Bruker\TopSpin3.6.2} 2411 4

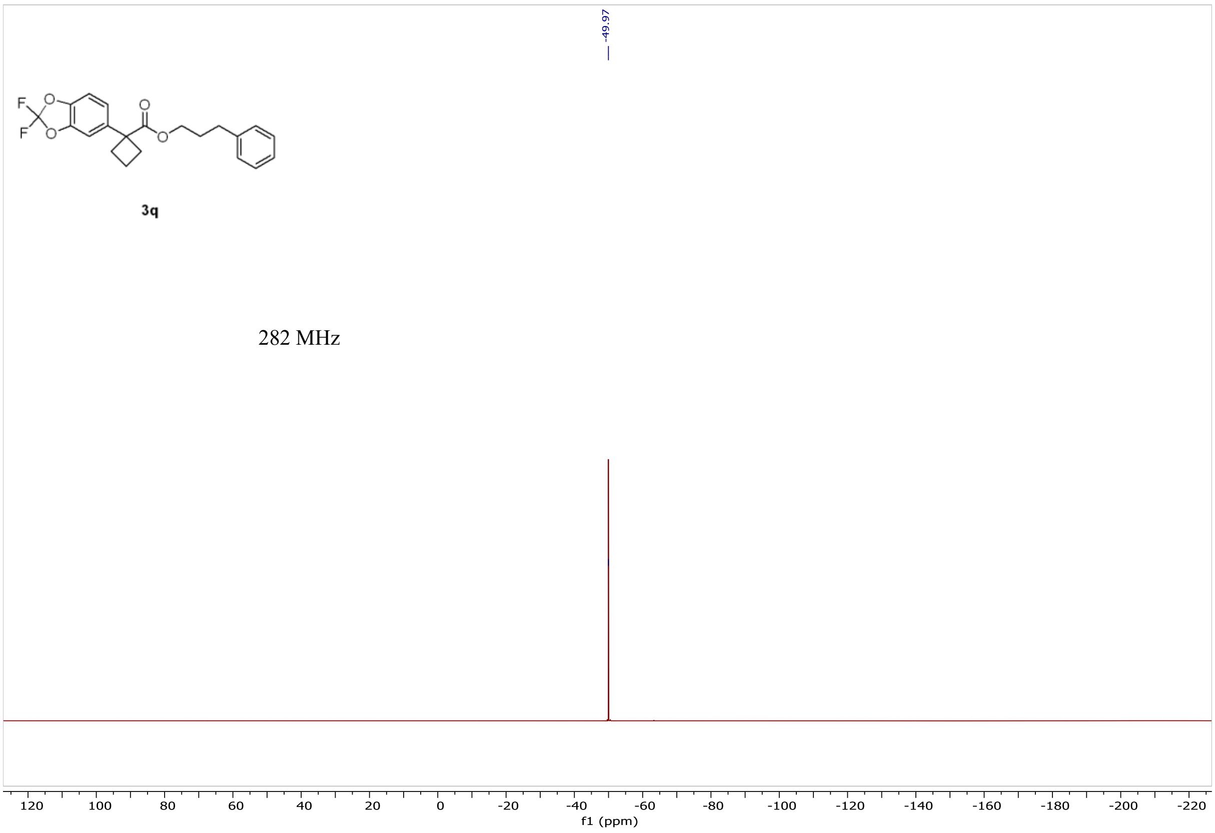

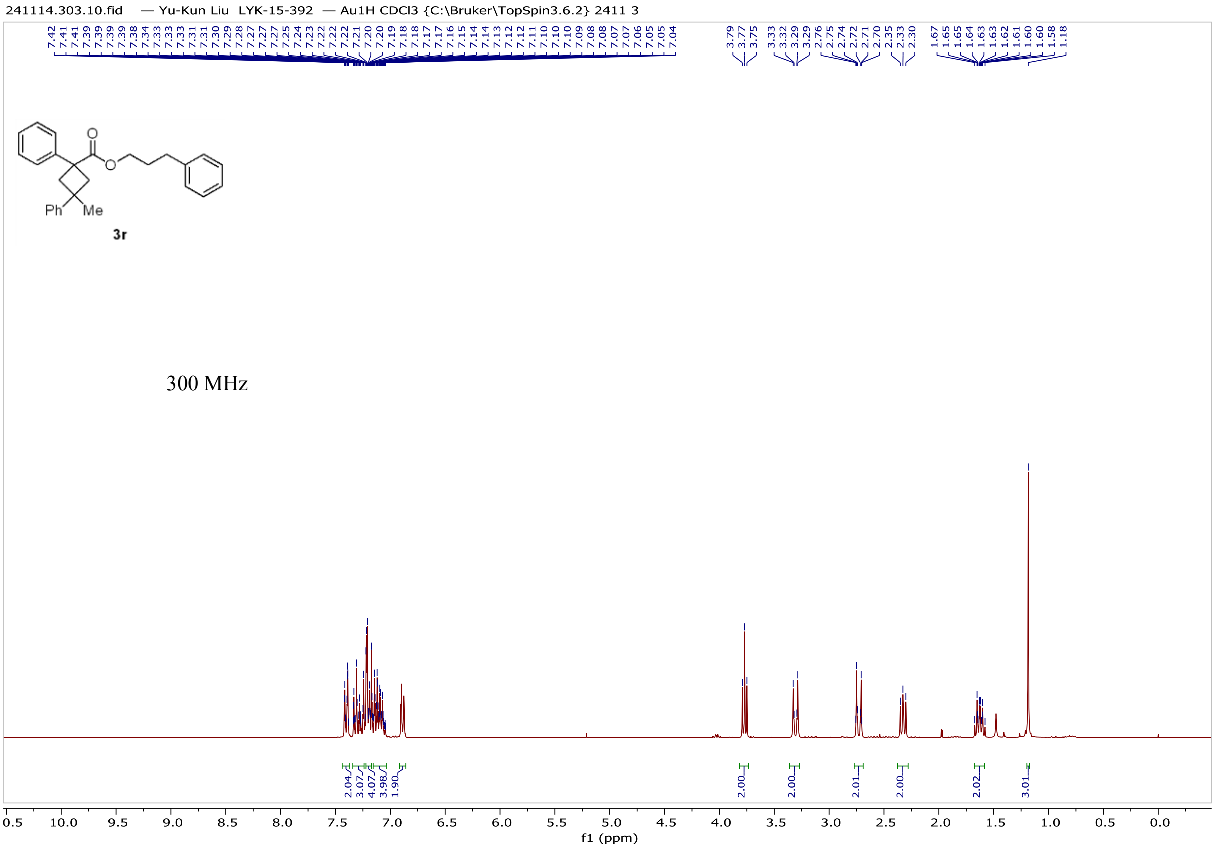
241114.303.10.fid — Yu-Kun Liu LYK-15-392 — Au1H CDCl3 {C:\Bruker\TopSpin3.6.2} 2411 3
7.42 7.41 7.41 7.39 7.39 7.39 7.38 7.34 7.33 7.33 7.31 7.31 7.30 7.29 7.28 7.27 7.27 7.25 7.24 7.23 7.22 7.22 7.21 7.20 7.20 7.19 7.18 7.17 7.17 7.16 7.15 7.14 7.13 7.12 7.12 7.11 7.10 7.10 7.09 7.08 7.08 7.07 7.07 7.06 7.05 7.04
3.79 3.77 3.75 3.33 3.32 3.29 3.29 2.76 2.75 2.74 2.72 2.71 2.70 2.35 2.33 2.30 1.67 1.65 1.65 1.64 1.63 1.63 1.62 1.61 1.60 1.60 1.58 1.18
Ph
Me
O
O
3r
300 MHz
2.04 3.07 4.07 3.98 1.90 2.00 2.00 2.01 2.00 2.02 3.01
0.5 10.0 9.5 9.0 8.5 8.0 7.5 7.0 6.5 6.0 5.5 5.0 4.5 4.0 3.5 3.0 2.5 2.0 1.5 1.0 0.5 0.0
f1 (ppm)

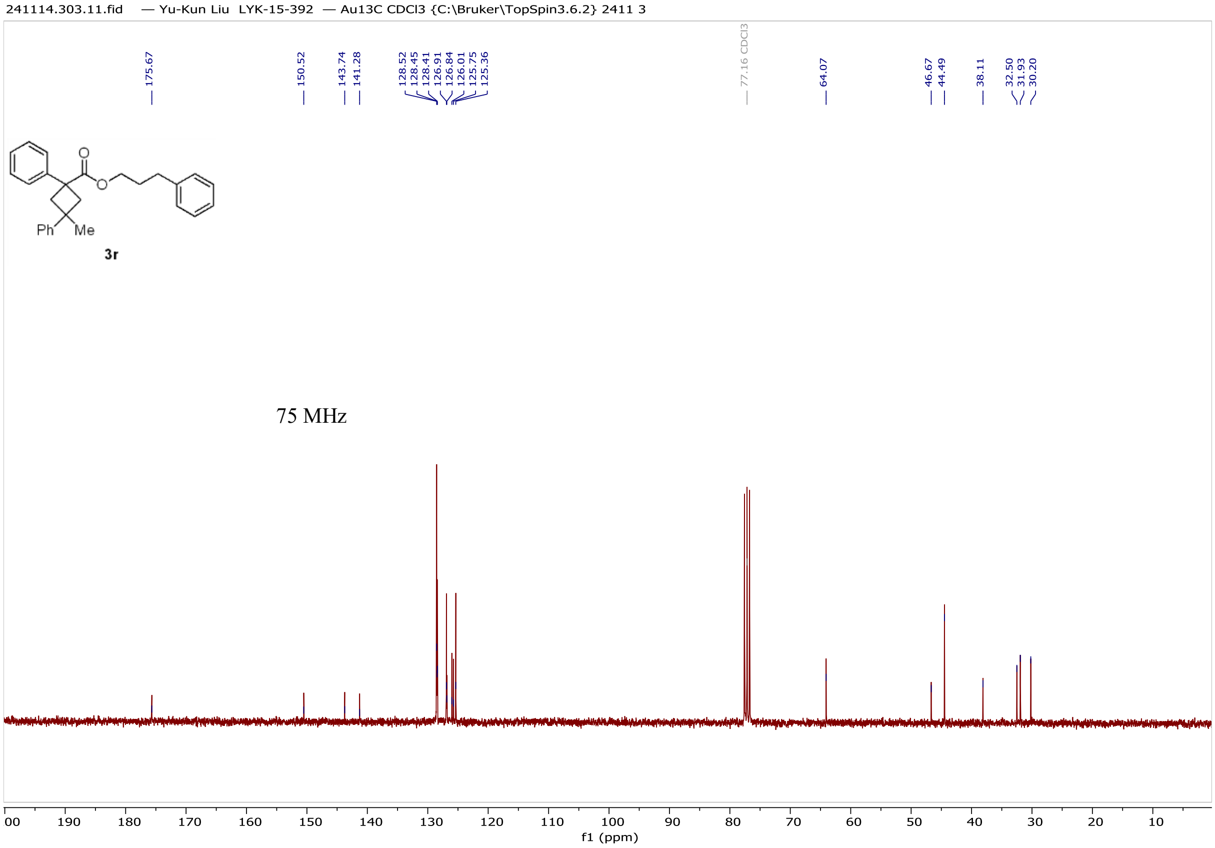
241114.303.11.fid — Yu-Kun Liu LYK-15-392 — Au13C CDCl3 {C:\Bruker\TopSpin3.6.2} 2411 3
175.67 150.52 143.74 141.28 128.52 128.45 128.41 126.91 126.84 126.01 125.75 125.36 77.16 CDCl3 64.07 46.67 44.49 38.11 32.50 31.93 30.20
Ph
Me
O
O
3r
75 MHz
00 190 180 170 160 150 140 130 120 110 100 90 80 70 60 50 40 30 20 10
f1 (ppm)

241120.302.10.fid — Yu-kun Liu LYK-15-404 — Au1H CDCl3 {C:\Bruker\TopSpin3.6.2} 2411 2

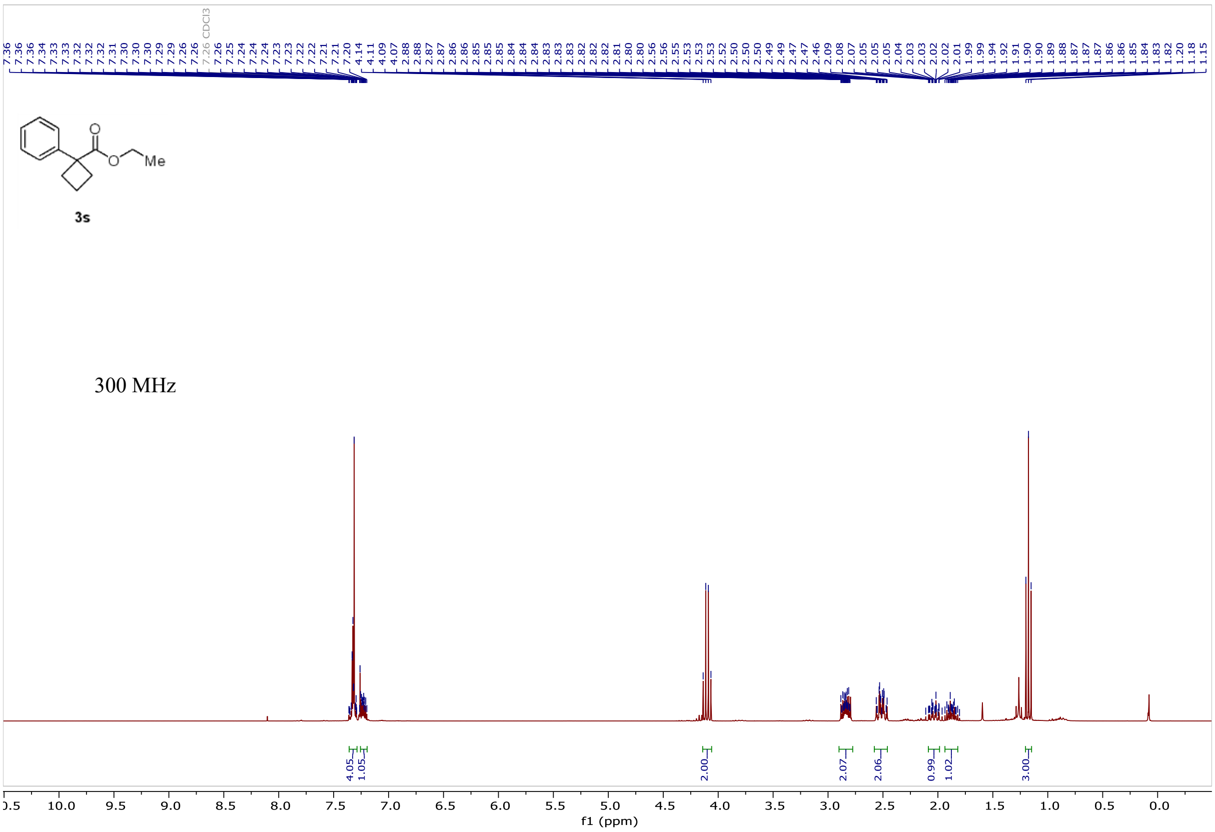

241120.302.11.fid — Yu-kun Liu LYK-15-404 — Au13C CDCl3 {C:\Bruker\TopSpin3.6.2} 2411 2

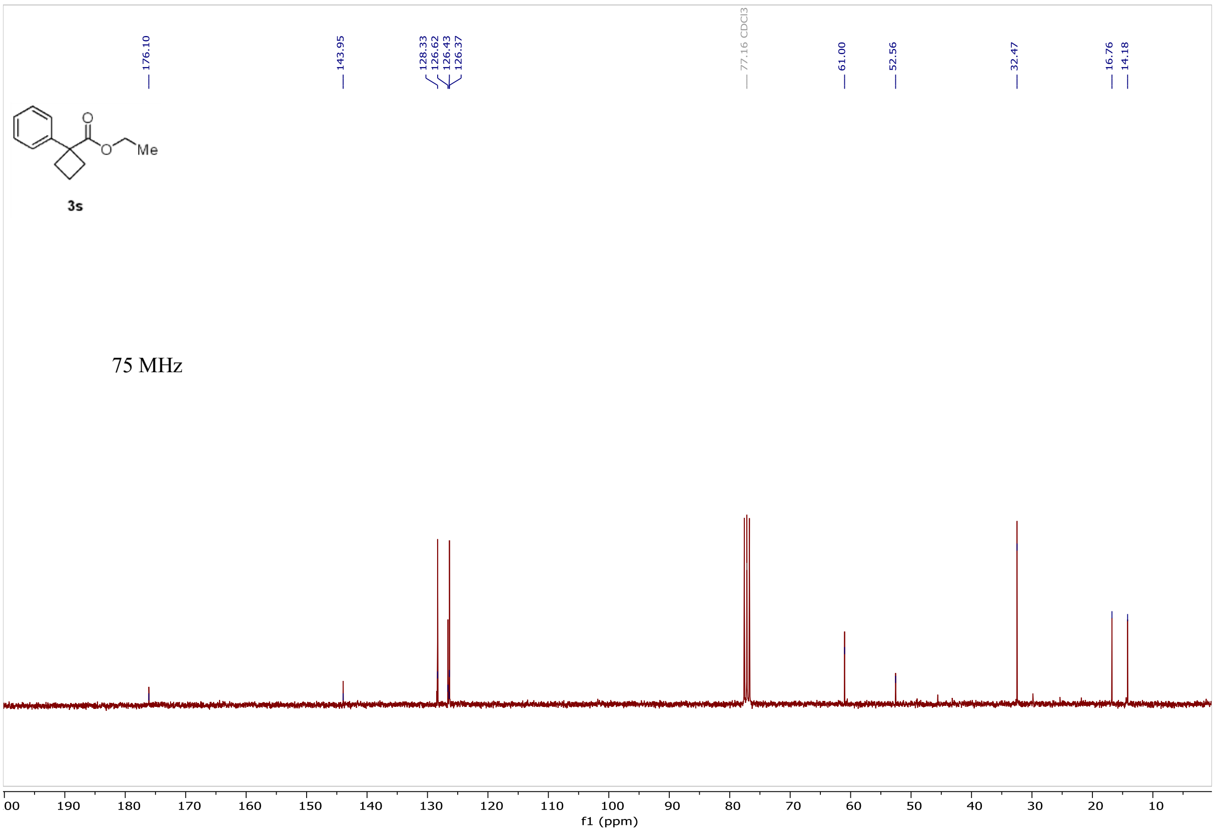

241120.303.10.fid — Yu-kun Liu LYK-15-405 — Au1H CDCl3 {C:\Bruker\TopSpin3.6.2} 2411 3

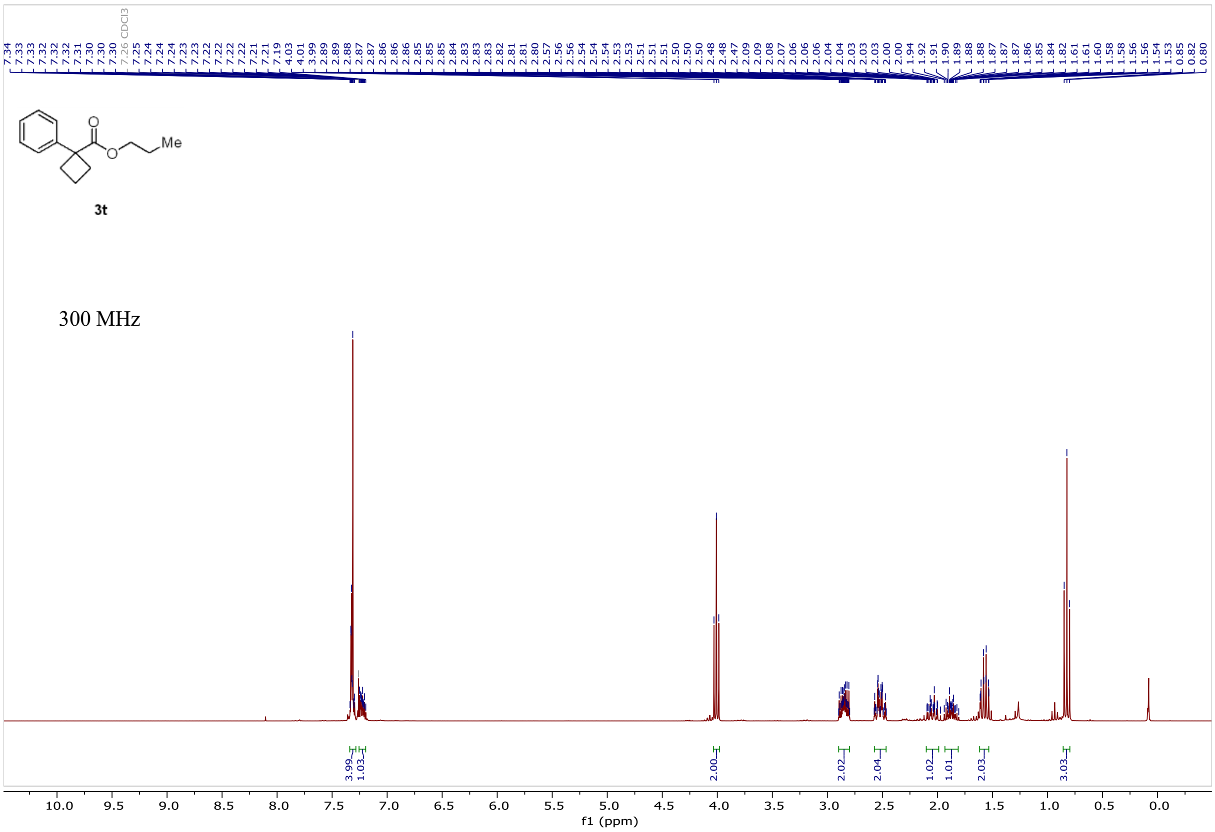

241120.303.11.fid — Yu-kun Liu LYK-15-405 — Au13C CDCl3 {C:\Bruker\TopSpin3.6.2} 2411 3

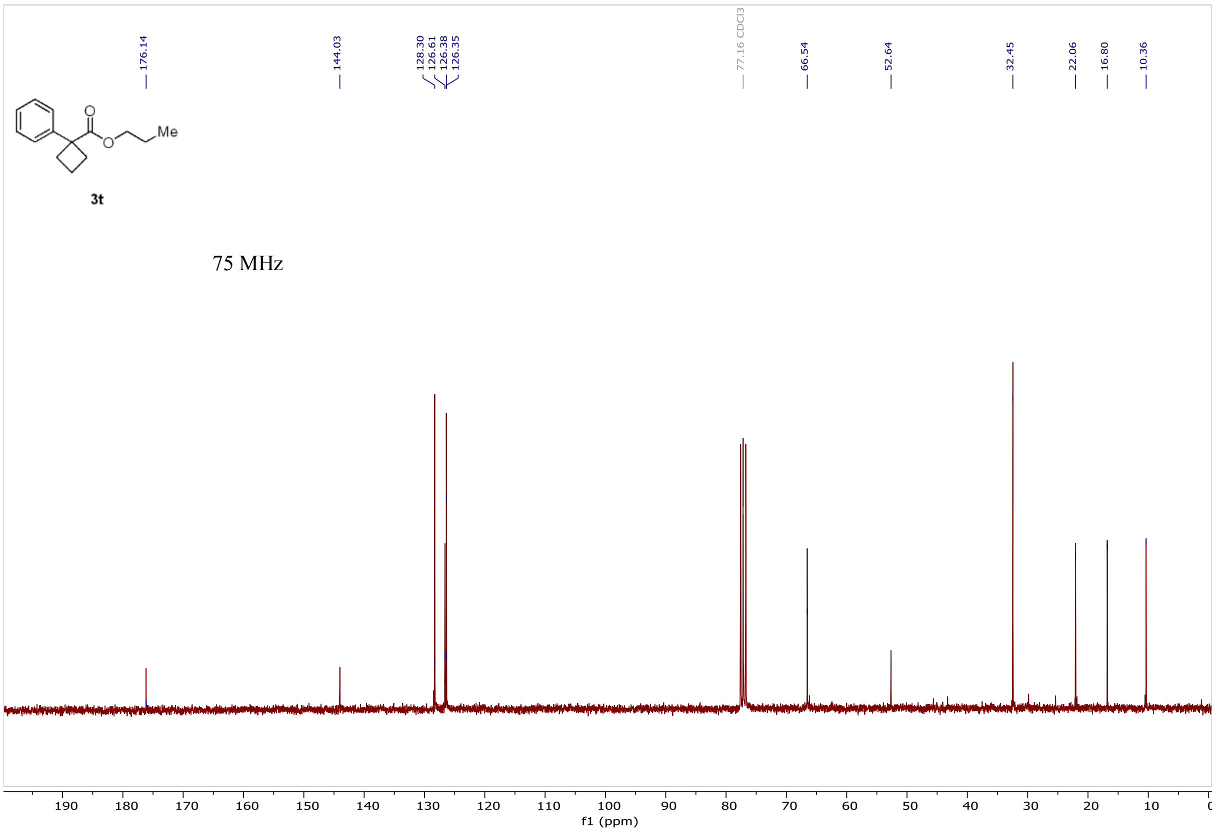

241202.408.10.fid — Yu-kun Liu LYK-15-437 — Au1H CDCl3 {C:\Bruker\TopSpin3.6.2} 2412 8

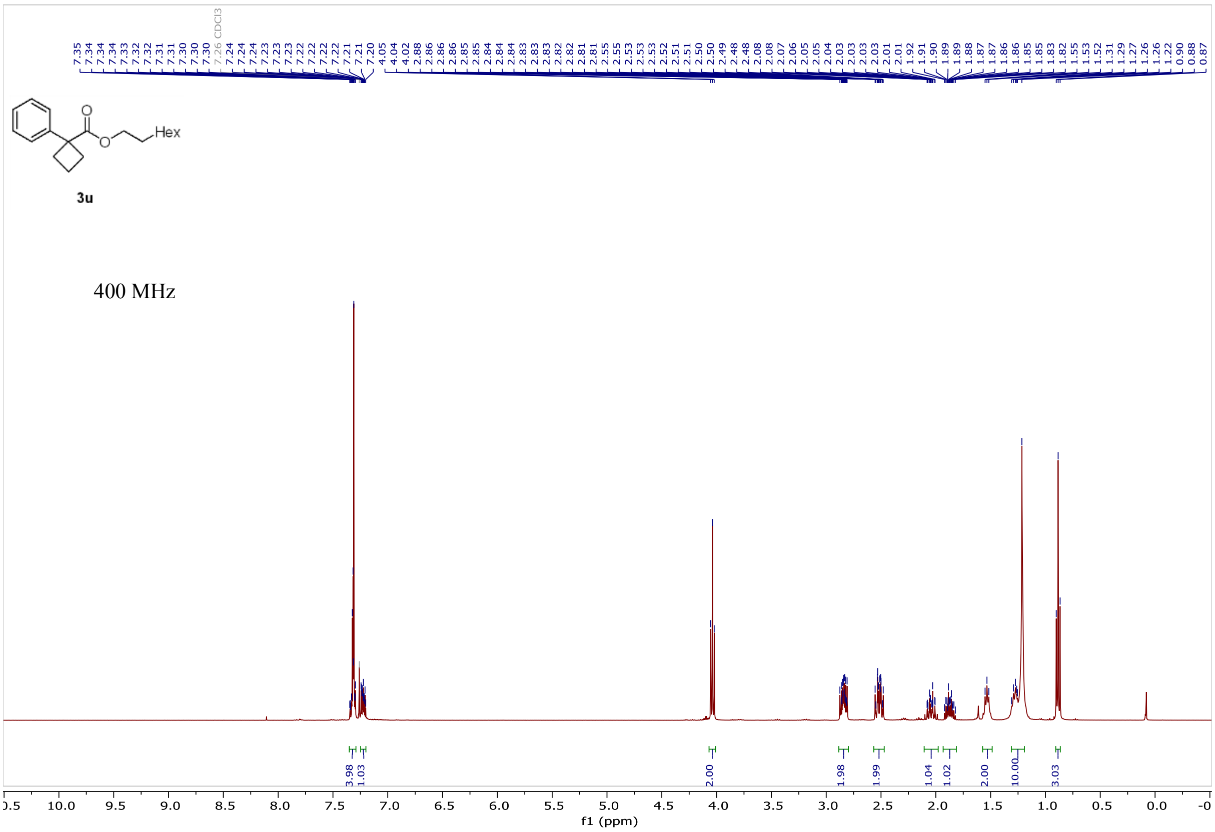

241202.408.11.fid — Yu-kun Liu LYK-15-437 — Au13C CDCl3 {C:\Bruker\TopSpin3.6.2} 2412 8

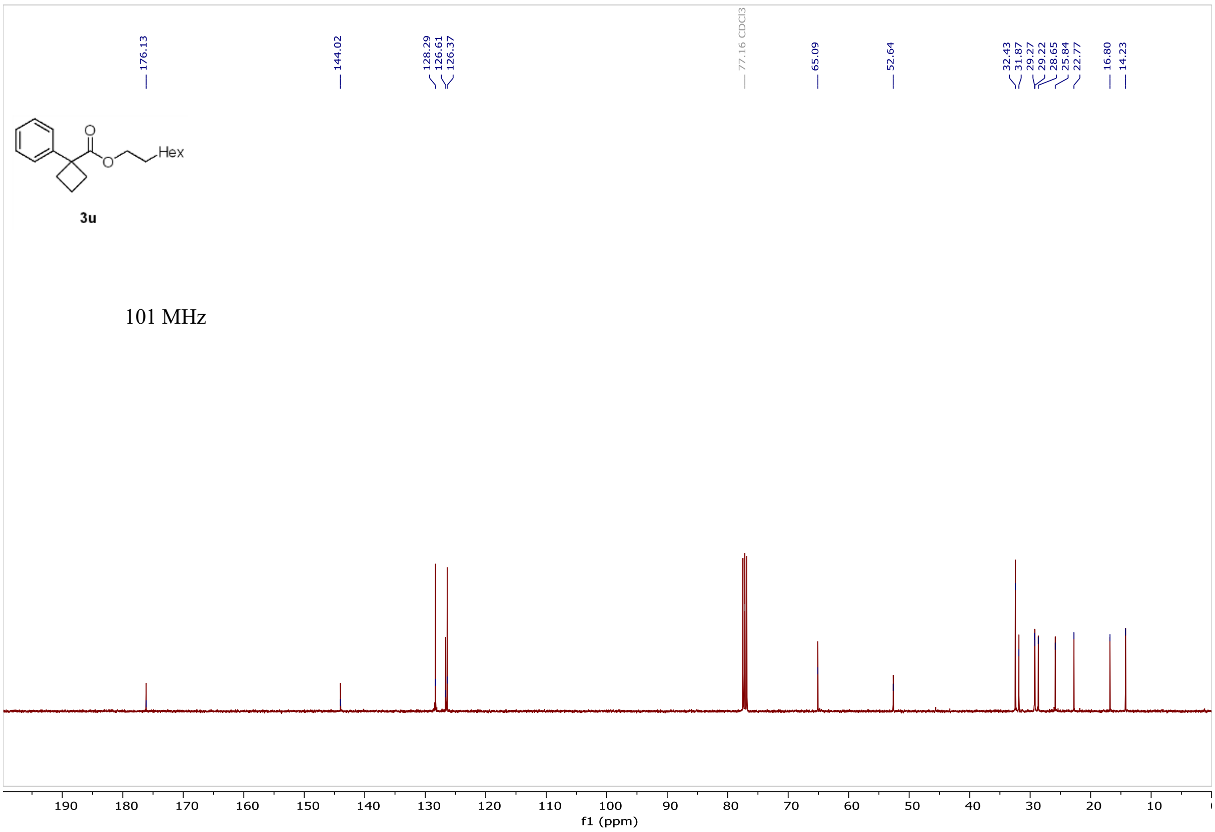

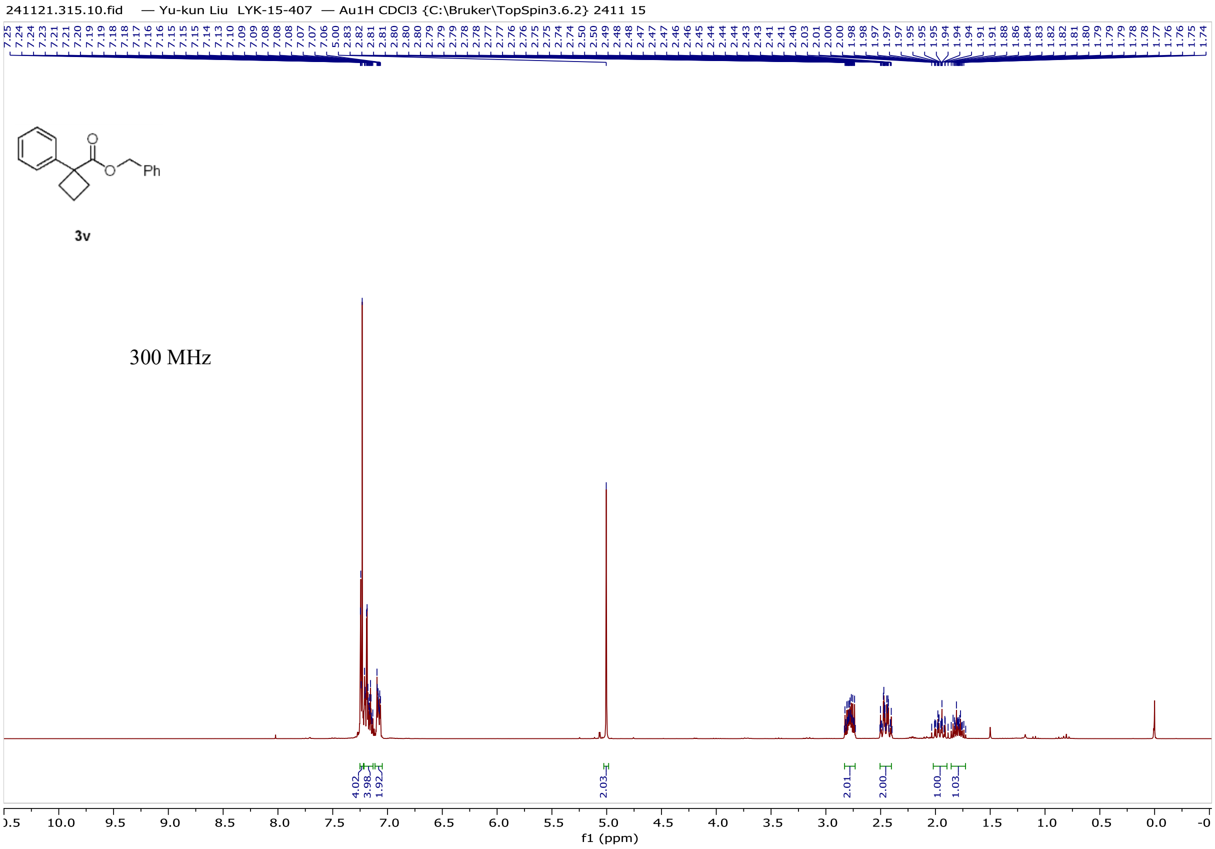
241121.315.10.fid — Yu-kun Liu LYK-15-407 — Au1H CDCl3 {C:\Bruker\TopSpin3.6.2} 2411 15
O
O
Ph
3v
300 MHz
4.02
3.98
1.92
2.03
2.01
2.00
1.00
1.03
f1 (ppm)

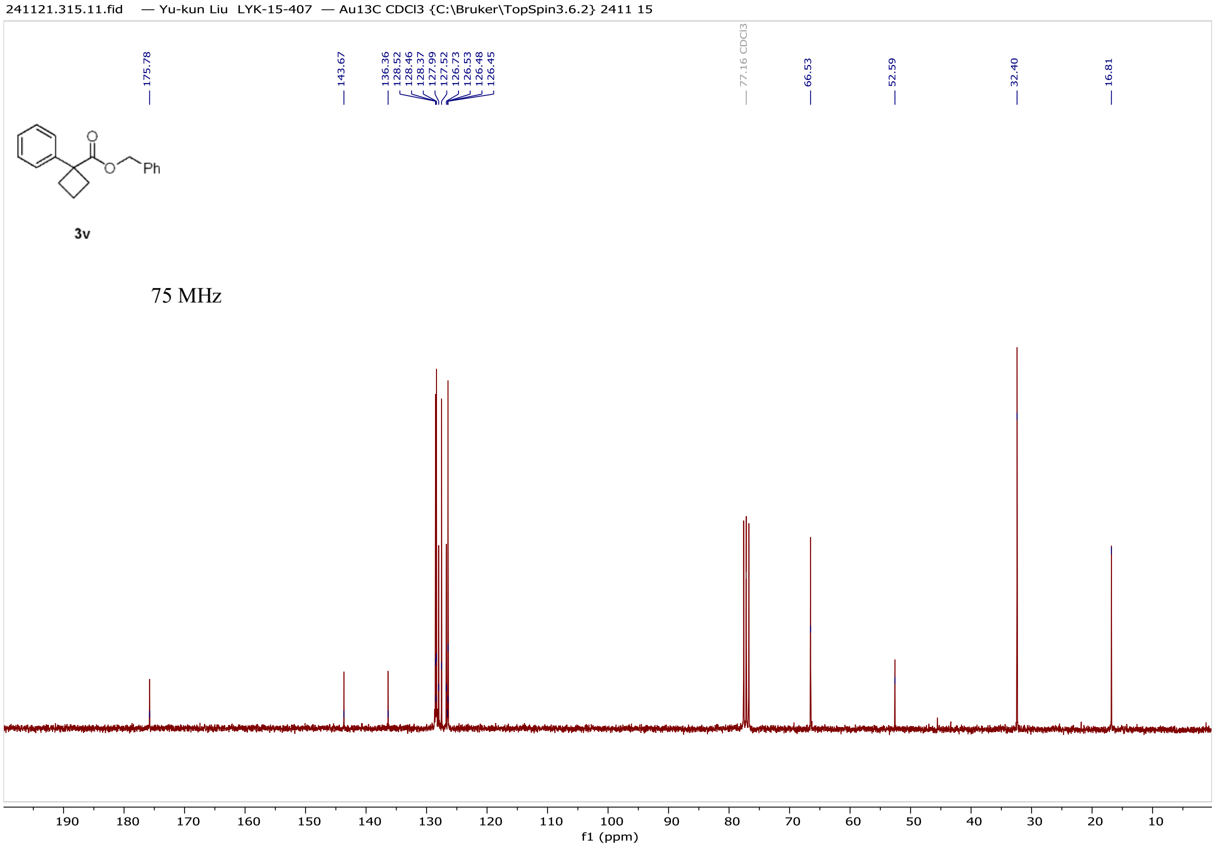
241121.315.11.fid — Yu-kun Liu LYK-15-407 — Au13C CDCl3 {C:\Bruker\TopSpin3.6.2} 2411 15
175.78
143.67
136.36
128.52
128.46
128.37
127.99
127.52
126.73
126.53
126.48
126.45
77.16 CDCl3
66.53
52.59
32.40
16.81
O
O
Ph
3v
75 MHz
f1 (ppm)

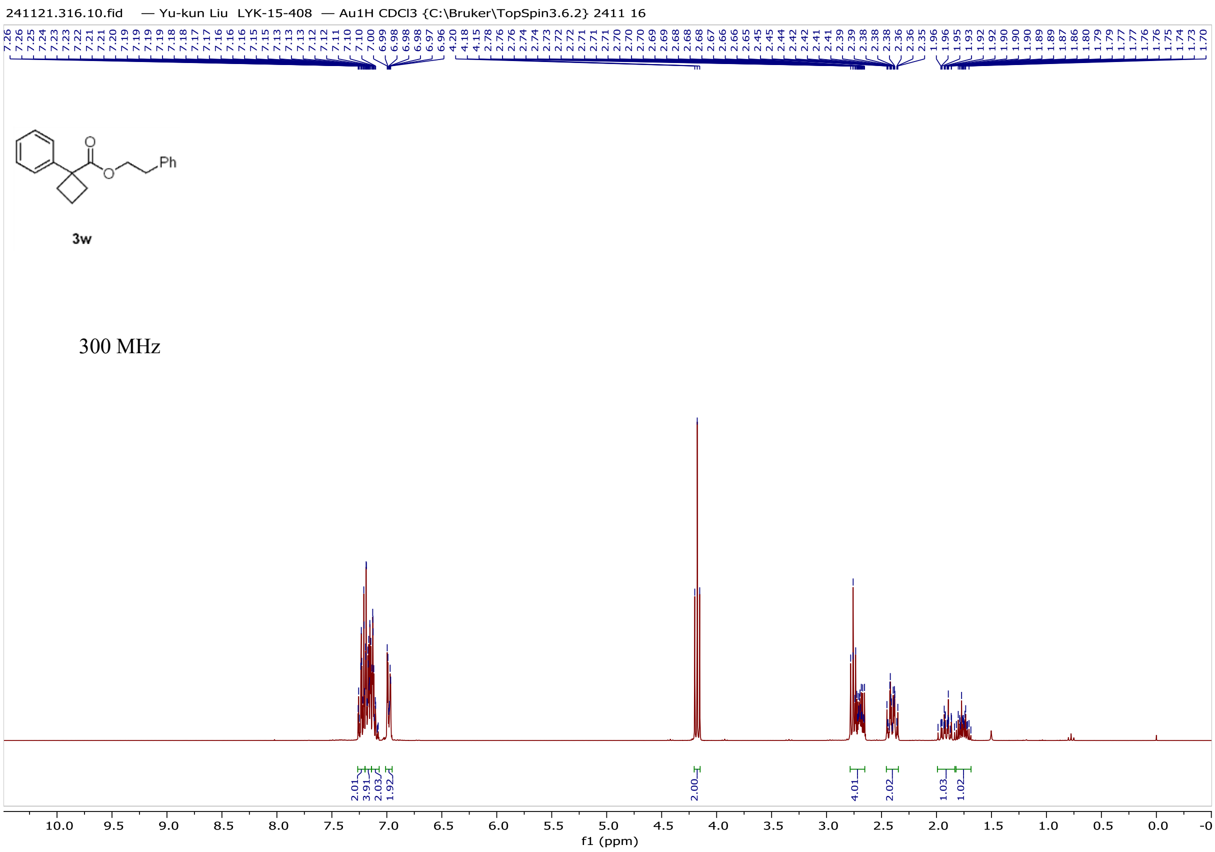
241121.316.10.fid — Yu-kun Liu LYK-15-408 — Au1H CDCl3 {C:\Bruker\TopSpin3.6.2} 2411 16
7.26 7.26 7.25 7.24 7.23 7.23 7.22 7.21 7.21 7.20 7.19 7.19 7.19 7.19 7.18 7.18 7.17 7.17 7.16 7.16 7.16 7.15 7.15 7.13 7.13 7.13 7.12 7.11 7.10 7.10 7.00 6.99 6.98 6.98 6.98 6.97 6.96 4.20 4.18 4.15 2.78 2.76 2.76 2.74 2.74 2.73 2.72 2.72 2.71 2.71 2.71 2.70 2.70 2.70 2.69 2.69 2.68 2.68 2.68 2.67 2.66 2.66 2.65 2.45 2.45 2.44 2.42 2.42 2.41 2.41 2.39 2.39 2.38 2.38 2.38 2.36 2.36 2.35 1.96 1.96 1.95 1.93 1.92 1.92 1.90 1.90 1.90 1.89 1.87 1.86 1.80 1.79 1.79 1.77 1.77 1.76 1.75 1.75 1.74 1.73 1.70
Ph
O
3w
300 MHz
2.01 3.91 2.03 1.92 2.00 4.01 2.02 1.03 1.02
f1 (ppm)

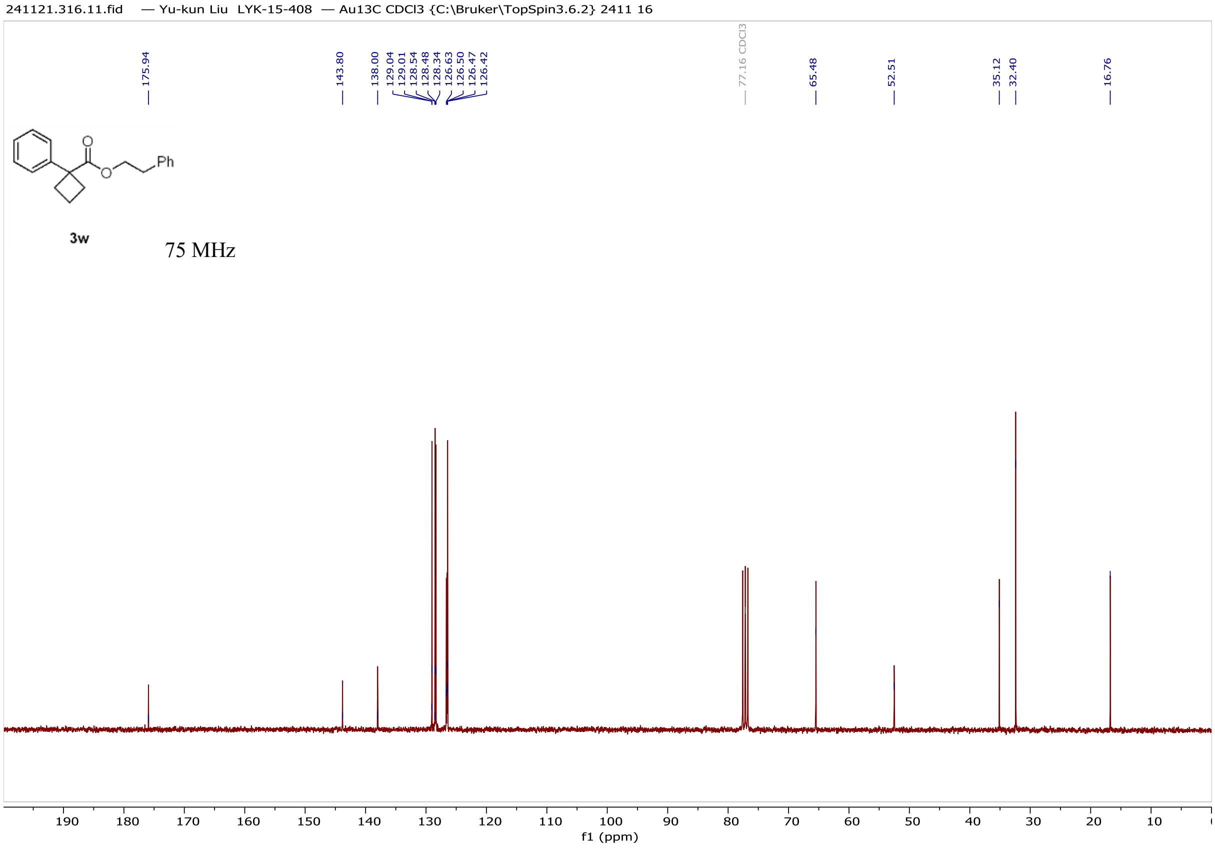
241121.316.11.fid — Yu-kun Liu LYK-15-408 — Au13C CDCl3 {C:\Bruker\TopSpin3.6.2} 2411 16
175.94
143.80 138.00 129.04 129.01 128.54 128.48 128.34 126.63 126.50 126.47 126.42
77.16 CDCl3
65.48
52.51
35.12 32.40
16.76
Ph
O
3w
75 MHz
f1 (ppm)

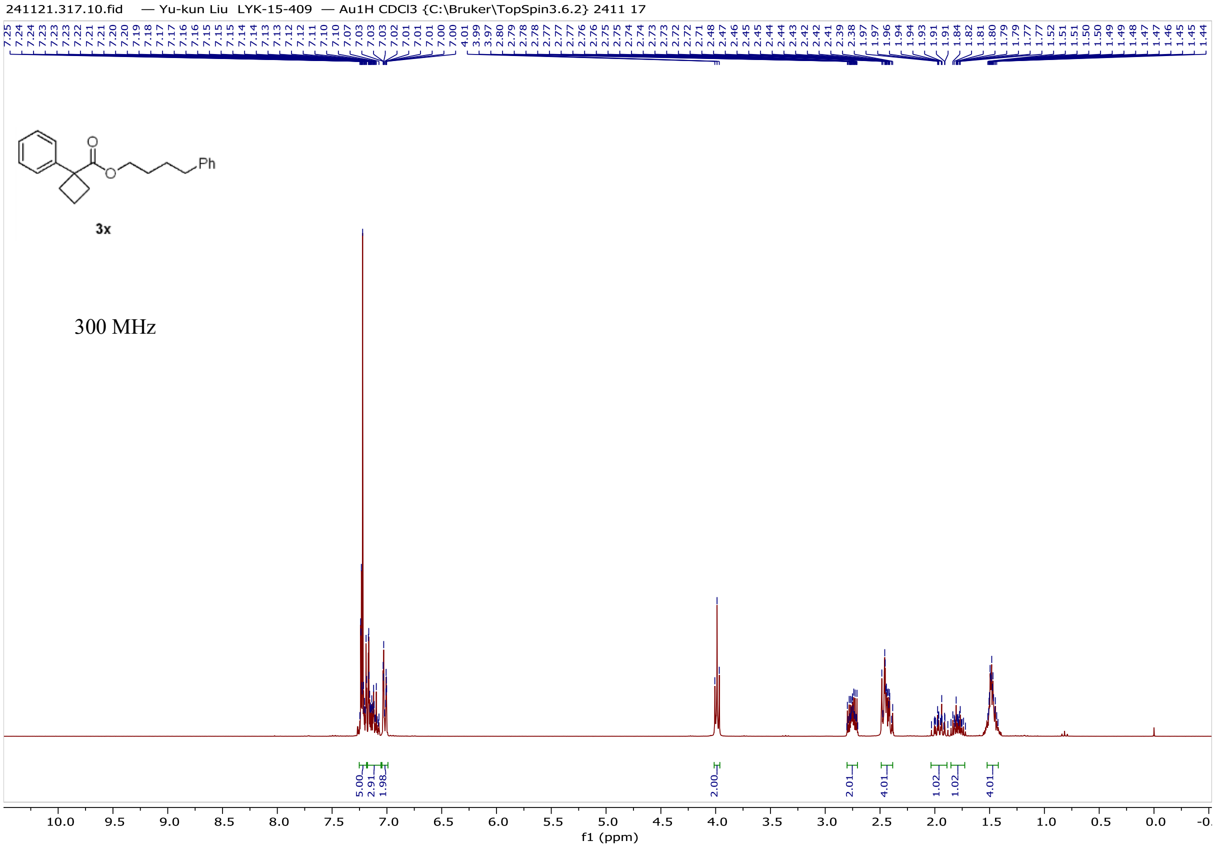

241121.317.10.fid — Yu-kun Liu LYK-15-409 — Au1H CDCl3 {C:\Bruker\TopSpin3.6.2} 2411 17
O
O
Ph
3x
300 MHz
5.00
2.91
1.98
2.00
2.01
4.01
1.02
1.02
4.01
10.0 9.5 9.0 8.5 8.0 7.5 7.0 6.5 6.0 5.5 5.0 4.5 4.0 3.5 3.0 2.5 2.0 1.5 1.0 0.5 0.0
f1 (ppm)

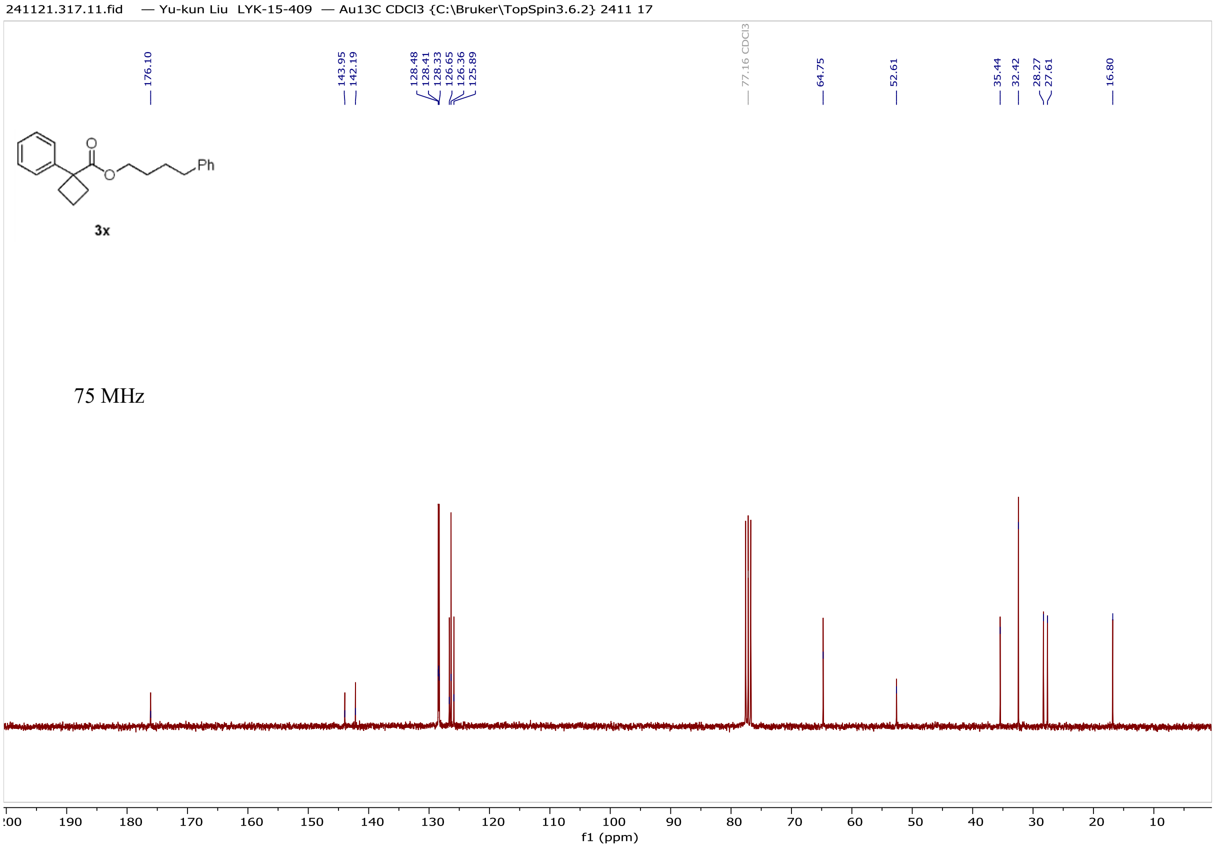

241121.317.11.fid — Yu-kun Liu LYK-15-409 — Au13C CDCl3 {C:\Bruker\TopSpin3.6.2} 2411 17
176.10
143.95
142.19
128.48
128.41
128.33
126.65
126.36
125.89
77.16 CDCl3
64.75
52.61
35.44
32.42
28.27
27.61
16.80
O
O
Ph
3x
75 MHz
190 180 170 160 150 140 130 120 110 100 90 80 70 60 50 40 30 20 10
f1 (ppm)

241202.402.10.fid — Yu-kun Liu LYK-15-430 — Au1H CDCl3 {C:\Bruker\TopSpin3.6.2} 2412 2

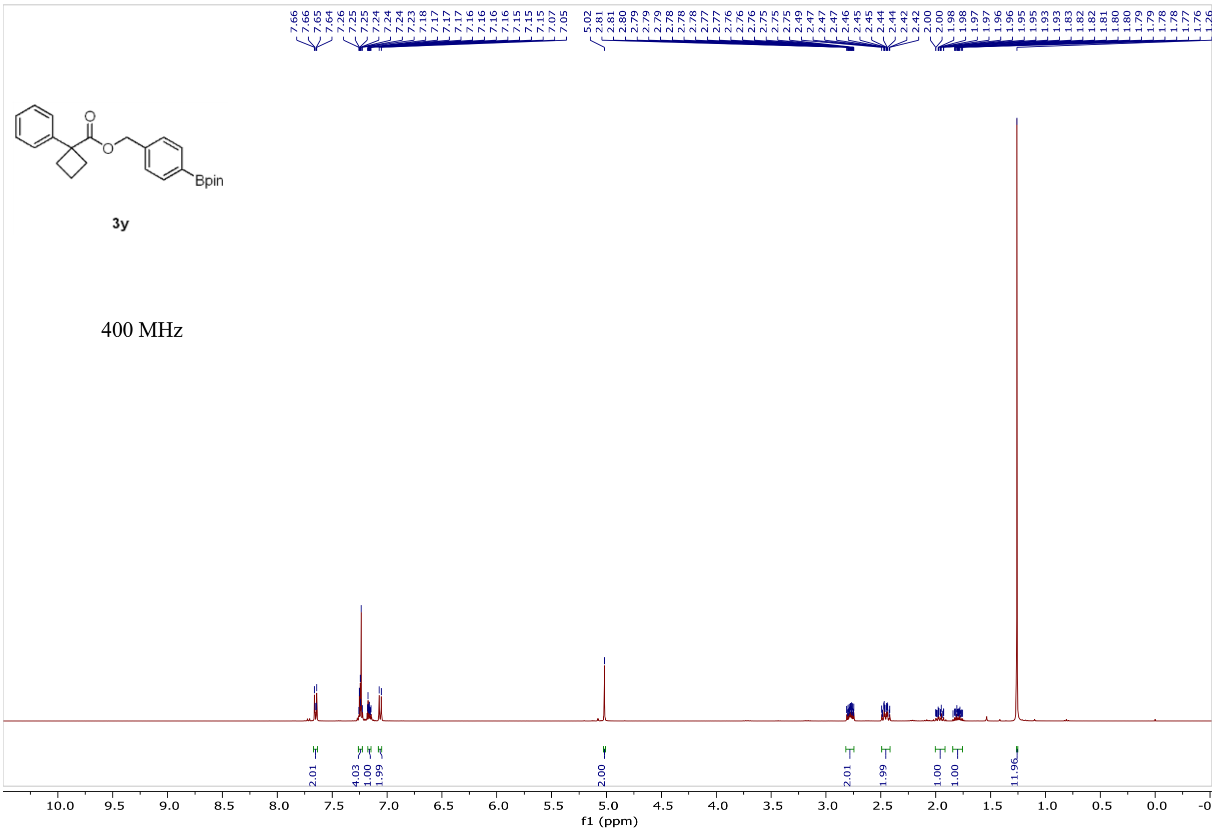

V

241202.402.11.fid — Yu-kun Liu LYK-15-430 — Au13C CDCl3 {C:\Bruker\TopSpin3.6.2} 2412 2

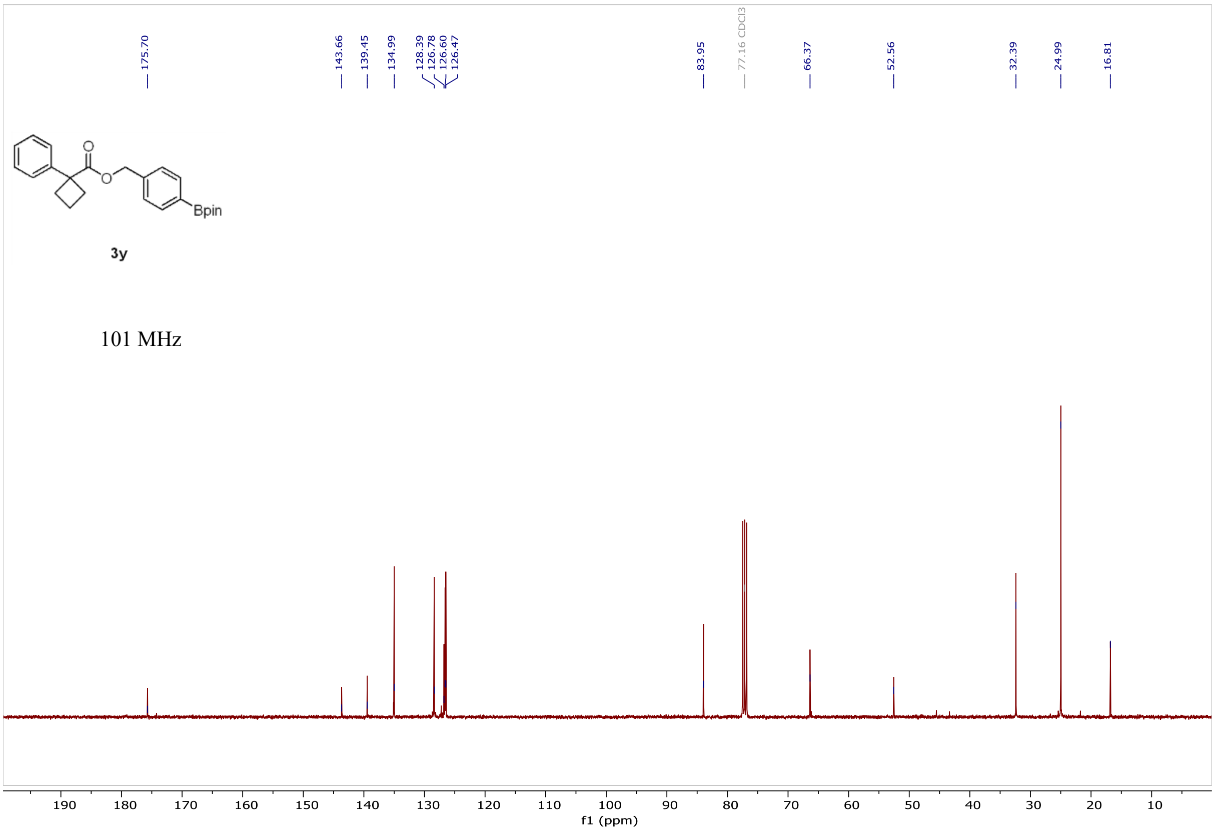

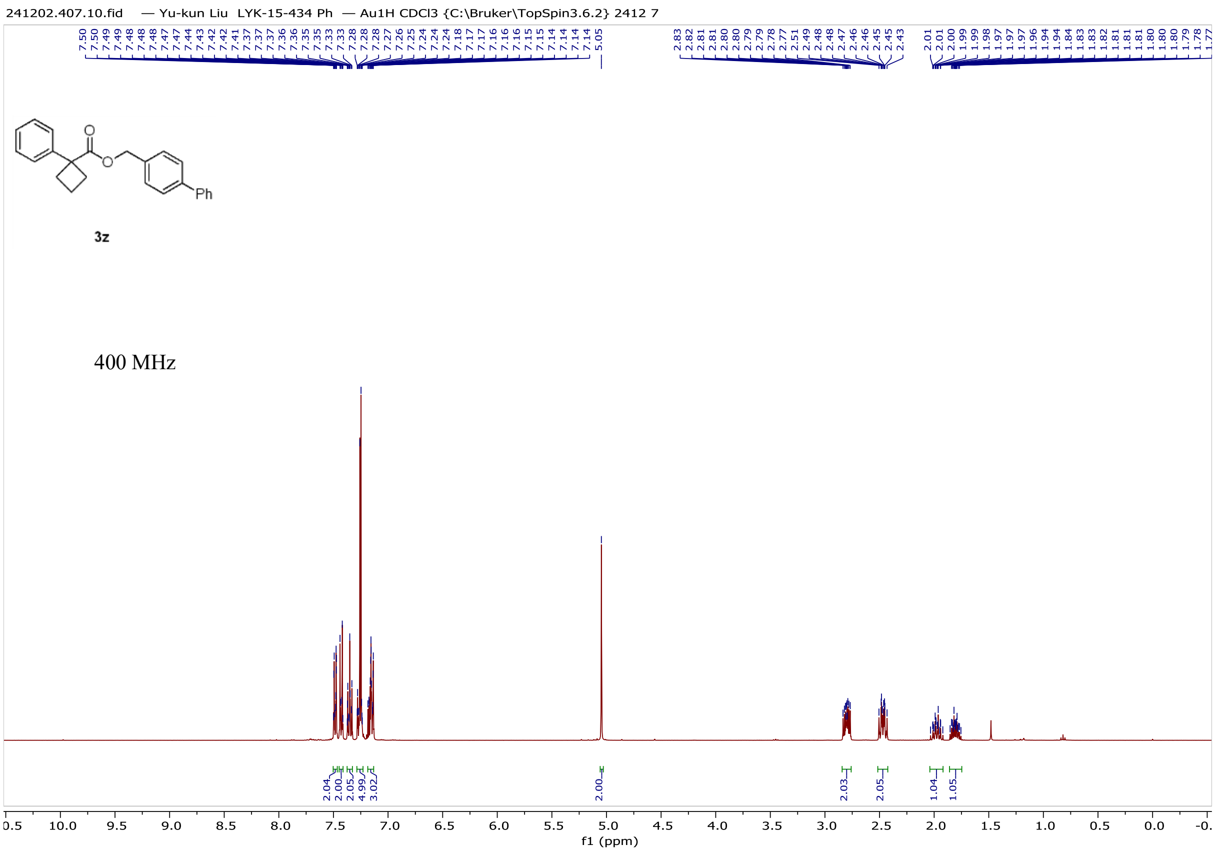
241202.407.10.fid — Yu-kun Liu LYK-15-434 Ph — Au1H CDCl3 {C:\Bruker\TopSpin3.6.2} 2412 7
7.50 7.50 7.49 7.48 7.48 7.47 7.47 7.44 7.43 7.42 7.41 7.37 7.37 7.36 7.35 7.35 7.33 7.33 7.28 7.28 7.27 7.26 7.25 7.24 7.24 7.24 7.18 7.17 7.17 7.16 7.16 7.15 7.15 7.14 7.14 7.14 7.14 5.05
2.83 2.82 2.81 2.80 2.80 2.79 2.79 2.78 2.77 2.51 2.49 2.48 2.48 2.47 2.47 2.46 2.45 2.45 2.43
2.01 2.01 2.00 1.99 1.99 1.98 1.97 1.97 1.97 1.96 1.94 1.94 1.84 1.83 1.83 1.82 1.81 1.81 1.81 1.80 1.80 1.79 1.78 1.77
O
O
Ph
3z
400 MHz
2.04 2.00 2.05 4.99 3.02 2.00 2.03 2.05 1.04 1.05
10.0 9.5 9.0 8.5 8.0 7.5 7.0 6.5 6.0 5.5 5.0 4.5 4.0 3.5 3.0 2.5 2.0 1.5 1.0 0.5 0.0
f1 (ppm)

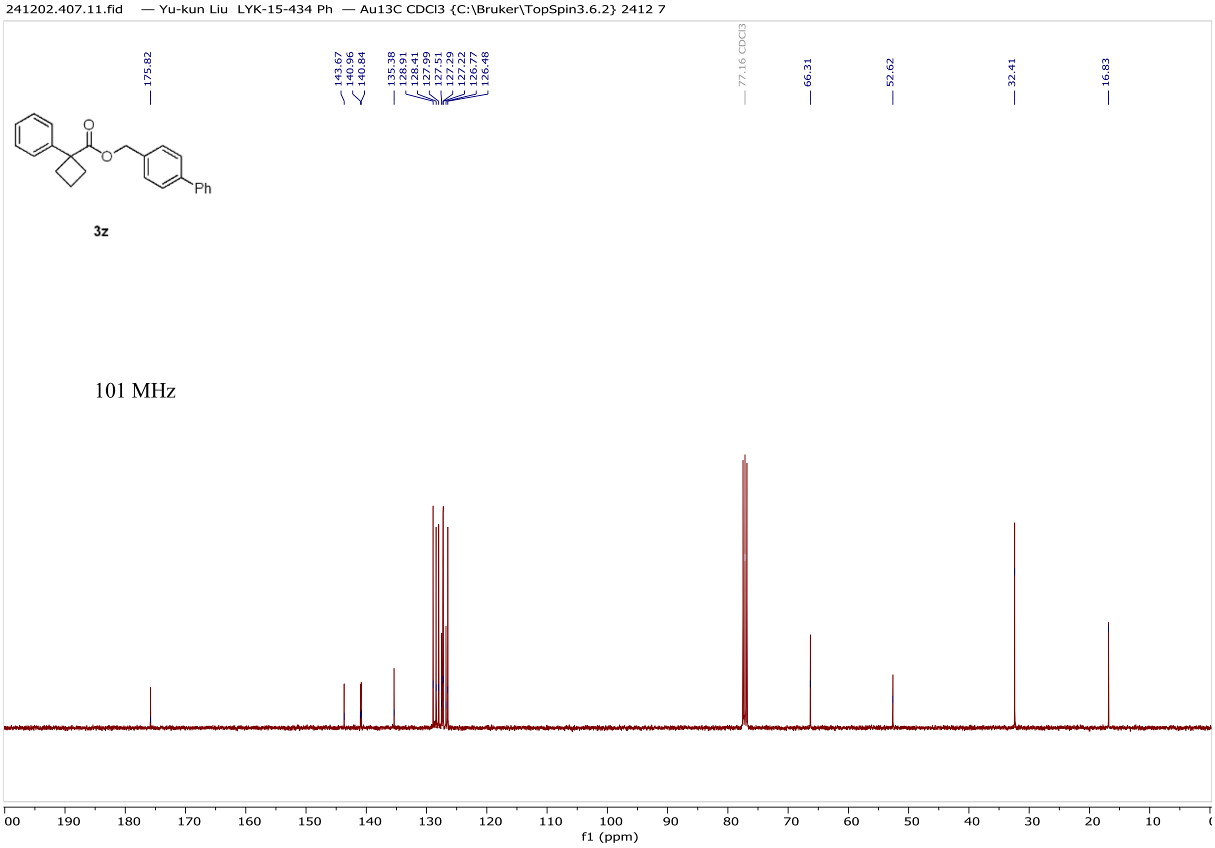
241202.407.11.fid — Yu-kun Liu LYK-15-434 Ph — Au13C CDCl3 {C:\Bruker\TopSpin3.6.2} 2412 7
175.82
143.67 140.96 140.84
135.38 128.91 128.41 127.99 127.51 127.29 127.22 126.77 126.48
77.16 CDCl3
66.31
52.62
32.41
16.83
O
O
Ph
3z
101 MHz
190 180 170 160 150 140 130 120 110 100 90 80 70 60 50 40 30 20 10
f1 (ppm)

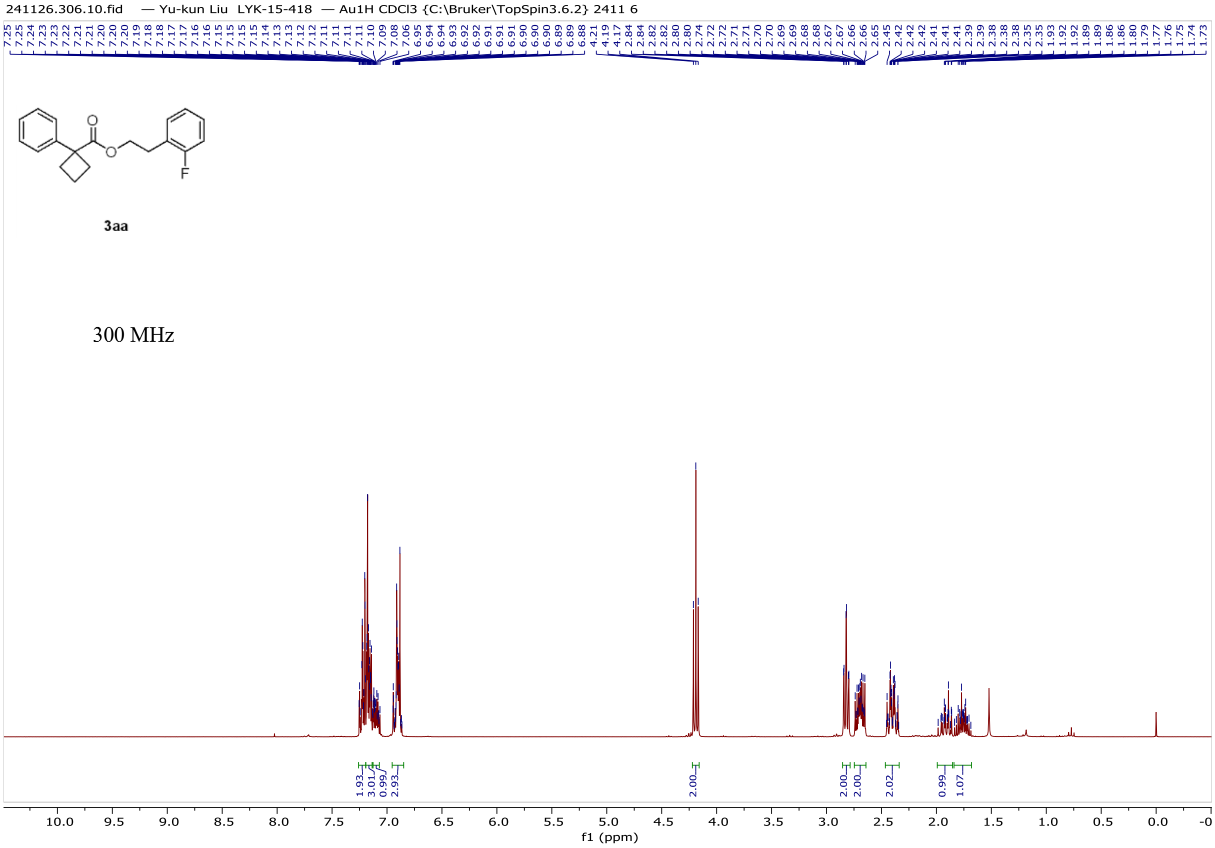
241126.306.10.fid — Yu-kun Liu LYK-15-418 — Au1H CDCl3 {C:\Bruker\TopSpin3.6.2} 2411 6
7.25 7.25 7.24 7.23 7.23 7.22 7.21 7.21 7.20 7.20 7.19 7.18 7.18 7.17 7.17 7.16 7.16 7.15 7.15 7.15 7.14 7.13 7.13 7.12 7.12 7.11 7.11 7.11 7.10 7.09 7.08 7.06 6.95 6.94 6.94 6.93 6.92 6.92 6.91 6.91 6.90 6.90 6.90 6.89 6.89 6.88 4.21 4.19 4.17 2.84 2.82 2.82 2.80 2.80 2.74 2.72 2.72 2.71 2.71 2.70 2.70 2.69 2.69 2.68 2.68 2.67 2.67 2.66 2.66 2.65 2.45 2.42 2.42 2.42 2.41 2.41 2.41 2.39 2.39 2.38 2.38 2.38 2.35 2.35 1.93 1.92 1.92 1.89 1.89 1.86 1.86 1.80 1.79 1.77 1.76 1.75 1.74 1.73
F
3aa
300 MHz
1.93 3.01 0.99 2.93 2.00 2.00 2.00 2.02 0.99 1.07
10.0 9.5 9.0 8.5 8.0 7.5 7.0 6.5 6.0 5.5 5.0 4.5 4.0 3.5 3.0 2.5 2.0 1.5 1.0 0.5 0.0
f1 (ppm)

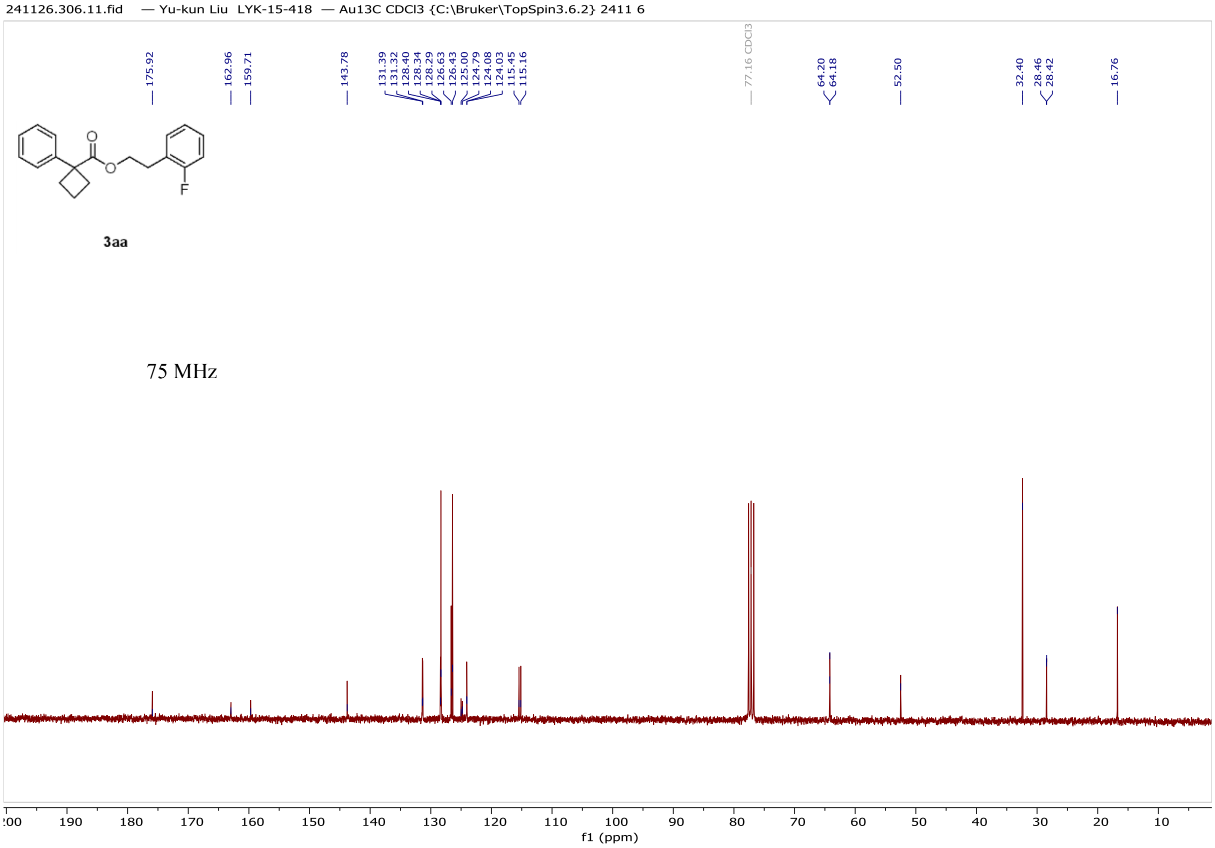
241126.306.11.fid — Yu-kun Liu LYK-15-418 — Au13C CDCl3 {C:\Bruker\TopSpin3.6.2} 2411 6
175.92
162.96 159.71
143.78
131.39 131.32 128.40 128.34 128.29 126.63 126.43 125.00 124.79 124.08 124.03 115.45 115.16
77.16 CDCl3
64.20 64.18
52.50
32.40 28.46 28.42
16.76
F
3aa
75 MHz
190 180 170 160 150 140 130 120 110 100 90 80 70 60 50 40 30 20 10
f1 (ppm)

241128.302.12.fid — Yu-Kun Liu LYK-15-418-re-F — Au19F CDCl3 {C:\Bruker\TopSpin3.6.2} 2411 2

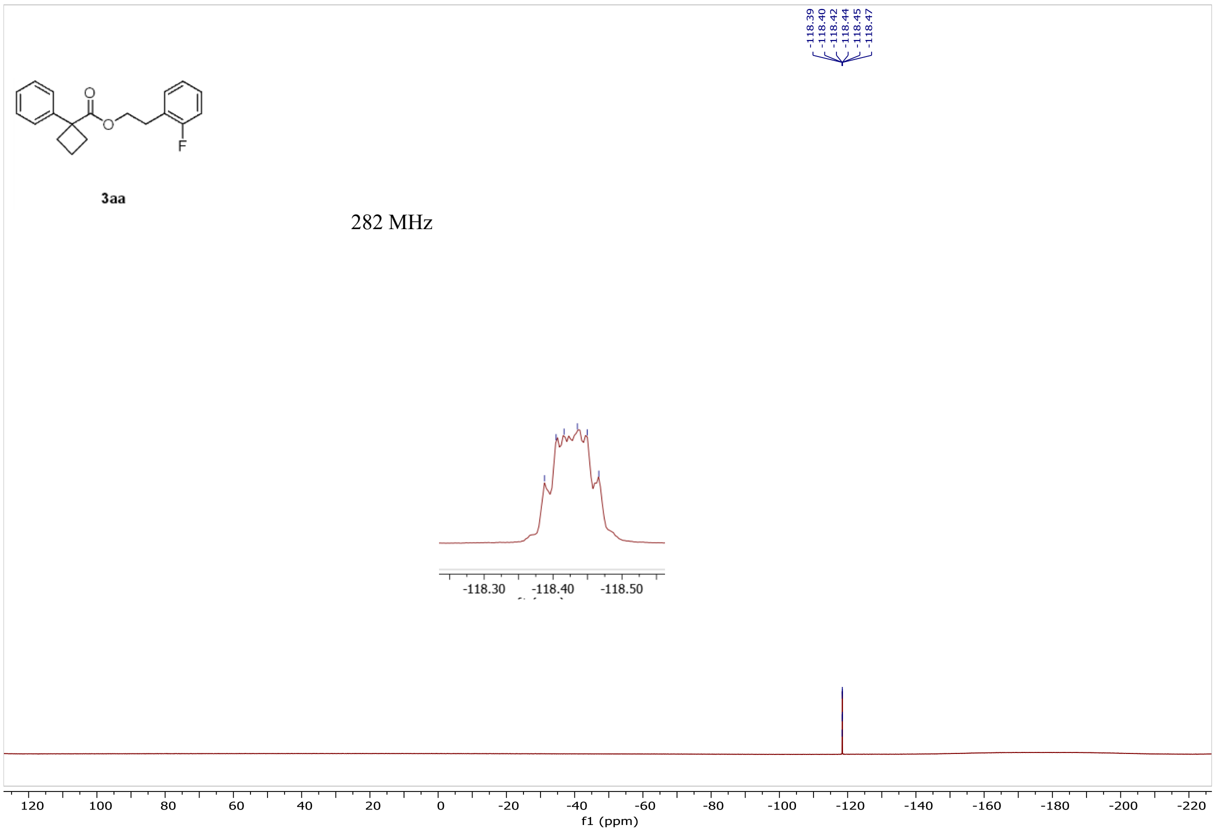

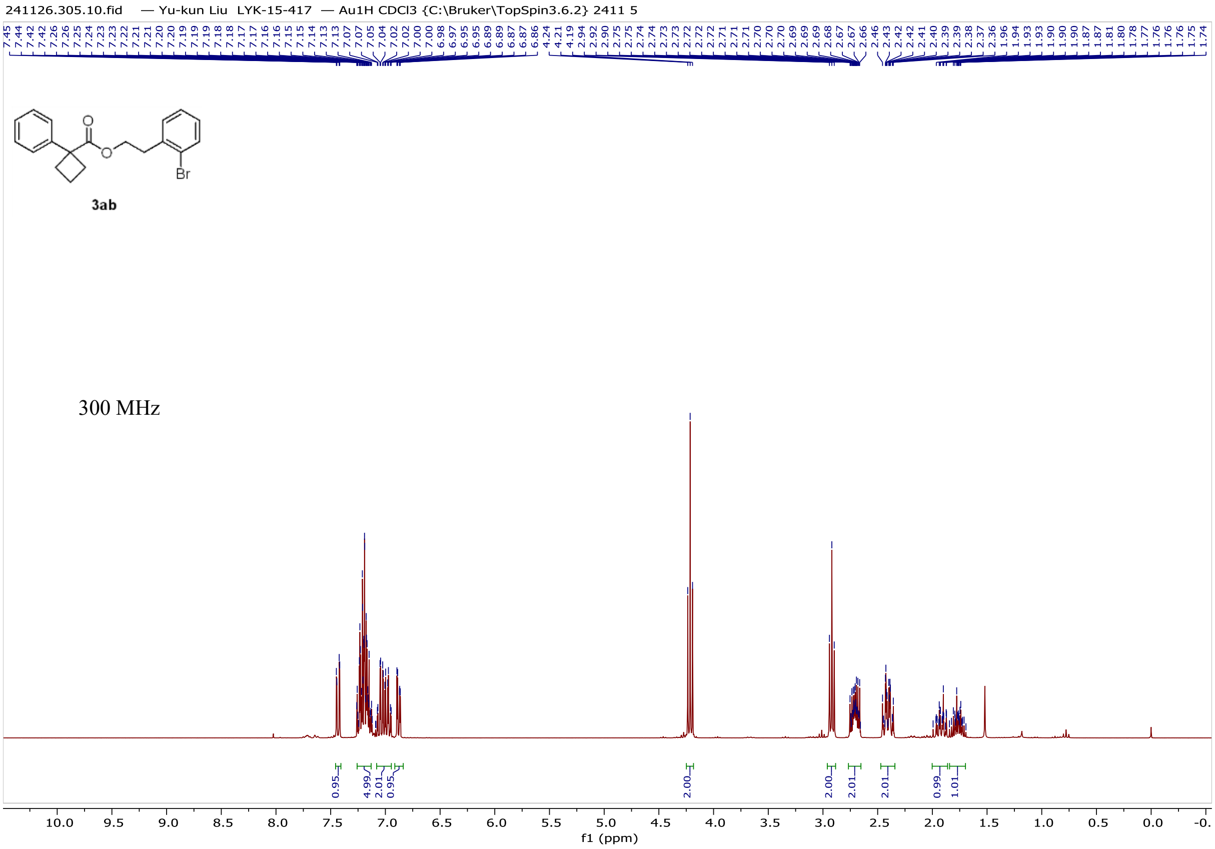
241126.305.10.fid — Yu-kun Liu LYK-15-417 — Au1H CDCl3 {C:\Bruker\TopSpin3.6.2} 2411 5
7.45 7.44 7.42 7.26 7.25 7.24 7.23 7.22 7.21 7.21 7.20 7.19 7.19 7.19 7.18 7.17 7.17 7.16 7.15 7.15 7.14 7.13 7.13 7.07 7.05 7.04 7.02 7.02 7.00 7.00 6.98 6.97 6.95 6.95 6.89 6.89 6.87 6.87 6.86 4.24 4.21 4.19 2.94 2.92 2.90 2.75 2.75 2.74 2.74 2.73 2.73 2.72 2.72 2.71 2.71 2.71 2.70 2.70 2.69 2.69 2.68 2.67 2.67 2.66 2.46 2.43 2.42 2.42 2.41 2.40 2.39 2.39 2.38 2.37 2.36 1.96 1.94 1.93 1.93 1.90 1.90 1.87 1.87 1.81 1.80 1.78 1.77 1.76 1.76 1.75 1.74
O
O
Br
3ab
300 MHz
0.95
4.99
2.01
0.95
2.00
2.00
2.01
2.01
0.99
1.01
f1 (ppm)

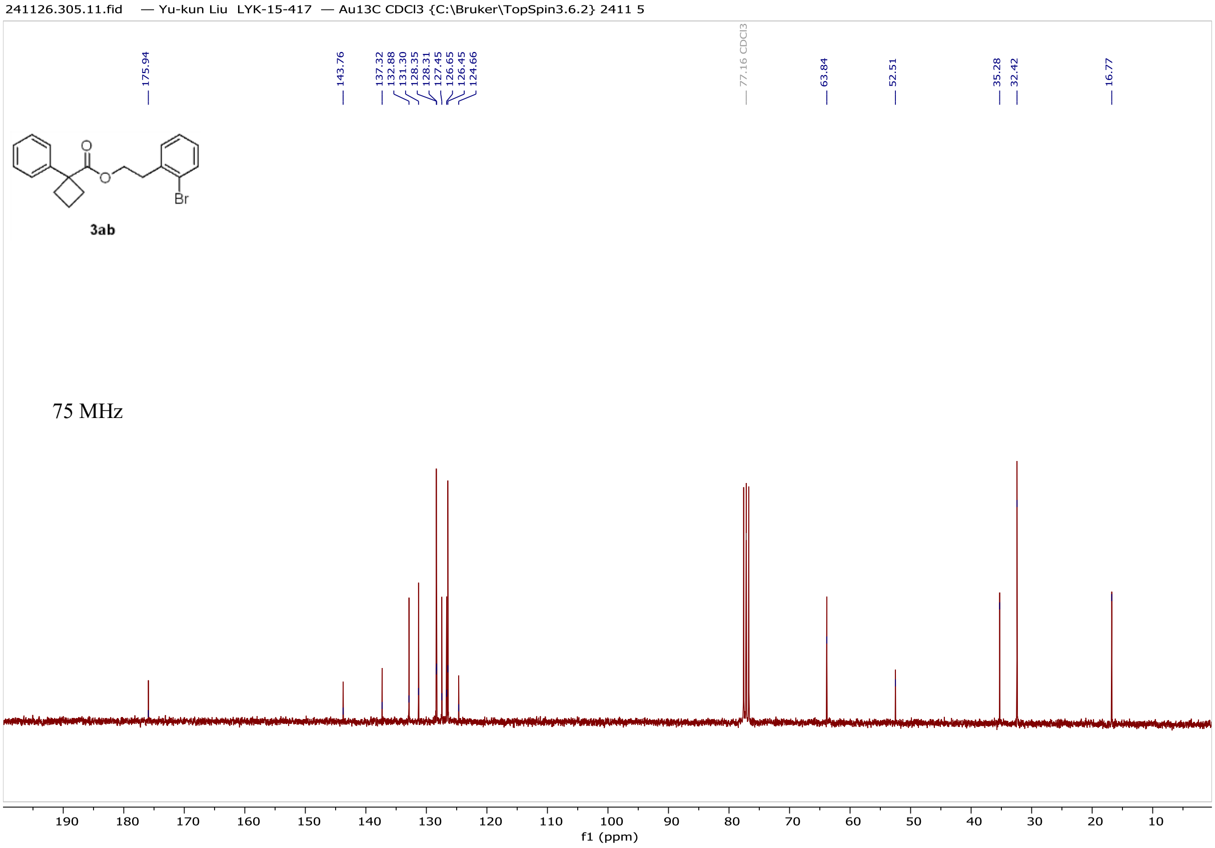
241126.305.11.fid — Yu-kun Liu LYK-15-417 — Au13C CDCl3 {C:\Bruker\TopSpin3.6.2} 2411 5
175.94
143.76
137.32 132.88 131.30 128.35 128.31 127.45 126.65 126.45 124.66
77.16 CDCl3
63.84
52.51
35.28
32.42
16.77
O
O
Br
3ab
75 MHz
f1 (ppm)

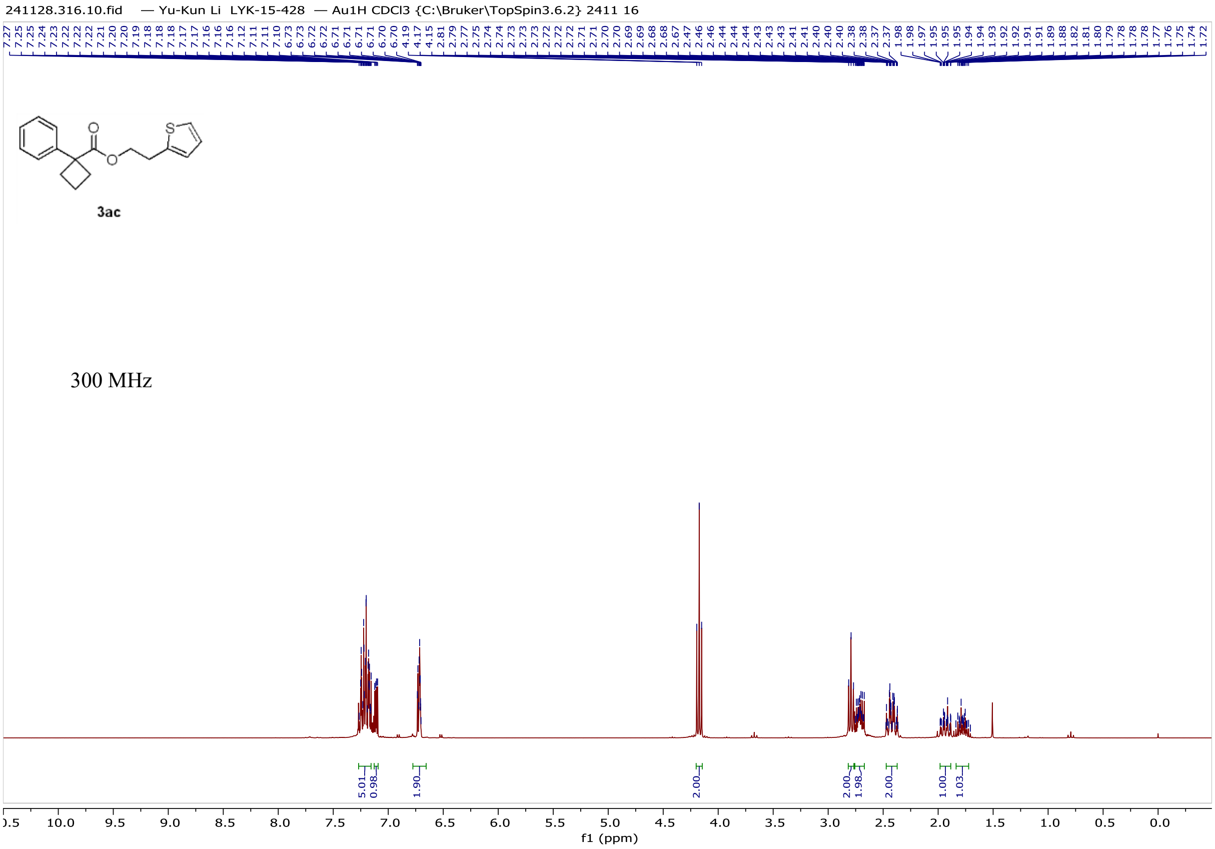
241128.316.10.fid — Yu-Kun Li LYK-15-428 — Au1H CDCl3 {C:\Bruker\TopSpin3.6.2} 2411 16
7.27 7.25 7.25 7.24 7.23 7.22 7.22 7.22 7.21 7.20 7.20 7.19 7.18 7.18 7.17 7.17 7.16 7.16 7.16 7.12 7.11 7.11 7.10 6.73 6.73 6.72 6.72 6.72 6.71 6.71 6.71 6.70 6.70 4.19 4.17 4.15 2.81 2.79 2.77 2.75 2.74 2.74 2.73 2.73 2.73 2.72 2.72 2.72 2.71 2.71 2.70 2.70 2.69 2.69 2.68 2.68 2.67 2.47 2.46 2.46 2.44 2.44 2.44 2.43 2.43 2.43 2.41 2.41 2.40 2.40 2.40 2.38 2.38 2.38 2.37 2.37 1.98 1.98 1.97 1.95 1.95 1.95 1.94 1.94 1.93 1.92 1.92 1.92 1.91 1.91 1.89 1.88 1.82 1.81 1.80 1.79 1.78 1.78 1.77 1.76 1.75 1.74 1.72
3ac
300 MHz
5.01 0.98 1.90 2.00 2.00 1.98 2.00 1.00 1.03
0.5 10.0 9.5 9.0 8.5 8.0 7.5 7.0 6.5 6.0 5.5 5.0 4.5 4.0 3.5 3.0 2.5 2.0 1.5 1.0 0.5 0.0
f1 (ppm)

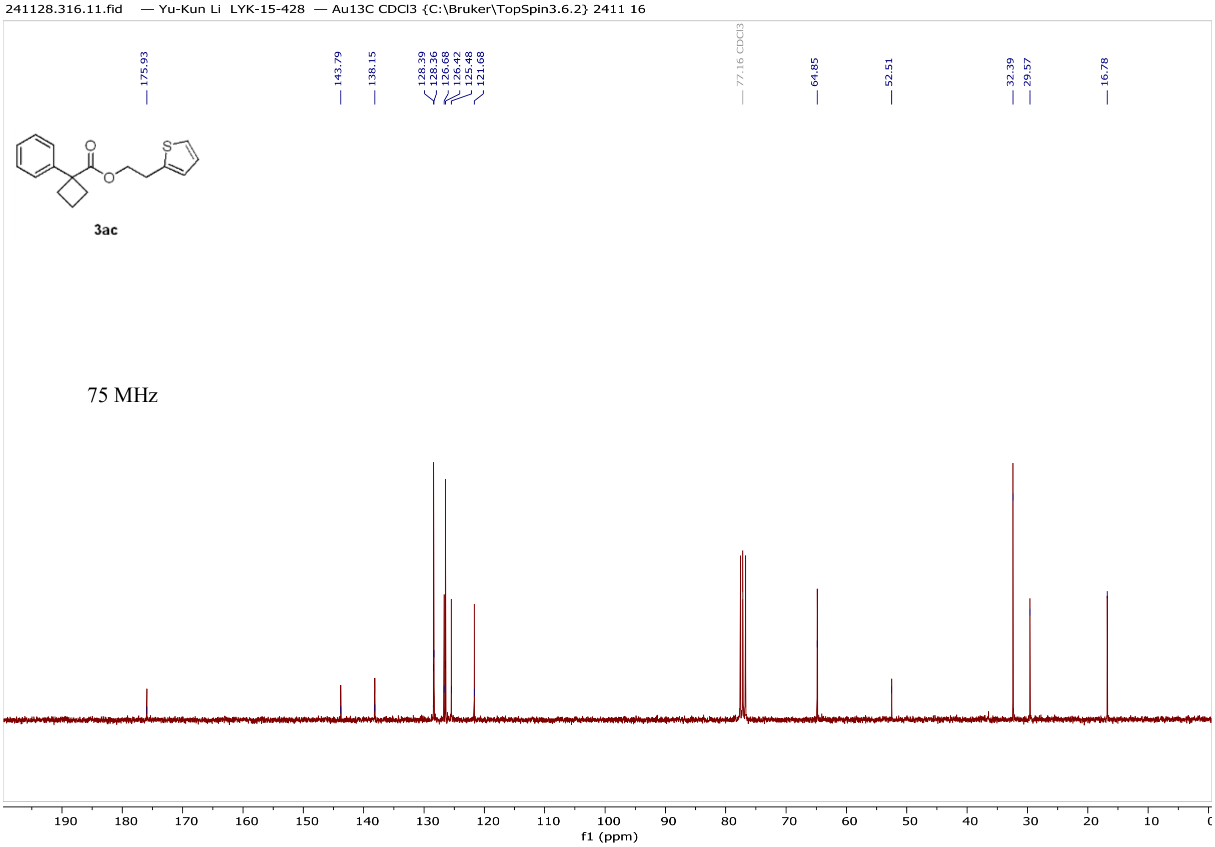
241128.316.11.fid — Yu-Kun Li LYK-15-428 — Au13C CDCl3 {C:\Bruker\TopSpin3.6.2} 2411 16
175.93 143.79 138.15 128.39 128.36 126.68 126.42 125.48 121.68 77.16 CDCl3 64.85 52.51 32.39 29.57 16.78
3ac
75 MHz
190 180 170 160 150 140 130 120 110 100 90 80 70 60 50 40 30 20 10
f1 (ppm)

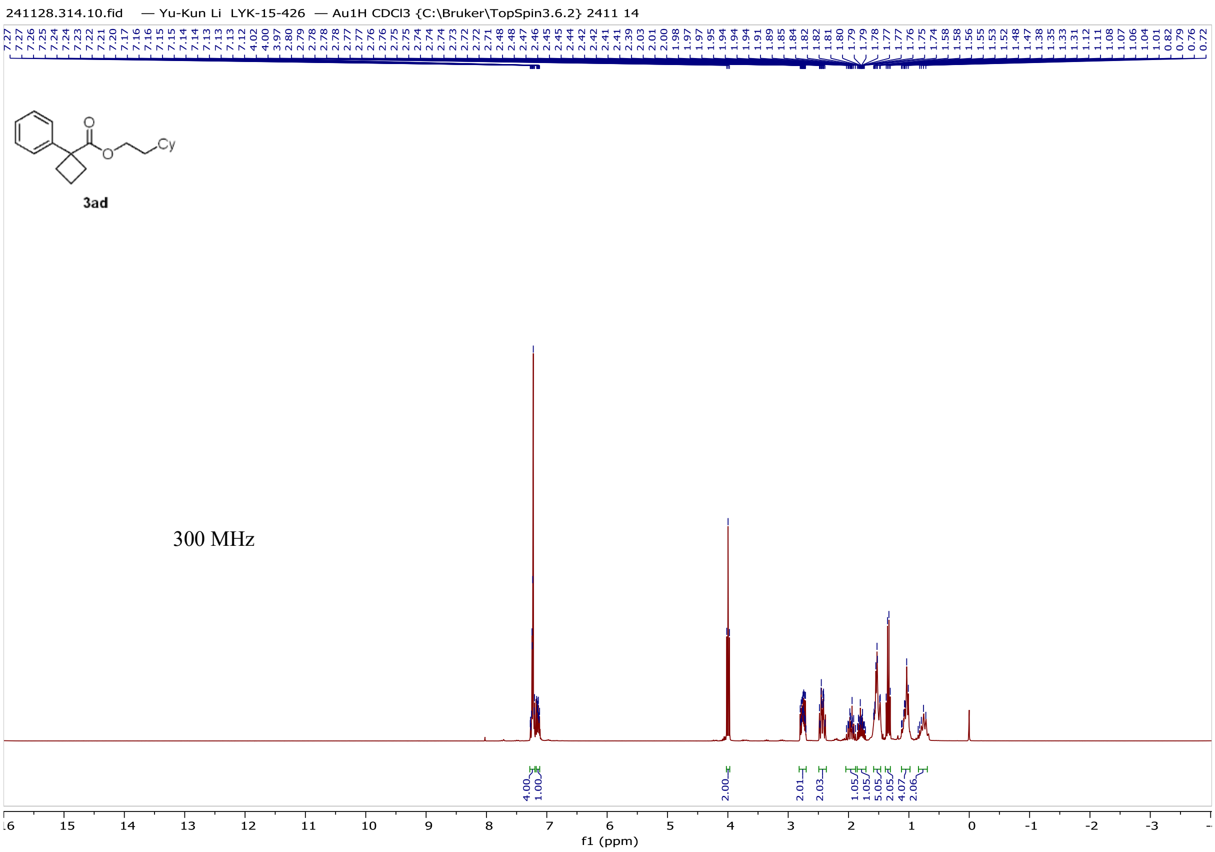
241128.314.10.fid — Yu-Kun Li LYK-15-426 — Au1H CDCl3 {C:\Bruker\TopSpin3.6.2} 2411 14
7.27 7.27 7.26 7.25 7.24 7.24 7.23 7.22 7.21 7.20 7.17 7.16 7.15 7.15 7.14 7.14 7.13 7.13 7.13 7.12 4.02 4.00 3.97 2.80 2.79 2.78 2.78 2.78 2.77 2.77 2.76 2.76 2.75 2.75 2.74 2.74 2.73 2.72 2.72 2.71 2.48 2.47 2.46 2.45 2.45 2.44 2.42 2.42 2.41 2.39 2.03 2.01 2.00 1.98 1.97 1.97 1.95 1.94 1.94 1.94 1.91 1.89 1.85 1.84 1.82 1.81 1.80 1.79 1.79 1.78 1.77 1.77 1.76 1.75 1.74 1.58 1.58 1.56 1.55 1.53 1.52 1.48 1.47 1.38 1.35 1.33 1.31 1.12 1.11 1.08 1.07 1.06 1.04 1.01 0.82 0.79 0.76 0.72
Cy
O
3ad
300 MHz
4.00 1.00 2.00 2.01 2.03 1.05 1.05 5.05 2.05 4.07 2.06
f1 (ppm)

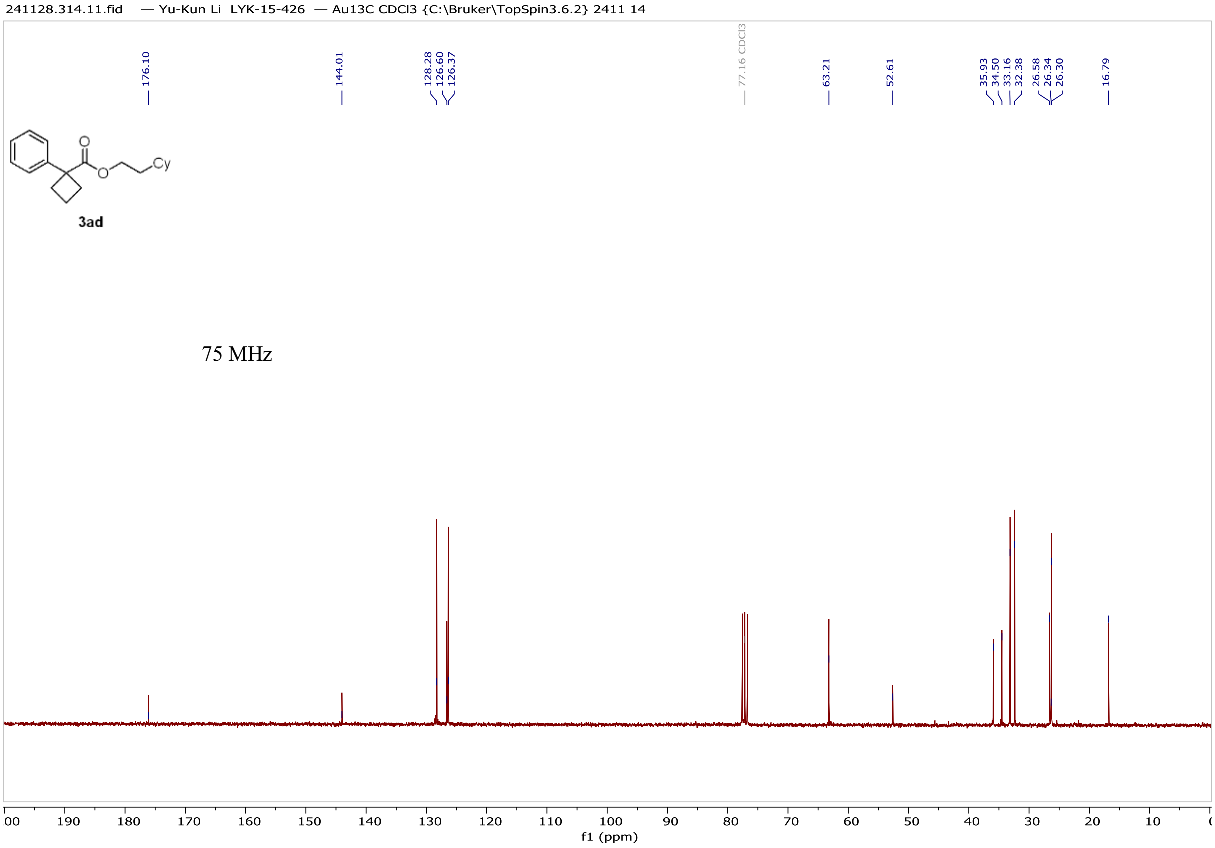
241128.314.11.fid — Yu-Kun Li LYK-15-426 — Au13C CDCl3 {C:\Bruker\TopSpin3.6.2} 2411 14
176.10
144.01
128.28 126.60 126.37
77.16 CDCl3
63.21
52.61
35.93 34.50 33.16 32.38 26.58 26.34 26.30
16.79
Cy
O
3ad
75 MHz
f1 (ppm)

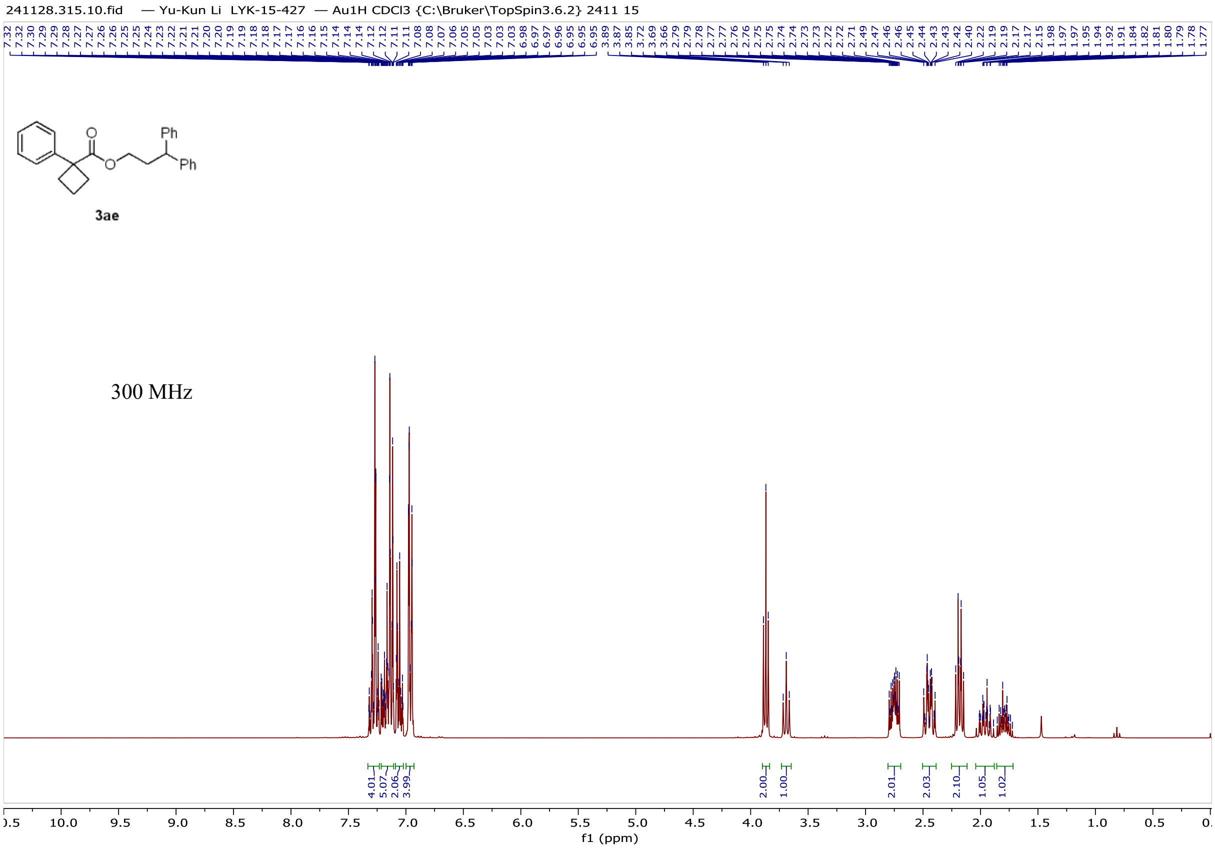

241128.315.10.fid — Yu-Kun Li LYK-15-427 — Au1H CDCl3 {C:\Bruker\TopSpin3.6.2} 2411 15
7.32 7.32 7.30 7.29 7.29 7.28 7.27 7.27 7.26 7.26 7.25 7.25 7.24 7.23 7.22 7.21 7.21 7.20 7.20 7.19 7.19 7.18 7.18 7.17 7.17 7.16 7.16 7.15 7.14 7.14 7.12 7.12 7.11 7.11 7.08 7.08 7.07 7.06 7.05 7.05 7.03 7.03 7.03 6.98 6.97 6.97 6.96 6.95 6.95 6.95
3.89 3.87 3.85 3.72 3.69 3.66 2.79 2.79 2.78 2.77 2.77 2.76 2.76 2.75 2.75 2.74 2.74 2.73 2.73 2.72 2.72 2.71 2.49 2.47 2.46 2.46 2.45 2.44 2.43 2.43 2.42 2.40 2.22 2.19 2.19 2.17 2.17 2.15 1.98 1.97 1.97 1.95 1.94 1.92 1.91 1.84 1.82 1.81 1.80 1.79 1.78 1.77
O
O
Ph
Ph
3ae
300 MHz
4.01 5.07 2.06 3.99 2.00 1.00 2.01 2.03 2.10 1.05 1.02
10.5 10.0 9.5 9.0 8.5 8.0 7.5 7.0 6.5 6.0 5.5 5.0 4.5 4.0 3.5 3.0 2.5 2.0 1.5 1.0 0.5 0
f1 (ppm)

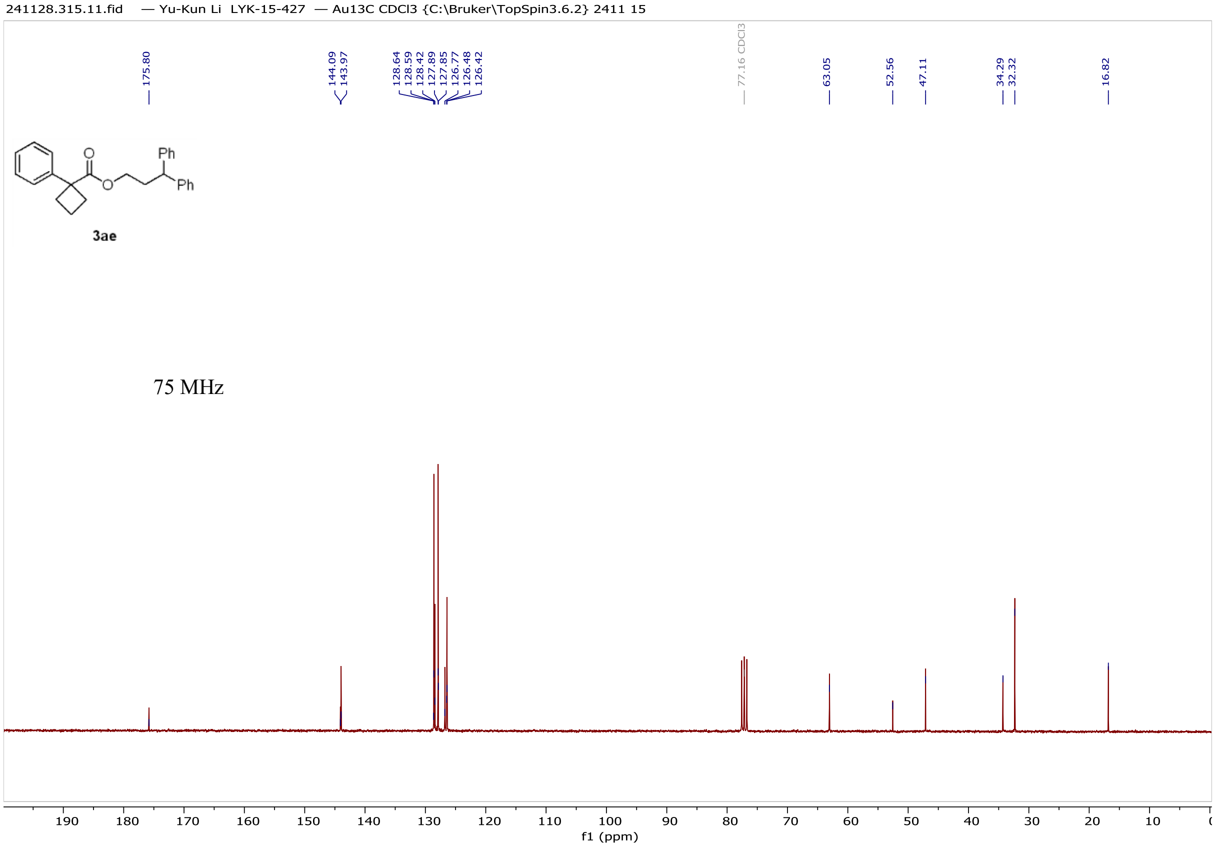

241128.315.11.fid — Yu-Kun Li LYK-15-427 — Au13C CDCl3 {C:\Bruker\TopSpin3.6.2} 2411 15
175.80
144.09 143.97
128.64 128.59 128.42 127.89 127.85 126.77 126.48 126.42
77.16 CDCl3
63.05
52.56
47.11
34.29 32.32
16.82
O
O
Ph
Ph
3ae
75 MHz
190 180 170 160 150 140 130 120 110 100 90 80 70 60 50 40 30 20 10 0
f1 (ppm)

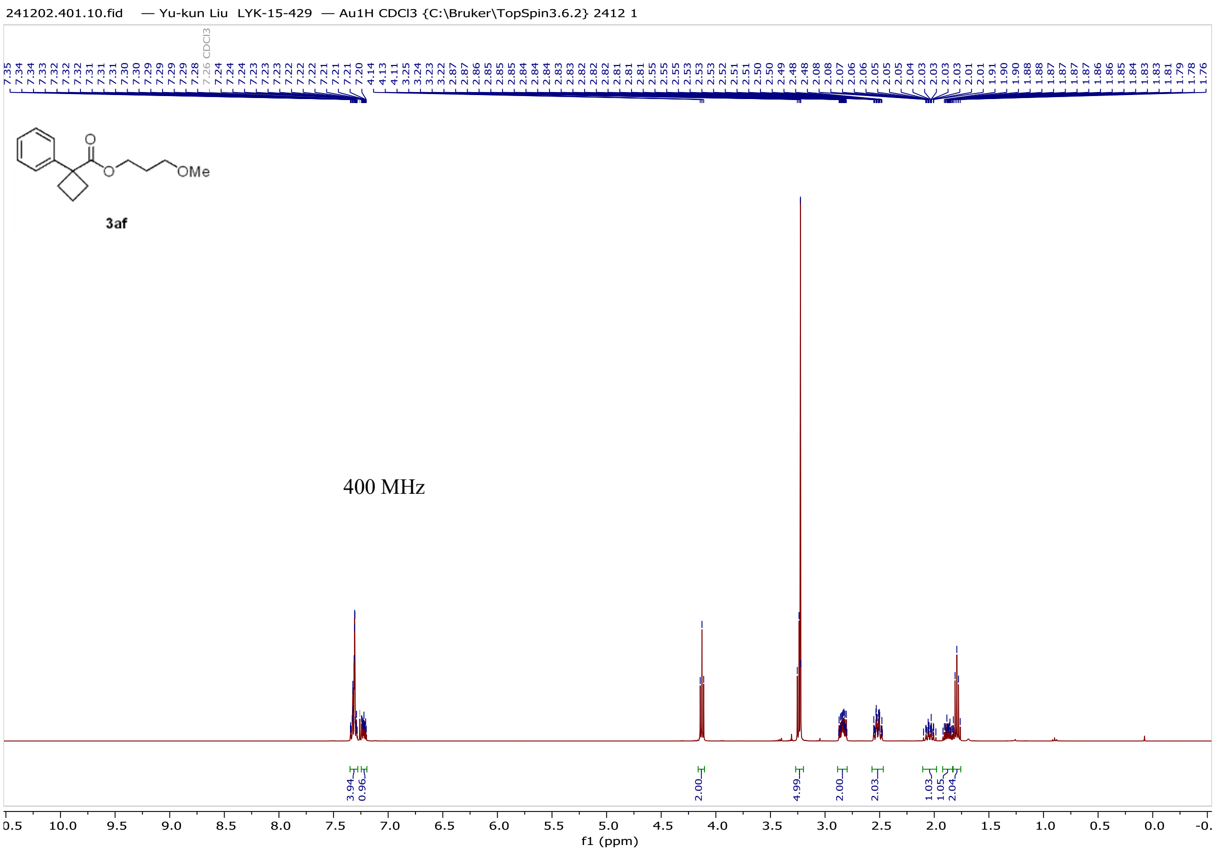
241202.401.10.fid — Yu-kun Liu LYK-15-429 — Au1H CDCl3 {C:\Bruker\TopSpin3.6.2} 2412 1
OMe
3af
400 MHz
f1 (ppm)

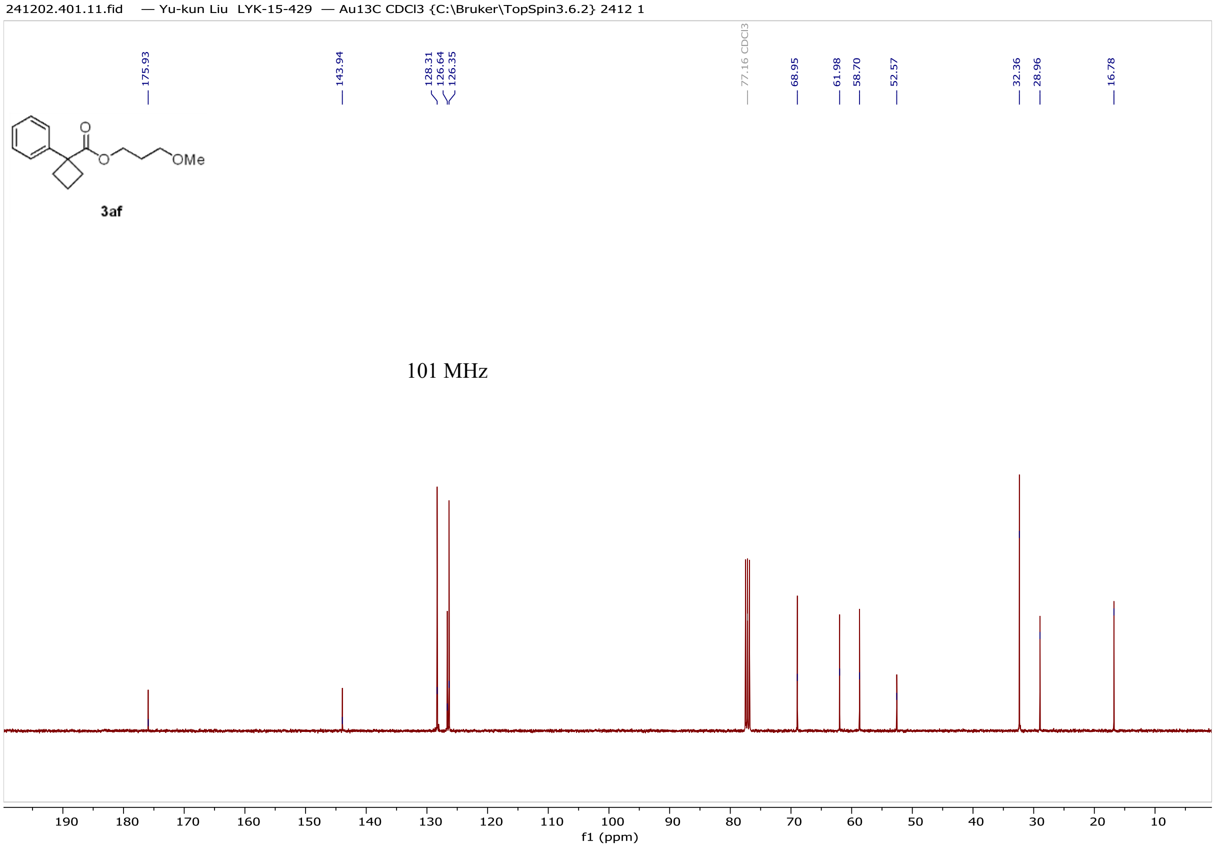
241202.401.11.fid — Yu-kun Liu LYK-15-429 — Au13C CDCl3 {C:\Bruker\TopSpin3.6.2} 2412 1
175.93
143.94
128.31
126.64
126.35
77.16 CDCl3
68.95
61.98
58.70
52.57
32.36
28.96
16.78
OMe
3af
101 MHz
f1 (ppm)

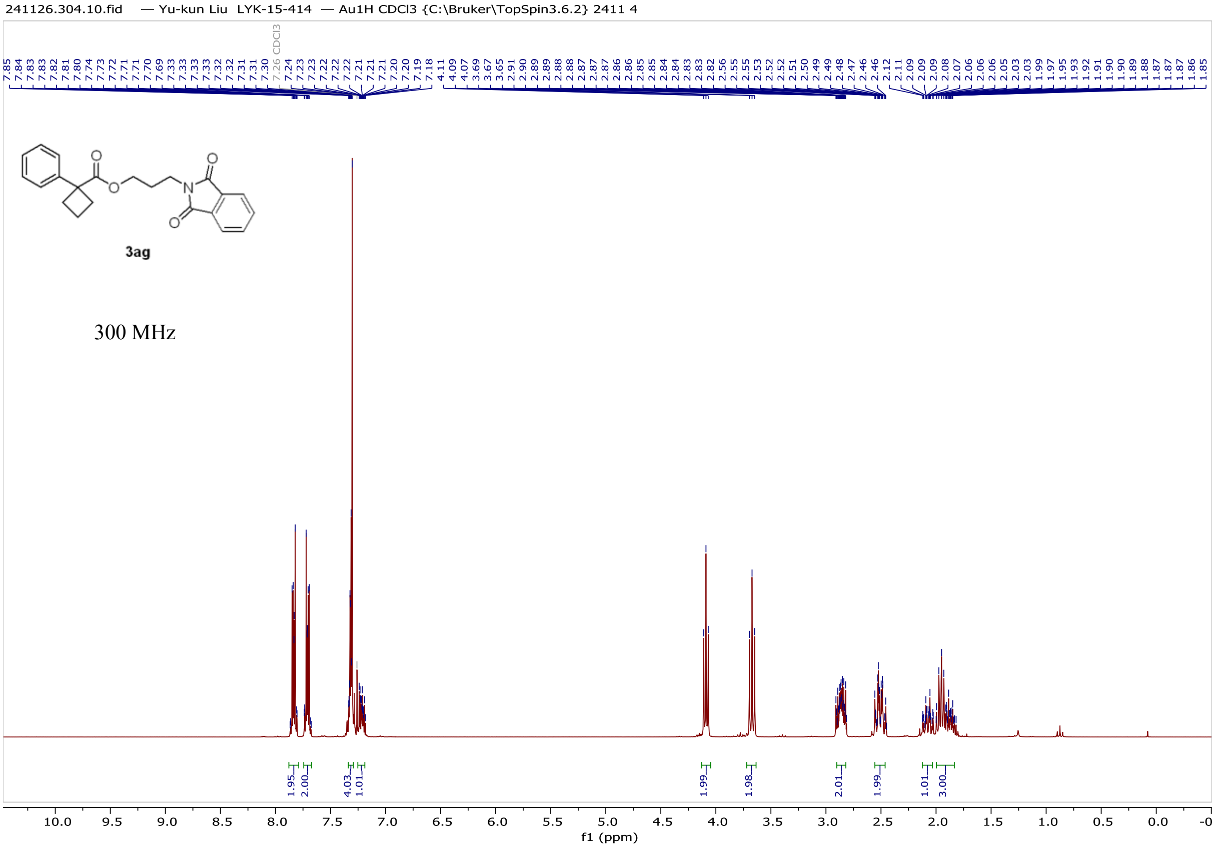
241126.304.10.fid — Yu-kun Liu LYK-15-414 — Au1H CDCl3 {C:\Bruker\TopSpin3.6.2} 2411 4
7.85 7.84 7.83 7.83 7.82 7.81 7.80 7.74 7.73 7.72 7.71 7.71 7.70 7.69 7.33 7.33 7.33 7.33 7.32 7.32 7.31 7.31 7.30 7.26 CDCl3 7.24 7.23 7.23 7.22 7.22 7.22 7.21 7.21 7.21 7.20 7.20 7.19 7.18 4.11 4.09 4.07 3.69 3.67 3.65 2.91 2.90 2.89 2.89 2.88 2.88 2.87 2.87 2.86 2.86 2.85 2.85 2.84 2.84 2.83 2.83 2.82 2.56 2.55 2.55 2.53 2.52 2.52 2.51 2.50 2.49 2.49 2.48 2.47 2.46 2.46 2.12 2.11 2.09 2.09 2.09 2.08 2.07 2.06 2.06 2.05 2.03 1.99 1.97 1.95 1.93 1.92 1.91 1.90 1.90 1.89 1.88 1.87 1.87 1.86 1.85
3ag
300 MHz
1.95 2.00 4.03 1.01 1.99 1.98 2.01 1.99 1.01 3.00
10.0 9.5 9.0 8.5 8.0 7.5 7.0 6.5 6.0 5.5 5.0 4.5 4.0 3.5 3.0 2.5 2.0 1.5 1.0 0.5 0.0
f1 (ppm)

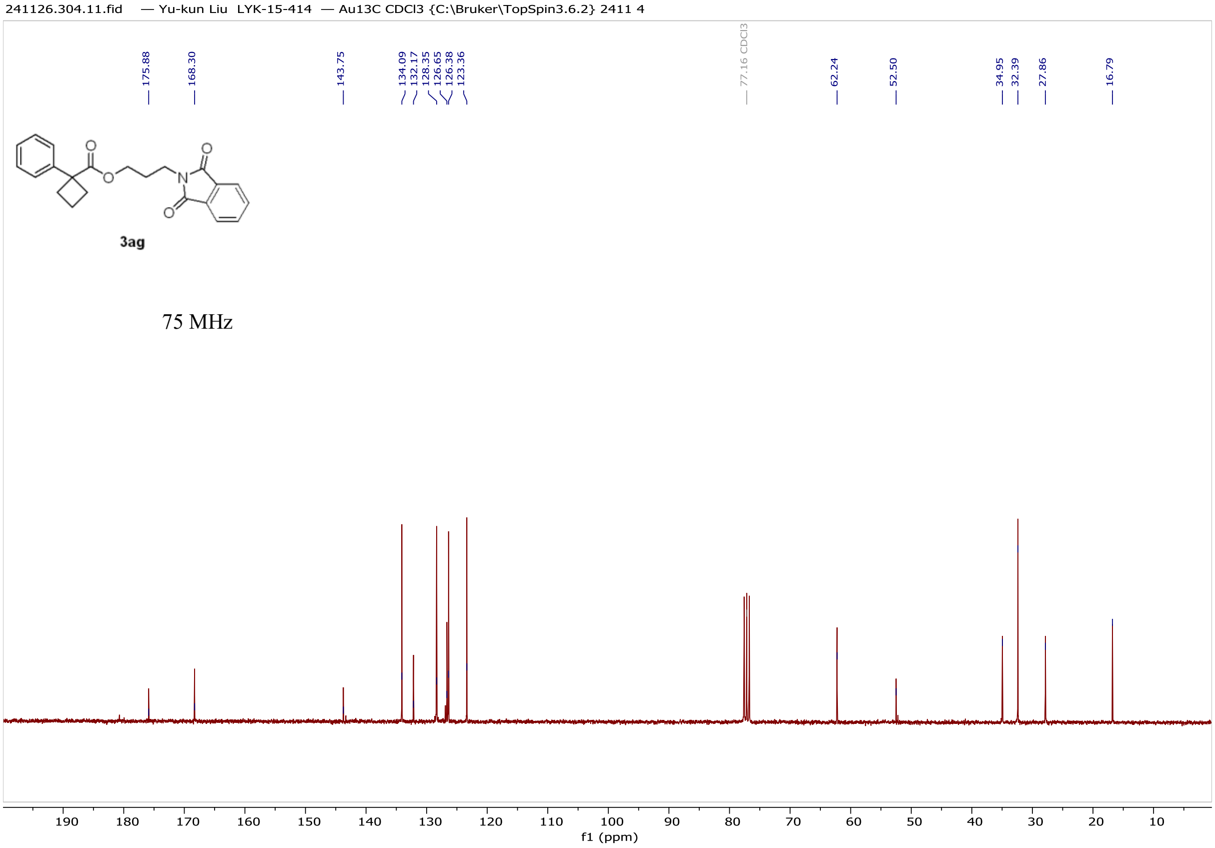
241126.304.11.fid — Yu-kun Liu LYK-15-414 — Au13C CDCl3 {C:\Bruker\TopSpin3.6.2} 2411 4
175.88 168.30 143.75 134.09 132.17 128.35 126.65 126.38 123.36 77.16 CDCl3 62.24 52.50 34.95 32.39 27.86 16.79
3ag
75 MHz
190 180 170 160 150 140 130 120 110 100 90 80 70 60 50 40 30 20 10
f1 (ppm)

241127.308.10.fid — Yu-kun Liu LYK-15-420 — Au1H CDCl3 {C:\Bruker\TopSpin3.6.2} 2411 8

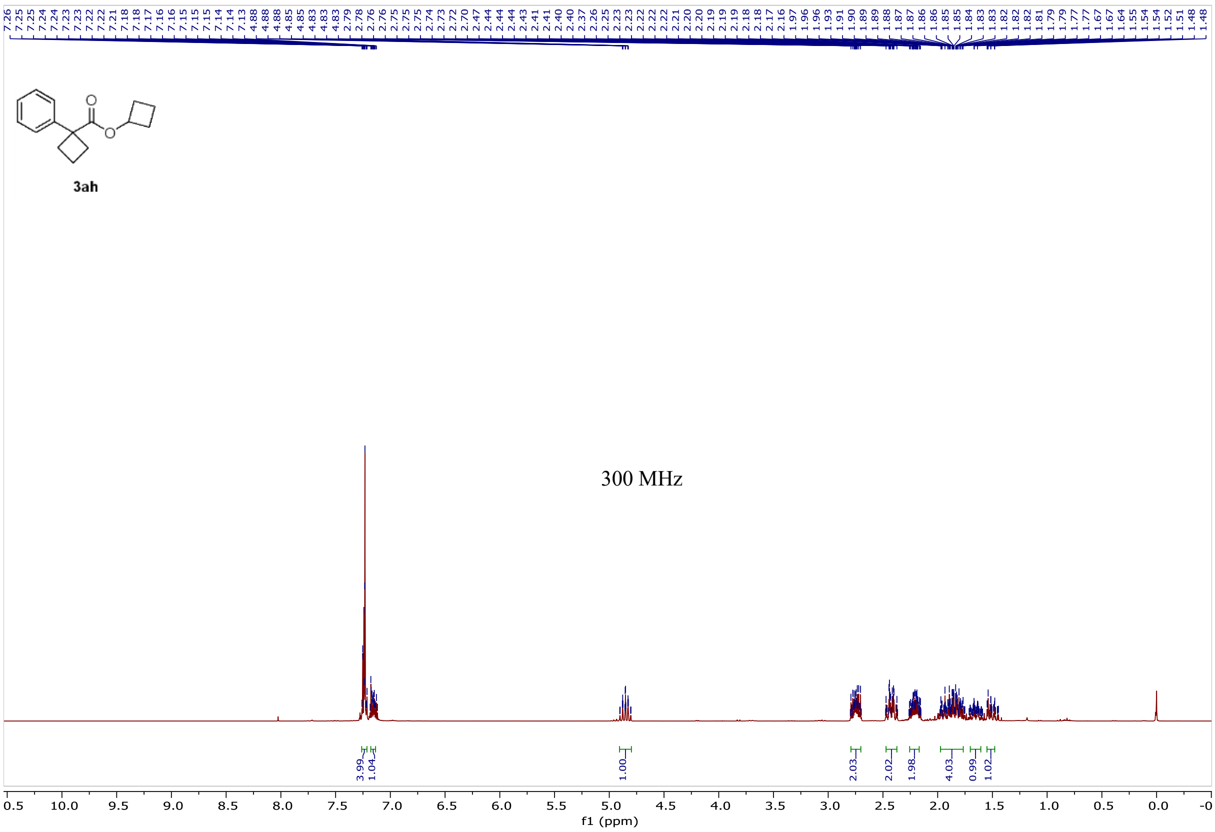

241127.308.11.fid — Yu-kun Liu LYK-15-420 — Au13C CDCl3 {C:\Bruker\TopSpin3.6.2} 2411 8

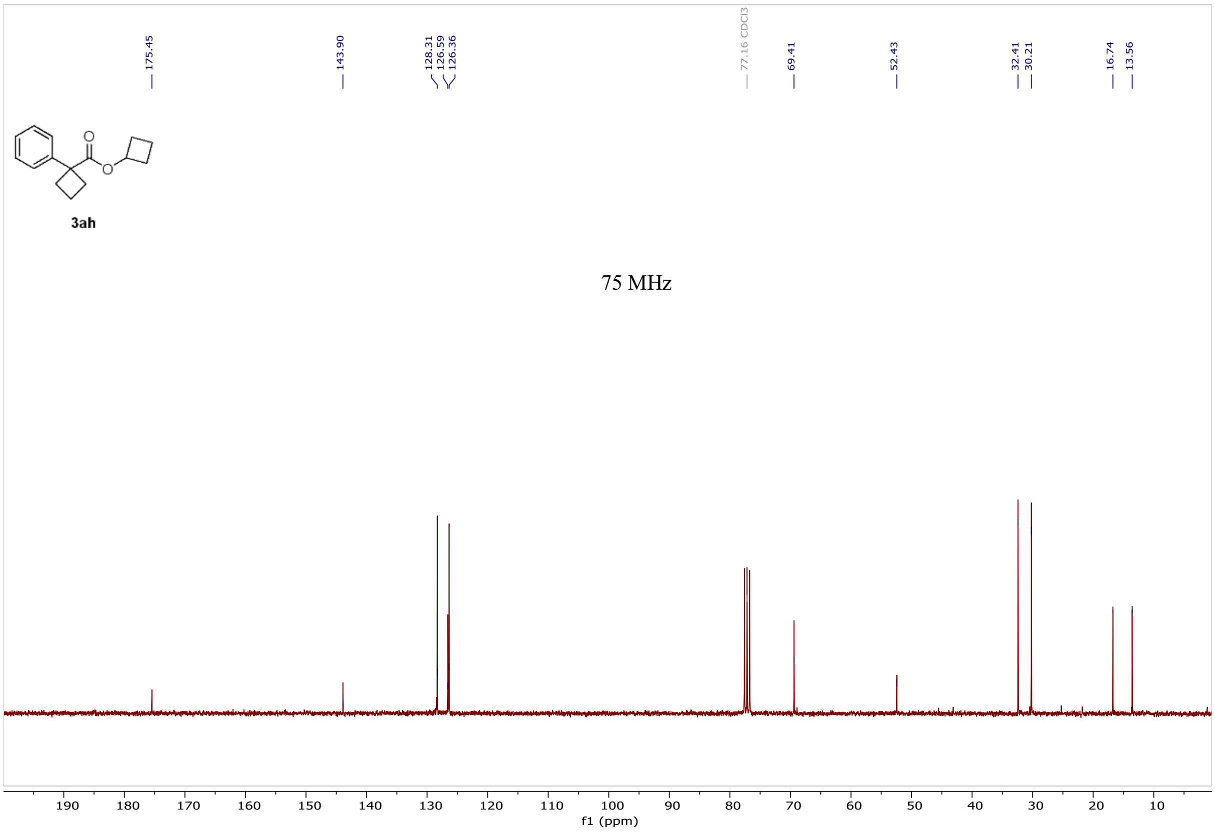

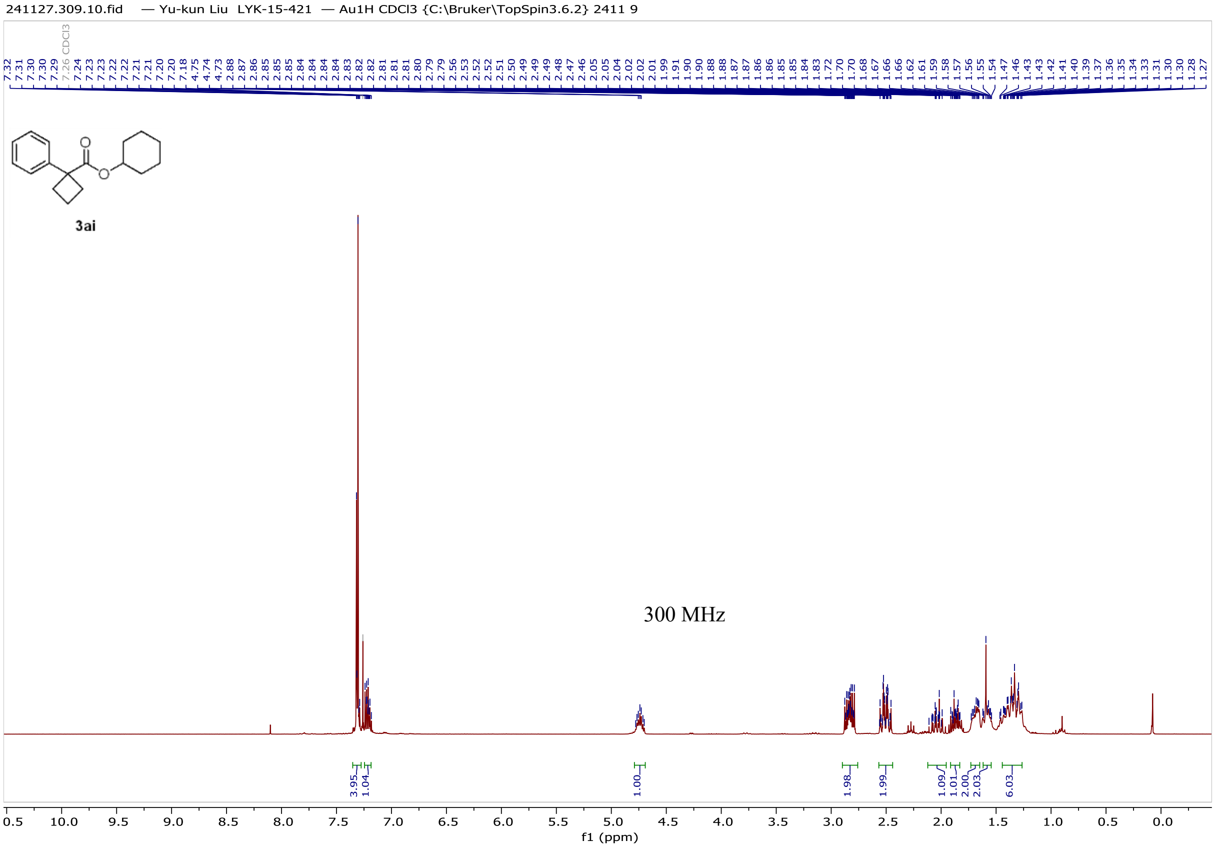
241127.309.10.fid — Yu-kun Liu LYK-15-421 — Au1H CDCl3 {C:\Bruker\TopSpin3.6.2} 2411 9
7.32 7.31 7.30 7.29 7.26 CDCl3 7.24 7.23 7.23 7.22 7.22 7.21 7.21 7.20 7.20 7.18 4.75 4.74 4.73 2.88 2.87 2.86 2.85 2.85 2.84 2.84 2.84 2.84 2.83 2.82 2.82 2.82 2.81 2.81 2.80 2.79 2.79 2.56 2.53 2.52 2.52 2.51 2.50 2.49 2.49 2.49 2.48 2.47 2.46 2.05 2.04 2.02 2.02 2.01 1.99 1.91 1.90 1.90 1.88 1.88 1.87 1.86 1.86 1.85 1.85 1.84 1.83 1.72 1.70 1.68 1.67 1.66 1.66 1.62 1.61 1.59 1.58 1.57 1.56 1.55 1.54 1.47 1.46 1.43 1.43 1.42 1.41 1.40 1.39 1.37 1.36 1.35 1.34 1.33 1.31 1.30 1.28 1.27
3ai
300 MHz
3.95 1.04 1.00 1.98 1.99 1.09 1.01 2.00 2.03 6.03
0.5 10.0 9.5 9.0 8.5 8.0 7.5 7.0 6.5 6.0 5.5 5.0 4.5 4.0 3.5 3.0 2.5 2.0 1.5 1.0 0.5 0.0
f1 (ppm)

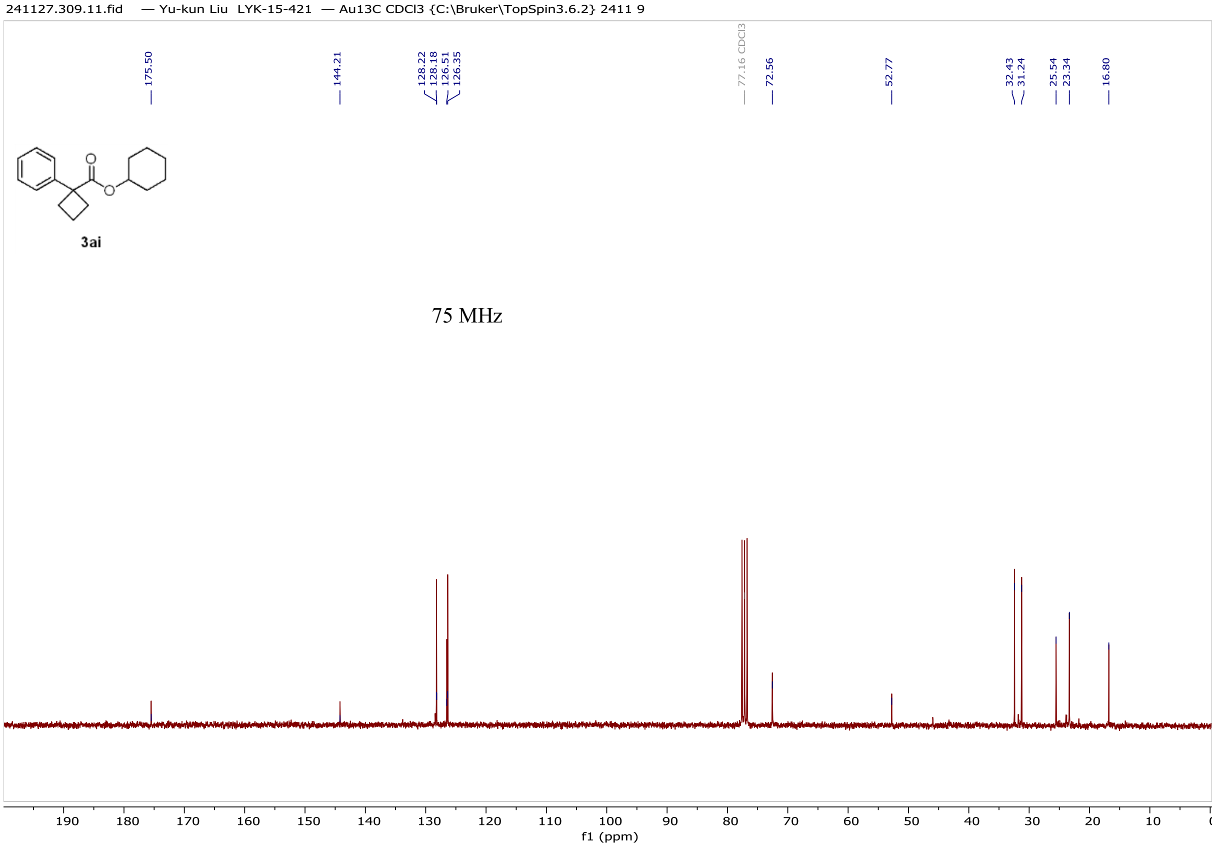
241127.309.11.fid — Yu-kun Liu LYK-15-421 — Au13C CDCl3 {C:\Bruker\TopSpin3.6.2} 2411 9
175.50
144.21
128.22 128.18 126.51 126.35
77.16 CDCl3
72.56
52.77
32.43 31.24
25.54 23.34
16.80
3ai
75 MHz
190 180 170 160 150 140 130 120 110 100 90 80 70 60 50 40 30 20 10
f1 (ppm)

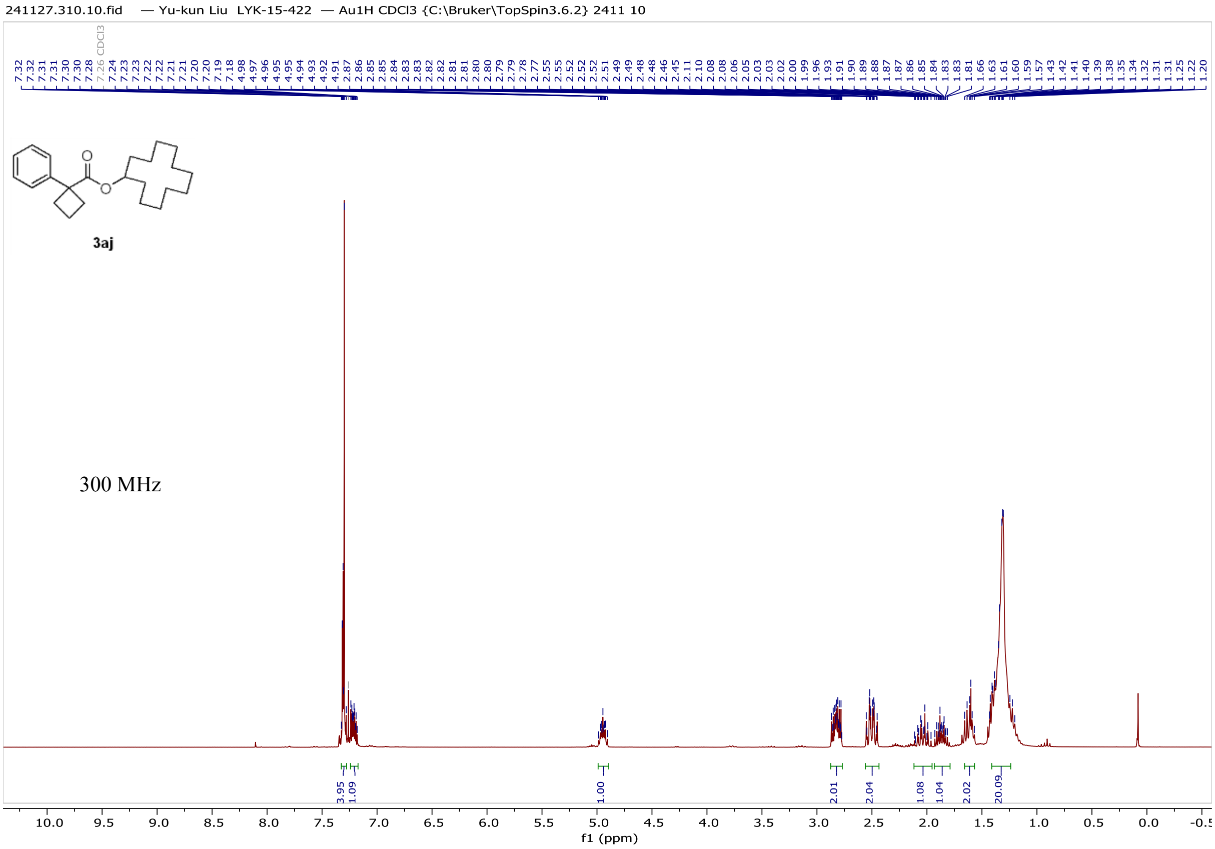
241127.310.10.fid — Yu-kun Liu LYK-15-422 — Au1H CDCl3 {C:\Bruker\TopSpin3.6.2} 2411 10
7.32 7.32 7.31 7.31 7.30 7.30 7.28 7.26 CDCl3 7.24 7.23 7.23 7.22 7.22 7.21 7.21 7.20 7.20 7.19 7.18 4.98 4.97 4.96 4.95 4.95 4.94 4.93 4.92 4.91 2.87 2.86 2.85 2.85 2.84 2.83 2.83 2.82 2.82 2.81 2.81 2.80 2.80 2.79 2.79 2.78 2.77 2.55 2.55 2.52 2.52 2.51 2.49 2.49 2.48 2.48 2.46 2.45 2.11 2.10 2.08 2.08 2.06 2.05 2.03 2.03 2.02 2.00 1.99 1.96 1.93 1.91 1.90 1.89 1.88 1.87 1.87 1.86 1.85 1.84 1.83 1.83 1.81 1.66 1.63 1.61 1.60 1.59 1.57 1.43 1.42 1.41 1.40 1.39 1.38 1.35 1.34 1.32 1.31 1.25 1.22 1.20
3aj
300 MHz
3.95 1.09 1.00 2.01 2.04 1.08 1.04 2.02 20.09
10.0 9.5 9.0 8.5 8.0 7.5 7.0 6.5 6.0 5.5 5.0 4.5 4.0 3.5 3.0 2.5 2.0 1.5 1.0 0.5 0.0 -0.5
f1 (ppm)

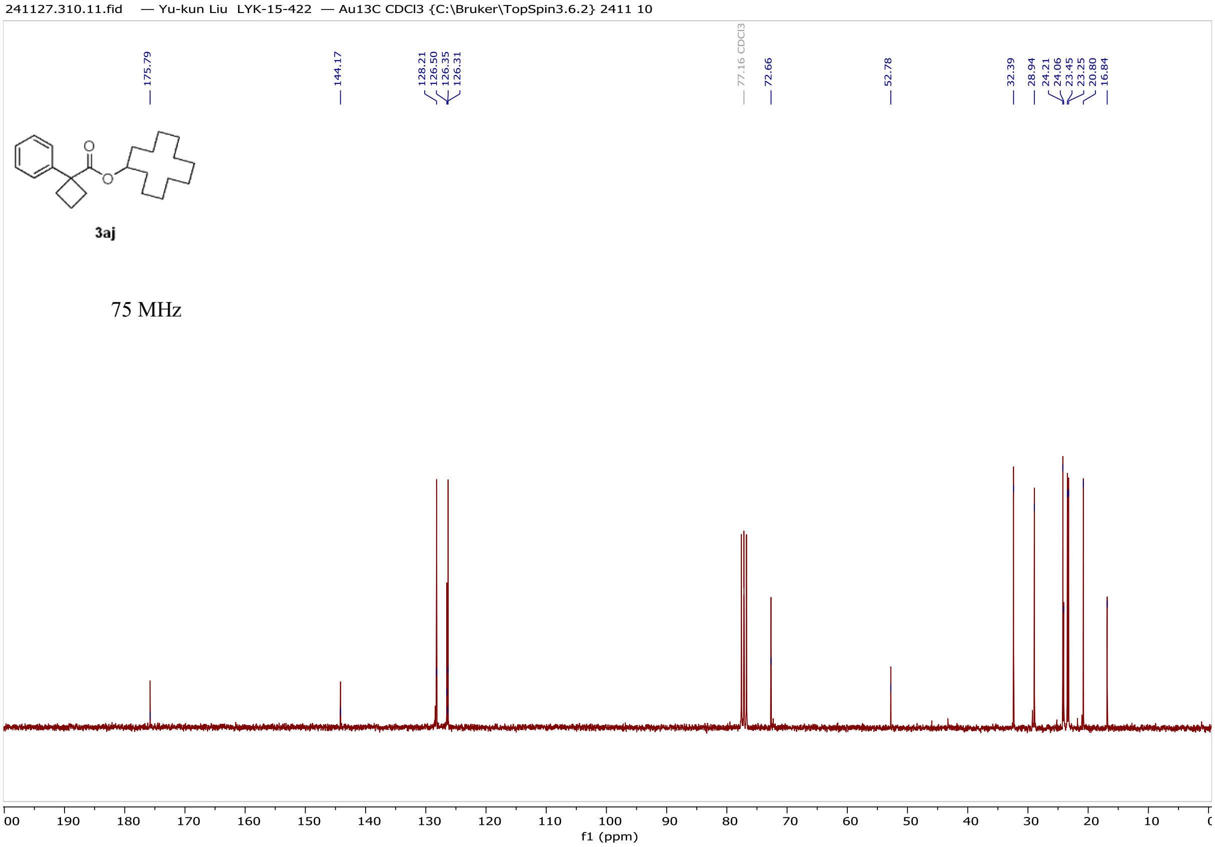
241127.310.11.fid — Yu-kun Liu LYK-15-422 — Au13C CDCl3 {C:\Bruker\TopSpin3.6.2} 2411 10
175.79
144.17
128.21 126.50 126.35 126.31
77.16 CDCl3
72.66
52.78
32.39 28.94 24.21 24.06 23.45 23.25 20.80 16.84
3aj
75 MHz
200 190 180 170 160 150 140 130 120 110 100 90 80 70 60 50 40 30 20 10 0
f1 (ppm)

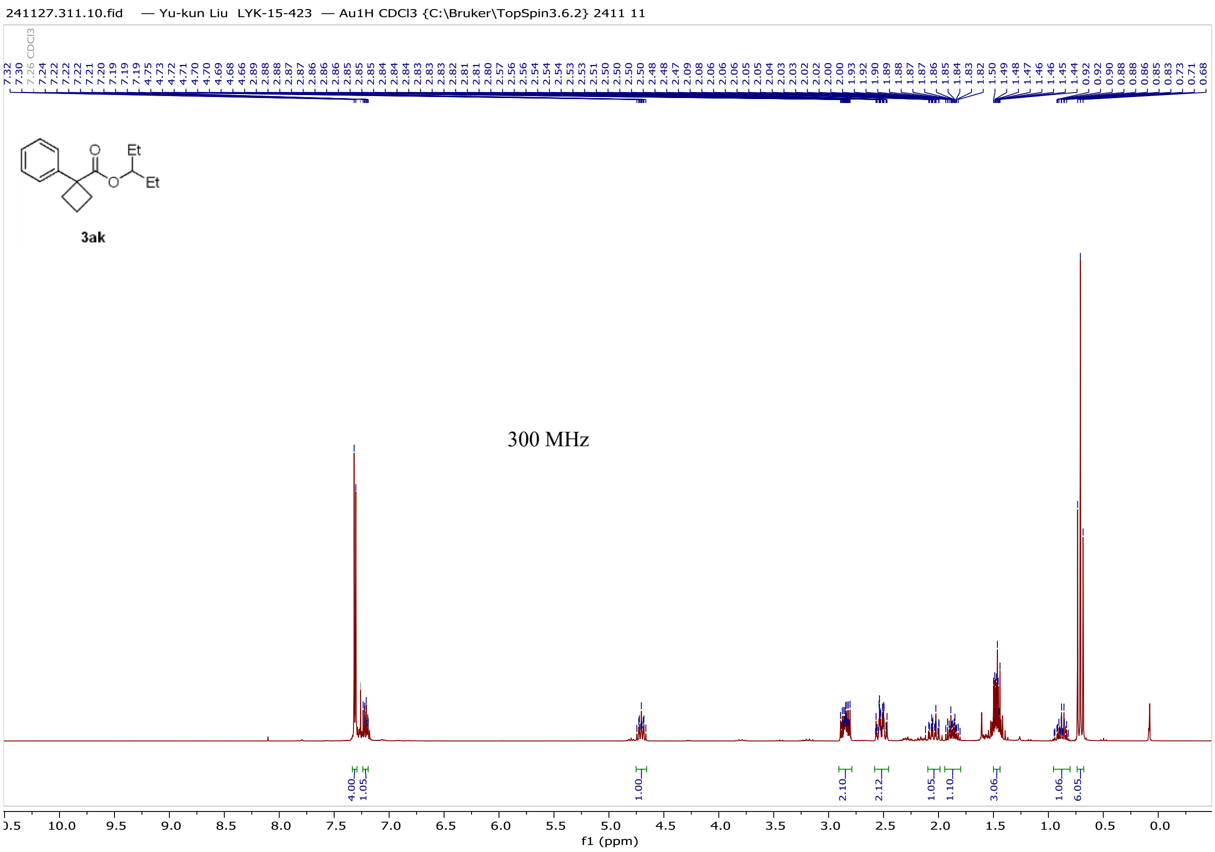
241127.311.10.fid — Yu-kun Liu LYK-15-423 — Au1H CDCl3 {C:\Bruker\TopSpin3.6.2} 2411 11
Et
O
O
Et
3ak
300 MHz
4.00
1.05
1.00
2.10
2.12
1.05
1.10
3.06
1.06
6.05
f1 (ppm)

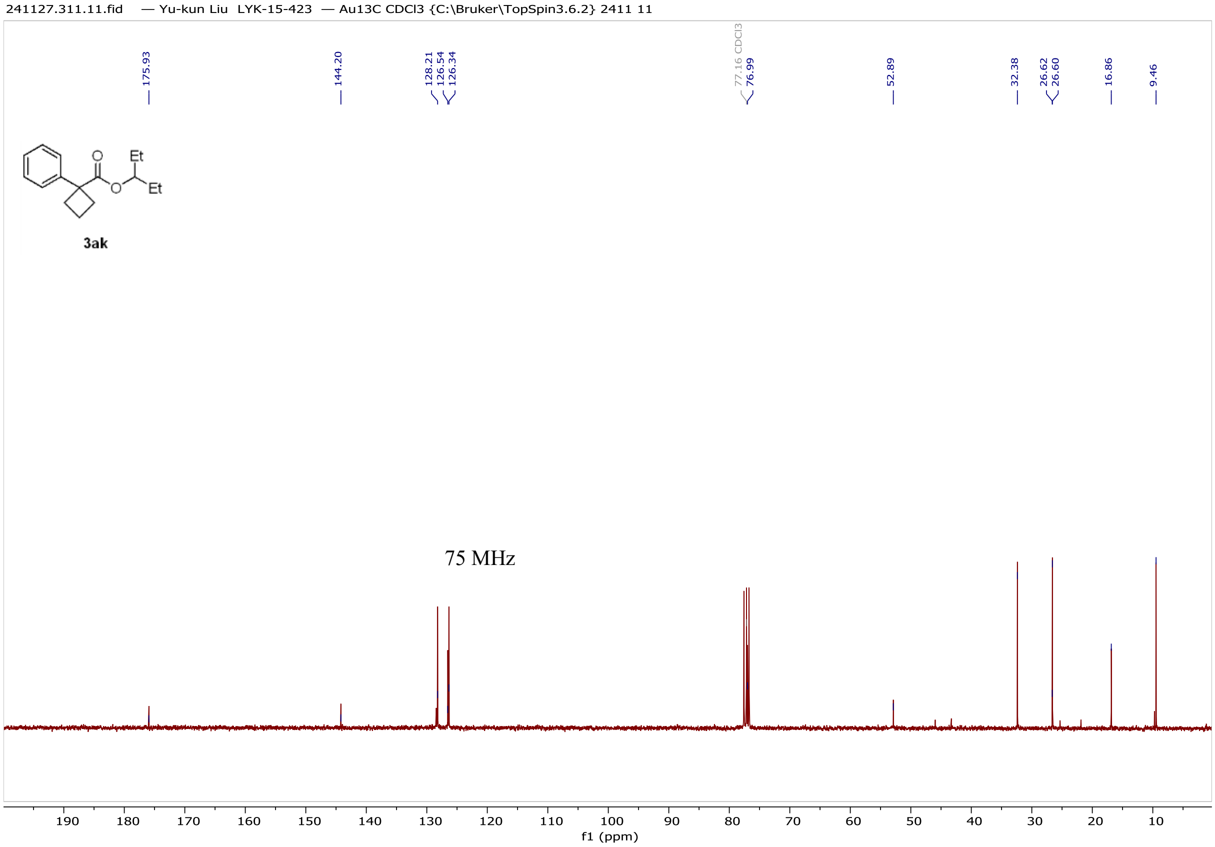
241127.311.11.fid — Yu-kun Liu LYK-15-423 — Au13C CDCl3 {C:\Bruker\TopSpin3.6.2} 2411 11
175.93
144.20
128.21
126.54
126.34
77.16 CDCl3
76.99
52.89
32.38
26.62
26.60
16.86
9.46
Et
O
O
Et
3ak
75 MHz
f1 (ppm)

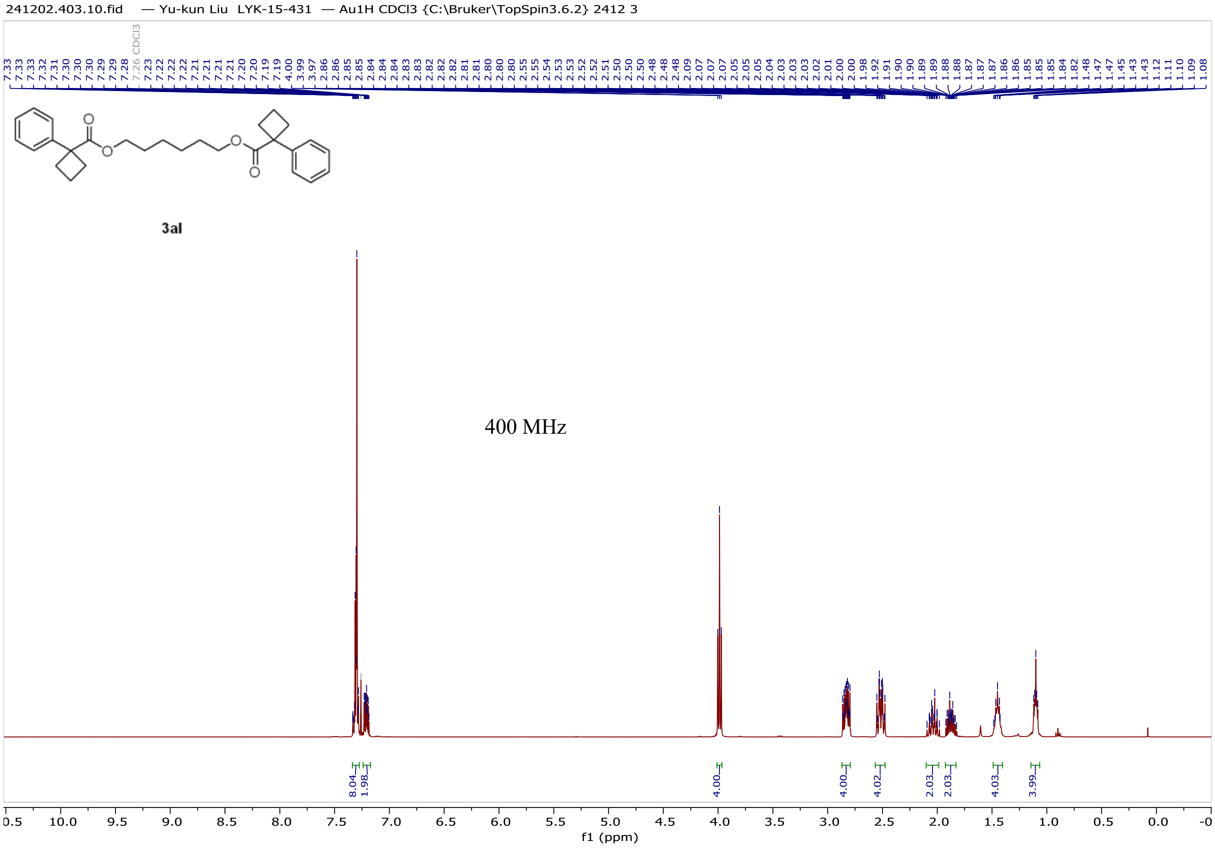
241202.403.10.fid — Yu-kun Liu LYK-15-431 — Au1H CDCl3 {C:\Bruker\TopSpin3.6.2} 2412 3
7.33 7.33 7.32 7.31 7.30 7.30 7.29 7.29 7.28 7.26 CDCl3 7.23 7.22 7.22 7.22 7.21 7.21 7.21 7.20 7.19 7.19 4.00 3.99 3.97 2.86 2.86 2.85 2.85 2.84 2.84 2.83 2.83 2.82 2.82 2.82 2.81 2.81 2.80 2.80 2.55 2.55 2.54 2.53 2.53 2.52 2.51 2.50 2.50 2.50 2.48 2.48 2.48 2.09 2.07 2.07 2.07 2.05 2.05 2.05 2.04 2.03 2.03 2.03 2.02 2.01 2.00 2.00 1.98 1.92 1.91 1.90 1.90 1.89 1.89 1.88 1.88 1.87 1.87 1.87 1.86 1.85 1.85 1.85 1.84 1.82 1.48 1.47 1.45 1.43 1.43 1.12 1.11 1.10 1.09 1.08
3al
400 MHz
8.04 1.98 4.00 4.00 4.02 2.03 2.03 4.03 3.99
f1 (ppm)

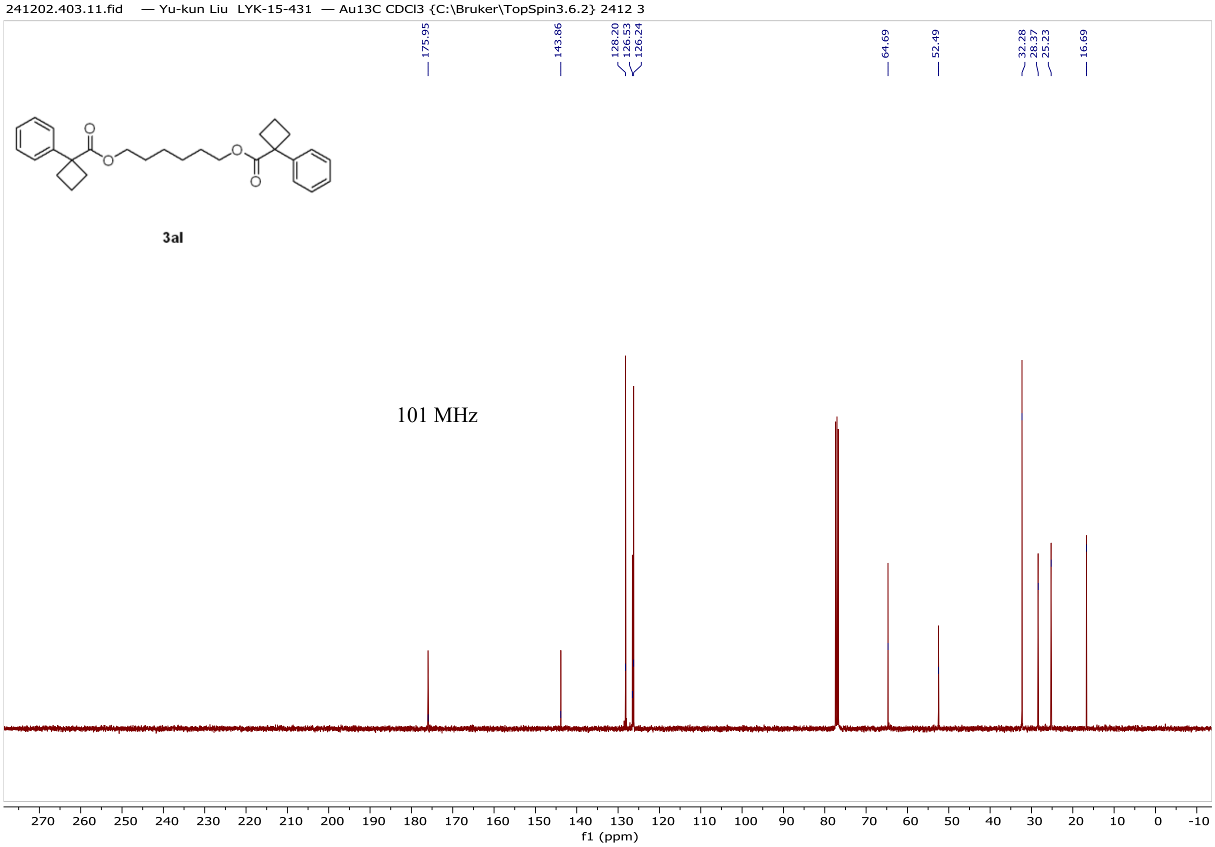
241202.403.11.fid — Yu-kun Liu LYK-15-431 — Au13C CDCl3 {C:\Bruker\TopSpin3.6.2} 2412 3
175.95 143.86 128.20 126.53 126.24 64.69 52.49 32.28 28.37 25.23 16.69
3al
101 MHz
f1 (ppm)

241202.404.10.fid — Yu-kun Liu LYK-15-432 — Au1H CDCl3 {C:\Bruker\TopSpin3.6.2} 2412 4

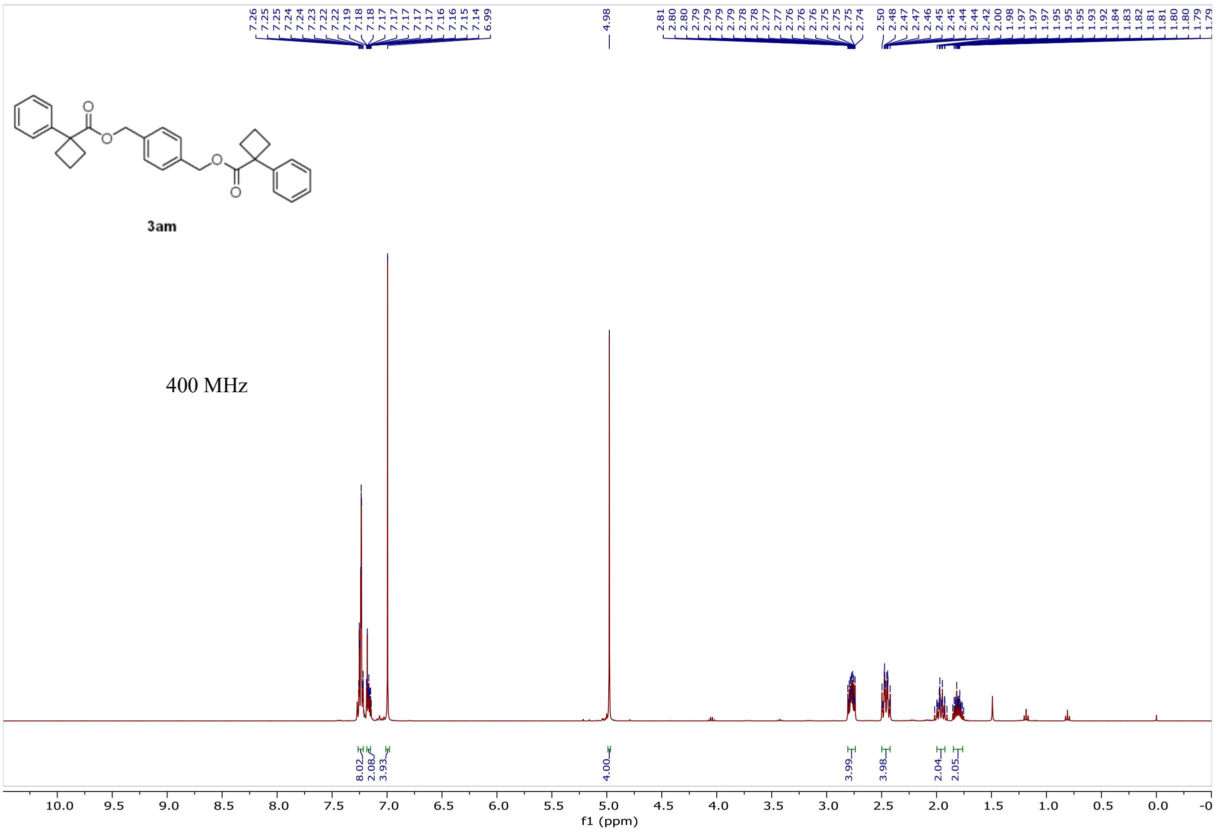

241202.404.11.fid — Yu-kun Liu LYK-15-432 — Au13C CDCl3 {C:\Bruker\TopSpin3.6.2} 2412 4

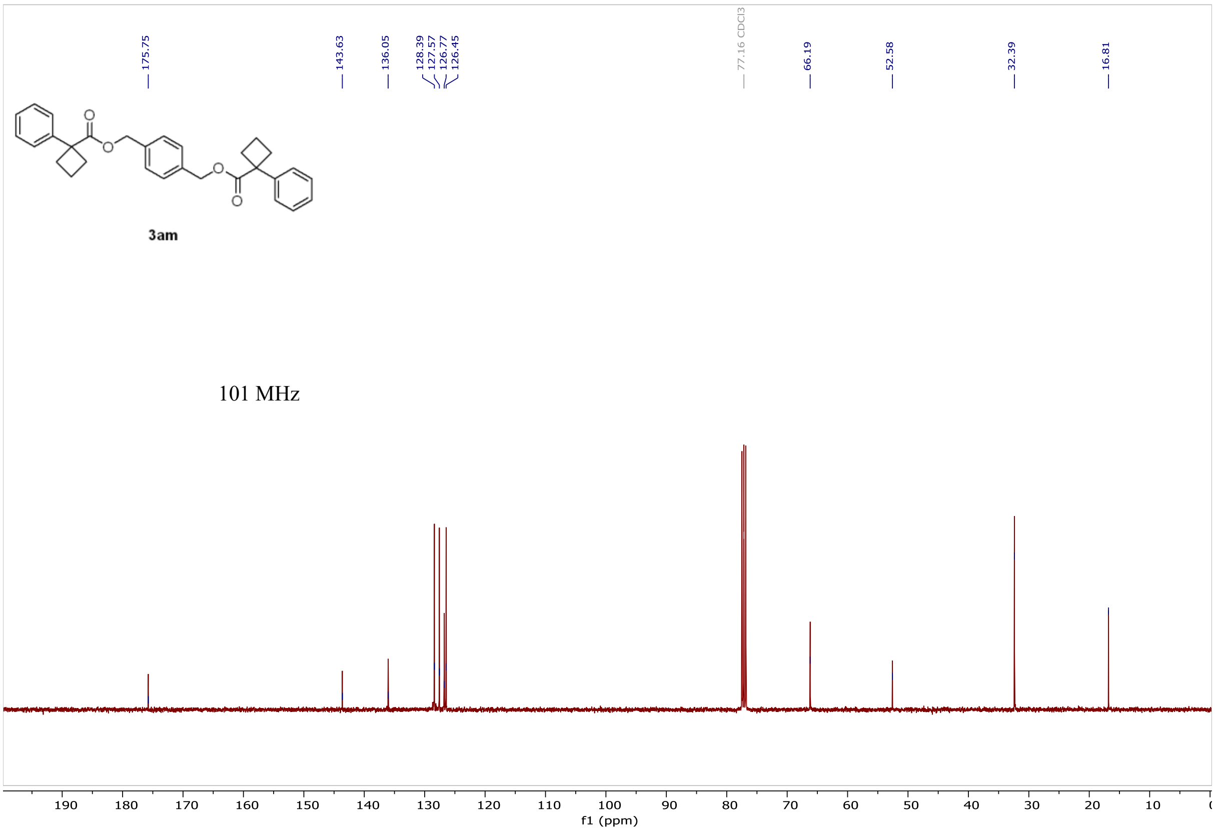

241202.406.10.fid — Yu-kun Liu LYK-15-434 — Au1H CDCl3 {C:\Bruker\TopSpin3.6.2} 2412 6

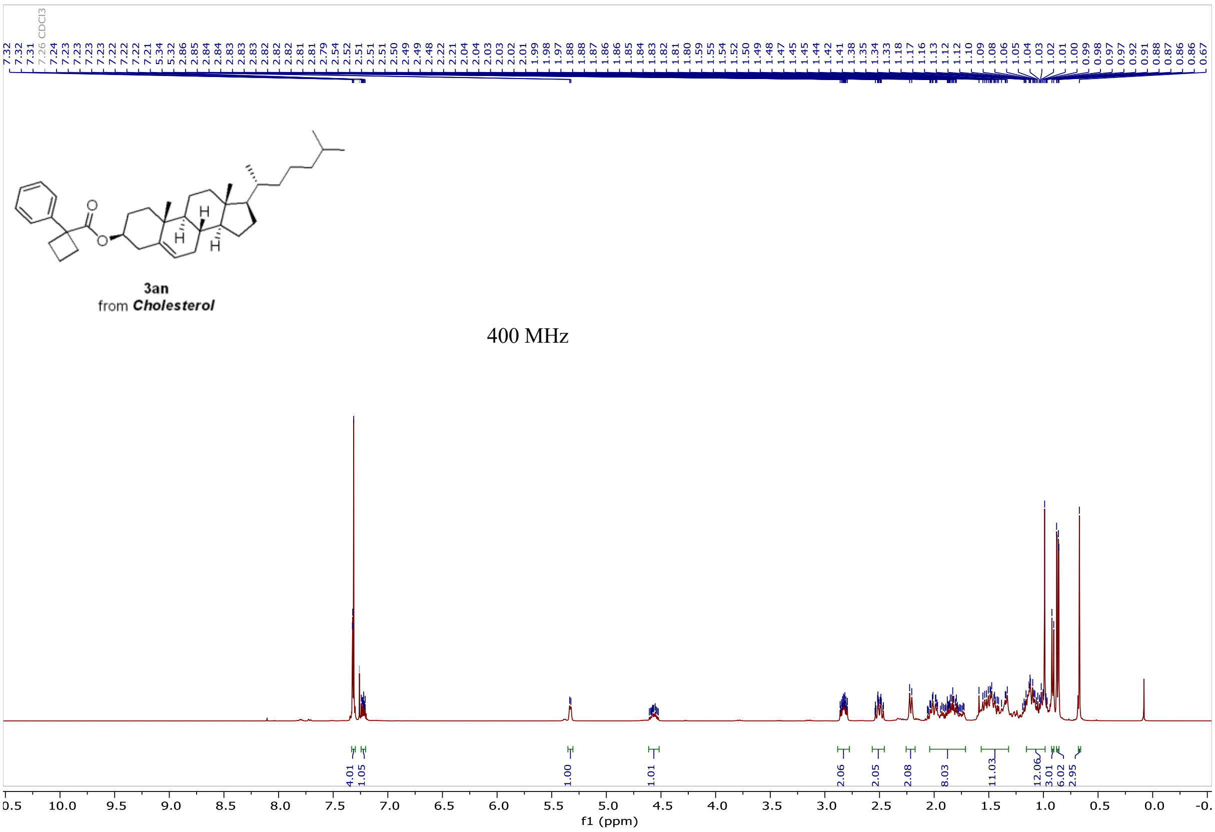

241202.406.11.fid — Yu-kun Liu LYK-15-434 — Au13C CDCl3 {C:\Bruker\TopSpin3.6.2} 2412 6

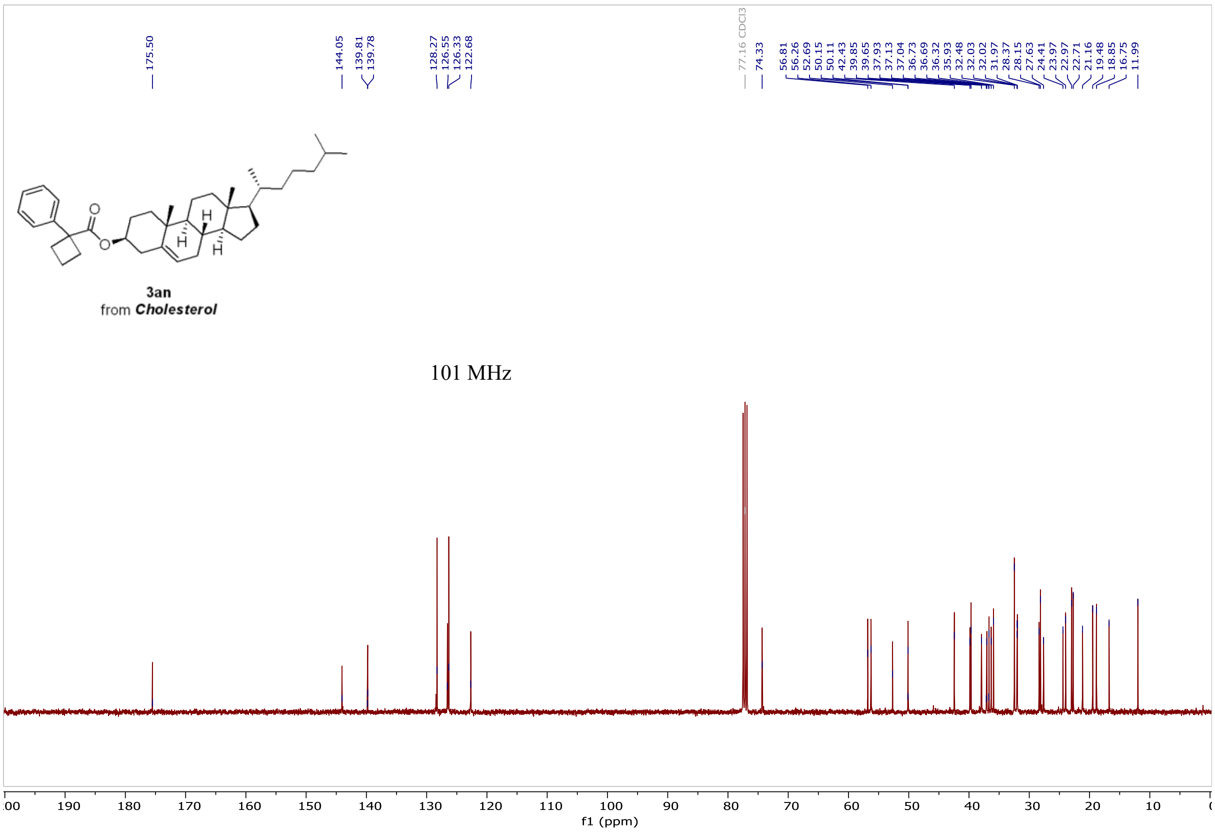

241218.f316.10.fid — Yu-kun Liu LYK-15-450-re — Au1H CDCl3 {C:\Bruker\TopSpin3.6.2} 2412 16

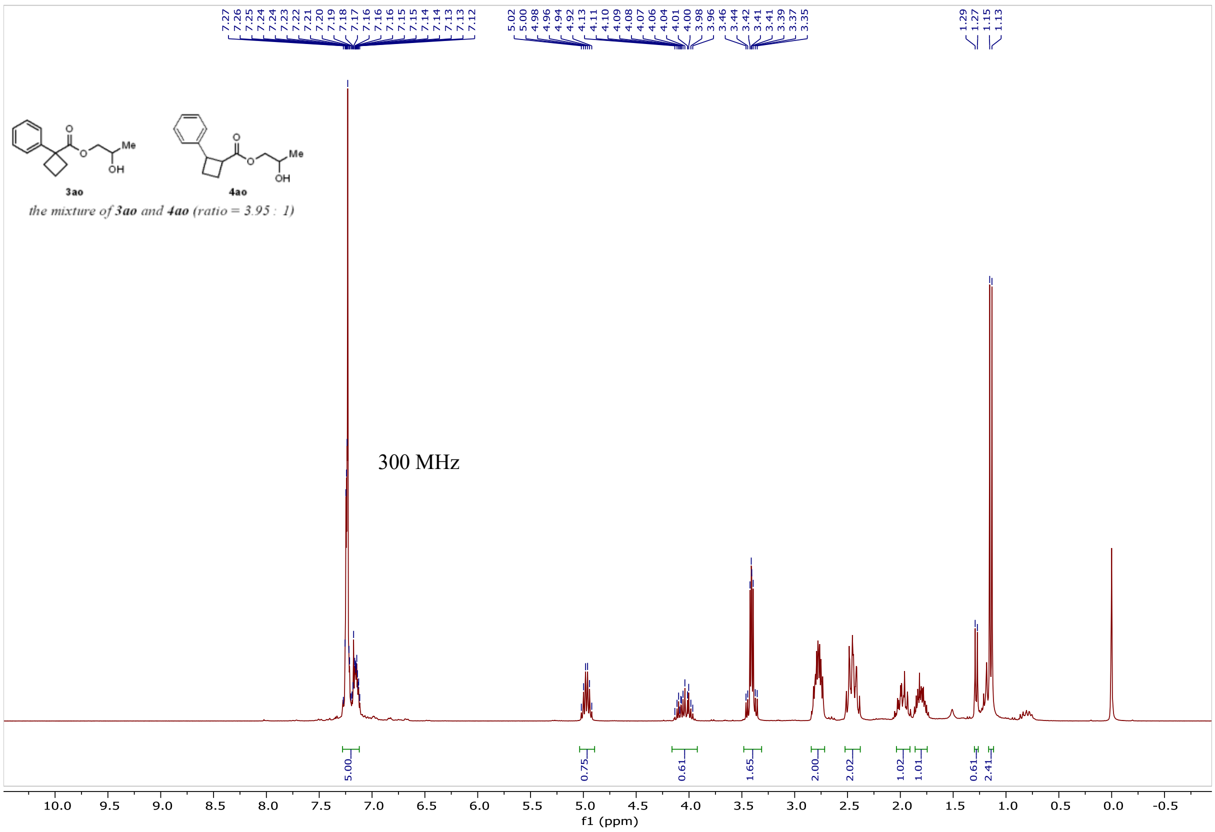

241218.f316.11.fid — Yu-kun Liu LYK-15-450-re — Au13C CDCl3 {C:\Bruker\TopSpin3.6.2} 2412 16

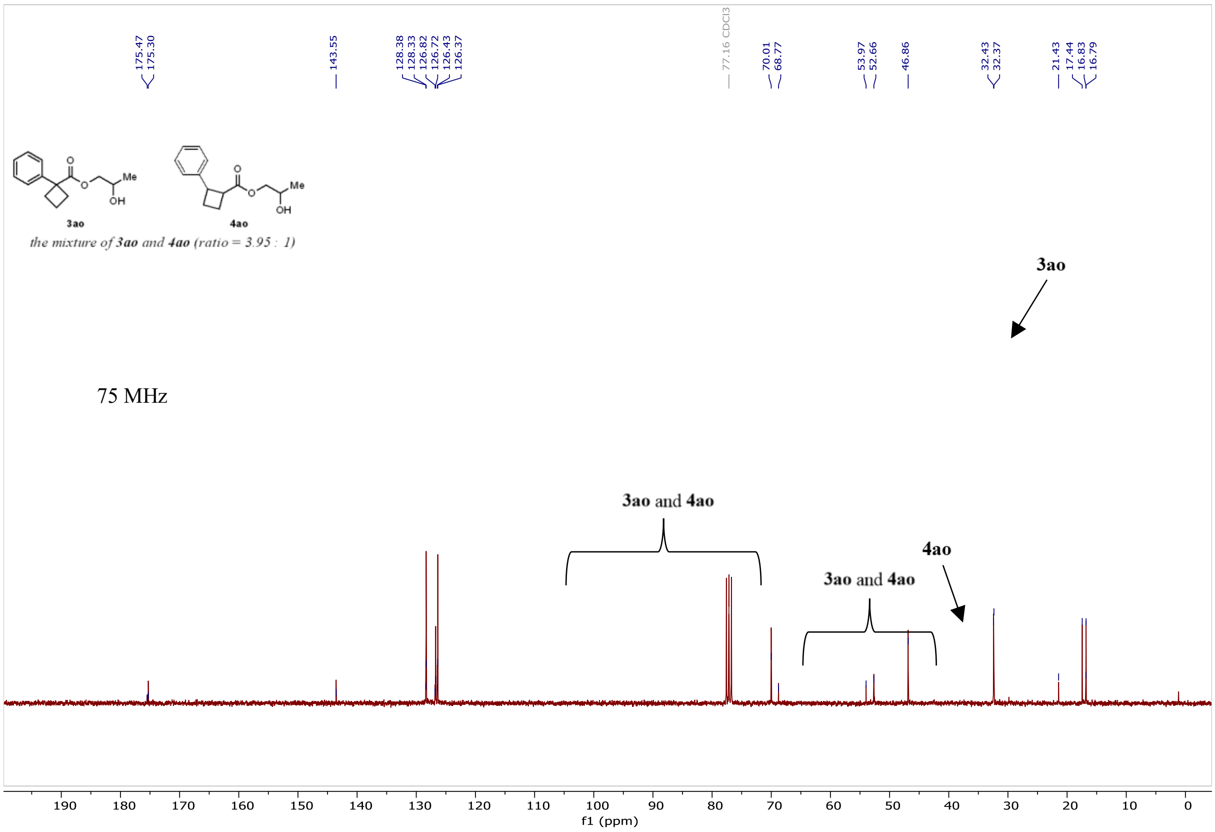

241205.305.10.fid — Yu-Kun Liu LYK-15-2-Syn-OH — Au1H CDCl3 {C:\Bruker\TopSpin3.6.2} 2412 5

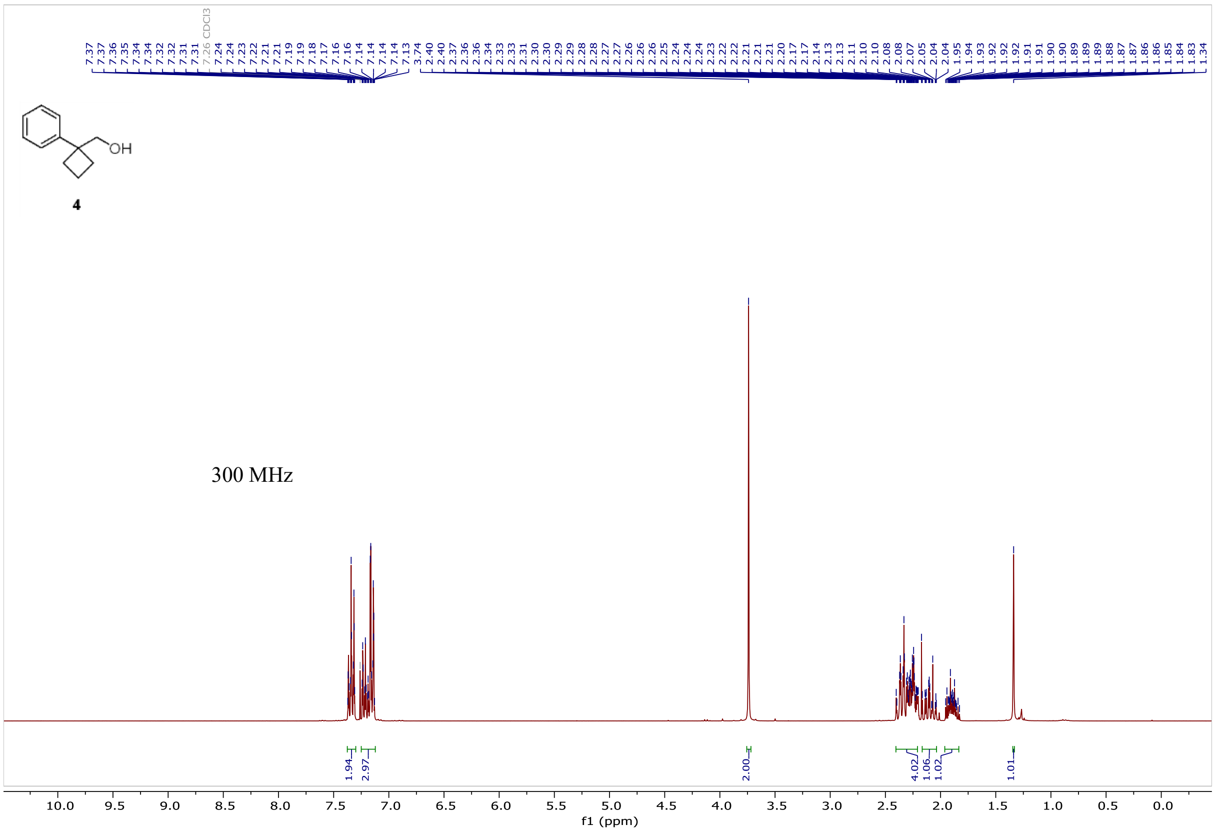

241205.305.11.fid — Yu-Kun Liu LYK-15-2-Syn-OH — Au13C CDCl3 {C:\Bruker\TopSpin3.6.2} 2412 5

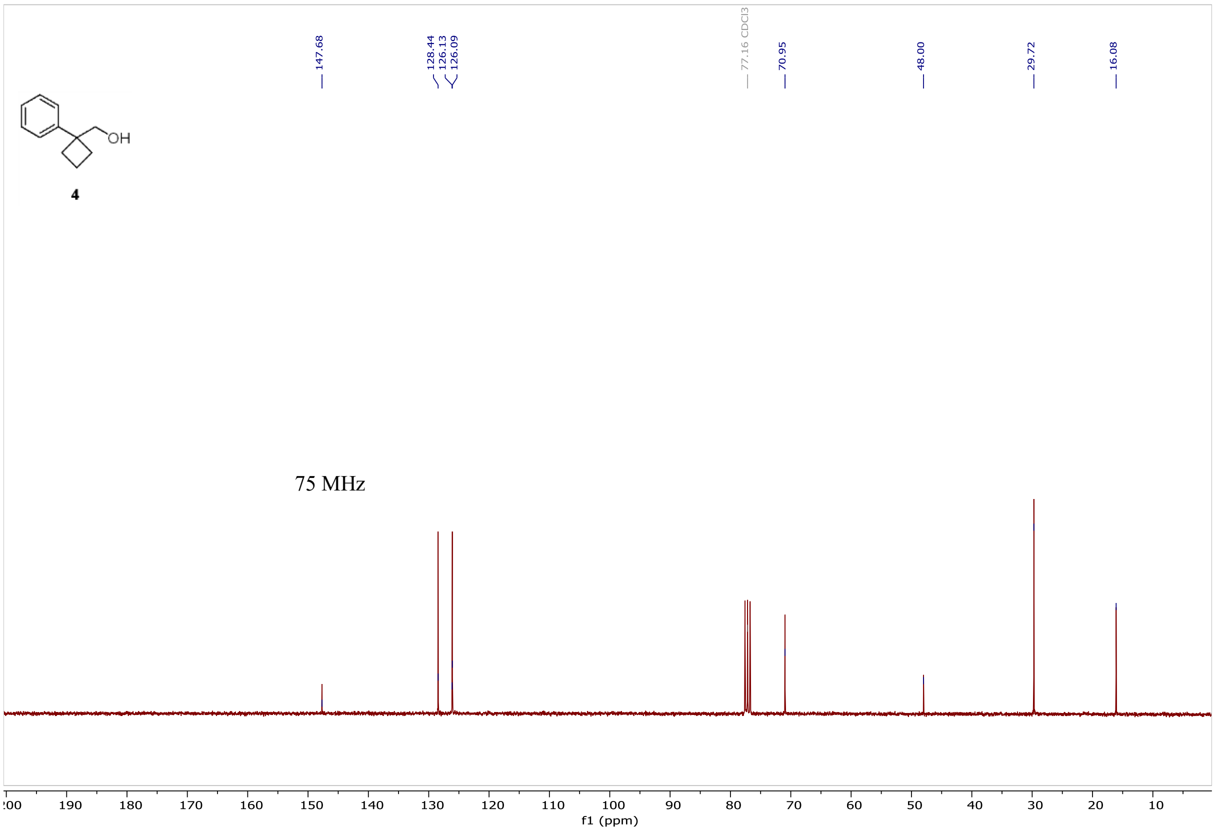

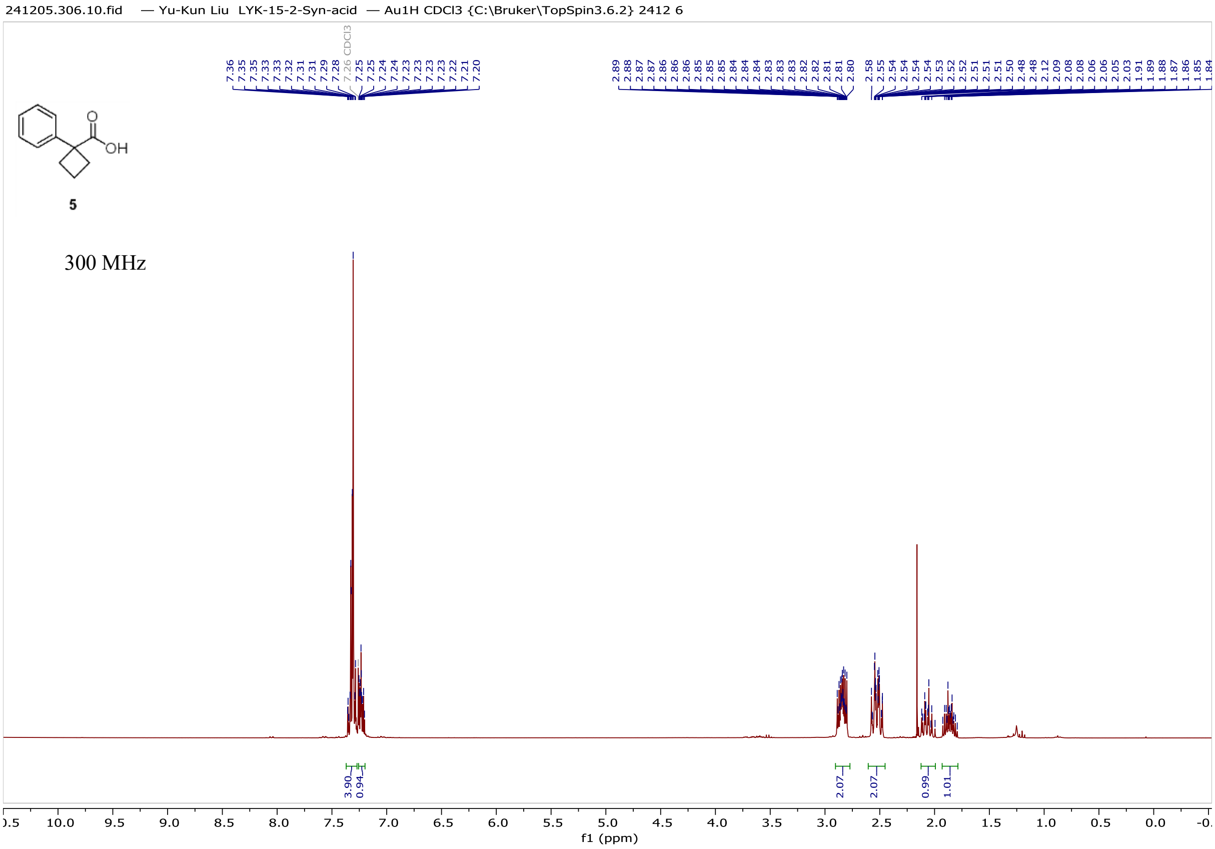
241205.306.10.fid — Yu-Kun Liu LYK-15-2-Syn-acid — Au1H CDCl3 {C:\Bruker\TopSpin3.6.2} 2412 6
7.36 7.35 7.35 7.33 7.33 7.32 7.31 7.31 7.29 7.28 7.26 CDCl3 7.25 7.25 7.24 7.24 7.23 7.23 7.23 7.23 7.22 7.21 7.20
2.89 2.88 2.87 2.87 2.86 2.86 2.85 2.85 2.85 2.84 2.84 2.84 2.83 2.83 2.83 2.82 2.82 2.82 2.81 2.81 2.80
2.58 2.55 2.54 2.54 2.54 2.54 2.53 2.52 2.52 2.51 2.51 2.51 2.50 2.48 2.48 2.12 2.09 2.08 2.08 2.06 2.06 2.05 2.03 1.91 1.89 1.88 1.87 1.86 1.85 1.84
O
OH
5
300 MHz
3.90 0.94 2.07 2.07 0.99 1.01
f1 (ppm)

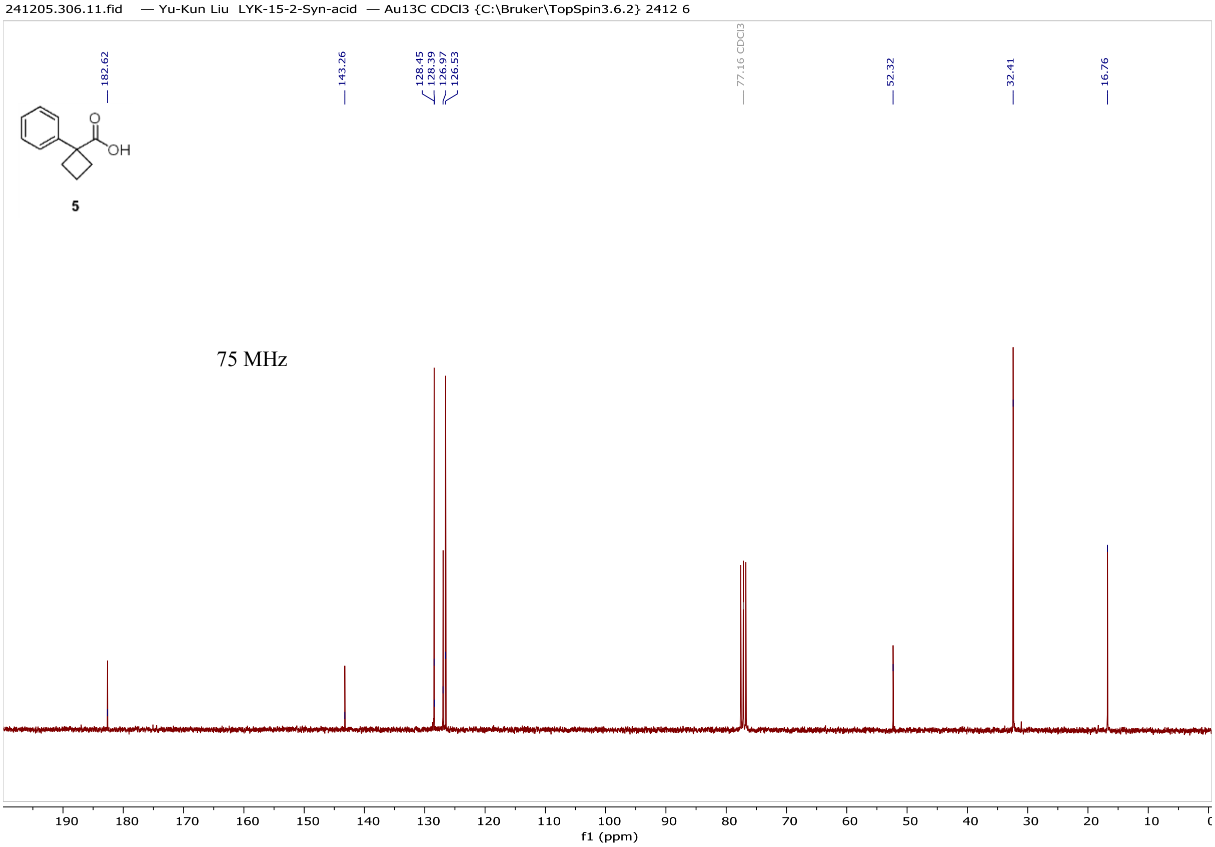
241205.306.11.fid — Yu-Kun Liu LYK-15-2-Syn-acid — Au13C CDCl3 {C:\Bruker\TopSpin3.6.2} 2412 6
182.62
143.26
128.45 128.39 126.97 126.53
77.16 CDCl3
52.32
32.41
16.76
O
OH
5
75 MHz
f1 (ppm)
